# Successive C–C bond cleavage, fluorination, trifluoromethylthio- and pentafluorophenylthiolation under metal-free conditions to provide compounds with dual fluoro-functionalization†
†Electronic supplementary information (ESI) available. CCDC 1415530 and 1415531. For ESI and crystallographic data in CIF or other electronic format see DOI: 10.1039/c5sc04208a


**DOI:** 10.1039/c5sc04208a

**Published:** 2015-12-09

**Authors:** Ibrayim Saidalimu, Shugo Suzuki, Etsuko Tokunaga, Norio Shibata

**Affiliations:** a Department of Nanopharmaceutical Sciences , Nagoya Institute of Technology , Gokiso, Showa-ku , Nagoya 466-8555 , Japan; b Department of Frontier Materials , Nagoya Institute of Technology , Gokiso, Showa-ku , Nagoya 466-8555 , Japan . Email: nozshiba@nitech.ac.jp

## Abstract

The selective C–C bond cleavage and simultaneous formation of two C–F bonds and one C–S bond in β-keto esters with nucleophilic fluorination reagents such as DAST under metal-free/catalyst-free conditions is disclosed. Double fluorination at two remote carbons with additional dialkylamino-sulfenylation provided unique fluorinated compounds in good to high yields. This method can be applied for the successive C–C bond cleavage/fluorination/trifluoromethylthiolation of β-keto esters using trifluoromethyl-DAST (CF_3_-DAST) providing different type of fluorinated and trifluoromethylthiolated compounds *via* a shunt pathway. Doubly fluoro-functionalized compounds obtained in these reactions are unique and difficult to synthesize by other methods.

## Abstract 2

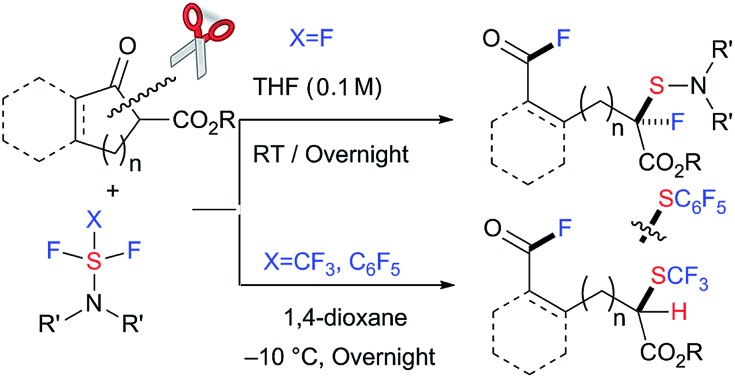
The selective C–C bond cleavage and simultaneous formation of two C–F bonds and one C–S bond with DAST, CF_3_-DAST under metal-free/catalyst-free conditions is disclosed.

## 

The selective cleavage/activation of carbon–carbon (C–C) bonds during chemical transformations poses a significant synthetic challenge in traditional organic synthesis.^1^ Due to the inherent solidity and stability or unreactivity of the C–C bond, this transformation requires harsh conditions. Moreover, following simultaneous chemical transformations, including the formation of new C–X bond(s), the process can be applied to more complex tasks. In recent years, significant achievements and progress have been reported in the area of transition metal catalysis.^2^ However, metal-free conditions to accomplish this, including C–C bond cleavage followed by C–X bond(s) formation, have clear advantages from a green chemistry viewpoint.^3^,^4^ Here we disclose the selective C–C bond cleavage and simultaneous formation of two C–F bonds and one C–S bond in β-keto esters with nucleophilic fluorination reagents such as diethylaminosulfur trifluoride (DAST) under metal- or catalyst-free conditions (Scheme 1). Double fluorination at two remote carbons with additional dialkylamino-sulfenylation provided unique acid fluorides with a tetra-substituted fluorinated/sulfenylated carbon center at a remote position in good to high yields (Scheme 1a). This method can be applied for the successive C–C bond cleavage, fluorination and trifluoromethylthiolation of β-keto esters using trifluoromethyl-DAST (CF_3_-DAST) to provide different types of fluorinated and trifluoromethylthiolated compounds with a tri-substituted carbon center (Scheme 1b). Doubly fluoro-functionalized compounds obtained in these reactions are unique and are difficult to synthesize by other methods. A pentafluorophenyl-thiolated analogue was also synthesized using pentafluorophenyl-DAST (C_6_F_5_-DAST). Our results suggest that unique sequential transformation that provides attractive fluorinated compounds is possible without any state-of-the-art catalyst, energy of the ring-strain or heating. Instead, it simply involves a suitable choice of substrates and reagents.

**Scheme 1:**
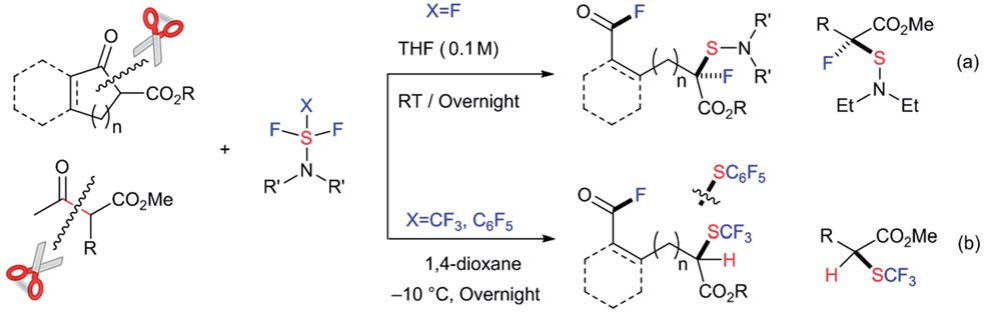
Sequential carbon–carbon bond cleavage, fluorination and fluorination, trifluoromethylthiolation or pentafluorophenylthiolation under a metal-free system.

A large number of commercial applications for fluorinated organic compounds have induced much interest in developing novel synthetic methods to incorporate fluorine or fluorinated groups into organic compounds.^5^ Fluorination (F),^6^ trifluoromethylation (CF_3_),^7^ and trifluoromethylthiolation (SCF_3_)^8^,^9^ reactions are among the three most important chemical transformations investigated in recent years due to the impressive electron-withdrawing effects and lipophilicity of the groups being introduced. While developing novel methodologies for fluoro-functionalization reactions, we unexpectedly transformed ethyl indanone carboxylate (**1a**) with DAST^10^ in CH_2_Cl_2_ to acyclic acid fluoride **2a** with a tetra-substituted carbon center with C–F and C–S bonds. Although the chemical yield was low, only 31%, the reaction was unique enough for further investigation since it effected four important chemical transformations without any catalysis: C–C bond cleavage, the formation of two C–F bonds at remote positions, and a C–S bond.^11^ We thus envisioned that this strategy might be viable for the synthesis of new types of fluoro-functionalized acid fluorides from ubiquitous carboxylic esters. With this idea in mind, we set out to investigate the use of β-keto ester **1a** and DAST.^12^,^13^ After thoroughly surveying reaction conditions, including temperature, solvent, concentration, *etc.* (see ESI, Table S1†), we found that the use of 2.0 equivalents of DAST in THF at room temperature gave the best result (**2a**, 85% yield).

We proceeded to evaluate the scope of these four metal-free, sequential transformations by DAST with a wide variety of β-keto esters **1** (Table 1). The sequential transformation of indanone substrates with DAST was in general independent of the size of the ester moiety (Me, Et, Bn), and a substitution on the benzene ring (MeO, Me, Br, Cl) provided the corresponding products **2a–2g** in good to high yields. Substrate **1h**, which is very rich in electrons, also underwent the same four sequential transformations to give the corresponding product in good yield (**2h**, 54%). Tetralone carboxylate **1i** and benzosuberanone carboxylate **1j** were also good substrates for transformation to furnish the desired products **2i** and **2j** in 30% and 39% yield, respectively. Non-aromatic benzyl 2-oxocyclopentanecarboxylate (**1k**) was also converted to fluorinated-sulfenylated acid fluoride **2k** in 39% yield. Other nucleophilic fluorination reagents such as (MeOCH_2_CH_2_)_2_NSF_3_ (DeoxoFluor®),10c,^14^ 4-morpholinylsulfur trifluoride (Morph-DAST),10a,^15^ and *N*,*N*-dimethylaminosulfur trifluoride (Me-DAST)10*a* were equally effective for these transformations, yielding the corresponding fluorinated dialkylaminosulfenylated acid fluoride products **3a–3c** in moderate to high yields. Finally, this strategy was also effective for an acyclic substrate, methyl 2-benzyl-3-oxobutanoate (**1o**) in DMF at 50 °C to provide the C–C bond cleavage/fluorination/sulfenylation product **2o** in moderate yield, while the acetyl fluoride moiety produced was separated due to its acyclic system. Information gleaned from ^1^H NMR, ^13^C NMR, ^19^F NMR, IR, and mass spectra led to the formulation of a unique fluorinated acid fluoride product, **2**. Finally, the structure of **2** was confirmed unambiguously by single crystal X-ray structure analysis of **2h** (CCDC ; 1415530).†

**Table 1:** Four sequential transformations including C–C bond cleavage, two fluorinations, and sulfenylation of **1** with nucleophilic fluorination reagents^*a*^

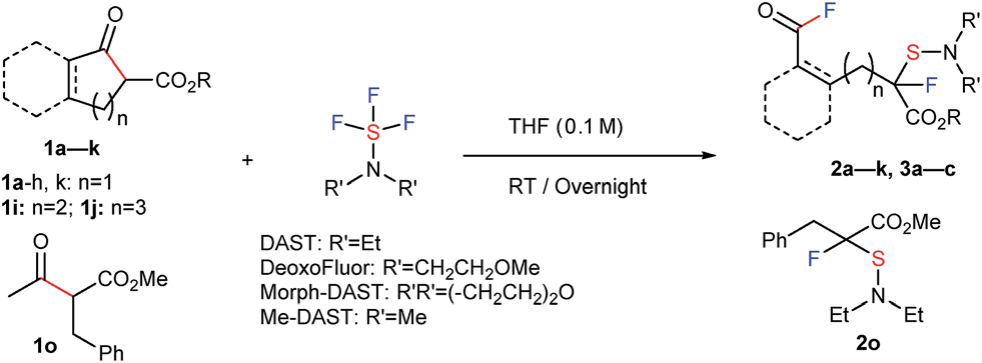
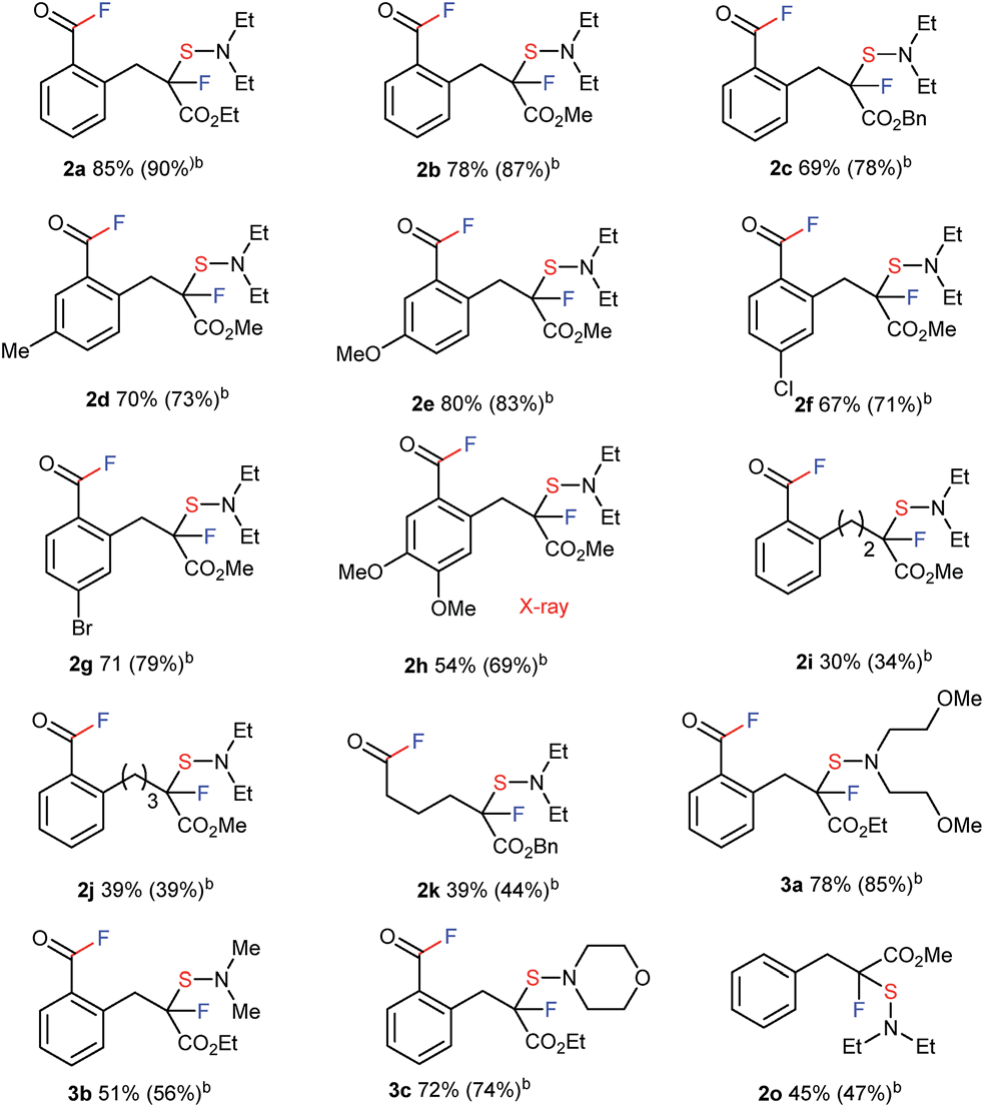

^*a*^The reaction of **1** with DAST or its derivatives (2.0 equiv.) was carried out overnight in THF (0.1 M) at room temperature. Isolated yields are indicated. For detailed reaction conditions, see ESI.

^*b*^ ^19^F NMR yields.

^*c*^The reaction of **1o** with DAST (2.0 equiv.) was carried out overnight in DMF (0.1 M) at 50 °C.

More unexpected supersizing results were obtained when a similar reaction of **1** with trifluoromethyl-DAST^16^ (CF_3_-DAST reagent) was attempted. The CF_3_-DAST reagent was readily prepared by mixing Ruppert–Prakash reagent (CF_3_SiMe_3_) with DAST under basic conditions, but it was not stable enough to be isolated. Thus, we directly used *in situ* generated CF_3_-DAST in CH_2_Cl_2_ instead of DAST for our reaction system with **1a** in THF at room temperature overnight. Acid fluoride **4a** with a trifluoromethylthiolated tri-substituted carbon center was detected in 28% yield. With this result in hand, the reaction conditions, including solvent, temperature, reagent equivalents, *etc.* (see ESI, Tables S2 and S3†), were further optimized. A set of optimal reaction conditions was screened: 2.0 equivalents of CF_3_-DAST (0.5 M mixed in DCM) and overnight reaction at –10 °C in 1,4-dioxane as solvent (up to 61% yield of **4a**). The substrate scope of the reaction is shown in Table 2. A variety of alkyl indanone carboxylates **1** (R = Me, Et, Bn) with different substitutions on the benzene ring (MeO, Me, Br, Cl, di-MeO), tetralone carboxylate **1q**, benzosuberanone carboxylate **1j** and benzyl 2-oxocyclopentanecarboxylate **1k** were employed under the same conditions to provide the corresponding sequential C–C bond cleavage, fluorination or trifluoromethylthiolation products **4a–4k** in moderate to high yields. A sterically demanding secondary ester, tertiary ester, and electron-withdrawing *p*-nitrobenzyl ester (R = iPr, 1-admantanyl, CH_2_C_6_H_4_*p*-NO_2_) were also successfully converted into the desired products **4l–4n** under the same conditions. Acyclic methyl 2-benzyl-3-oxobutanoate **1o** was converted into the desired C–C bond cleavage trifluoromethylthiolation product **4o** in acceptable yield (19%) after the release of the acid fluoride part. The isolated yields are somewhat lower than the NMR yields due to instability during purification by silica-gel chromatography. The structures of the trifluoromethylthiolated acid fluorides were assigned by spectroscopy and clearly determined by X-ray crystallographic analysis of **4h** (CCDC ; 1415531).†

**Table 2:** Three sequential transformations including C–C bond cleavage, fluorination, and trifluoromethylthiolation of **1** with CF_3_-DAST^*a*^

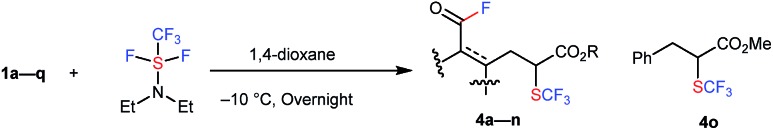
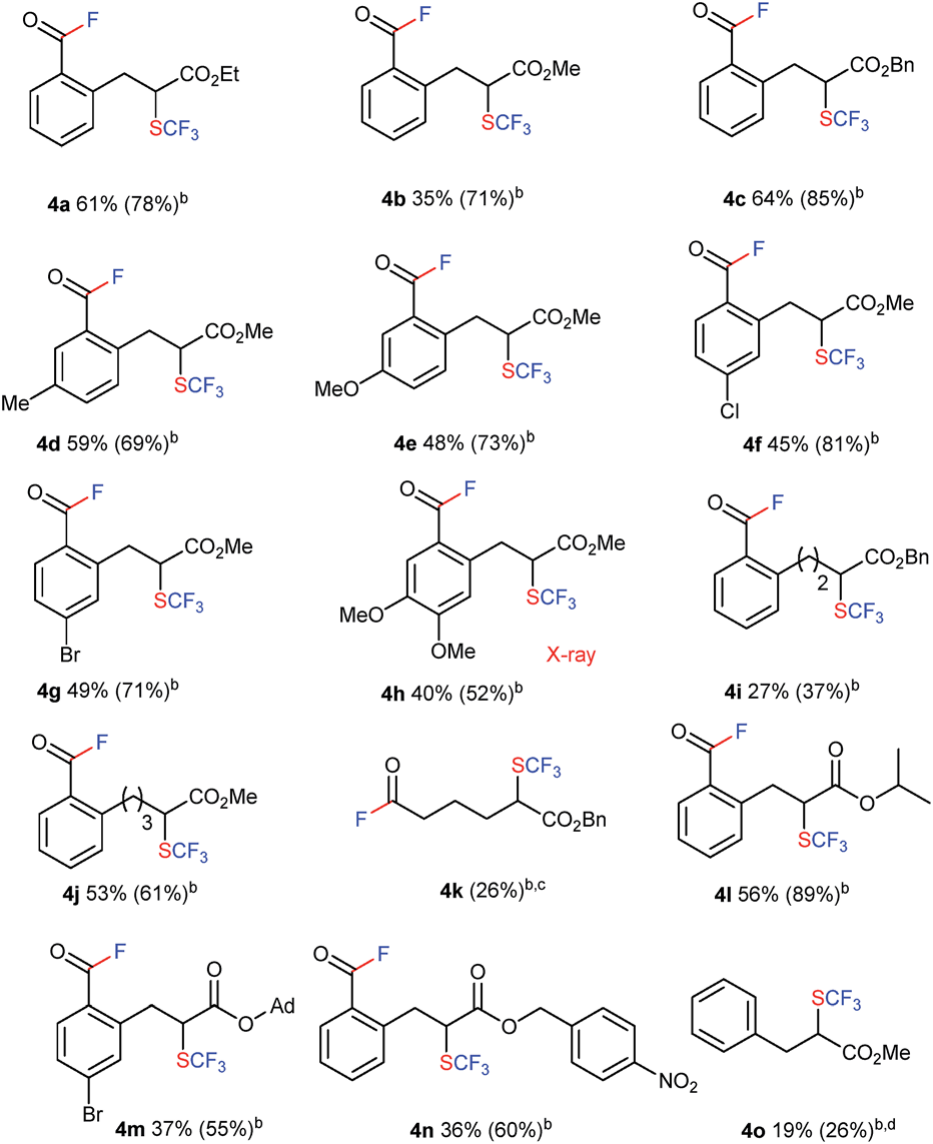

^*a*^The reaction of **1** with 2.0 equivalents of CF_3_-DAST (0.5 M mixture in DCM) was carried out overnight in 1,4-dioxane at –10 °C. Isolated yields are indicated. For detailed reaction conditions, see ESI.

^*b*^ ^19^F NMR yields.

^*c*^ **4k** is too unstable to be isolated after purification.

^*d*^The reaction with 2.0 equivalents of CF_3_-DAST (0.5 M mixture in DCM) was carried out overnight in DMF at 50 °C.

Acid fluorides are versatile building blocks.^17^ In particular, they are popular for peptide coupling reactions without epimerization, and thus a range of more complex fluorinated compounds can be synthesized. As shown in Scheme 2, **2b** and **4b** easily underwent alkylation, amination, and esterification to form the corresponding fluorinated and sulfenylated products **5a,b** and trifluoromethylthiolated products **6a–6c** in good to high yields (Scheme 2).

**Scheme 2:**
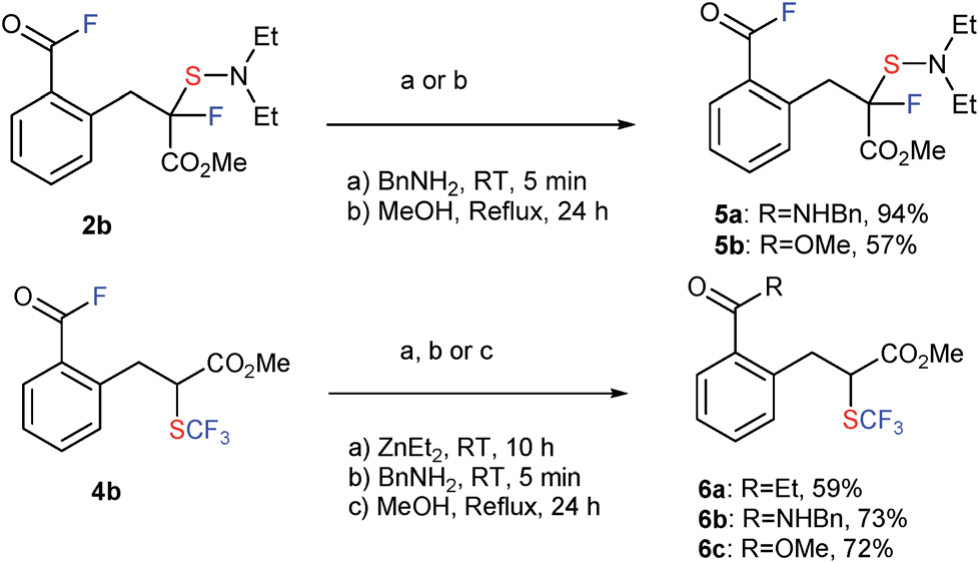
Transformation of acid fluorides **2b** and **4b** to **5a,b** and **6a–6c**.

It is interesting to note that this methodology was effectively extended to the reaction of **1b** with *in situ* generated, previously unknown pentafluorophenyl-DAST (C_6_F_6_-DAST) to provide SC_6_F_5_-analogue **7b** in 53% isolated yield (Scheme 3).

**Scheme 3:**
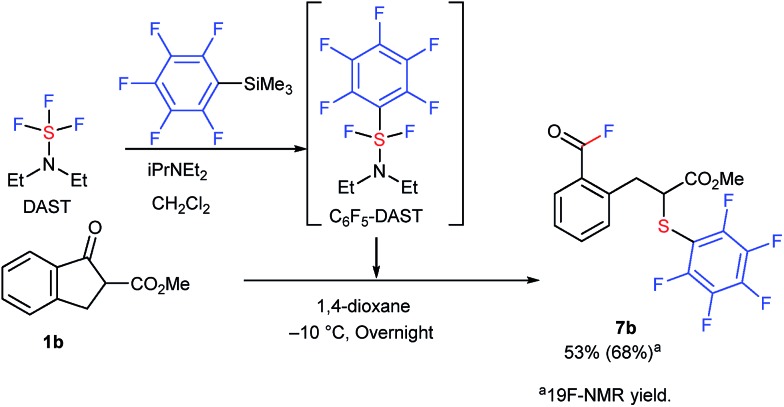
Reaction of **1b** with C_6_F_6_-DAST. Reaction details are shown in ESI.†

Moreover, 1,3-diketone **8** also reacted with DAST or CF_3_-DAST to provide the corresponding unexpected fluorinated or sulfenylated product **9** or trifluoromethylthiolated product **10** in 63% and 54% yield, respectively. Although it was possible to isolate both compounds, **9** was not very stable during silica-gel column chromatography. Deacetylation was observed in this case, similar to the reaction of acyclic substrates **1o** to **2o** or **1o** to **4o** (Scheme 4).

**Scheme 4:**
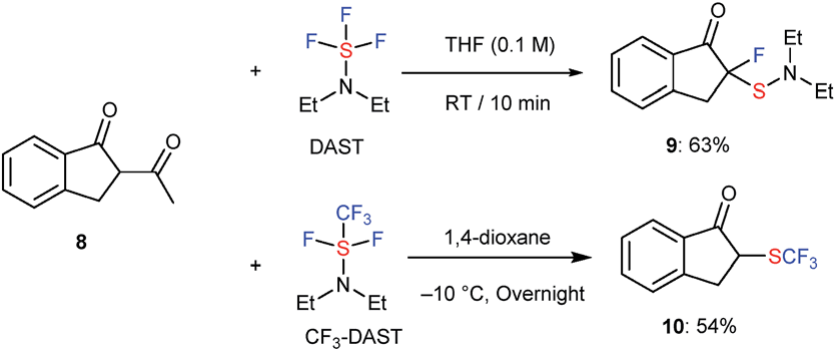
Reaction of 1,3-diketone **8** with DAST or CF_3_-DAST. Reaction details are shown in ESI.†

A possible reaction mechanism (Fig. 1) is based on the unexpected formation of two different types of products **2** and **4**. Initially, the fluorine anion generated from DAST or CF_3_-DAST selectively attacks the ketone moiety of **1a** to give the acid fluoride enolate A *via* a ring-opening reaction through a retro-Dieckmann^18^,^19^ type reaction (for acyclic substrates **2o** and 1,3-diketone **8**, a “retro-Claisen”19*c* type reaction might be suitable due to the de-acetylation). The enolate rapidly attacks the sulfur atom of the DAST or CF_3_-DAST residue providing unstable intermediate B. In the case of the reaction with DAST (X = F), intermediate B promptly releases HF initiated by the attack from the internal nitrogen moiety. This is followed by intramolecular fluoro-Pummerer-type rearrangement^20^ to furnish final product **2a** as an HF salt *via* thionium intermediate C (route a). On the other hand, the reaction with CF_3_-DAST (X = CF_3_) enters route b instead of route a due to the presence of diisopropylethylamine (iPr_2_NEt). CF_3_-DAST should be prepared *in situ* from an equivalent mixture of DAST, CF_3_SiMe_3_, and iPr_2_NEt. A molar equivalent of iPr_2_NEt is crucial for complete transformation to CF_3_-DAST, and iPr_2_NEt is presumably required to initiate the reaction and stabilize the generated CF_3_-DAST.^16^ The acidic proton in intermediate B needs to be removed by iPr_2_NEt to furnish D rather than the elimination of HF before heading into route a. Unstable intermediate D promptly releases HF as an *N*-ethylidene ethanamine salt,^16^ resulting in trifluoromethylthiolation product **4a***via* enolate E.

**Fig. 1:**
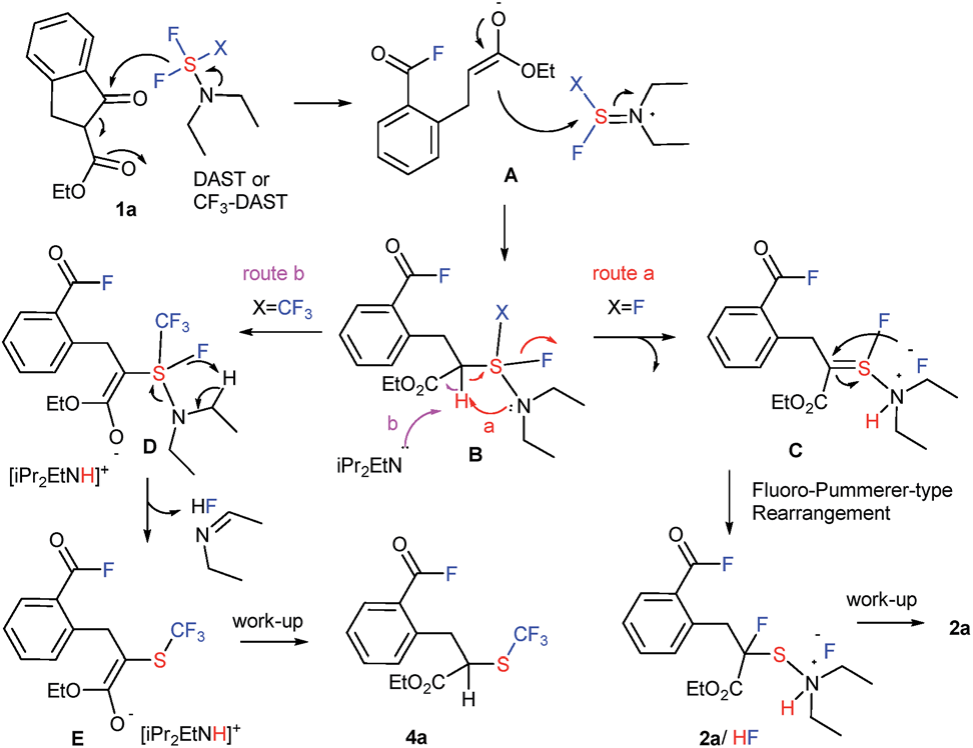
A plausible reaction mechanism.

## Conclusions

In summary, we have efficiently synthesized acid fluorides with a tetra-substituted fluorinated and sulfenylated carbon center at a remote position *via* a metal- or catalyst-free ring opening reaction of β-keto esters with DAST. The chemical transformation undergoes a sequence of C–C bond cleavages, two C–F bonds form at the remote positions of C1 to C5–C6 and C–S bond formation affords a wide range of unique fluorinated acid fluorides in good to high yields under mild reaction conditions. This sequential transformation was extended to the reaction of β-keto esters with CF_3_-DAST. More interestingly, trifluoromethylthiolated acid fluorides with a tri-substituted carbon center were produced under the same reaction conditions. 1,3-Diketones are also acceptable substrates in these transformations with DAST and CF_3_-DAST. All these reactions are triggered by an attack by fluoride on the carbonyl through a retro-Dieckmann or retro-Claisen type reaction. Both fluoro-functionalized compounds unexpectedly obtained here are otherwise difficult to prepare. Although a large number of reactions have been reported using DAST and related reagents with a variety of substrates^10^,^17^,^18^ including β-keto esters,^12^,^13^ the present sequential reaction has never been reported. The reaction mechanism, the utility of this strategy for the development of new chemical transformations and the synthesis of biologically attractive molecules using these fluorinated products are under investigation.

## Supplementary Material

Supplementary information

Crystal structure data
