# Supplementary material for: Successive C–C bond cleavage, fluorination, trifluoromethylthio- and pentafluorophenylthiolation under metal-free conditions to provide compounds with dual fluoro-functionalization
Source: Chem Sci. 2015 Dec 9;7(3):2106–10. doi: 10.1039/c5sc04208a (PMC5968529; doi:10.1039/c5sc04208a)
Supplement: Supplementary file 1 [file SC-007-C5SC04208A-s001.pdf]

Electronic Supplementary Material (ESI) for Chemical Science.
This journal is © The Royal Society of Chemistry 2015

SUPPORTING INFORMATION

Successive C-C bond cleavage, fluorination, trifluoromethylthio- or pentafluorophenylthiolation under metal-free conditions to provide compounds with dual fluoro-functionalization

Ibrayim Saidalimu,[a] Shugo Suzuki,[b] Etsuko Tokunaga,[a] and Norio Shibata *[a,b]

[a]*Department of Nanopharmaceutical Sciences, Nagoya Institute of Technology, Gokiso, Showa-ku, Nagoya 466-8555, Japan.*

[b]*Dpartment of Frontier Materials, Nagoya Institute of Technology, Gokiso, Showa-ku, Nagoya 466-8555, Japan.*

Correspondence and requests for materials should be addressed to N.S. (e-mail: nozshiba@nitech.ac.jp.)

Table of Contents

1. Experimental Section ... 2
1.1 General Methods ... 2
1.2 Preparation of 4-nitrobenzyl 1-oxo-2,3-dihydro-1H-indene-2-carboxylate (1n); ... 3
2. Optimization of ring-opening reaction condition (mono-fluorination) ... 4
3. General procedure and product characterization data for 2a—2k, 2o and 3a—3c; ... 4
4. X-ray crystallography data for the 2h (CCDC 1415530) ... 15
5. Optimization of ring-opening reaction condition (SCF_3) ... 17
6. Typical procedure preparation of CF_3-DAST reagents ... 19
7. General procedure and product characterization data for 4a—4o; ... 19
8. X-ray crystallography data for the 4h (CCDC 1415531) ... 30
9. General procedure and product characterization data for 5a; ... 32
10. General procedure and product characterization data for 5b; ... 32
11. General procedure and product characterization data for 6a; ... 33
12. General procedure and product characterization data for 6b; ... 34
13. General procedure and product characterization data for 6c; ... 35
14. Typical procedure preparation of C_6F_5-DAST reagents ... 36
15. General procedure and product characterization data for 7b; ... 36
16. General procedure and product characterization data for 9; ... 37
17. General procedure and product characterization data for 10; ... 38
18. References ... 38
19. ^{1}H NMR and ^{13}C NMR spectra for starting material 1n; ... 40
20. ^{1}H NMR, ^{13}C NMR and ^{19}F NMR spectra for doubly fluoro-functionalization compounds 2a—2k, 2o and 3a—3c (Table 1) ... 42
21. ^{1}H NMR, ^{13}C NMR and ^{19}F NMR spectra for doubly fluoro-functionalization compounds 4a—4o (Table 2) ... 88
22. ^{1}H NMR, ^{13}C NMR and ^{19}F NMR spectra for transformation of acid fluorides 2b and 4b to ketones 5a, 6a amides 5b, 6b and ester 6c (Scheme 2); ... 132
23. ^{1}H NMR, ^{13}C NMR and ^{19}F NMR spectra for product 7b (Scheme 3) ... 148
24. ^{1}H NMR, ^{13}C NMR and ^{19}F NMR spectra for product 9 and 10 (Scheme 4); ... 151

1. Experimental Section

1.1 General Methods

All reactions were performed in oven-dried glassware under a positive pressure of nitrogen. Solvents were transferred via syringe and were introduced into the reaction vessels though a rubber septum. All reactions were monitored by thin-layer chromatography (TLC) carried out on 0.25 mm Merck silica-gel (60-F254). The TLC plates were visualized with UV light and 7% phosphomolybdic acid or $KMnO_4$ in water/heat. Column chromatography was carried out on a column packed with silica-gel 60N spherical neutral size 63—210 μm. The ^{1}H-NMR (500 MHz or 400 MHz), ^{19}F-NMR (282 MHz), ^{13}C-NMR (125.7 MHz) spectra for solution in $CDCl_3$ were recorded on a Buruker Avance 500, Buruker Avance 400 and a Varian Mercury 300. Chemical shifts (δ) are expressed in ppm downfield from internal TMS (δ = 0.00). The C_6F_6 [δ = −162.2 ($CDCl_3$)] was used as internal standard for ^{19}F NMR. Mass spectra were recorded on a SHIMADZU GCMS-QP5050A (EI-MS) and SHIMAZU LCMS-2020 (ESI-MS). High resolution mass spectrometry were recorded on a Waters Synapt G2 HDMS (ESI-MS). Infrared spectra were recorded on a JASCO FT/IR-4100 spectrometer. Melting point were recorded on a BUCHI M-565. The balance used is ATX-224 (Shimadzu Corporation); Minimum Display 0.1 mg; Repeatability (Standard Deviation); 0.1 mg; Linearity error ±0.2 mg. All solvents were dried and distilled before use. Chemist Plaza CP-170 (SIBATA SCIENTIFIC TECHNOLOGY LTD.) for heating and cooling was used for all the reaction.

DAST [(Diethylamino)sulfur Trifluoride] (Purity: >90.0%, Product Number; D1868), Methoxy-DAST [Bis(2-methoxyethyl)aminosulfur Trifluoride] (Purity: >90.0%, Product Number; B2440) and Morph-DAST (Morpholinosulfur Trifluoride) (Purity: >93.0%, Product Number; M1573) were purchased from Tokyo Chemical Industry Co., Ltd., Japan. Methyl-DAST (Dimethylaminosulfur trifluoride) was purchased from Aldrich, USA. (Purity: >95.0%, Product Number; 248215).

The *β*-keto esters **1a—m** and **1o—1q** are known compounds, and all these compounds were synthesized according to the literature procedures.[1—5]

1.2 Preparation of 4-nitrobenzyl 1-oxo-2,3-dihydro-1H-indene-2-carboxylate (1n);

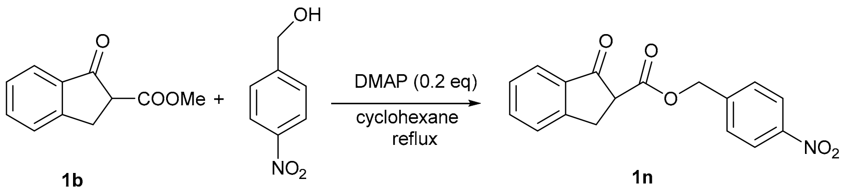

To a flask equipped with a Dean-Stark trap and reflux condenser was added methyl 1-oxo-2,3-dihydro-1H-indene-2-carboxylate **1b** (0.5 g, 2.6 mmol), (4-nitrophenyl)methanol (0.6 g, 3.9 mmol), DMAP (0.06 g, 0.52 mmol) and cyclohexane (4 mL). The mixture was heated to reflux, distilling the methanol formed during the reaction. The mixture was refluxed until complete conversion was observed by TLC, then concentrated under reduced pressure and the crude product was purified by flash column chromatography (Hex: AcOEt = 8:1—3:1) to provide the title compound **1n** as a white solid in 45% yield (0.36 g). This compound was observed as a mixture of two isomers (keto- and enol forms) in the NMR. mp: 97.9—99.1 °C; ^{1}H NMR (400 MHz, $CDCl_3$) δ 10.41 (s, 1H), 8.22—8.27 (m, 4H), 7.79 (d, 1H, *J* = 8.4 Hz), 7.63—7.68 (m, 2H), 7.57—7.59 (m, 4H), 7.50—7.53 (m, 1H), 7.40—7.48 (m, 4H), 5.26—5.40 (m, 4H), 3.83 (dd, 1H, *J* = 8.4, J = 4.0 Hz), 3.57—3.62 (m, 3H), 3.41 (dd, 1H, *J* = 17.2, J = 8.0 Hz); ^{13}C NMR (75.5 MHz, $CDCl_3$) δ 199.0, 168.7, 153.5, 147.7, 143.5, 142.9, 136.6, 135.7, 135.0, 129.9, 128.2, 128.0, 127.0, 126.6, 124.9, 124.8, 123.9, 121.0, 101.1, 65.7, 64.2, 53.2, 32.5, 30.1; IR (KBr): 3283, 1658, 1573, 1514, 1341, 1270, 1218, 1180, 759 cm^{-1}; HRMS (ESI): Calcd. for $C_{17}H_{13}NNaO_5$ $[M+H]^+$: 334.0691; Found: 334.0696.

2. Optimization of ring-opening reaction condition (mono-fluorination)

Table S1. Optimization of ring-opening reaction condition[a]

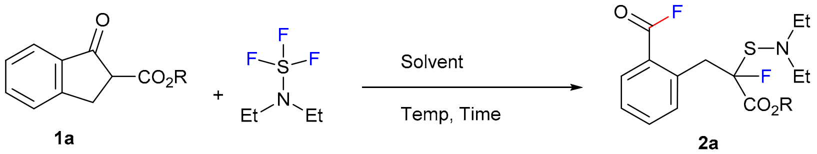

Entry	Solvent	DAST (eq)	Temp (°C)	Time	**2a** (%)[b]
1	DMF	2.0	RT	Overnight	54
2	DMF	1.0	RT	Overnight	30
3	DMF	3.0	RT	Overnight	50
4	DMF	4.0	RT	Overnight	54
5	DMF	2.0	50	Overnight	36
6	CH_2Cl_2	2.0	RT	Overnight	31
7	toluene	2.0	RT	Overnight	14
**8**	**THF**	**2.0**	RT	**Overnight**	**85**
9	EtOH	2.0	RT	Overnight	NR
**10**	**CH_3CN**	**2.0**	RT	**Overnight**	**85**
11	THF	1.5	RT	Overnight	71
12	CH_3CN	1.5	RT	Overnight	66
13[c]	THF	2.0	RT	Overnight	60

[a]The reaction of **1a** with DAST was carried out in different solvents and at different temperature. For detailed reaction conditions, see the table S1. [b]Yield was determined by ^{19}F NMR spectroscopy with an internal standard as C_6H_5F. [c] Following the general procedure, and use 1.0 eq of base (DIEA).

3. General procedure and product characterization data for 2a—2k, 2o and 3a—3c;

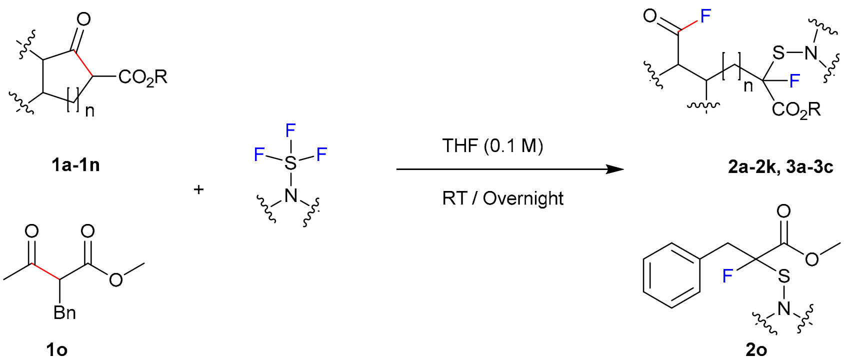

A flame-dried vessel was successively charged, under nitrogen, with *β*-keto esters **1a—1k** and **1o** (0.1 mmol, 1.0 equiv) and anhydrous THF (1.0 mL). The solution was cooled to 0 °C and the DAST (0.2 mmol, 2.0 equiv) was added slowly by syringe. Then the reaction mixture was stirred at room temperature for overnight, quenched by addition of water (10 mL), extracted with ethyl acetate (3 x 20 mL), dried over with Na_2SO_4 and then concentrated in vacuo. The crude product was purified by flash column chromatography to provide the title compound **2a—2k, 2o and 3a—3c**.

3.1 Ethyl 2-((diethylamino)thio)-2-fluoro-3-(2-(fluorocarbonyl)phenyl)propanoate (2a);

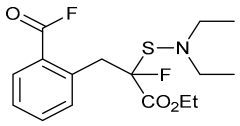

Following the general procedure, using ethyl 1-oxo-2,3-dihydro-1*H*-indene-2-carboxylate **1a** (20.4 mg, 0.1 mmol, 1.0 equiv) and DAST (30 μL, 0.2 mmol, 2.0 equiv) in THF (1 mL), the reaction mixture was stirred at room temperature for overnight. The crude product was purified by flash column chromatography (Hex: AcOEt = 12:1) to provide the title compound **2a** as a yellow oil in 85% yield (29.6 mg). ^{1}H NMR ($CDCl_3$, 500 MHz) δ: 7.96 (dd, 1H, *J* = 8.0 Hz, *J* = 1.0 Hz), 7.54—7.58 (m, 1H), 7.38—7.42 (m, 2H), 4.12—4.23 (m, 2H), 3.94 (dd, 1H, *J* = 15.0 Hz, *J* = 11.0 Hz), 3.79 (dd, 1H, *J* = 25.5 Hz, *J* = 14.5 Hz), 3.02—3.08 (m, 4H), 1.25 (t, 3H, *J* = 7.2 Hz), 1.10 (t, 6H, *J* = 7.0 Hz); ^{19}F NMR ($CDCl_3$, 282 MHz) δ: +31.3 (s, 1F), –140.1 (dd, 1F, *J* = 25.6 Hz, *J* = 10.7 Hz); ^{13}C NMR ($CDCl_3$, 125.7 MHz) δ: 168.3 (d, *J* = 31.3 Hz), 156.9 (d, *J* = 346.6 Hz), 137.8 (d, *J* = 6.0 Hz), 134.2,

132.7 (d, $J = 3.0$ Hz), 132.4 (d, $J = 1.2$ Hz), 127.9, 125.7 (d, $J = 57.3$ Hz), 107.2 (d, $J = 234.8$ Hz), 62.3, 52.3, 36.8 (d, $J = 21.8$ Hz), 14.1, 13.7; IR (NaCl): 2985, 2924, 2854, 1725, 1696, 1450, 1261, 715, 531 cm^{-1}; HRMS (ESI): Calcd. for $C_{16}H_{22}F_2NO_3S$ $[M+H]^+$: 346.1288; Found: 346.1291.

3.2 Methyl 2-((diethylamino)thio)-2-fluoro-3-(2-(fluorocarbonyl)phenyl)propanoate (2b);

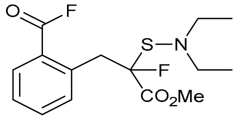

Following the general procedure, using methyl 1-oxo-2,3-dihydro-1H-indene-2-carboxylate **1b** (19.0 mg, 0.1 mmol, 1.0 equiv) and DAST (30 μL, 0.2 mmol, 2.0 equiv) in THF (1 mL), the reaction mixture was stirred at room temperature for overnight. The crude product was purified by flash column chromatography (Hex: AcOEt = 12:1) to provide the title compound **2b** as a yellow oil in 78% yield (25.8 mg). ^{1}H NMR ($CDCl_3$, 500 MHz) δ: 7.96 (dd, 1H, $J = 8.5$ Hz, $J = 1.5$ Hz), 7.55—7.58 (m, 1H), 7.39—7.42 (m, 2H), 3.94 (dd, 1H, $J = 14.5$ Hz, $J = 10.5$ Hz), 3.79 (dd, 1H, $J = 25.0$ Hz, $J = 14.5$ Hz), 3.74 (s, 3H), 2.96—3.03 (m, 4H), 1.09 (t, 6H, $J = 7.2$ Hz); ^{19}F NMR ($CDCl_3$, 282 MHz) δ: +31.4 (s, 1F), –140.4 (dd, 1F, $J = 23.6$ Hz, $J = 9.5$ Hz); ^{13}C NMR ($CDCl_3$, 125.7 MHz) δ: 168.7 (d, $J = 31.4$ Hz), 156.9 (d, $J = 346.9$ Hz), 137.7 (d, $J = 6.2$ Hz), 134.3, 132.7 (d, $J = 3.7$ Hz), 132.4 (d, $J = 1.2$ Hz), 127.9, 125.7 (d, $J = 57.8$ Hz), 107.3 (d, $J = 235.0$ Hz), 52.9, 52.3, 36.8 (d, $J = 22.6$ Hz), 13.7; IR (NaCl): 2973, 2931, 2858, 1812, 1758, 1237, 1119, 756, 704 cm^{-1}; HRMS (ESI): Calcd. for $C_{15}H_{19}F_2NNaO_3S$ $[M+Na]^+$: 354.0951; Found: 354.0955.

3.3 Benzyl 2-((diethylamino)thio)-2-fluoro-3-(2-(fluorocarbonyl)phenyl)propanoate (2c);

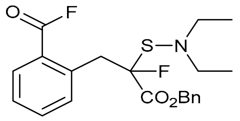

Following the general procedure, using benzyl 1-oxo-2,3-dihydro-1H-indene-2-carboxylate **1c** (26.6 mg, 0.1 mmol, 1.0 equiv) and

DAST (30 μL, 0.2 mmol, 2.0 equiv) in THF (1 mL), the reaction mixture was stirred at room temperature for overnight. The crude product was purified by flash column chromatography (Hex: AcOEt = 10:1) to provide the title compound **2c** as a yellow oil in 69% yield (28.1 mg). ^{1}H NMR ($CDCl_3$, 500 MHz) δ: 7.92 (dd, 1H, J = 8.0 Hz, J = 1.5 Hz), 7.39—7.43 (m, 1H), 7.29—7.36 (m, 6H), 7.23—7.26 (m, 1H), 5.20, 5.11 (AB quartet, J=12.5 Hz, 2H), 3.98 (dd, 1H, J = 14.5 Hz, J = 10.0 Hz), 3.73 (dd, 1H, J = 25.5 Hz, J = 14.5 Hz), 2.83—3.06 (m, 4H), 1.04 (s, 6H); ^{19}F NMR ($CDCl_3$, 282 MHz) δ: +31.1 (s, 1F), –140.4 (dd, 1F, J = 25.6 Hz, J = 9.6 Hz); ^{13}C NMR ($CDCl_3$, 125.7 MHz) δ: 168.2 (d, J = 31.4 Hz), 156.8 (d, J = 346.9 Hz), 137.7 (d, J = 6.2 Hz), 134.9, 134.3, 132.6 (d, J = 3.7 Hz), 132.4 (d, J = 1.2 Hz), 128.9, 128.7, 128.6, 127.8, 125.6 (d, J = 57.8 Hz), 107.2 (d, J = 235.0 Hz), 67.8, 52.3, 36.8 (d, J = 21.3 Hz), 13.7; IR (NaCl): 2970, 2935, 2873, 1811, 1749, 1236, 1002, 741, 698 cm^{-1}; HRMS (ESI): Calcd. for $C_{21}H_{23}F_2NNaO_3S$ $[M+Na]^+$: 430.1264; Found: 430.1257.

3.4 Methyl 2-((diethylamino)thio)-2-fluoro-3-(2-(fluorocarbonyl)-4-methylphenyl)propanoate (2d);

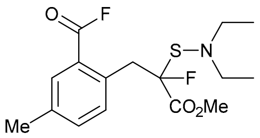

Following the general procedure, using methyl 6-methyl-1-oxo-2,3-dihydro-1H-indene-2-carboxylate **1d** (20.2 mg, 0.1 mmol, 1.0 equiv) and DAST (30 μL, 0.2 mmol, 2.0 equiv) in THF (1 mL), the reaction mixture was stirred at room temperature for overnight. The crude product was purified by flash column chromatography (Hex: AcOEt = 12:1) to provide the title compound **2d** as a yellow oil in 70% yield (24.2 mg). ^{1}H NMR ($CDCl_3$, 500 MHz) δ: 7.76 (d, 1H, J = 1.5 Hz), 7.36 (dd, 1H, J = 8.0 Hz, J = 1.5 Hz), 7.26—7.28 (m, 1H), 3.88 (dd, 1H, J = 14.5 Hz, J = 11.0 Hz), 3.70—3.78 (m, 4H), 2.97—3.02 (m, 4H), 2.37 (s, 3H), 1.09 (t, 6H, J = 7.0 Hz); ^{19}F NMR ($CDCl_3$, 282 MHz) δ: +31.3 (s, 1F), –140.3 (dd, 1F, J = 24.5 Hz, J = 10.8 Hz); ^{13}C NMR ($CDCl_3$, 125.7 MHz) δ: 168.8 (d, J = 31.6 Hz), 157.1

(d, J = 346.9 Hz), 137.9, 135.1, 134.5 (d, J = 5.0 Hz), 132.9, 132.6 (d, J = 3.7 Hz), 125.4 (d, J = 56.5 Hz), 107.4 (d, J = 234.3 Hz), 52.9, 52.3, 36.5 (d, J = 21.8 Hz), 20.9, 13.7; IR (NaCl): 2973, 2927, 2870, 1817, 1756, 1259, 1175, 1015, 764, 728 cm^{-1}; HRMS (ESI): Calcd. for $C_{16}H_{21}F_2NNaO_3S$ $[M+Na]^+$: 368.1108; Found: 368.1100.

3.5 Methyl 2-((diethylamino)thio)-2-fluoro-3-(2-(fluorocarbonyl)-4-methoxyphenyl)propanoate (2e);

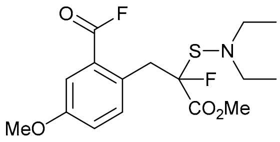

Following the general procedure, using methyl 6-methoxy-1-oxo-2,3-dihydro-1H-indene-2-carboxylate **1e** (22.0 mg, 0.1 mmol, 1.0 equiv) and DAST (30 μL, 0.2 mmol, 2.0 equiv) in THF (1 mL), the reaction mixture was stirred at room temperature for overnight. The crude product was purified by flash column chromatography (Hex: AcOEt = 10:1) to provide the title compound **2e** as a yellow oil in 80% yield (28.9 mg). ^{1}H NMR ($CDCl_3$, 500 MHz) δ: 7.44 (d, 1H, J = 2.5 Hz), 7.29 (d, 1H, J = 8.5 Hz), 7.09 (dd, 1H, J = 8.5 Hz, J = 2.5 Hz), 3.82—3.87 (m, 4H), 3.67—3.75 (m, 4H), 2.95—3.06 (m, 4H), 1.09 (t, 6H, J = 7.0 Hz); ^{19}F NMR ($CDCl_3$, 282 MHz) δ: +31.3 (s, 1F), –140.3 (dd, 1F, J = 24.8 Hz, J = 10.7 Hz); ^{13}C NMR ($CDCl_3$, 125.7 MHz) δ: 168.8 (d, J = 31.6 Hz), 158.8, 156.7 (d, J = 347.4 Hz), 133.9 (d, J = 3.7 Hz), 129.3 (d, J = 5.0 Hz), 126.4 (d, J = 56.5 Hz), 120.3, 117.0, 107.5 (d, J = 234.0 Hz), 55.6, 52.8, 52.3, 36.5 (dd, J = 21.7 Hz, J = 1.2 Hz), 13.7; IR (NaCl): 2970, 2931, 2858, 1813, 1755, 1507, 1267, 1004, 760, 724 cm^{-1}; HRMS (ESI): Calcd. for $C_{16}H_{21}F_2NNaO_4S$ $[M+Na]^+$: 384.1057; Found: 384.1071.

3.6 Methyl 3-(4-chloro-2-(fluorocarbonyl)phenyl)-2-((diethylamino)thio)-2-fluoropropanoate (2f);

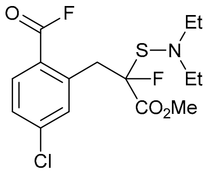

Following the general procedure, using methyl 6-chloro-1-oxo-2,3-dihydro-1H-indene-2-carboxylate **1f** (22.6 mg, 0.1 mmol, 1.0 equiv) and DAST (30 μL, 0.2 mmol, 2.0 equiv) in THF (1 mL), the reaction mixture was stirred at room temperature for overnight. The crude product was purified by flash column chromatography (Hex: AcOEt = 12:1) to provide the title compound **2f** as a yellow oil in 67% yield (24.5 mg). ^{1}H NMR ($CDCl_3$, 500 MHz) δ: 7.90 (d, 1H, *J* = 8.5 Hz), 7.38—7.40 (m, 2H), 3.91 (dd, 1H, *J* = 14.5 Hz, *J* = 11.0 Hz), 3.72—3.80 (m, 4H), 2.97—3.03 (m, 4H), 1.10 (t, 6H, *J* = 7.0 Hz); ^{19}F NMR ($CDCl_3$, 282 MHz) δ: +31.4 (s, 1F), –140.3 (dd, 1F, *J* = 23.6 Hz, *J* = 10.7 Hz); ^{13}C NMR ($CDCl_3$, 125.7 MHz) δ: 168.5 (d, *J* = 31.1 Hz), 156.1 (d, *J* = 345.8 Hz), 140.9, 139.7 (d, *J* = 6.2 Hz), 133.6, 132.9 (d, *J* = 2.7 Hz), 128.3, 124.0 (d, *J* = 58.8 Hz), 107.0 (d, *J* = 235.0 Hz), 53.0, 52.3, 36.6 (d, *J* = 21.7 Hz), 13.7; IR (NaCl): 2973, 2933, 2868, 1815, 1757, 1236, 1005, 769, 698 cm^{-1}; HRMS (ESI): Calcd. for $C_{15}H_{18}ClF_2NNaO_3S$ $[M+Na]^+$: 388.0562; Found: 388.0574.

3.7 Methyl 3-(4-bromo-2-(fluorocarbonyl)phenyl)-2-((diethylamino)thio)-2-fluoropropanoate (2g);

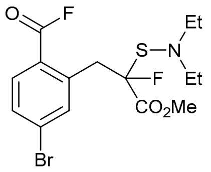

Following the general procedure, using methyl 6-bromo-1-oxo-2,3-dihydro-1H-indene-2-carboxylate **1g** (26.9 mg, 0.1 mmol, 1.0 equiv) and DAST (30 μL, 0.2 mmol, 2.0 equiv) in THF (1 mL), the reaction mixture was stirred at room temperature for overnight. The crude product was purified by flash column chromatography (Hex: AcOEt = 12:1) to provide the title compound **2g**

as a yellow oil in 71% yield (29.0 mg). ^{1}H NMR ($CDCl_3$, 500 MHz) δ: 7.81 (d, 1H, J = 8.0 Hz), 7.54—7.56 (m, 2H), 3.91 (dd, 1H, J = 14.5 Hz, J = 10.5 Hz), 3.70—3.78 (m, 4H), 2.97—3.03 (m, 4H), 1.10 (t, 6H, J = 7.0 Hz); ^{19}F NMR ($CDCl_3$, 282 MHz) δ: +31.4 (s, 1F), –140.3 (dd, 1F, J = 24.5 Hz, J = 10.7 Hz); ^{13}C NMR ($CDCl_3$, 125.7 MHz) δ: 168.3 (d, J = 31.1 Hz), 156.3 (d, J = 345.9 Hz), 139.5 (d, J = 6.2 Hz), 135.7 (d, J = 2.4 Hz), 133.4, 131.2, 129.4, 124.4 (d, J = 58.9 Hz), 106.9 (d, J = 235.0 Hz), 52.9, 52.1, 36.4 (d, J = 21.7 Hz), 13.6; IR (NaCl): 2972, 2931, 2867, 1815, 1757, 1238, 1004, 767, 697 cm^{-1}; HRMS (ESI): Calcd. for $C_{15}H_{18}BrF_2NNaO_3S$ [M+Na]$^+$: 432.0057; Found: 432.0049.

3.8 Methyl 2-((diethylamino)thio)-2-fluoro-3-(2-(fluorocarbonyl)-4,5-dimethoxyphenyl)propanoate (2h);

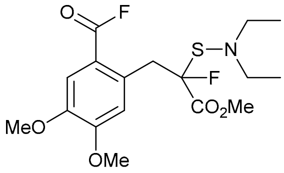

Following the general procedure, using methyl 5,6-dimethoxy-1-oxo-2,3-dihydro-1H-indene-2-carboxylate **1h** (25.0 mg, 0.1 mmol, 1.0 equiv) and DAST (30 μL, 0.2 mmol, 2.0 equiv) in THF (1 mL), the reaction mixture was stirred at room temperature for overnight. The crude product was purified by flash column chromatography (Hex: AcOEt = 10:1—3:1) to provide the title compound **2h** as a white solid in 54% yield (21.2 mg); mp: 102—103 °C; ^{1}H NMR ($CDCl_3$, 500 MHz) δ: 7.42 (s, 1H), 6.87 (s, 1H), 3.93 (s, 3H), 3.86—3.92 (m, 4H), 3.74—3.82 (m, 4H), 2.96—3.06 (m, 4H), 1.09 (t, 6H, J = 7.2 Hz); ^{19}F NMR ($CDCl_3$, 282 MHz) δ: +28.6 (s, 1F), –140.0 (dd, 1F, J = 24.5 Hz, J = 12.6 Hz); ^{13}C NMR ($CDCl_3$, 125.7 MHz) δ: 168.8 (d, J = 31.4 Hz), 156.4 (d, J = 342.1 Hz), 153.5, 147.8, 132.8 (d, J = 6.2 Hz), 116.7 (d, J = 57.6 Hz), 114.8 (d, J = 1.8 Hz), 114.3 (d, J = 1.4 Hz), 107.3 (d, J = 234.4 Hz), 56.1, 56.0, 52.8, 52.1, 36.5 (d, J = 21.7 Hz), 13.6; IR (NaCl): 2961, 2918, 2853, 1802, 1523, 1275, 1130, 1048, 800, 717, 592 cm^{-1};

HRMS (ESI): Calcd. for $C_{17}H_{23}F_2NNaO_5S$ $[M+Na]^+$: 414.1163; Found: 414.1189.

3.9 Methyl 2-((diethylamino)thio)-2-fluoro-4-(2-(fluorocarbonyl)phenyl)butanoate (2i);

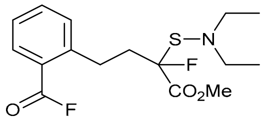

Following the general procedure, using methyl 1-oxo-1,2,3,4-tetrahydronaphthalene-2-carboxylate **1i** (40.8 mg, 0.2 mmol, 1.0 equiv) and DAST (60 μL, 0.4 mmol, 2.0 equiv) in THF (2 mL), the reaction mixture was stirred at room temperature for overnight. The crude product was purified by flash column chromatography (Hex: AcOEt = 20:1—12:1) to provide the title compound **2i** as a yellow oil in 30% yield (20.7 mg). ^{1}H NMR ($CDCl_3$, 500 MHz) δ: 7.99 (dd, 1H, J = 7.0 Hz, J = 1.0 Hz), 7.56—7.59 (m, 1H), 7.34—7.38 (m, 2H), 3.73 (s, 3H), 3.18—3.24 (m, 1H), 2.94—3.10 (m, 5H), 2.36—2.47 (m, 1H), 2.13—2.20 (m, 1H), 1.09 (s, 6H); ^{19}F NMR ($CDCl_3$, 282 MHz) δ: +28.7 (s, 1F), –142.3 (dd, 1F, J = 19.4 Hz, J = 6.4 Hz); ^{13}C NMR ($CDCl_3$, 125.7 MHz) δ: 169.0 (d, J = 32.5 Hz), 156.3 (d, J = 345.8 Hz), 145.6 (d, J = 7.5 Hz), 135.0, 132.9 (d, J = 1.2 Hz), 132.3 (d, J = 4.2 Hz), 127.2, 123.4 (d, J = 56.5 Hz), 107.7 (d, J = 230.2 Hz), 52.7, 52.2, 35.0 (d, J = 21.9 Hz), 28.9 (d, J = 3.2 Hz), 13.7; IR (NaCl): 2973, 2935, 2868, 1809, 1754, 1233, 1001, 928, 742, 695 cm^{-1}; HRMS (ESI): Calcd. for $C_{16}H_{21}F_2NNaO_3S$ $[M+Na]^+$: 368.1108; Found: 368.1105.

3.10 Methyl 2-((diethylamino)thio)-2-fluoro-5-(2-(fluorocarbonyl)phenyl)pentanoate (2j);

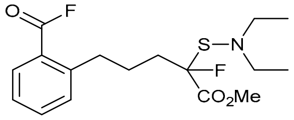

Following the general procedure, using methyl 5-oxo-6,7,8,9-tetrahydro-5H-benzo[7]annulene-6-carboxylate **1j** (43.6 mg, 0.2 mmol, 1.0 equiv) and DAST (60 μL, 0.4 mmol, 2.0 equiv) in THF (2 mL), the reaction mixture was stirred at room temperature for overnight. The crude product was

purified by flash column chromatography (Hex: AcOEt = 15:1—12:1) to provide the title compound **2j** as a yellow oil in 39% yield (28.0 mg). ^{1}H NMR ($CDCl_3$, 500 MHz) δ: 7.99 (dd, 1H, *J* = 7.5 Hz, *J* = 1.0 Hz), 7.56—7.59 (m, 1H), 7.31—7.36 (m, 2 H), 3.80 (s, 3H), 2.93—3.05 (m, 6H), 2.08—2.19 (m, 1H), 1.95—2.03 (m, 1H), 1.79—1.84 (m, 1H), 1.62—1.68 (m, 1H), 1.08 (t, 6H, *J* = 7.0 Hz); ^{19}F NMR ($CDCl_3$, 282 MHz) δ: +29.3 (s, 1F), –141.9 (dd, 1F, *J* = 25.6 Hz, *J* = 11.8 Hz); ^{13}C NMR ($CDCl_3$, 125.7 MHz) δ: 169.4 (d, *J* = 31.9 Hz), 156.4 (d, *J* = 345.6 Hz), 147.0 (d, *J* = 7.4 Hz), 134.9, 132.9 (d, *J* = 1.5 Hz), 131.6 (d, *J* = 4.2 Hz), 126.8, 123.2 (d, *J* = 56.3 Hz), 108.2 (d, *J* = 230.0 Hz), 52.8, 52.2, 34.0 (d, *J* = 22.3 Hz), 33.9, 25.0 (d, *J* = 2.2 Hz), 13.7; IR (NaCl): 2971, 2930, 2870, 1808, 1749, 1229, 999, 743, 697 cm^{-1}; HRMS (ESI): Calcd. for $C_{17}H_{23}F_2NNaO_3S$ $[M+Na]^+$: 382.1264; Found: 382.1260.

3.11 Benzyl 2-((diethylamino)thio)-2,6-difluoro-6-oxohexanoate (2k);

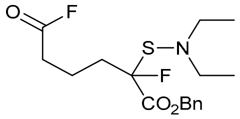

Following the general procedure, using benzyl 2-oxocyclopentanecarboxylate **2k** (43.6 mg, 0.2 mmol, 1.0 equiv) and DAST (60 μL, 0.4 mmol, 2.0 equiv) in THF (2 mL), the reaction mixture was stirred at room temperature for overnight. The crude product was purified by flash column chromatography (Hex: AcOEt = 12:1—5:1) to provide the title compound **2k** as a yellow oil in 39% yield (28.1 mg). ^{1}H NMR ($CDCl_3$, 400 MHz) δ: 7.32—7.42 (m, 5H), 5.29, 5.18 (AB quartet, *J*=12.0 Hz, 2H), 3.90—3.00 (m, 4H), 2.47—2.51 (m, 2H), 2.10—2.44 (m, 1H), 1.92—2.08 (m, 1H), 1.78—1.89 (m, 1H), 1.63—1.73 (m, 1H), 1.04—1.10 (m, 6H); ^{19}F NMR ($CDCl_3$, 282 MHz) δ: +44.9 (s, 1F),–142.0 (dd, 1F, *J* = 24.2 Hz, *J* = 9.5 Hz); ^{13}C NMR ($CDCl_3$, 125.7 MHz) δ: 168.5 (d, *J* = 31.8 Hz), 162.8 (d, *J* = 360.1 Hz), 134.9, 128.8, 128.7, 107.8 (d, *J* = 230.7 Hz), 67.9, 52.2, 32.9 (d, *J* = 22.3 Hz), 31.6 (d, *J* = 51.5 Hz), 18.3, 13.6; IR (NaCl): 2974, 2938, 2866, 1847, 1748, 1119, 1264, 1001, 753, 698, 669 cm^{-1}; HRMS (ESI): Calcd. for $C_{17}H_{24}F_2NO_3S$ $[M+H]^+$: 360.1445; Found: 360.1444.

3.12 Ethyl

2-((bis(2-methoxyethyl)amino)thio)-2-fluoro-3-(2-(fluorocarbonyl)phenyl)propanoate (3a);

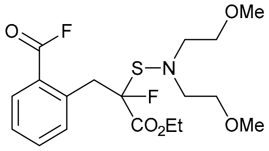

Following the general procedure, using ethyl 1-oxo-2,3-dihydro-1*H*-indene-2-carboxylate **1a** (20.4 mg, 0.1 mmol, 1.0 equiv) and Methoxy-DAST [Bis(2-methoxyethyl)aminosulfur Trifluoride] (41 μL, 0.2 mmol, 2.0 equiv) in THF (1 mL), the reaction mixture was stirred at room temperature for overnight. The crude product was purified by flash column chromatography (Hex: AcOEt = 12:1) to provide the title compound **3a** as a yellow oil in 77% yield (31.4 mg). ^{1}H NMR ($CDCl_3$, 500 MHz) δ: 7.97 (dd, 1H, J = 8.0 Hz, J = 1.5 Hz), 7.55—7.58 (m, 1H), 7.39—7.42 (m, 2 H), 4.17—4.22 (m, 2H), 3.91 (dd, 1H, J = 14.5 Hz, J = 10.5 Hz), 3.80 (dd, 1H, J = 25.5 Hz, J = 14.5 Hz), 3.51 (s, 4H), 3.33 (s, 6H), 3.22 (s, 4H), 1.26 (t, 3H, J = 7.0 Hz); ^{19}F NMR ($CDCl_3$, 282 MHz) δ: +31.2 (s, 1F), –140.7 (dd, 1F, J = 24.5 Hz, J = 10.7 Hz); ^{13}C NMR ($CDCl_3$, 125.7 MHz) δ: 168.1 (d, J = 31.4 Hz), 156.8 (d, J = 346.6 Hz), 137.7 (d, J = 6.2 Hz), 134.3, 132.7 (d, J = 2.5 Hz), 132.4, 127.9, 125.6 (d, J = 57.3 Hz), 107.2 (d, J = 234.3 Hz), 71.3, 71.0, 62.4, 58.8, 58.2, 36.5 (d, J = 21.4 Hz), 14.1; IR (NaCl): 2981, 2927, 1812, 1751, 1449, 1237, 1004, 741, 703 cm^{-1}; HRMS (ESI): Calcd. for $C_{18}H_{25}F_2NNaO_5S$ $[M+Na]^+$: 428.1319; Found: 428.1324.

3.13 Ethyl 2-((dimethylamino)thio)-2-fluoro-3-(2-(fluorocarbonyl)phenyl)propanoate (3b);

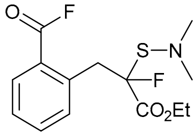

Following the general procedure, using ethyl 1-oxo-2,3-dihydro-1*H*-indene-2-carboxylate **1a** (20.4 mg, 0.1 mmol, 1.0 equiv) and Methyl-DAST (Dimethylaminosulfur trifluoride) (22 μL, 0.2 mmol, 2.0 equiv) in

THF (1 mL), the reaction mixture was stirred at room temperature for overnight. The crude product was purified by flash column chromatography (Hex: AcOEt = 15:1) to provide the title compound **3b** as a yellow oil in 51% yield (16.2 mg). ^{1}H NMR ($CDCl_3$, 500 MHz) δ: 7.97 (dd, 1H, *J* = 8.0 Hz, *J* = 1.5 Hz), 7.55—7.58 (m, 1H), 7.39—7.42 (m, 2H), 4.19—4.23 (m, 2H), 3.92 (dd, 1H, *J* = 14.5 Hz, *J* = 11.5 Hz), 3.80 (dd, 1H, *J* = 25.0 Hz, *J* = 14.5 Hz), 2.86 (s, 6H), 1.27 (t, 3H, *J* = 7.2 Hz); ^{19}F NMR ($CDCl_3$, 282 MHz) δ: +31.2 (s, 1F), –140.4 (dd, 1F, *J* = 24.5 Hz, *J* = 11.5 Hz); ^{13}C NMR ($CDCl_3$, 125.7 MHz) δ: 168.6 (d, *J* = 31.1 Hz), 156.8 (d, *J* = 346.6 Hz), 137.6 (d, *J* = 6.4 Hz), 134.3, 132.8 (d, *J* = 3.2 Hz), 132.4, 128.0, 125.6 (d, *J* = 57.3 Hz), 107.1 (d, *J* = 234.0 Hz), 62.4, 49.3, 36.8 (d, *J* = 20.7 Hz), 14.1; IR (NaCl): 2986, 2937, 2791, 1811, 1749, 1237, 1003, 755, 742, 702 cm^{-1}; HRMS (ESI): Calcd. for $C_{14}H_{17}F_2NNaO_3S$ $[M+Na]^+$: 340.0795; Found: 340.0789.

3.14 Ethyl 2-fluoro-3-(2-(fluorocarbonyl)phenyl)-2-(morpholinothio)propanoate (3c);

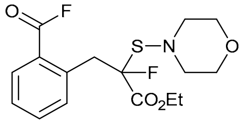

Following the general procedure, using ethyl 1-oxo-2,3-dihydro-1*H*-indene-2-carboxylate **1a** (20.4 mg, 0.1 mmol, 1.0 equiv) and Morph-DAST (Morpholinosulfur Trifluoride) (27 μL, 0.2 mmol, 2.0 equiv) in THF (1 mL), the reaction mixture was stirred at room temperature for overnight. The crude product was purified by flash column chromatography (Hex: AcOEt = 10:1) to provide the title compound **3c** as a yellow oil in 72% yield (25.9 mg). ^{1}H NMR ($CDCl_3$, 500 MHz) δ: 7.99 (dd, 1H, *J* = 8.0 Hz, *J* = 1.5 Hz), 7.56—7.59 (m, 1H), 7.39—7.44 (m, 2H), 4.19—4.24 (m, 2H), 4.00 (dd, 1H, *J* = 14.5 Hz, *J* = 12.0 Hz), 3.79 (dd, 1H, *J* = 24.5 Hz, *J* = 14.5 Hz), 3.58—3.65 (m, 4H), 3.05—3.12 (m, 4H), 1.27 (t, 3H, *J* = 7.2 Hz); ^{19}F NMR ($CDCl_3$, 282 MHz) δ: +31.2 (s, 1F), –138.6 (dd, 1F, *J* = 23.6 Hz, *J* = 11.8 Hz); ^{13}C NMR ($CDCl_3$, 125.7 MHz) δ: 168.0 (d, *J* = 30.9 Hz), 156.8 (d, *J* = 346.5 Hz), 137.4 (d, *J* = 6.2 Hz), 134.3, 132.8 (d, *J* = 3.2 Hz), 132.5,

128.1, 125.5 (d, J = 57.4 Hz), 107.6 (d, J = 235.3 Hz), 67.8, 62.5, 57.3, 37.0 (d, J = 21.6 Hz), 14.2; IR (NaCl): 2961, 2913, 2853, 1811, 1750, 1237, 1113, 1003, 742, 702 cm^{-1}; HRMS (ESI): Calcd. for $C_{16}H_{19}F_2NNaO_4S$ $[M+Na]^+$: 382.0901; Found: 382.0894.

Methyl 2-((diethylamino)thio)-2-fluoro-3-phenylpropanoate (2o);

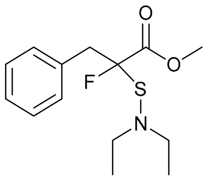

Following the general procedure, using methyl 2-benzyl-3-oxobutanoate **1o** (41.2 mg, 0.2 mmol, 1.0 equiv) and DAST (60 μL, 0.4 mmol, 2.0 equiv) in DMF (2 mL), the reaction mixture was stirred at 50 °C for overnight. After cooling to room temperature, the reaction was quenched by addition of water (10 mL), extracted with ethyl acetate (3 x 20 mL), dried over with Na_2SO_4 and then concentrated in vacuo. The crude product was purified by flash column chromatography (Hex: AcOEt = 20:1—10:1) to provide the title compound **2o** as a yellow oil in 45% yield (25.6 mg). ^1H NMR ($CDCl_3$, 500 MHz) δ: 7.21—7.29 (m, 5H), 3.73 (s, 3H), 3.33 (dd, 1H, J = 28.0 Hz, J = 14.5 Hz), 3.21 (t, 1H, J = 14.0 Hz), 2.95—3.02 (m, 4H), 1.10 (t, 6H, J = 7.2 Hz); ^{19}F NMR ($CDCl_3$, 282 MHz) δ: –140.2 (dd, 1F, J = 26.7 Hz, J = 12.9 Hz); ^{13}C NMR ($CDCl_3$, 125.7 MHz) δ: 168.9 (d, J = 31.6 Hz), 133.6, 130.2, 128.4, 127.4, 107.9 (d, J = 233.1 Hz), 52.7, 52.3, 40.3 (d, J = 21.7 Hz), 13.7; IR (NaCl): 2970, 2920, 2861, 1584, 1237, 1167, 1082, 913, 785, 708, 609 cm^{-1}; HRMS (ESI): Calcd. for $C_{14}H_{20}FNNaO_2S$ $[M+Na]^+$: 308.1096; Found: 308.1079.

4. X-ray crystallography data for the 2h (CCDC 1415530)

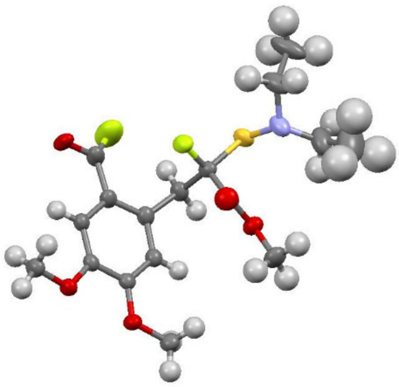

EXPERIMENTAL DETAILS

A. Crystal Data

Empirical Formula	$C_{17}H_{23}F_{2}NO_{5}S$
Formula Weight	391.43
Crystal Color, Habit	colorless, needle
Crystal Dimensions	0.200 X 0.100 X 0.100 mm
Crystal System	monoclinic
Lattice Type	C-centered
Lattice Parameters	a = 38.0429(9) Å<br>b = 5.9645(2) Å<br>c = 19.5829(5) Å<br>β = 120.9238(7) ^{o}<br>V = 3811.8(2) Å^{3}
Space Group	C2/c (#15)
Z value	8
D_{calc}	1.364 g/cm^{3}
F_{000}	1648.00
μ(MoKα)	2.153 cm^{-1}

5. Optimization of ring-opening reaction condition (SCF_3)

Table S2. Optimization of ring-opening reaction condition[a]

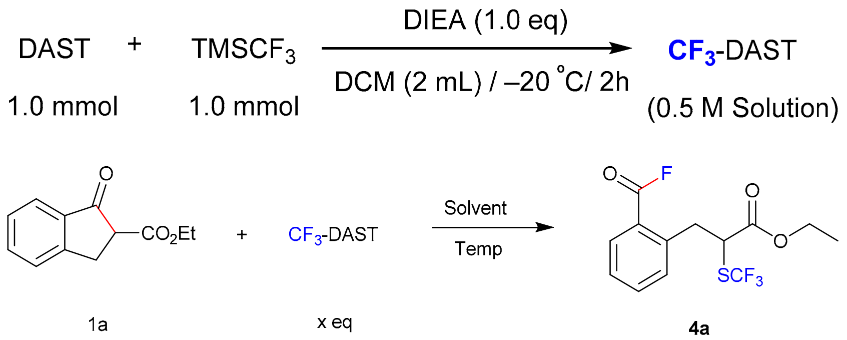

Entry	Solvent	CF_3-DAST (eq)	Temp(°C)	Time	**4a** (%)[b]
1	THF	2.0	RT	Overnight	28
2	THF	2.0	50	Overnight	18
3	THF	2.0	0	Overnight	17
4	THF	2.0	–20	Overnight	22
5	DMF	2.0	RT	Overnight	24
6	DCM	2.0	RT	Overnight	14
7	CH_3CN	2.0	RT	Overnight	3
8	MeOH	2.0	RT	Overnight	0
9	Toluene	2.0	RT	Overnight	18
10	Et_2O	2.0	RT	Overnight	34
11	dioxane	2.0	RT	Overnight	49
12	Benzene	2.0	RT	Overnight	17
13	$CHCl_3$	2.0	RT	Overnight	30
14	Pridine	2.0	RT	Overnight	32
15	dioxane	2.0	50	Overnight	38

16	dioxane	2.0	0	Overnight	67
**17**	**dioxane**	**2.0**	**–10**	**Overnight**	**78**
18	dioxane	1.0	–10	Overnight	52
19	dioxane	1.5	–10	Overnight	61

[a]The reaction of **1a** with 2.0 equivalents of CF_3-DAST (0.5 M mixture in DCM) was carried out in different solvents and at different temperature. For detailed reaction conditions, see the table S2. [b]Yield was determined by ^{19}F NMR spectroscopy with an internal standard as $C_6H_5CF_3$.

Table S3. Optimization of ring-opening reaction condition[a]

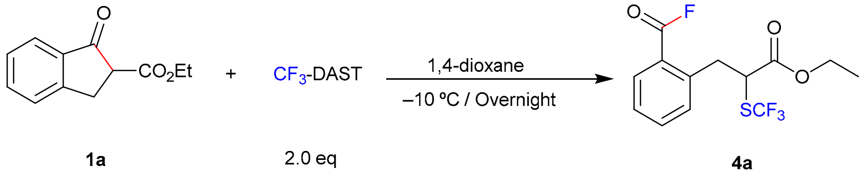

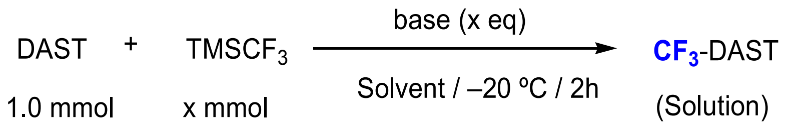

Entry	Solvent	Base (eq)	$TMSCF_3$	Concentration	**4a** (%)[b]	
					Time	yield
1	THF	DIEA (1.0)	1.0	0.5	Overnight	46
2	Et_2O	DIEA (1.0)	1.0	0.5	Overnight	40
3	$CHCl_3$	DIEA (1.0)	1.0	0.5	Overnight	0
4	dioxane	DIEA (1.0)	1.0	0.5	Overnight	5
5	DCM	TEA (1.0)	1.0	0.5	Overnight	54
6	DCM	DBU (1.0)	1.0	0.5	Overnight	trace
7	DCM	DIEA (1.0)	2.0	0.5	Overnight	61
8	DCM	DIEA (1.0)	3.0	0.5	Overnight	38
9	DCM	DIEA (1.0)	1.0	1.0	Overnight	66
10	DCM	DIEA (0.5)	1.0	0.5	Overnight	53
11	DCM	DIEA (1.5)	1.0	0.5	Overnight	72
12	DCM	DIEA (1.0)	1.0	0.5	30 min	42
13	DCM	DIEA (1.0)	1.0	0.5	1 h	47

14	DCM	DIEA (1.0)	1.0	0.5	3 h	58
15	DCM	---------	1.0	0.5	Overnight	6
16	DCM	DIEA (0.1)	1.0	0.5	Overnight	19

[a]The reaction of**1a** with 2.0 equivalents of CF_3-DAST (0.5 M mixture in DCM) was carried out in different solvents and different bases. For detailed reaction conditions, see the table S3. [b] Yield was determined by ^{19}F NMR spectroscopy with an internal standard as $C_6H_5CF_3$.

6. Typical procedure preparation of CF_3-DAST reagents

A flame-dried vessel was successively charged, under nitrogen, with diisopropylethylamine (0.17 mL, 1.0 mmol) and anhydrous dichloromethane (2 mL). The resulting mixture was cooled to –20 °C, the diethylaminosulfurtrifluoride (0.15 mL, 1.0 mmol) was added slowly by syringe, stirred for 15 min at same temperature, then the trimethylsilyltrifluoromethane (0.16 mL, 1.0 mmol) was added slowly by syringe, and stirring for two hours under the same reaction temperature. After two hours, the solution was directly used for next step without purification. See below.

7. General procedure and product characterization data for 4a—4o;

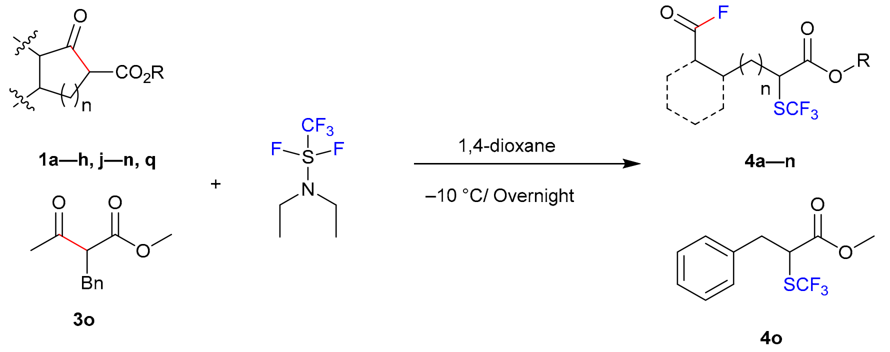

A flame-dried vessel was successively charged, under nitrogen, with *β*-keto esters **1a—h, j—n, q and 1o** (0.1 mmol, 1.0 equiv) and anhydrous 1,4-dioxane (1.0 mL). The solution was cooled to –10 °C and the 0.5M solution of CF_3-DAST (0.2 mmol, 2.0 equiv, 0.45 mL taken from the solution above mentioned) or (0.4 mmol, 4.0 equiv,

0.9 mL taken from the solution above mentioned) in CH_2Cl_2 was added slowly by syringe. Then the reaction mixture was stirred at –10 °C or room temperature for overnight, quenched by addition of water (10 mL), extracted with ethyl acetate (3 x 20 mL), dried over with Na_2SO_4 and then concentrated in vacuo. The crude product was purified by flash column chromatography to provide the title compound **4a—4n** and **4o**.

7.1 Ethyl 3-(2-(fluorocarbonyl)phenyl)-2-((trifluoromethyl)thio)propanoate (4a);

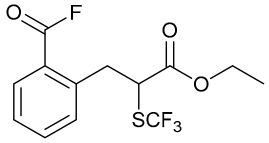

Following the general procedure, using ethyl 1-oxo-2,3-dihydro-1*H*-indene-2-carboxylate **1a** (20.4 mg, 0.1 mmol, 1.0 equiv) and CF_3-DAST (0.45 mL, 2.0 equiv) in dioxane (1 mL), the reaction mixture was stirred at –10 °C for overnight. The crude product was purified by flash column chromatography (Hex: AcOEt: DCM = 8:1:1) to provide the title compound **4a** as a yellow oil in 61% yield (19.7 mg). ^{1}H NMR ($CDCl_3$, 500 MHz) δ: 8.00 (dd, 1H, J = 7.5 Hz, J = 1.0 Hz), 7.54—7.57 (m, 1H), 7.37—7.40 (m, 1H), 7.30 (d, 1H, J = 7.5 Hz), 4.05—4.10 (m, 2H), 3.98—4.04 (m, 1H), 3.53 (dd, 1H, J = 13.5 Hz, J = 7.0 Hz), 3.47 (dd, 1H, J = 13.5 Hz, J = 9.0 Hz), 1.09 (t, 3 H, J = 7.2 Hz; ^{19}F NMR ($CDCl_3$, 282 MHz) δ: +27.9 (s, 1F), –40.7 (s, 3F); ^{13}C NMR ($CDCl_3$, 125.7 MHz) δ: 170.3, 156.4 (d, J = 345.5 Hz), 141.2 (d, J = 8.2 Hz), 135.1, 133.3 (d, J = 4.2 Hz), 133.2, 130.0 (q, J = 307.4 Hz), 128.4, 123.7 (d, J = 56.9 Hz), 62.1, 46.3, 37.0, 13.9; IR (NaCl): 2986, 2942, 1806, 1740, 1238, 1112, 1009, 757, 742, 696 cm^{-1}; HRMS (ESI): Calcd. for $C_{13}H_{12}F_4NaO_3S$ [M+Na]$^+$: 347.0341; Found: 347.0326.

7.2 Methyl 3-(2-(fluorocarbonyl)phenyl)-2-((trifluoromethyl)thio)propanoate (4b);

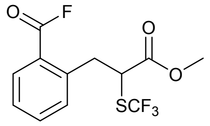

Following the general procedure, using methyl 1-oxo-2,3-dihydro-1H-indene-2-carboxylate **1b** (19.1 mg, 0.1 mmol, 1.0 equiv) and CF_3-DAST (0.45 mL, 2.0 equiv) in dioxane (1 mL), the reaction mixture was stirred at –10 °C for overnight. The crude product was purified by flash column chromatography (Hex: AcOEt: DCM = 8:1:1) to provide the title compound **4b** as a yellow oil in 35% yield (10.9 mg). ^{1}H NMR ($CDCl_3$, 500 MHz) δ: 8.00 (dd, 1H, *J* = 7.5 Hz, *J* = 1.0 Hz), 7.55—7.58 (m, 1H), 7.38—7.41 (m, 1H), 7.29 (d, 1H, *J* = 8.0 Hz), 4.08—4.11 (m, 1H), 3.61 (s, 3H), 3.48—3.54 (m, 2H); ^{19}F NMR ($CDCl_3$, 282 MHz) δ: +27.9 (s, 1F), –40.9 (s, 3F); ^{13}C NMR ($CDCl_3$, 125.7 MHz) δ: 170.8, 156.5 (d, *J* = 345.6 Hz), 141.1 (d, *J* = 8.2 Hz), 135.1, 133.2 (d, *J* = 4.0 Hz), 133.2, 129.9 (q, *J* = 307.7 Hz), 128.5, 123.7 (d, *J* = 56.9 Hz), 53.0, 46.1, 37.0; IR (NaCl): 2957, 1806, 1745, 1237, 1110, 1008, 757, 742, 696 cm^{-1}; HRMS (ESI): Calcd. for $C_{12}H_{10}F_4NaO_3S$ $[M+Na]^+$: 333.0184; Found: 333.0193.

7.3 Benzyl 3-(2-(fluorocarbonyl)phenyl)-2-((trifluoromethyl)thio)propanoate (4c);

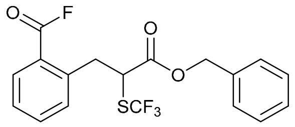

Following the general procedure, using benzyl 1-oxo-2,3-dihydro-1H-indene-2-carboxylate **1c** (26.7 mg, 0.1 mmol, 1.0 equiv) and CF_3-DAST (0.45 mL, 2.0 equiv) in dioxane (1 mL), the reaction mixture was stirred at –10 °C for overnight. The crude product was purified by flash column chromatography (Hex: AcOEt: DCM = 10:1:1) to provide the title compound **4c** as a yellow oil in 64% yield (24.7 mg). ^{1}H NMR ($CDCl_3$, 500 MHz) δ: 7.96 (dd, 1H, *J* = 8.0 Hz, *J* = 1.5 Hz), 7.43—7.46 (m, 1H), 7.32—7.36 (m, 1H), 7.24—7.26 (m, 3H), 7.18—7.20 (m, 1H), 7.13—7.15 (m, 2H), 4.98—5.04 (m, 2H), 4.12—4.15 (m, 1H),

3.55 (dd, 1H, *J* = 13.5 Hz, *J* = 7.0 Hz), 3.45 (dd, 1H, *J* = 13.5 Hz, *J* = 9.0 Hz); ^{19}F NMR ($CDCl_3$, 282 MHz) δ: +27.8 (s, 1F), –40.7 (s, 3F); ^{13}C NMR ($CDCl_3$, 125.7 MHz) δ: 170.2, 156.4 (d, *J* = 345.5 Hz), 141.0 (d, *J* = 8.2 Hz), 135.1, 134.9, 133.2 (d, *J* = 3.6 Hz), 129.9 (q, *J* = 307.8 Hz), 128.7, 128.6, 128.5, 128.4, 123.7 (d, *J* = 56.8 Hz), 67.8, 46.2, 37.1; IR (NaCl): 3068, 3035, 2959, 1804, 1741, 1238, 1161, 1110, 1007, 741, 696 cm^{-1}; HRMS (ESI): Calcd. for $C_{18}H_{14}F_4NaO_3S$ $[M+Na]^+$: 409.0497; Found: 409.0486.

7.4 Methyl 3-(2-(fluorocarbonyl)-4-methylphenyl)-2-((trifluoromethyl)thio)propanoate (4d);

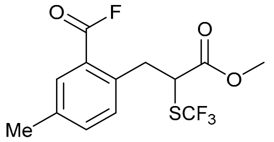

Following the general procedure, using methyl 6-methyl-1-oxo-2,3-dihydro-1H-indene-2-carboxylate **1d** (20.4 mg, 0.1 mmol, 1.0 equiv) and CF_3-DAST (0.45 mL, 2.0 equiv) in dioxane (1 mL), the reaction mixture was stirred at –10 °C for overnight. The crude product was purified by flash column chromatography (Hex: AcOEt: DCM = 10:1:1) to provide the title compound **4d** as a yellow oil in 59% yield (19.1 mg). ^{1}H NMR ($CDCl_3$, 500 MHz) δ: 7.79 (d, 1H, *J* = 1.5 Hz), 7.35 (dd, 1H, *J* = 7.5 Hz, *J* = 1.0 Hz), 7.15—7.17 (m, 1H), 4.06—4.09 (m, 1H), 3.61 (s, 3H), 3.41—3.49 (m, 2H), 2.33 (s, 3H); ^{19}F NMR ($CDCl_3$, 282 MHz) δ: +27.6 (s, 1F), —40.8 (s, 3F); ^{13}C NMR ($CDCl_3$, 125.7 MHz) δ: 170.9, 156.6 (d, *J* = 345.5 Hz), 138.5, 138.0 (d, *J* = 7.9 Hz), 135.9, 133.7, 133.1 (d, *J* = 4.3 Hz), 129.9 (q, *J* = 307.3 Hz), 123.4 (d, *J* = 55.9 Hz), 53.0, 46.2, 36.5, 21.0; IR (NaCl): 2957, 1803, 1746, 1501, 1439, 1261, 1165, 1111, 1019, 758, 730, 697 cm^{-1}; HRMS (ESI): Calcd. for $C_{13}H_{12}F_4NaO_3S$ $[M+Na]^+$: 347.0341; Found: 347.0336.

7.5 Methyl 3-(2-(fluorocarbonyl)-4-methoxyphenyl)-2-((trifluoromethyl)thio)propanoate (4e);

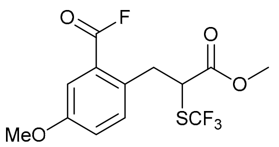

Following the general procedure, using methyl 6-methoxy-1-oxo-2,3-dihydro-1H-indene-2-carboxylate **1e** (22.0 mg, 0.1 mmol, 1.0 equiv) and CF_3-DAST (0.45 mL, 2.0 equiv) in dioxane (1 mL), the reaction mixture was stirred at –10 °C for overnight. The crude product was purified by flash column chromatography (Hex: AcOEt: DCM = 10:1:1) to provide the title compound **4e** as a yellow oil in 48% yield (16.3 mg). ^{1}H NMR ($CDCl_3$, 500 MHz) δ: 7.47 (d, 1H, J = 2.5 Hz), 7.18—7.20 (m, 1H), 7.08 (dd, 1H, J = 8.5 Hz, J = 3.0 Hz), 4.04—4.07 (m, 1H), 3.78 (s, 3H), 3.61 (s, 3H), 3.37—3.44 (m, 2H); ^{19}F NMR ($CDCl_3$, 282 MHz) δ: +27.8 (s, 1F), –40.8 (s, 3F); ^{13}C NMR ($CDCl_3$, 125.7 MHz) δ: 170.9, 159.1, 156.3 (d, J = 346.0 Hz), 134.4 (d, J = 4.5 Hz), 132.9 (d, J = 7.5 Hz), 130.0 (q, J = 307.2 Hz), 124.5 (d, J = 56.4 Hz), 121.1, 117.7, 55.7, 53.0, 46.3, 36.2; IR (NaCl): 3009, 2956, 2844, 1807, 1744, 1504, 1165, 1111, 1010, 758, 731, 700 cm^{-1}; HRMS (ESI): Calcd. for $C_{13}H_{12}F_4NaO_4S$ $[M+Na]^+$: 363.0290; Found: 363.0296.

7.6 Ethyl 3-(5-chloro-2-(fluorocarbonyl)phenyl)-2-((trifluoromethyl)thio)propanoate (4f);

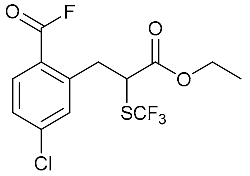

Following the general procedure, using ethyl 5-chloro-1-oxo-2,3-dihydro-1H-indene-2-carboxylate **1f** (23.9 mg, 0.1 mmol, 1.0 equiv) and CF_3-DAST (0.45 mL, 2.0 equiv) in dioxane (1 mL), the reaction mixture was stirred at –10 °C for overnight. The crude product was purified by flash column chromatography (Hex: AcOEt: DCM = 10:1:1) to provide the title compound **4f** as a yellow oil in 45% yield (16.1 mg). ^{1}H NMR ($CDCl_3$, 500 MHz) δ: 7.93 (d, 1H, J = 8.5 Hz), 7.37 (dd, 1H, J = 8.5 Hz, J = 2.0 Hz), 7.30 (s, 1H), 4.02—4.12 (m, 3H), 3.51 (dd, 1H, J = 13.5 Hz, J = 7.0 Hz), 3.44 (dd, 1H, J = 13.5 Hz, J = 9.0 Hz), 1.13 (t, 3H,

J = 7.2 Hz); ^{19}F NMR ($CDCl_3$, 282 MHz) δ: +28.2 (s, 1F), –40.7 (s, 3F); ^{13}C NMR ($CDCl_3$, 125.7 MHz) δ: 170.0, 155.7 (d, J = 344.7 Hz), 143.1 (d, J = 8.2 Hz), 141.8, 134.4, 133.4 (d, J = 4.1 Hz), 129.9 (q, J = 307.5 Hz), 128.7, 122.1 (d, J = 58.1 Hz), 62.4, 46.0, 36.8, 13.9; IR (NaCl): 2984, 2943, 1809, 1740, 1595, 1566, 1236, 1111, 1010, 768, 758, 690 cm^{-1}; HRMS (ESI): Calcd. for $C_{13}H_{11}ClF_4NaO_3S$ $[M+Na]^+$: 380.9951; Found: 380.9955.

7.7 Methyl 3-(5-bromo-2-(fluorocarbonyl)phenyl)-2-((trifluoromethyl)thio)propanoate (4g);

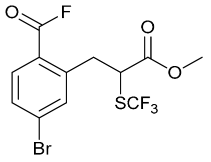

Following the general procedure, using methyl 5-bromo-1-oxo-2,3-dihydro-1H-indene-2-carboxylate **1g** (26.9 mg, 0.1 mmol, 1.0 equiv) and CF_3-DAST (0.45 mL, 2.0 equiv) in dioxane (1 mL), the reaction mixture was stirred at –10 °C for overnight. The crude product was purified by flash column chromatography (Hex: AcOEt: DCM = 10:1:1) to provide the title compound **4g** as a yellow oil in 49% yield (19.0 mg). ^{1}H NMR ($CDCl_3$, 500 MHz) δ: 7.84 (d, 1H, J = 8.5 Hz), 7.54 (dd, 1H, J = 8.5 Hz, J = 2.0 Hz), 7.47 (s, 1H), 4.04—4.07 (m, 1H), 3.64 (s, 3H), 3.46—3.49 (m, 2H); ^{19}F NMR ($CDCl_3$, 282 MHz) δ: +28.2 (s, 1F), –40.8 (s, 3F); ^{13}C NMR ($CDCl_3$, 125.7 MHz) δ: 170.4, 155.9 (d, J = 344.9 Hz), 142.9 (d, J = 8.1 Hz), 136.3 (d, J = 4.1 Hz), 134.3, 131.8, 130.6, 129.8 (q, J = 307.8 Hz), 122.5 (d, J = 58.0 Hz), 53.2, 45.9, 36.7; IR (NaCl): 3022, 2956, 1807, 1742, 1589, 1561, 1239, 1111, 1011, 758, 689, 669 cm^{-1}; HRMS (ESI): Calcd. for $C_{12}H_9BrF_4NaO_3S$ $[M+Na]^+$: 410.9290; Found: 410.9261.

7.8 Methyl 3-(2-(fluorocarbonyl)-4,5-dimethoxyphenyl)-2-((trifluoromethyl)thio)propanoate (4h);

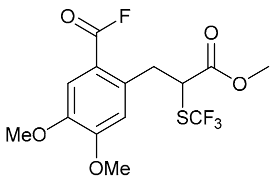

Following the general procedure, using methyl 5,6-dimethoxy-1-oxo-2,3-dihydro-1H-indene-2-carboxylate **1h** (25.0 mg, 0.1 mmol, 1.0 equiv) and CF_3-DAST (0.45 mL, 2.0 equiv) in dioxane (1 mL), the reaction mixture was stirred at –10 °C for overnight. The crude product was purified by flash column chromatography (Hex: AcOEt: DCM = 5:1:1) to provide the title compound **4h** as a white solid in 40% yield (14.7 mg). mp: 89.7—90.9. ^{1}H NMR ($CDCl_3$, 500 MHz) δ: 7.40 (s, 1H), 6.72 (s, 1H), 4.06—4.10 (m, 1H), 3.89 (s, 3H), 3.86 (s, 3H), 3.62 (s, 3H), 3.49 (dd, 1H, J = 13.5 Hz, J = 7.0 Hz), 3.41 (dd, 1H, J = 13.5 Hz, J = 8.5 Hz); ^{19}F NMR ($CDCl_3$, 282 MHz) δ: +25.5 (s, 1F), –40.7 (s, 3F); ^{13}C NMR ($CDCl_3$, 125.7 MHz) δ: 171.0, 156.2 (d, J = 341.2 Hz), 154.2, 148.2, 136.4 (d, J = 8.2 Hz), 129.9 (q, J = 307.6 Hz), 115.5 (d, J = 4.3 Hz), 115.0 (d, J = 57.4 Hz), 114.7, 56.4, 56.2, 53.0, 46.0, 36.9; IR (NaCl): 2944, 2850, 1794, 1741, 1576, 1526, 1277, 1214, 1127, 962, 878, 768, 720, 651 cm^{-1}; HRMS (ESI): Calcd. for $C_{14}H_{14}F_4NaO_5S$ $[M+Na]^+$: 393.0396; Found: 393.0386.

7.9 Benzyl 4-(2-(fluorocarbonyl)phenyl)-2-((trifluoromethyl)thio)butanoate (4i);

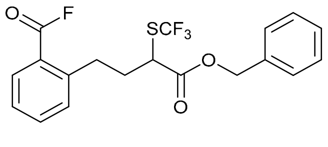

Following the general procedure, using benzyl 1-oxo-1,2,3,4-tetrahydronaphthalene-2-carboxylate **1q** (56.1 mg, 0.2 mmol, 1.0 equiv) and CF_3-DAST (1.8 mL, 4.0 equiv) in dioxane (2 mL), the reaction mixture was stirred at room temperature for overnight. The crude product was purified by flash column chromatography (Hex: AcOEt = 12:1) to provide the title compound **4i** as a yellow oil in 27% yield (21.6 mg). ^{1}H NMR ($CDCl_3$, 500 MHz) δ: 8.01 (dd, 1H, J = 8.0 Hz, J = 1.5 Hz), 7.55—7.59 (m, 1H), 7.34—7.39 (m, 6H), 7.26 (d, 1H, J = 6.0 Hz), 5.21 (s, 2H), 3.90—3.93 (m, 1H), 3.14—3.20 (m, 1H), 3.01—3.06 (m, 1H),

2.21—2.29 (m, 1H), 2.12—2.19 (m, 1H); ^{19}F NMR ($CDCl_3$, 282 MHz) δ: +28.6 (s, 1F), –40.8 (s, 3F); ^{13}C NMR ($CDCl_3$, 125.7 MHz) δ: 170.4, 156.3 (d, J = 345.2 Hz), 145.3 (d, J = 8.0 Hz), 135.2, 135.0, 133.1, 131.9 (d, J = 4.2 Hz), 130.1 (q, J = 307.5 Hz), 128.8, 128.7, 128.6, 127.4, 123.2 (d, J = 56.4 Hz), 68.0, 46.3, 33.1, 31.8; IR (NaCl): 3068, 3034, 2960, 1806, 1740, 1231, 1113, 1003, 756, 696, 646 cm^{-1}; HRMS (ESI): Calcd. for $C_{19}H_{16}F_4NaO_3S$ [M+Na]$^+$: 423.0654; Found: 423.0652.

7.10 Methyl 5-(2-(fluorocarbonyl)phenyl)-2-((trifluoromethyl)thio)pentanoate (4j);

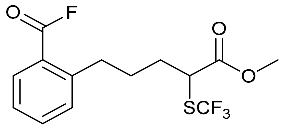

Following the general procedure, using methyl 5-oxo-6,7,8,9-tetrahydro-5H-benzo[7]annulene-6-carboxylate **1j** (43.7 mg, 0.2 mmol, 1.0 equiv) and CF_3-DAST (1.8 mL, 4.0 equiv) in dioxane (2 mL), the reaction mixture was stirred at room temperature for overnight. The crude product was purified by flash column chromatography (Hex: AcOEt = 12:1) to provide the title compound **4j** as a yellow oil in 53% yield (36.2 mg). ^{1}H NMR ($CDCl_3$, 500 MHz) δ: 7.94 (dd, 1H, J = 8.0 Hz, J = 1.5 Hz), 7.50—7.54 (m, 1H), 7.25—7.31 (m, 2H), 3.74—3.77 (m, 1H), 3.70 (s, 3H), 2.91—3.02 (m, 2H), 1.94—2.01 (m, 1H), 1.81—1.89 (m, 1H), 1.69—1.74 (m, 2H); ^{19}F NMR ($CDCl_3$, 282 MHz) δ: +29.1 (s, 1F), –41.1 (s, 3F); ^{13}C NMR ($CDCl_3$, 125.7 MHz) δ: 171.3, 156.4 (d, J = 345.2 Hz), 146.8 (d, J = 8.0 Hz), 135.0, 133.0 (d, J = 1.4 Hz), 131.7 (d, J = 4.2 Hz), 130.1 (q, J = 306.9 Hz), 127.0, 123.2 (d, J = 56.5 Hz), 53.1, 46.1 (d, J = 1.5 Hz), 33.9, 31.8, 28.3; IR (NaCl): 2956, 2870, 1807, 1744, 1449, 1231, 1115, 1000, 758, 742, 697 cm^{-1}; HRMS (ESI): Calcd. for $C_{14}H_{14}F_4NaO_3S$ [M+Na]$^+$: 361.0497; Found: 361.0481.

7.11 Benzyl 6-fluoro-6-oxo-2-((trifluoromethyl)thio)hexanoate (4k);

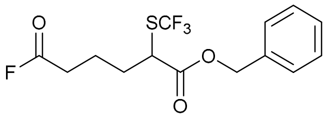

Following the general procedure, using methyl 5-oxo-6,7,8,9-tetrahydro-5H-benzo[7]annulene-6-carboxylate **1k** (43.7 mg, 0.2 mmol, 1.0 equiv) and CF_3-DAST (0.9 mL, 2.0 equiv) in dioxane (2 mL), the reaction mixture was stirred at room temperature for overnight. Target product **4k** is too unstable to be isolated under purification, so **4k** was ascertained only by ^{19}F NMR of the crude product and yield was also decided by ^{19}F NMR, in 26% yield. ^{19}F NMR ($CDCl_3$, 282 MHz) δ: +44.5 (s, 1F), –40.8 (s, 3F);

7.12 Isopropyl 3-(2-(fluorocarbonyl)phenyl)-2-((trifluoromethyl)thio)propanoate (4l);

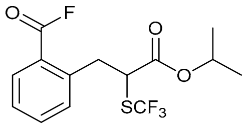

Following the general procedure, using isopropyl 1-oxo-2,3-dihydro-1H-indene-2-carboxylate **1l** (21.8 mg, 0.1 mmol, 1.0 equiv) and CF_3-DAST (0.45 mL, 2.0 equiv) in dioxane (1 mL), the reaction mixture was stirred at –10 °C for overnight. The crude product was purified by flash column chromatography (Hex: AcOEt: DCM = 8:1:1) to provide the title compound **4l** as a yellow oil in 56% yield (18.9 mg). ^{1}H NMR ($CDCl_3$, 500 MHz) δ: 8.00 (dd, 1H, *J* = 8.0 Hz, *J* = 1.0 Hz), 7.53—7.56 (m, 1H), 7.37—7.40 (m, 1H), 7.30 (d, 1H, *J* = 8.0 Hz), 4.83—4.91 (m, 1H), 4.03—4.06 (m, 1H), 3.54 (dd, 1H, *J* = 13.5 Hz, *J* = 7.0 Hz), 3.43 (dd, 1H, *J* = 13.5 Hz, *J* = 9.0 Hz), 1.13 (d, 3H, *J* = 6.5 Hz), 0.97 (d, 3H, *J* = 6.0 Hz); ^{19}F NMR ($CDCl_3$, 282 MHz) δ: +27.9 (s, 1F), —40.7 (s, 3F); ^{13}C NMR ($CDCl_3$, 125.7 MHz) δ: 169.9, 156.4 (d, *J* = 345.5 Hz), 141.2 (d, *J* = 8.0 Hz), 135.1, 133.3 (d, *J* = 4.0 Hz), 133.2, 130.0 (q, *J* = 307.5 Hz), 128.4, 123.7 (d, *J* = 56.5 Hz), 69.9, 46.4, 37.0, 21.5, 21.4; IR (NaCl): 2985, 2938, 1807, 1736, 1237, 1169, 1100, 1008, 757, 741, 696 cm^{-1}; HRMS (ESI): Calcd. for $C_{14}H_{14}F_4NaO_3S$ $[M+Na]^+$: 361.0497; Found: 361.0500.

7.13 Adamantan-1-yl

3-(5-bromo-2-(fluorocarbonyl)phenyl)-2-((trifluoromethyl)thio)propanoate (4m);

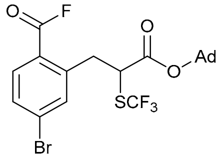

Following the general procedure, using adamantan-1-yl 5-bromo-1-oxo-2,3-dihydro-1H-indene-2-carboxylate **1m** (38.9 mg, 0.1 mmol, 1.0 equiv) and CF_3-DAST (0.45 mL, 2.0 equiv) in dioxane (1 mL), the reaction mixture was stirred at –10 °C for overnight. The crude product was purified by flash column chromatography (Hex: AcOEt: DCM = 8:1:1) to provide the title compound **4m** as a yellow oil in 37% yield (18.8 mg). ^{1}H NMR ($CDCl_3$, 500 MHz) δ: 7.84 (d, 1H, *J* = 8.5 Hz), 7.53 (dd, 1H, *J* = 8.5 Hz, *J* = 2.0 Hz), 7.48 (s, 1H), 3.92—3.95 (m, 1H), 3.54 (dd, 1H, *J* = 13.5 Hz, *J* = 6.5 Hz), 3.30 (dd, 1H, *J* = 13.5 Hz, *J* = 10.0 Hz), 2.08 (s, 3H), 1.89—1.94 (m, 6H), 1.56 (t, 6H, *J* = 2.7 Hz); ^{19}F NMR ($CDCl_3$, 282 MHz) δ: +28.2 (s, 1F), –40.5 (s, 3F); ^{13}C NMR ($CDCl_3$, 125.7 MHz) δ: 169.8, 155.8 (d, *J* = 344.6 Hz), 143.3 (d, *J* = 8.0 Hz), 136.4 (d, *J* = 4.0 Hz), 134.3, 131.6, 130.6, 130.0 (q, *J* = 307.3 Hz), 122.6 (d, *J* = 58.1 Hz), 83.4, 47.1, 41.0, 36.9, 36.1, 30.9; IR (NaCl): 2913, 2855, 1809, 1732, 1589, 1239, 1159, 1111, 1007, 768, 758, 690 cm^{-1}; HRMS (ESI): Calcd. for $C_{21}H_{21}BrF_4NaO_3S$ $[M+Na]^+$: 531.0229; Found: 531.0237.

7.14 4-nitrobenzyl 3-(2-(fluorocarbonyl)phenyl)-2-((trifluoromethyl)thio)propanoate (4n);

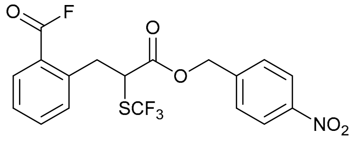

Following the general procedure, using 4-nitrobenzyl 1-oxo-2,3-dihydro-1H-indene-2-carboxylate **1n** (31.1 mg, 0.1 mmol, 1.0 equiv) and CF_3-DAST (0.45 mL, 2.0 equiv) in dioxane (1 mL), the reaction mixture was stirred at –10 °C for overnight. The crude product was purified by flash column chromatography (Hex: AcOEt: DCM = 8:1:1) to provide the title compound **4n** as a yellow oil in 36% yield (15.5 mg). ^{1}H NMR ($CDCl_3$, 500 MHz) δ: 8.10—8.13 (m,

2H), 8.00 (dd, 1H, *J* = 7.5 Hz, *J* = 1.0 Hz), 7.48—7.52 (m, 1H), 7.37—7.40 (m, 1H), 7.31 (d, 2H, *J* = 9.0 Hz), 7.25 (d, 1H, *J* = 7.5 Hz), 5.09—5.15 (m, 2H), 4.15—4.18 (m, 1H), 3.47—3.55 (m, 2H); ^{19}F NMR ($CDCl_3$, 282 MHz) δ: +27.9 (s, 1F), –40.7 (s, 3F); ^{13}C NMR ($CDCl_3$, 125.7 MHz) δ: 170.1, 156.5 (d, *J* = 345.8 Hz), 147.9, 142.0, 140.8 (d, *J* = 8.4 Hz), 135.2, 133.4 (d, *J* = 4.2 Hz), 133.3, 129.8 (q, *J* = 307.7 Hz), 128.6, 123.9, 123.7 (d, *J* = 60.7 Hz), 66.2, 46.0, 36.8; IR (NaCl): 2924, 2861, 1803, 1746, 1523, 1348, 1237, 1160, 1107, 1008, 740, 696, 647 cm^{-1}; HRMS (ESI): Calcd. for $C_{18}H_{13}F_4NNaO_5S$ $[M+Na]^+$: 454.0348; Found: 454.0340.

7.15 Methyl 3-phenyl-2-((trifluoromethyl)thio)propanoate (4o);

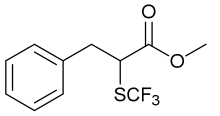

Following the general procedure, using methyl 2-benzyl-3-oxobutanoate **1o** (41.2 mg, 0.2 mmol, 1.0 equiv) and CF_3-DAST (1.8 mL, 4.0 equiv) in DMF (2 mL), the reaction mixture was stirred at 50 °C for overnight. After cooling to room temperature, quenched by addition of water (10 mL), extracted with ethyl acetate (3 x 20 mL), dried over with Na_2SO_4 and then concentrated in vacuo. The crude product was purified by flash column chromatography (Hex: AcOEt =10:1) to provide the title compound **4o** as a yellow oil in 19% yield (10.1 mg). ^{1}H NMR ($CDCl_3$, 500 MHz) δ: 7.31—7.33 (m, 2H), 7.26—7.28 (m, 1H), 7.17—7.19 (m, 2H), 4.01—4.04 (m, 1H), 3.68 (s, 3H), 3.23 (dd, 1H, *J* = 14.0 Hz, *J* = 9.5 Hz), 3.12 (dd, 1H, *J* = 14.0 Hz, *J* = 6.5 Hz); ^{19}F NMR ($CDCl_3$, 282 MHz) δ: –41.0 (s, 3F); ^{13}C NMR ($CDCl_3$, 125.7 MHz) δ: 170.8, 136.1, 130.1 (q, *J* = 307.0 Hz), 129.1, 128.8, 127.6, 53.0, 47.5, 38.4; IR (NaCl): 3032, 2955, 1747, 1438, 1162, 1110, 845, 747, 699 cm^{-1}; HRMS (ESI): Calcd. For $C_{11}H_{11}F_3NaO_2S$ $[M+Na]^+$: 287.0330; Found: 287.0332.

8. X-ray crystallography data for the 4h (CCDC 1415531)

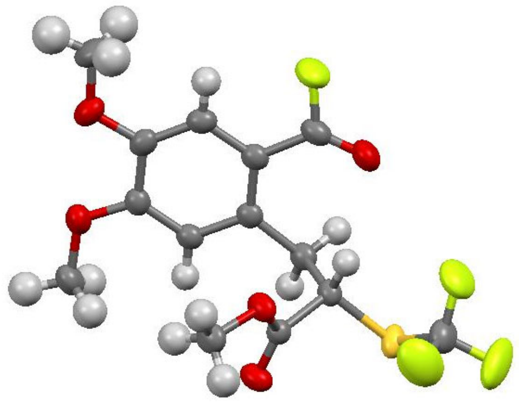

EXPERIMENTAL DETAILS

A. Crystal Data

Empirical Formula	$C_{14}H_{14}F_4O_5S$
Formula Weight	370.32
Crystal Color, Habit	colorless, block
Crystal Dimensions	0.300 X 0.200 X 0.200 mm
Crystal System	monoclinic
Lattice Type	Primitive
Lattice Parameters	a = 8.1328(3) Å<br>b = 19.0087(6) Å<br>c = 10.7270(4) Å<br>β = 104.6528(9) ^{o}<br>V = 1604.4(1) $Å^3$
Space Group	$P2_1/n$ (#14)
Z value	4
D_{calc}	1.533 g/cm^3
F_{000}	760.00
$\mu(MoK\alpha)$	2.665 cm^{-1}

9. General procedure and product characterization data for 5a;

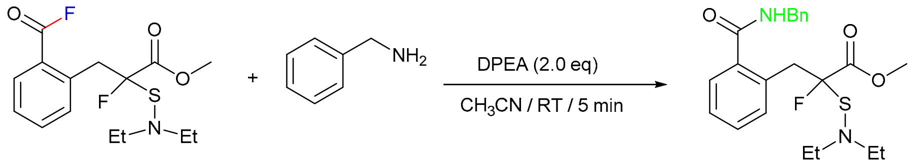

A flame-dried vessel was successively charged, under nitrogen, with methyl 2-((diethylamino)thio)-2-fluoro-3-(2-(fluorocarbonyl)phenyl)propanoate (**2b**) (66.2 mg, 0.2 mmol) and anhydrous acetonitrile (2.0 mL), stirred at room temperature for 5 min, then benzylamine (44 μL, 0.4 mmol, 2.0 equiv) and diisopropylethylamine (68 μL, 0.4 mmol, 2.0 equiv) were added slowly by syringe. The reaction mixture was stirred at room temperature for 5 min, quenched with water (10 mL), extracted with ethyl acetate (3 x 20 mL), dried over with Na_2SO_4, concentrated in vacuo. The crude product was purified by flash column chromatography (Hex: AcOEt =5:1—3:1) to provide the target product **5a** as a colourless oil in 94% yield (78.6 mg). ^{1}H NMR ($CDCl_3$, 500 MHz) δ: 7.39—7.41 (m, 3H), 7.31—7.36 (m, 2H), 7.29—7.30 (m, 1H), 7.24—7.27 (m, 3H), 6.49 (s, 1H), 4.58—4.66 (m, 2H), 3.71—3.76 (m, 4H), 3.53 (dd, 1H, J = 27.2 Hz, J = 15.0 Hz), 2.92—2.98 (m, 4H), 1.07 (t, 6H, J = 7.0 Hz); ^{19}F NMR ($CDCl_3$, 282 MHz) δ: –137.9 (dd, 1F, J = 26.7 Hz, J = 12.8 Hz); ^{13}C NMR ($CDCl_3$, 125.7 MHz) δ: 169.8, 169.0 (d, J = 31.8 Hz), 138.1, 131.8, 131.1, 130.0, 128.8, 128.1, 127.7, 127.6, 127.5, 108.6 (d, J = 232.6 Hz), 52.8, 52.2, 44.2, 36.3 (d, J = 21.9 Hz), 13.7; IR (NaCl): 3307, 2971, 2931, 2360, 2341, 1749, 1652, 1539, 1268, 911, 731 cm^{-1}; HRMS (ESI): Calcd. For $C_{22}H_{27}FN_2NaO_3S$ $[M+Na]^+$: 441.1624; Found: 441.1621.

10. General procedure and product characterization data for 5b;

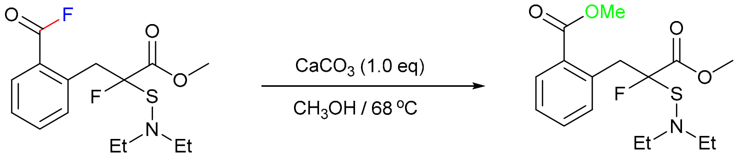

A flame-dried vessel was successively charged, under nitrogen, with methanol (2.0

mL) and $CaCO_3$ (20.0 mg, 0.2 mmol, 1.0 equiv), stirred at 45 °C for 10 min, the solution of methyl 2-((diethylamino)thio)-2-fluoro-3-(2-(fluorocarbonyl)phenyl)propanoate (**2b**) (66.2 mg, 0.2 mmol) in methanol (1.0 mL) was added slowly by syringe at same temperature. Then the reaction mixture was stirred at 68 °C for 24 hours. The mixture was cooled to room temperature for filtration and concentrated in vacuo. The crude product was purified by flash column chromatography (Hex: AcOEt =10:1—6:1) to provide the target product **5b** as a colourless oil in 57% yield (39.1 mg). ^{1}H NMR ($CDCl_3$, 500 MHz) δ: 7.80 (dd, 1H, *J* = 8.5 Hz, *J* = 1.5 Hz), 7.38—7.42 (m, 1H), 7.28—7.32 (m, 2H), 3.88—3.93 (m, 4H), 3.76 (dd, 1H, *J* = 26.5 Hz, *J* = 14.5 Hz), 3.70 (s, 3H), 2.95—3.04 (m, 4H), 1.07 (t, 6H, *J* = 7.0 Hz); ^{19}F NMR ($CDCl_3$, 282 MHz) δ: –140.4 (dd, 1F, *J* = 24.8 Hz, *J* = 11.8 Hz); ^{13}C NMR ($CDCl_3$, 125.7 MHz) δ: 169.0 (d, *J* = 31.8 Hz), 168.4, 134.4, 132.2, 131.8, 131.6, 130.5, 127.4, 107.7 (d, *J* = 234.1 Hz), 52.7, 52.2, 36.8 (d, *J* = 21.4 Hz), 13.7; IR (NaCl): 2971, 2952, 2360, 2341, 1719, 1435, 1256, 1085, 668 cm^{-1}; HRMS (ESI): Calcd. for $C_{16}H_{22}FNNaO_4S$ $[M+Na]^+$: 366.1151 Found: 366.1155.

11. General procedure and product characterization data for 6a;

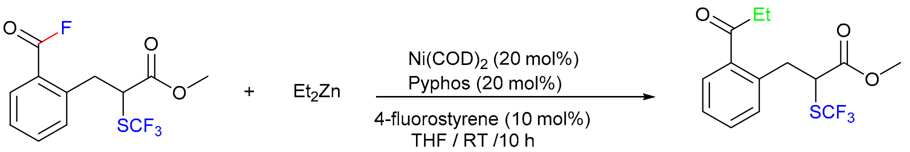

4b 1.0 eq **6a** (59%)

A flame-dried round bottom flask was charged with $Ni(COD)_2$ (5.5 mg, 0.02 mmol, 20 mol%) and pyphos (7.0 mg, 24 mol%) in an inert atmosphere (N_2) glove box. Upon removal from the glove box, anhydrous THF (0.5 mL) was added via syringe under an atmosphere of argon and stirred at ambient temperature for 15 minutes. 4-fluorostyrene (2.4μL, 0.02 mmol, 20 mol%) was introduced via syringe followed by a solution of diethylzinc (0.1 mL,1.0 equiv, 1M solution in Hexane). Then the solution of methyl 3-(2-(fluorocarbonyl)phenyl)-2-((trifluoromethyl)thio)propanoate (**4b**)

(31.0 mg, 0.1 mmol) in THF (0.5 mL) was added. The reaction mixture was stirred at the room temperature for 12 hours, quenched with 1M HCl (4 mL), extracted with diethyl ether (3 x 10 mL). The combined organic layers were washed with brine, dried over Na_2SO_4, filtered, concentrated in vacuo. The crude product was purified by flash column chromatography (Hex: AcOEt =10:1) to provide the target product **6a** as a colourless oil in 59% yield (18.8 mg). ^{1}H NMR ($CDCl_3$, 400 MHz) δ: 7.78 (dd, 1H, J = 7.6 Hz, J = 1.6 Hz), 7.42—7.46 (m, 1H), 7.35—7.40 (m, 1H), 7.25 (dd, 1H, J = 7.6 Hz, J = 1.2 Hz), 4.28 (dd, 1H, J = 8.2 Hz, J = 7.4 Hz), 3.67 (s, 3H), 3.32—3.42 (m, 2H), 2.91—3.01 (m, 2H), 1.21 (t, 3H, J = 7.4 Hz); ^{19}F NMR ($CDCl_3$, 282 MHz) δ: –40.8 (s, 3F); ^{13}C NMR ($CDCl_3$, 125.7 MHz) δ: 204.3, 171.4, 137.4, 136.5, 132.9, 131.8, 130.1 (q, J = 307.6 Hz), 129.6, 127.7, 52.8, 47.0, 36.8, 34.4, 8.6; IR (NaCl): 2940, 1744, 1686, 1438, 1159, 1112, 1032, 952, 756 cm^{-1}; HRMS (ESI): Calcd. for $C_{14}H_{15}F_3NaO_3S$ [M+Na]$^+$: 343.0592; Found: 343.0603.

12. General procedure and product characterization data for 6b;

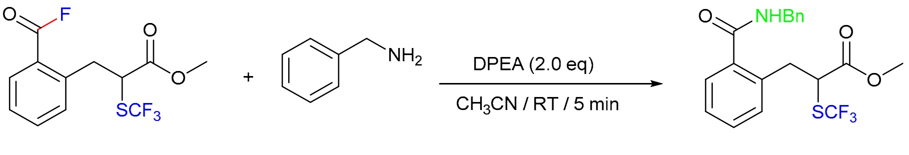

A flame-dried vessel was successively charged, under nitrogen, with methyl 3-(2-(fluorocarbonyl)phenyl)-2-((trifluoromethyl)thio)propanoate (**4b**) (31.0 mg, 0.1 mmol) and anhydrous acetonitrile (1.0 mL), stirred at room temperature for 5 min, then the benzylamine (22 μL, 0.2 mmol, 2.0 equiv) and diisopropylethylamine (34 μL, 0.2 mmol, 2.0 equiv) was added slowly by syringe. The reaction mixture was stirred at room temperature for 5 min, quenched with water (10 mL), extracted with ethyl acetate (3 x 20 mL), dried over with Na_2SO_4, concentrated in vacuo. The crude product was purified by flash column chromatography (Hex: AcOEt =20:1—10:1) to provide the target product **6b** as a colourless oil in 73% yield (28.9 mg). ^{1}H NMR ($CDCl_3$, 400 MHz) δ: 7.42 (dd, 1H, J = 7.6 Hz, J = 1.2 Hz), 7.33—7.39 (m, 5H),

7.26—7.32 (m, 2H), 7.24 (dd, 1H, J = 7.6 Hz, J = 1.2Hz), 6.29 (s, 1H), 4.57—4.66 (m, 2H), 4.37 (dd, 1H, J = 8.4 Hz, J = 7.6 Hz), 3.66 (s, 3H), 3.42 (dd, 1H, J = 14.0 Hz, J = 8.8 Hz), 3.42 (dd, 1H, J = 13.6 Hz, J = 7.6 Hz); ^{19}F NMR ($CDCl_3$, 282 MHz) δ: –40.7 (s, 3F); ^{13}C NMR ($CDCl_3$, 125.7 MHz) δ: 171.3, 169.1, 138.0, 136.2, 135.2, 131.6, 130.4, 130.0 (q, J = 307.4 Hz), 128.9, 128.0, 127.8, 127.7, 127.3, 52.9, 47.0, 44.2, 35.7; IR (NaCl): 3298, 3064, 3031, 2953, 1743, 1641, 1529, 1156, 1112, 752, 699, 660 cm^{-1}; HRMS (ESI): Calcd. for $C_{19}H_{18}F_3NNaO_3S$ $[M+Na]^+$: 420.0857; Found: 420.0848.

13. General procedure and product characterization data for 6c;

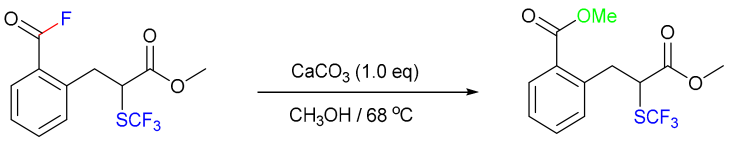

4b **6c** (72%)

A flame-dried vessel was successively charged, under nitrogen, with methanol (1.0 mL) and $CaCO_3$ (10.0 mg, 0.1 mmol, 1.0 equiv), stirred at 45 °C for 10 min, the solution of methyl 3-(2-(fluorocarbonyl)phenyl)-2-((trifluoromethyl)thio)propanoate (**4b**) (31.0 mg, 0.1 mmol) in methanol (0.5 mL) was added slowly by syringe at same temperature. Then the reaction mixture was stirred at 68 °C for 24 hours. Which was cooled to room temperature, filtration, concentrated in vacuo. The crude product was purified by flash column chromatography (Hex: AcOEt =20:1) to provide the target product **6c** as a colourless oil in 72% yield (23.1 mg). ^{1}H NMR ($CDCl_3$, 400 MHz) δ: 8.01 (dd, 1H, J = 7.6 Hz, J = 1.2 Hz), 7.44—7.48 (m, 1H), 7.33—7.38 (m, 1H), 7.24 (dd, 1H, J = 7.6 Hz, J = 0.8 Hz), 4.28 (dd, 1H, J = 8.4 Hz, J = 7.0 Hz), 3.92 (s, 3H), 3.66 (s, 3H), 3.45—3.56 (m, 2H); ^{19}F NMR ($CDCl_3$, 282 MHz) δ: –40.9 (s, 3F); ^{13}C NMR ($CDCl_3$, 125.7 MHz) δ: 171.3, 167.4, 138.1, 132.6, 132.5, 131.6, 130.1 (q, J = 307.2 Hz), 129.5, 127.8, 52.9, 52.4, 46.9, 37.1; IR (NaCl): 3003, 2956, 2845, 1745, 1720, 1437, 1269, 1113, 844, 751, 707, 664 cm^{-1}; HRMS (ESI): Calcd. for $C_{13}H_{13}F_3NaO_4S$ $[M+Na]^+$: 345.0384 Found: 345.0370.

14. Typical procedure preparation of C_6F_5-DAST reagents

A flame-dried vessel was successively charged, under nitrogen, with diisopropylethylamine (0.17 mL, 1.0 mmol) and anhydrous dichloromethane (2 mL). The resulting mixture was cooled to –10 °C, the diethylaminosulfurtrifluoride (0.15 mL, 1.0 mmol) was added slowly by syringe, stirred for 15 min at same temperature, then the trimethyl(perfluorophenyl)silane (0.19 mL, 1.0 mmol) was added slowly by syringe, and stirring for two hours under the same reaction temperature, after two hours directly use for next step reactions without purifacation.

15. General procedure and product characterization data for 7b;

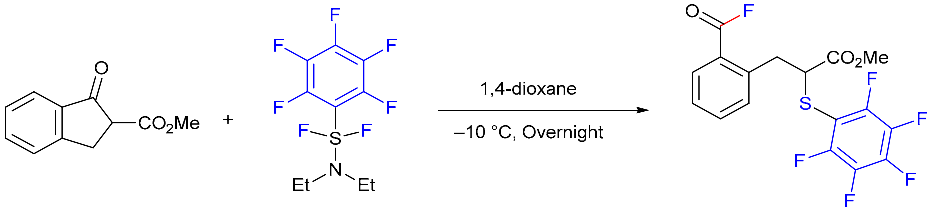

1b

7b

A flame-dried vessel was successively charged, under nitrogen, with *β*-keto esters **1b** (19.0 mg, 0.1 mmol, 1.0 equiv) and anhydrous 1,4-dioxane (1.0 mL). The solution was cooled to –10 °C and the 0.5M solution of C_6F_5-DAST (0.2 mmol, 2.0 equiv, 0.46 mL) in CH_2Cl_2 was added slowly by syringe. Then the reaction mixture was stirred at –10 °C or room temperature for overnight, quenched by addition of water (10 mL), extracted with ethyl acetate (3 x 20 mL), dried over with Na_2SO_4 and then concentrated in vacuo. The crude product was purified by flash column chromatography (Hex: AcOEt = 12:1) to provide the title compound **7b** as a colourless oil in 53% yield (21.6 mg). ^{1}H NMR ($CDCl_3$, 400 MHz) δ: 8.03 (dd, 1H, *J* = 8.0 Hz, *J* = 1.6 Hz), 7.58—7.62 (m, 1H), 7.38—7.45 (m, 2H), 3.91—3.94 (m, 1H), 3.62 (s, 3H), 3.44—3.58 (m, 2H); ^{19}F NMR ($CDCl_3$, 282 MHz) δ: +28.0 (s, 1F), –

131.01— –131.08 (m, 2F), –149.94— –150.09 (m, 1F), –160.84— –160.97 (m, 2F); ^{13}C NMR ($CDCl_3$, 125.7 MHz) δ: 170.7, 156.4 (d, J = 345.5 Hz), 149.3, 147.3, 141.8 (d, J = 8.1 Hz), 141.6, 138.7, 136.8, 135.1, 133.2, 133.0 (d, J = 4.1 Hz), 128.2, 123.4 (d, J = 56.5 Hz), 106.2, 52.7, 50.0, 36.8; IR (NaCl): 2956, 2920, 2862, 1804, 1738, 1515, 1489, 1237, 1093, 1008, 863, 756, 696 cm^{-1}; HRMS (ESI): Calcd. for $C_{17}H_{10}F_6NaO_3S$ $[M+Na]^+$: 431.0153; Found: 431.0155.

16. General procedure and product characterization data for 9;

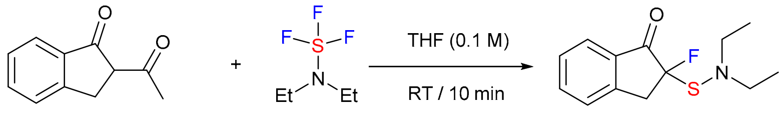

8 DAST **9**: 63%

A flame-dried vessel was successively charged, under nitrogen, with 2-acetyl-2,3-dihydro-1H-inden-1-one **8** (34.8 mg, 0.2 mmol, 1.0 equiv) and anhydrous THF (2.0 mL). The solution was cooled to 0 °C and the DAST (0.4 mmol, 2.0 equiv) was added slowly by syringe. Then the reaction mixture was stirred at room temperature for 10 min, quenched by addition of water (10 mL), extracted with ethyl acetate (3 x 20 mL), dried over with Na_2SO_4 and then concentrated in vacuo. The crude product was purified by flash column chromatography (Hex: AcOEt = 5:1) to provide the title compound **9** as a yellow oil in 63% yield (31.9 mg). ^{1}H NMR ($CDCl_3$, 500 MHz) δ: 7.81 (d, 1H, J = 7.5 Hz), 7.61—7.64 (m, 1H), 7.43 (t, 1H, J = 7.5 Hz), 7.37 (d, 1H, J = 7.5 Hz), 3.26—3.39 (m, 2H), 3.04 (s, 4H), 1.08 (s, 6H); ^{19}F NMR ($CDCl_3$, 282 MHz) δ: –147.0 (d, 1F, J = 12.9 Hz); ^{13}C NMR ($CDCl_3$, 125.7 MHz) δ: 1195.4 (d, J = 24.8 Hz), 147.2 (d, J = 4.5 Hz), 135.7, 133.6 (d, J = 2.7 Hz), 128.6, 126.4, 125.3, 108.3 (d, J = 241.7 Hz), 52.4 (d, J = 17.8 Hz), 36.7 (d, J = 23.5 Hz), 14.1; IR (NaCl): 2969, 2930, 2360, 2341, 1732, 1717, 1558, 1540, 1084, 668 cm^{-1}; HRMS (ESI): Calcd. for $C_{13}H_{17}FNOS$ $[M+H]^+$: 254.1015; Found: 254.1035. This compound is not so stable under column chromatography on silica-gel.

17. General procedure and product characterization data for 10;

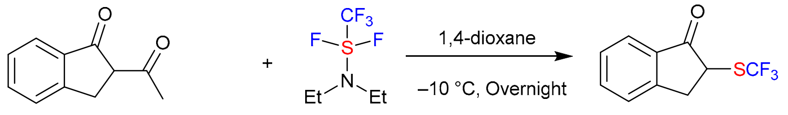

A flame-dried vessel was successively charged, under nitrogen, with 2-acetyl-2,3-dihydro-1H-inden-1-one **8** (34.8 mg, 0.2 mmol, 1.0 equiv) and anhydrous 1,4-dioxane (2.0 mL). The solution was cooled to –10 °C and the 0.5M solution of CF_3-DAST (0.4 mmol, 2.0 equiv, 0.90 mL taken from the solution above mentioned) in CH_2Cl_2 was added slowly by syringe. Then the reaction mixture was stirred at –10 °C or room temperature for overnight, quenched by addition of water (10 mL), extracted with ethyl acetate (3 x 20 mL), dried over with Na_2SO_4 and then concentrated in vacuo. The crude product was purified by flash column chromatography (Hex: AcOEt = 10:1) to provide the title compound **10** as a yellow oil in 54% yield (25.1 mg). ^{1}H NMR ($CDCl_3$, 500 MHz) δ: 7.82 (d, 1H, J = 8.0 Hz), 7.66—7.69 (m, 1H), 7.47—7.49 (m, 1H), 7.42—7.45 (m, 1H), 4.24 (dd, 1H, J = 8.0 Hz, J = 4.5 Hz), 3.79 (dd, 1H, J = 17.5 Hz, J = 8.0 Hz), 3.37 (dd, 1H, J = 17.5 Hz, J = 4.5 Hz); ^{19}F NMR ($CDCl_3$, 282 MHz) δ: –40.0 (s, 3F); ^{13}C NMR ($CDCl_3$, 125.7 MHz) δ: 199.3, 151.5, 134.6, 130.7 (q, J = 307.4 Hz), 128.4, 126.5, 124.9,47.5, 36.4; IR (NaCl): 2925, 2360, 2341, 1732, 1717, 1540, 1507, 1110, 668 cm^{-1}; MS (ESI, m/z): 231 [M-H]$^-$. The NMR data are consistent with those reported in the literature. [6]

18. References

1. Cordova, A.; Janda, K. D. *J. Org. Chem.* **2001,** *66***,** 1906.
2. Nieman, J. A.; Keay, B. A. *Tetrahedron Asymmetry* **1995**, *6*, 1575.
3. Smith, A. M. R.; Rzepa, H. S.; White, A. J. P.; Billen, D.; Hii, K. K. *J. Org. Chem.* **2010**, *75*, 3085.
4. Nakajima, M.; Yamamoto, S.; Yamaguchi, Y.; Nakamura, S.; Hashimoto, S.

Tetrahedron **2003**, *59*, 7307.

5. Lian, M.; Li, Z.; Du, J.; Meng, Q.; Gao, Z. *Eur. J. Org. Chem.* **2010***,* 6525.
6. Alazet, S.; Zimmer, L.; Billard, T. *Chem. Eur. J.* **2014**, *20*, 8589.

19. ^{1}H NMR **and** [13]**C NMR spectra for** starting material **1n**;

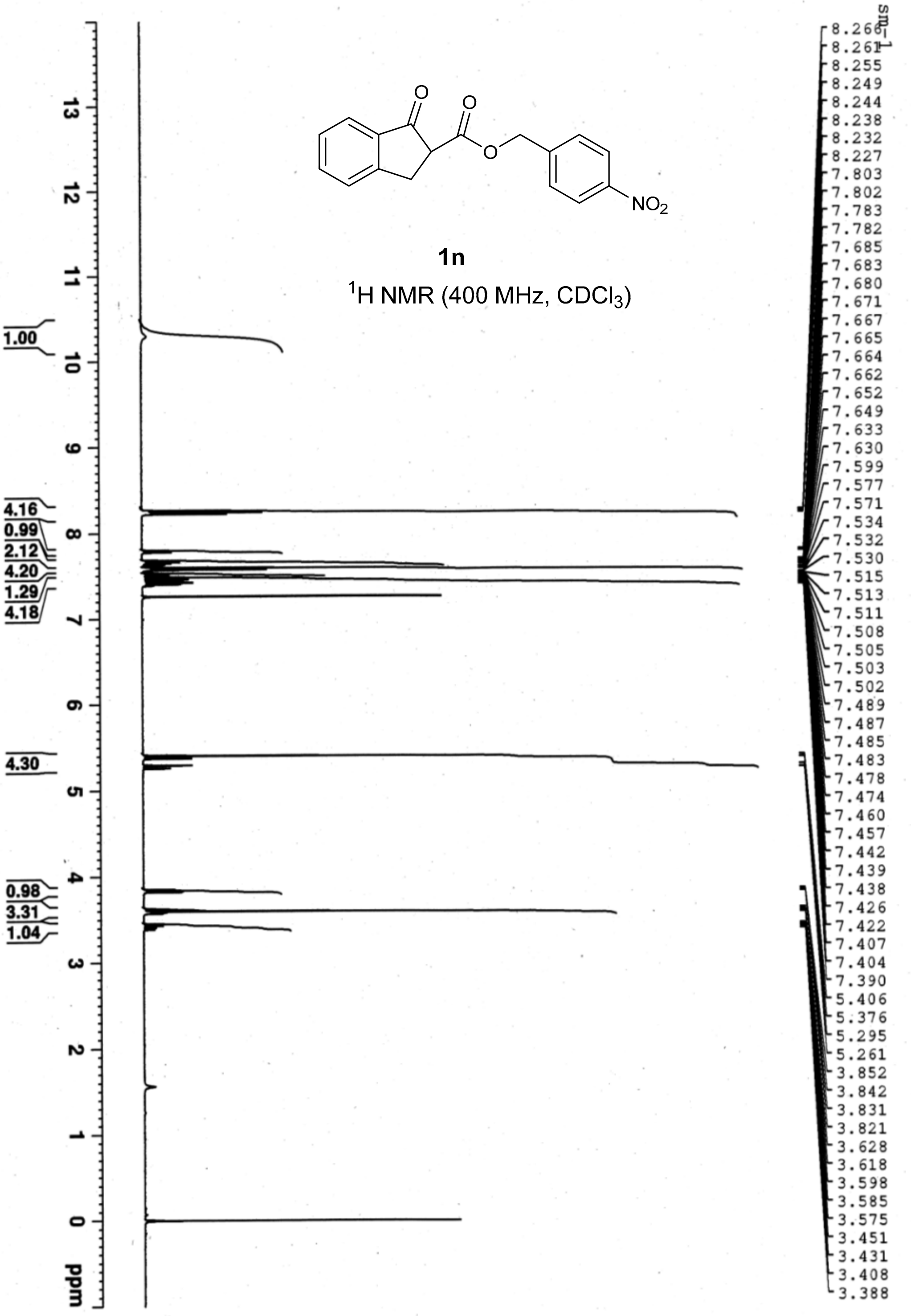

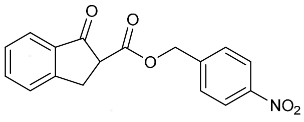

1n

^{13}C NMR (75.5 MHz, $CDCl_3$)

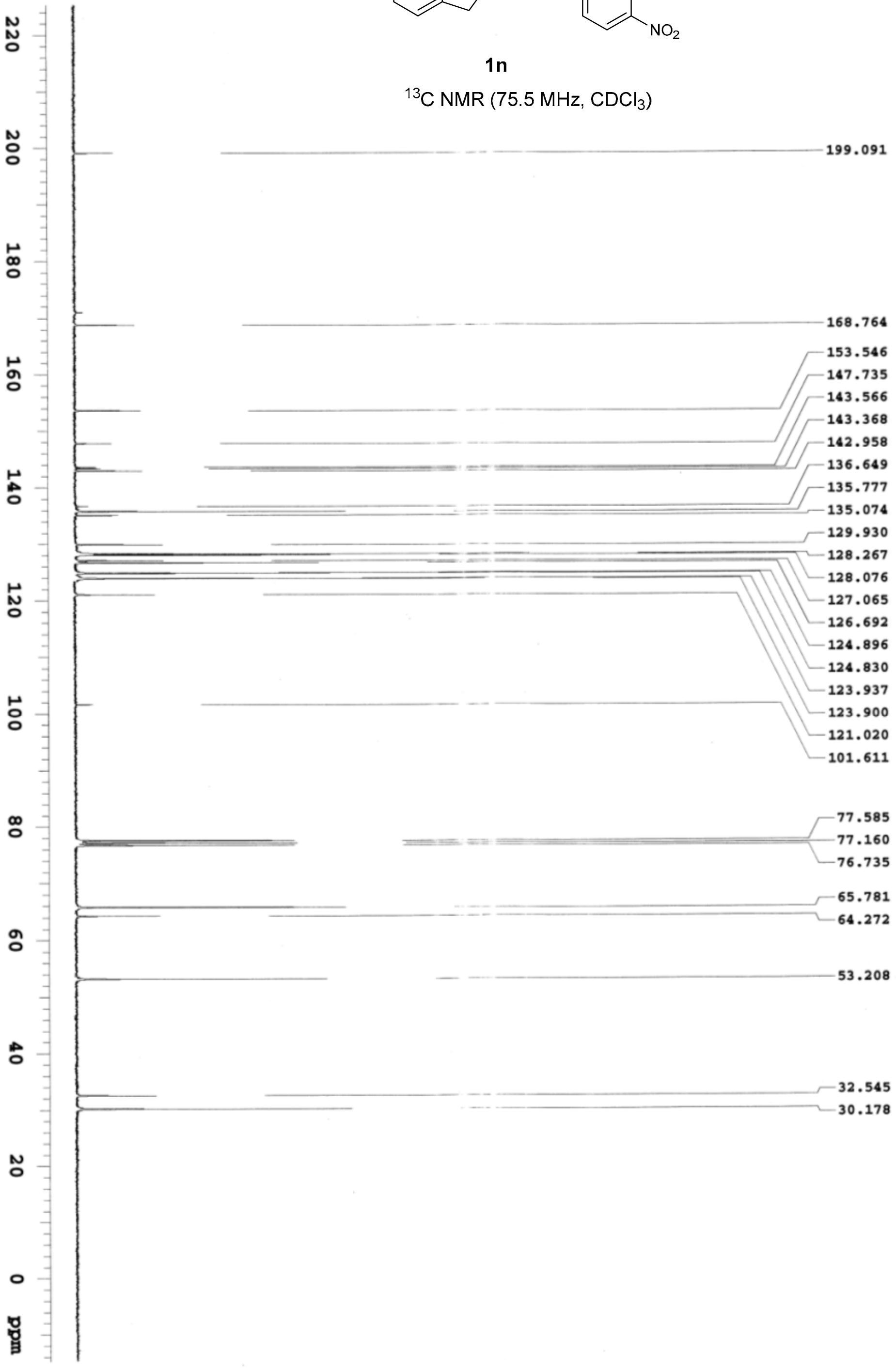

20. ^{1}H NMR, ^{13}C NMR and ^{19}F NMR spectra for doubly fluoro-functionalization compounds 2a—2k, 2o and 3a—3c (Table 1)

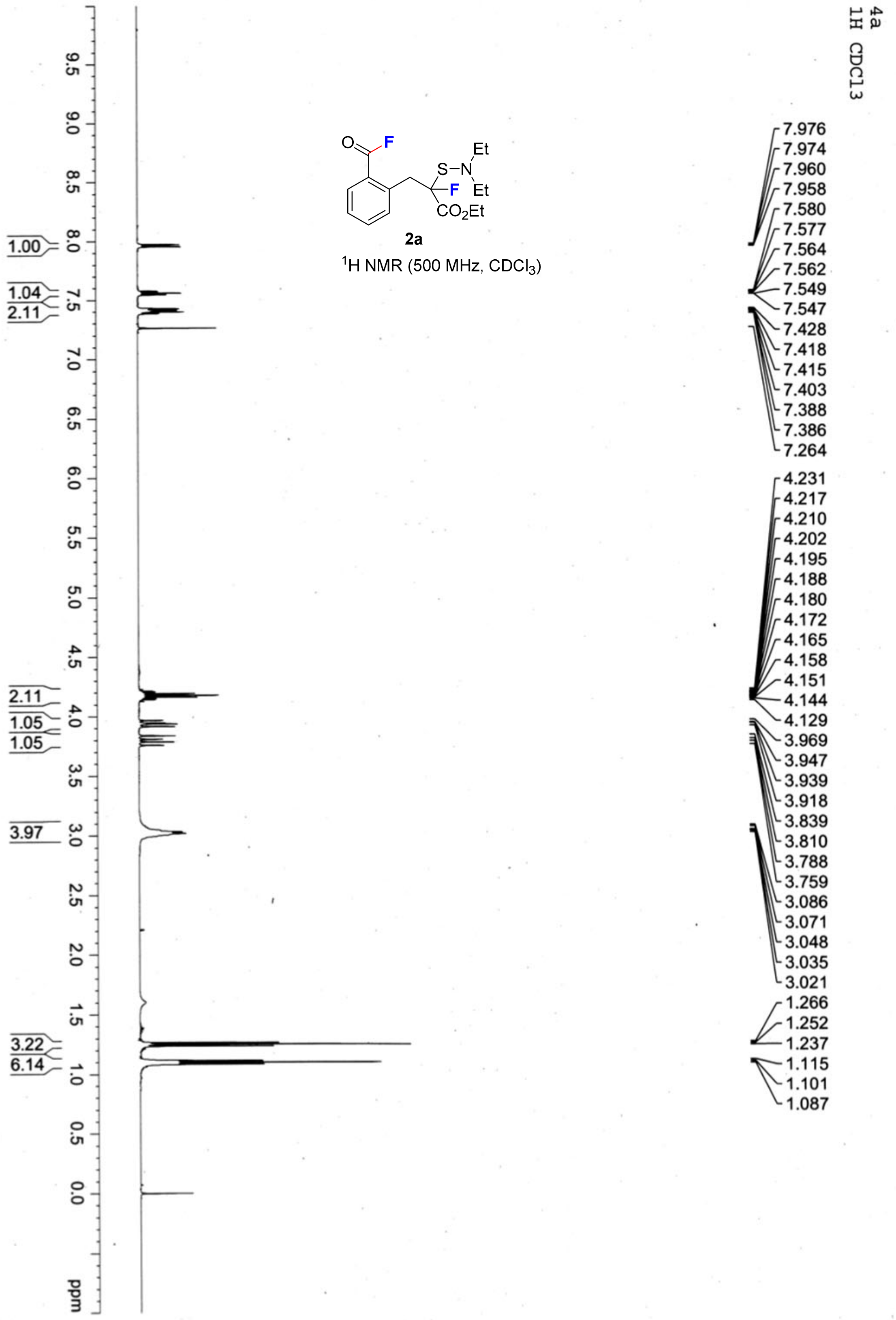

2a
^{1}H NMR (500 MHz, CDCl$_3$)
4a
1H CDCl3
7.976 7.974 7.960 7.958 7.580 7.577 7.564 7.562 7.549 7.547 7.428 7.418 7.415 7.403 7.388 7.386 7.264
4.231 4.217 4.210 4.202 4.195 4.188 4.180 4.172 4.165 4.158 4.151 4.144 4.129 3.969 3.947 3.939 3.918 3.839 3.810 3.788 3.759 3.086 3.071 3.048 3.035 3.021
1.266 1.252 1.237 1.115 1.101 1.087
1.00 1.04 2.11 2.11 1.05 1.05 3.97 3.22 6.14
ppm

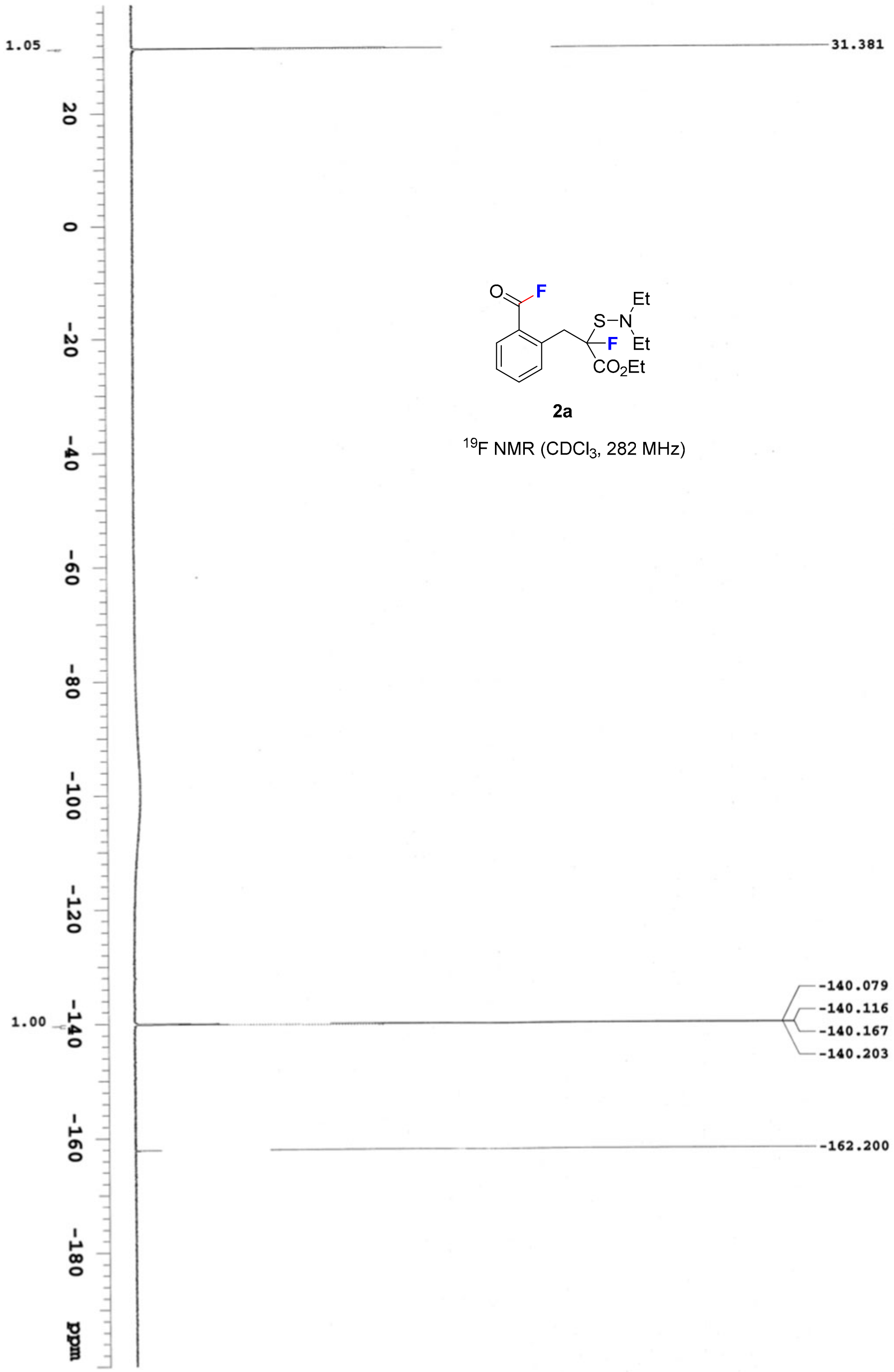

2a

^{19}F NMR ($CDCl_3$, 282 MHz)

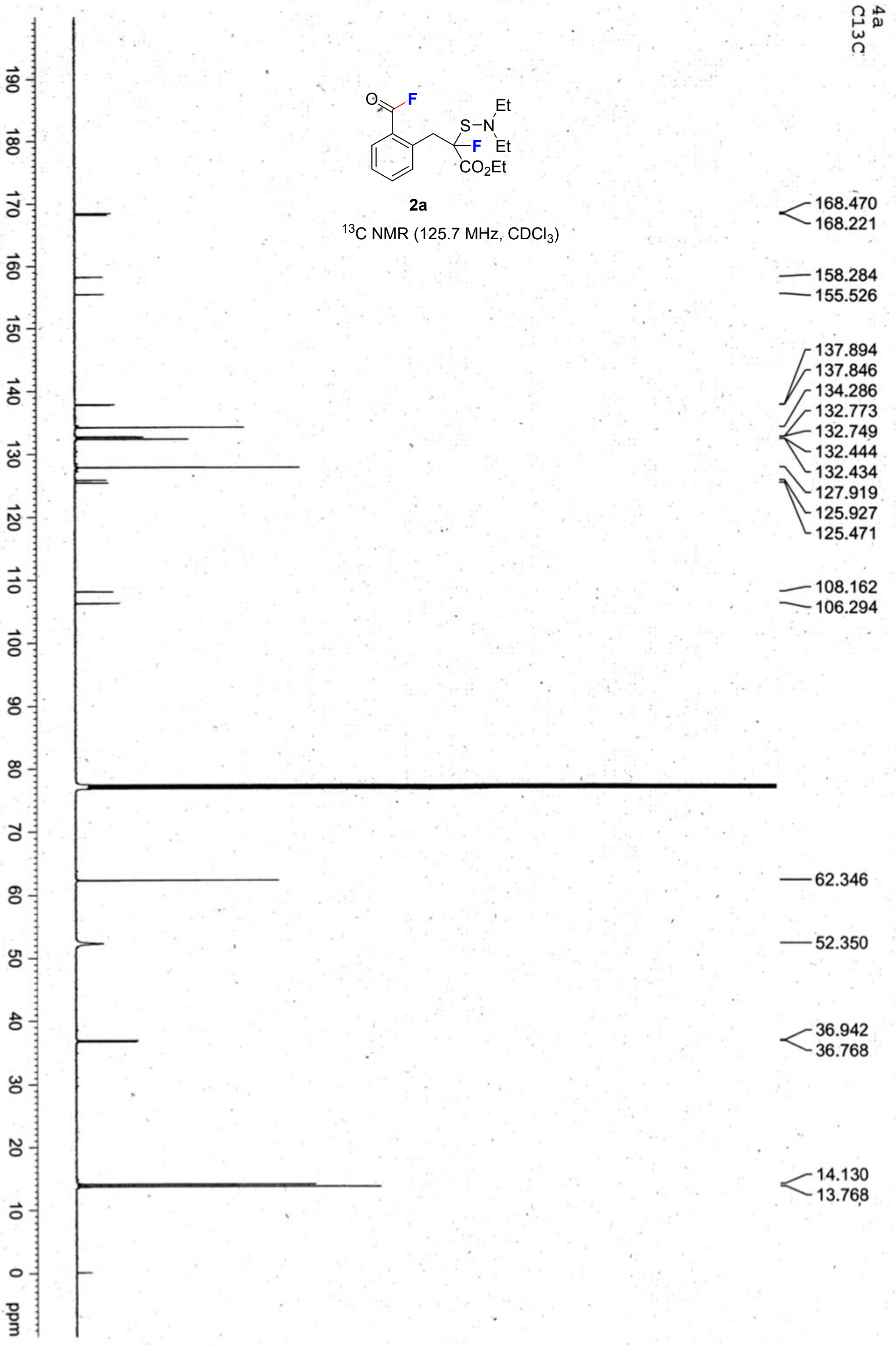

2a

^{13}C NMR (125.7 MHz, $CDCl_3$)

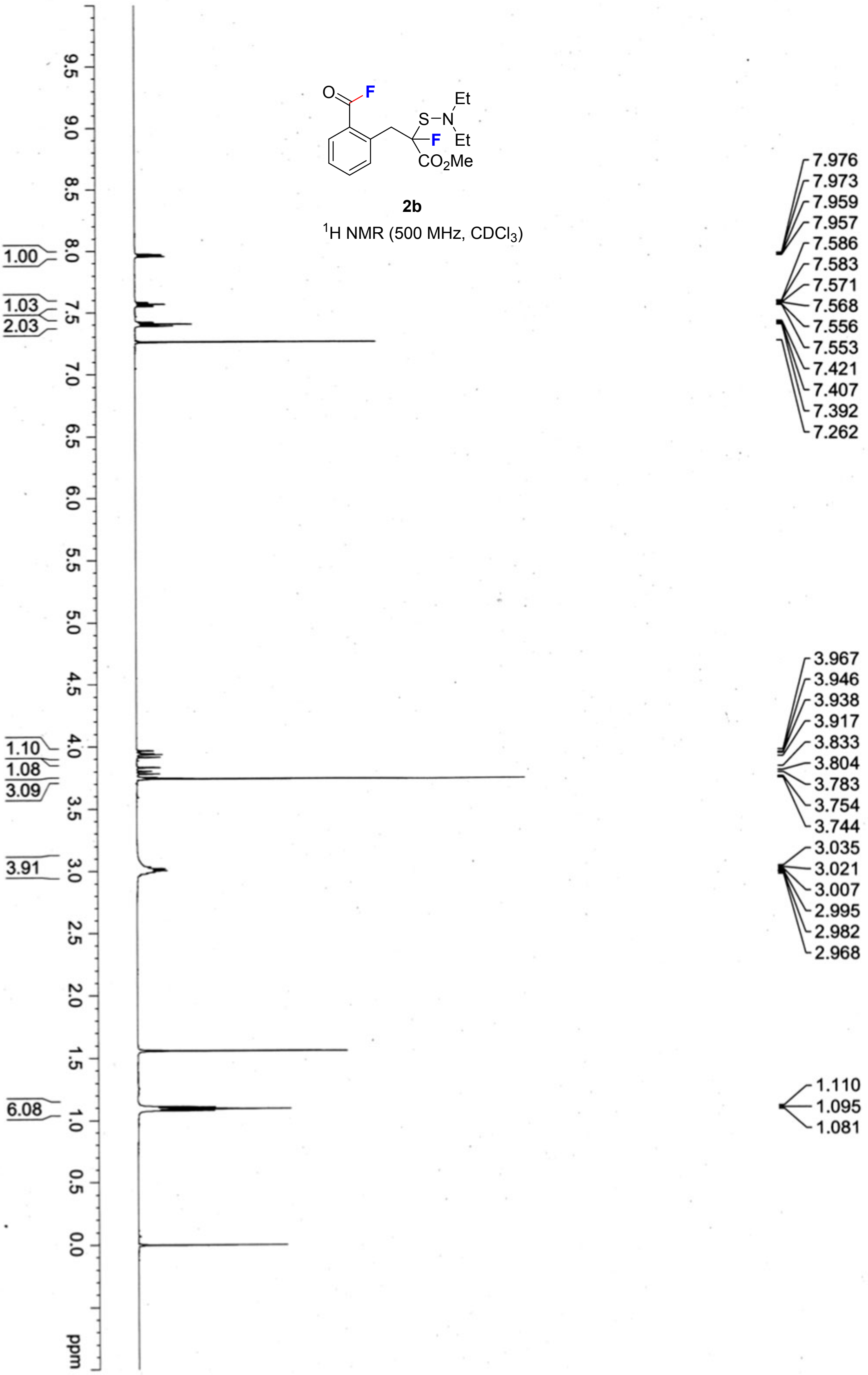

O
F
S
N
Et
Et
F
CO2Me
2b
1H NMR (500 MHz, CDCl3)
7.976
7.973
7.959
7.957
7.586
7.583
7.571
7.568
7.556
7.553
7.421
7.407
7.392
7.262
3.967
3.946
3.938
3.917
3.833
3.804
3.783
3.754
3.744
3.035
3.021
3.007
2.995
2.982
2.968
1.110
1.095
1.081
1.00
1.03
2.03
1.10
1.08
3.09
3.91
6.08
9.5
9.0
8.5
8.0
7.5
7.0
6.5
6.0
5.5
5.0
4.5
4.0
3.5
3.0
2.5
2.0
1.5
1.0
0.5
0.0
ppm

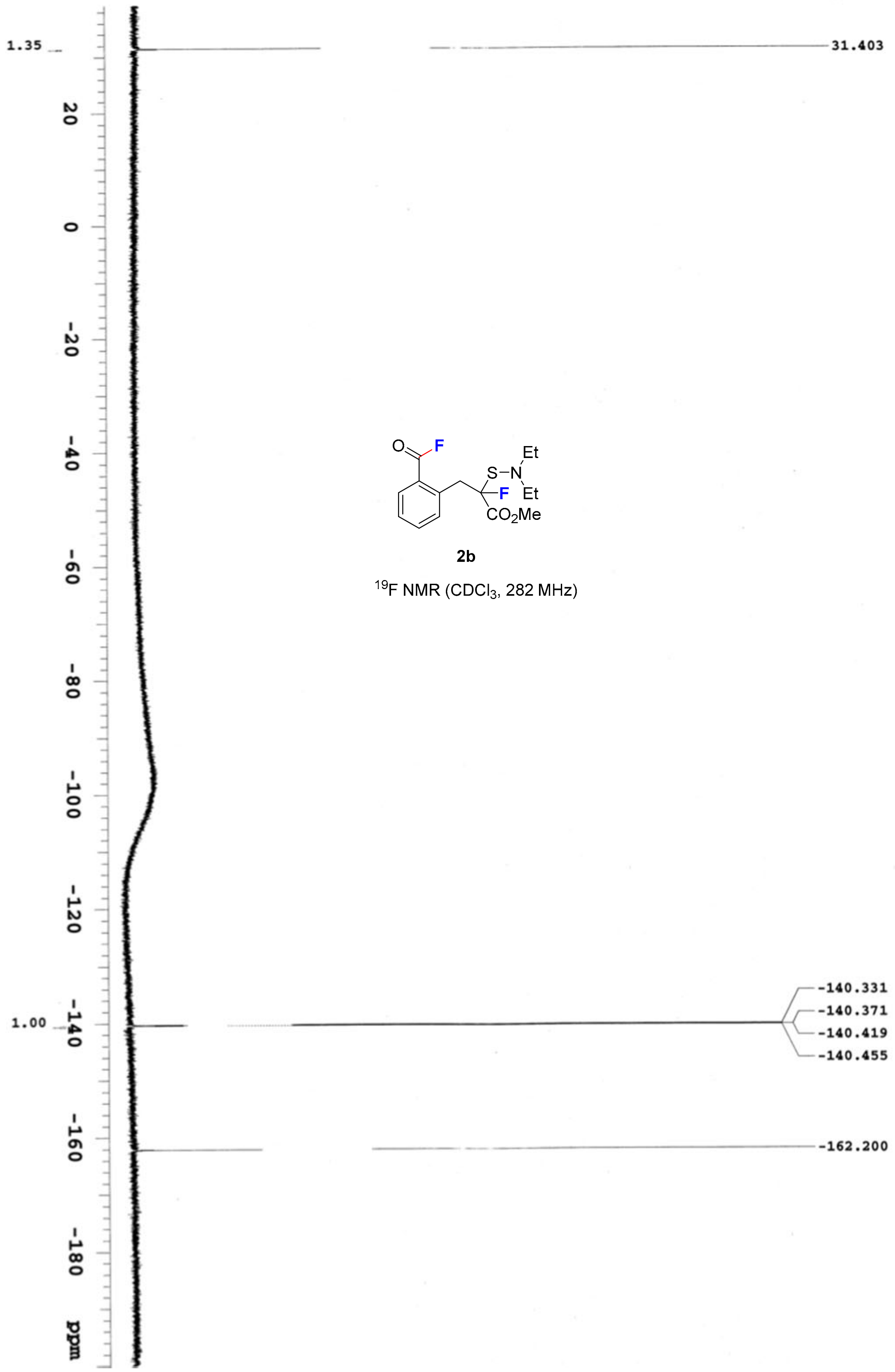

2b

^{19}F NMR ($CDCl_3$, 282 MHz)

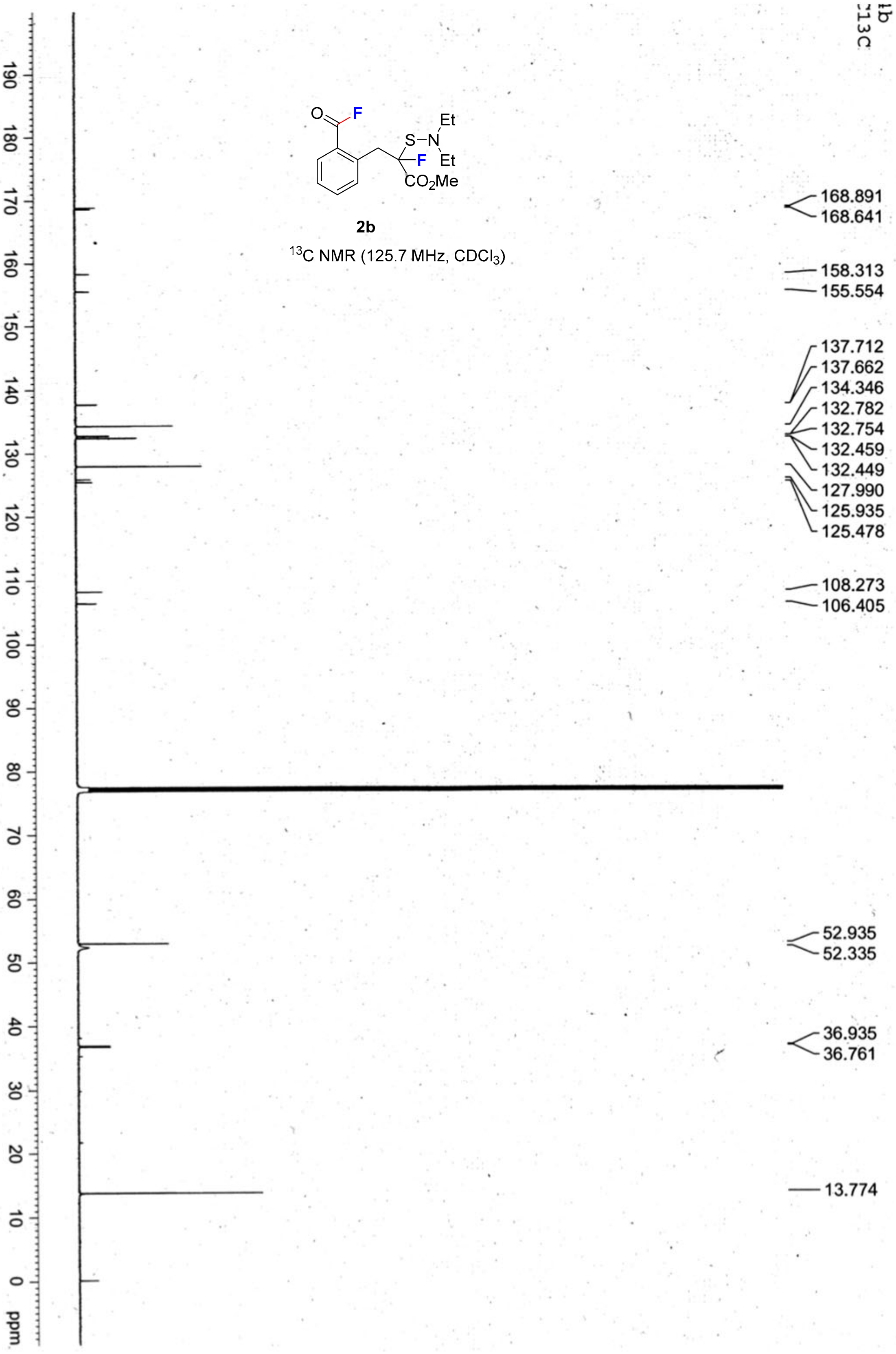

2b

^{13}C NMR (125.7 MHz, $CDCl_3$)

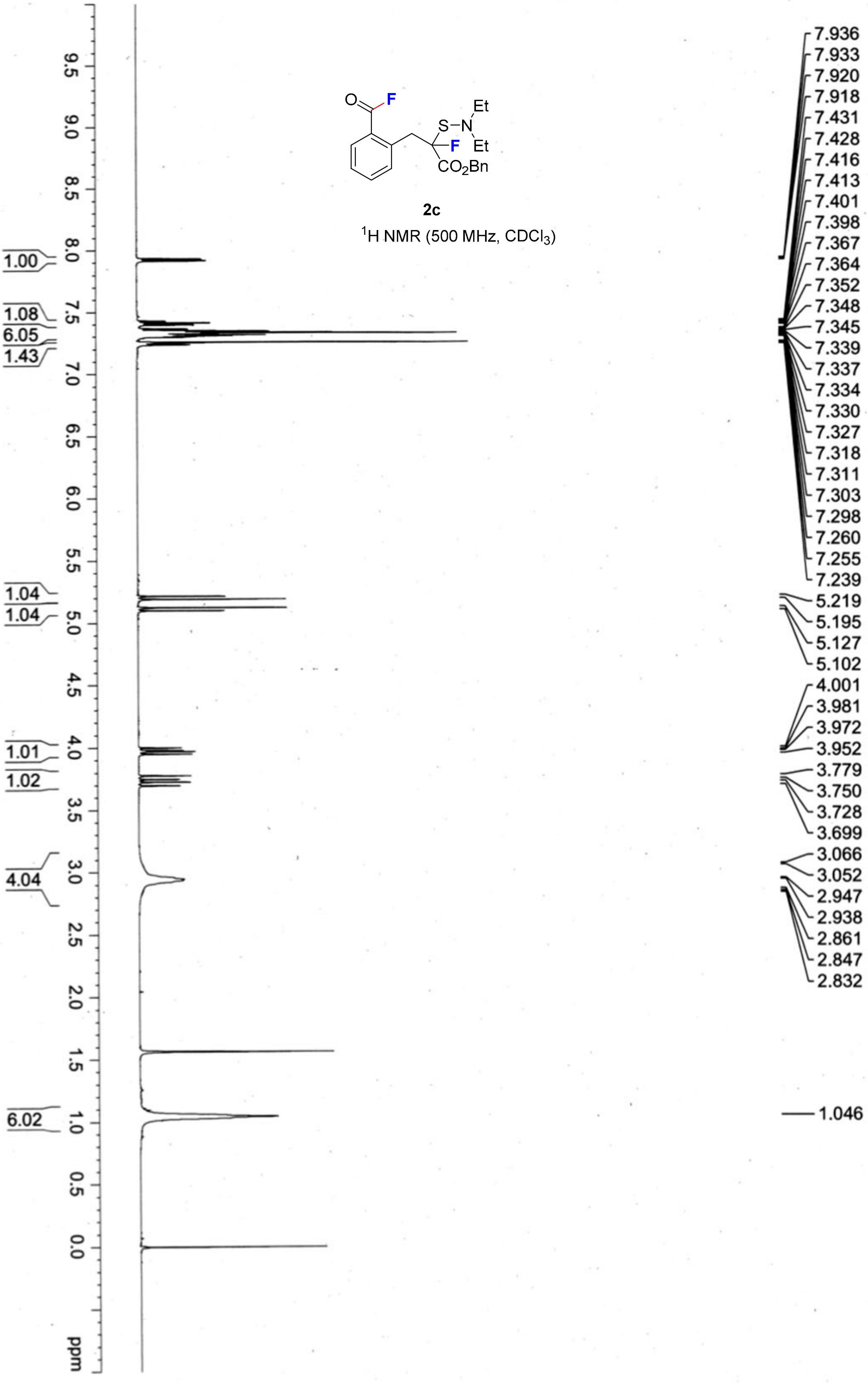

2c

^1H NMR (500 MHz, $CDCl_3$)

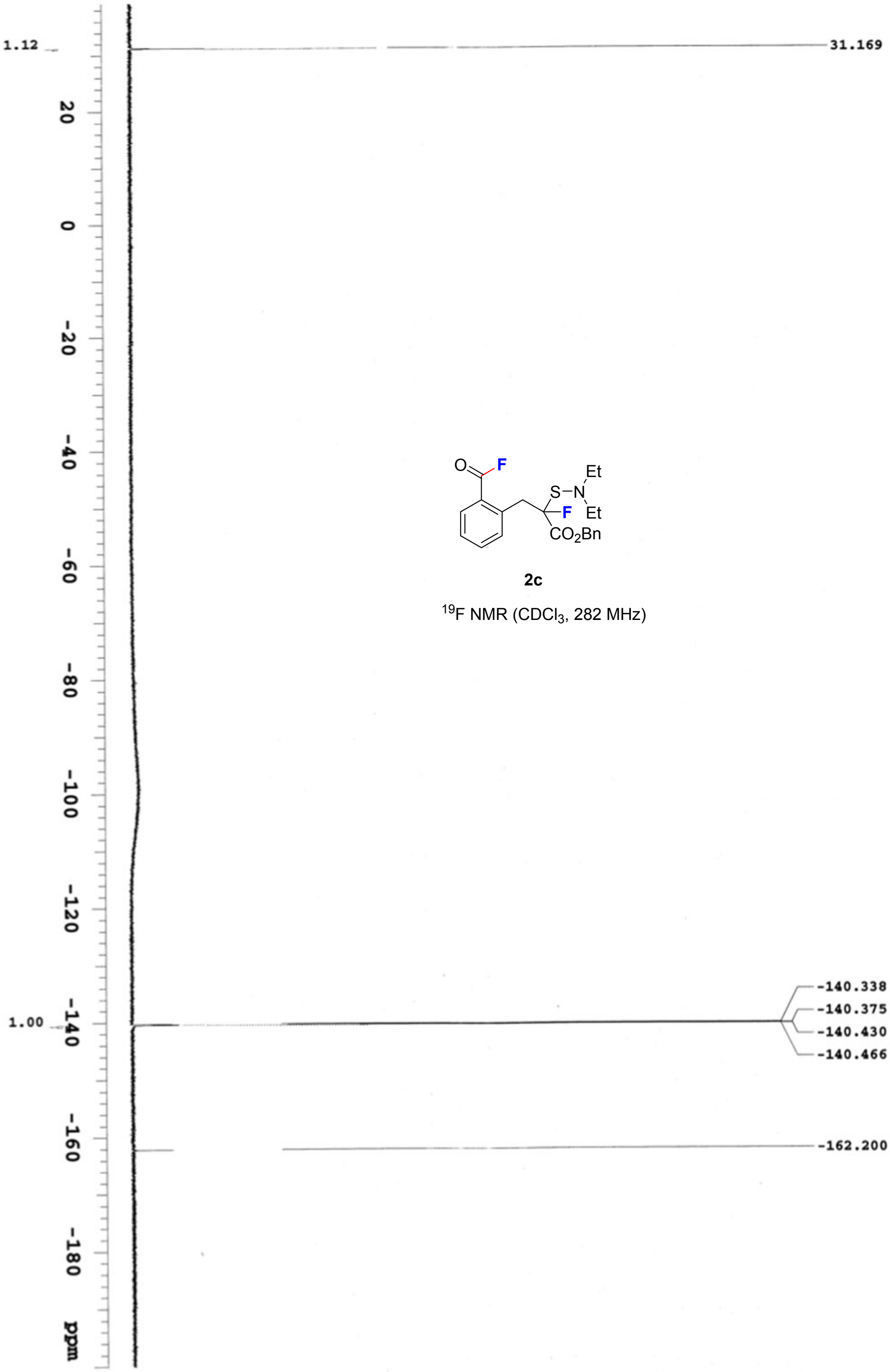

2c

^{19}F NMR ($CDCl_3$, 282 MHz)

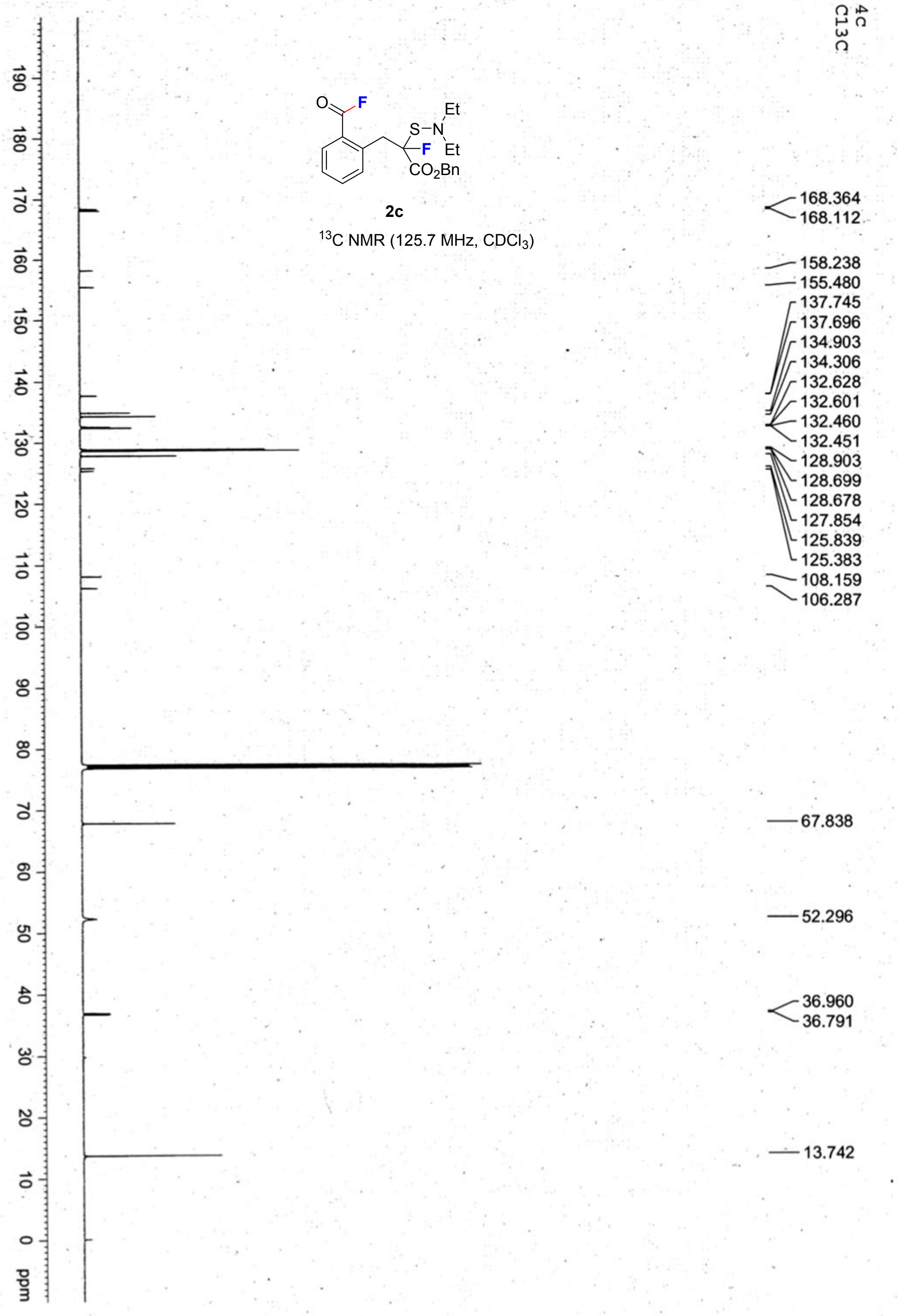

2c

^{13}C NMR (125.7 MHz, $CDCl_3$)

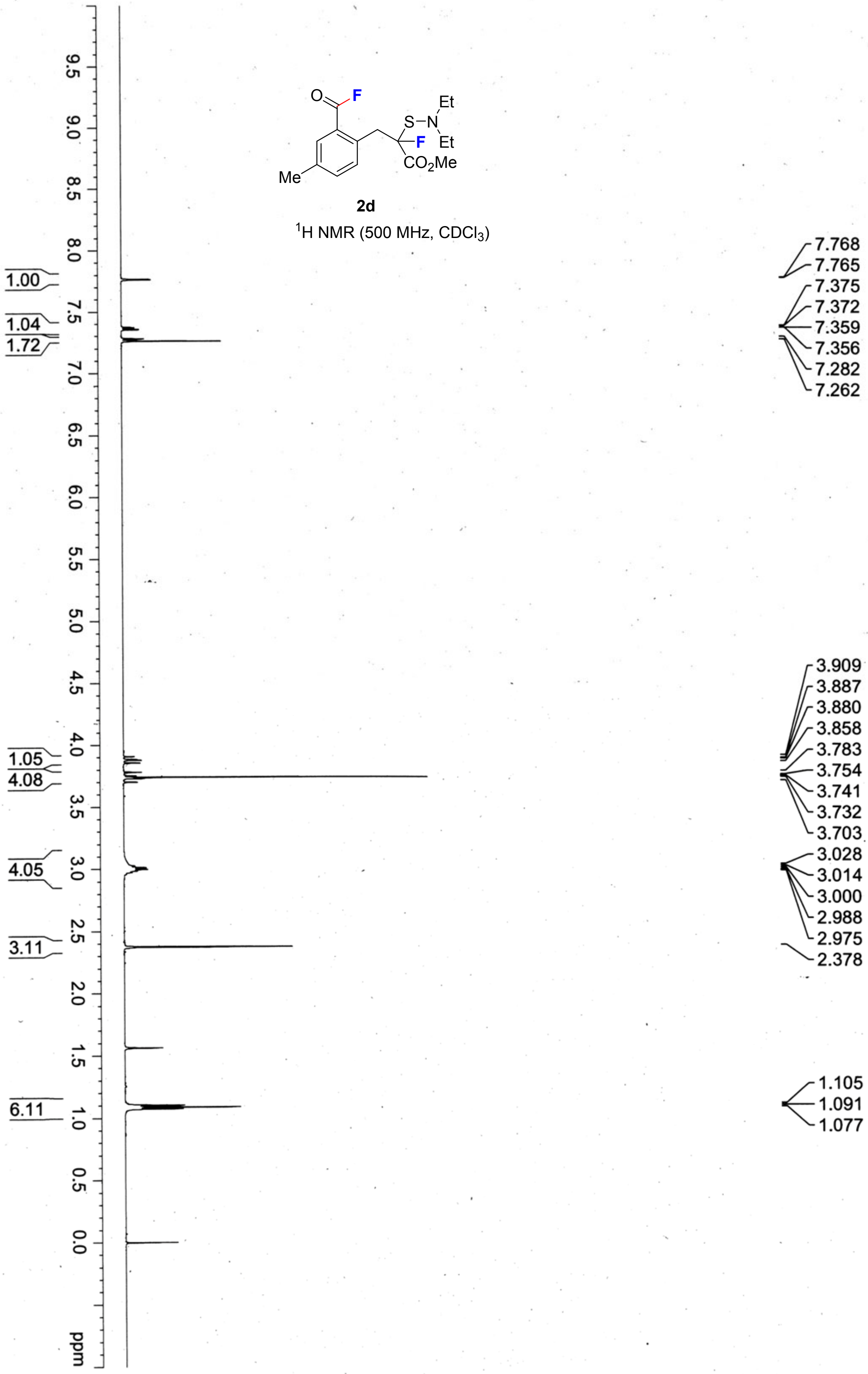
O
F
S
N
Et
Et
F
Me
CO_2Me
2d
^1H NMR (500 MHz, $CDCl_3$)
9.5
9.0
8.5
8.0
7.5
7.0
6.5
6.0
5.5
5.0
4.5
4.0
3.5
3.0
2.5
2.0
1.5
1.0
0.5
0.0
ppm
1.00
1.04
1.72
1.05
4.08
4.05
3.11
6.11
7.768
7.765
7.375
7.372
7.359
7.356
7.282
7.262
3.909
3.887
3.880
3.858
3.783
3.754
3.741
3.732
3.703
3.028
3.014
3.000
2.988
2.975
2.378
1.105
1.091
1.077

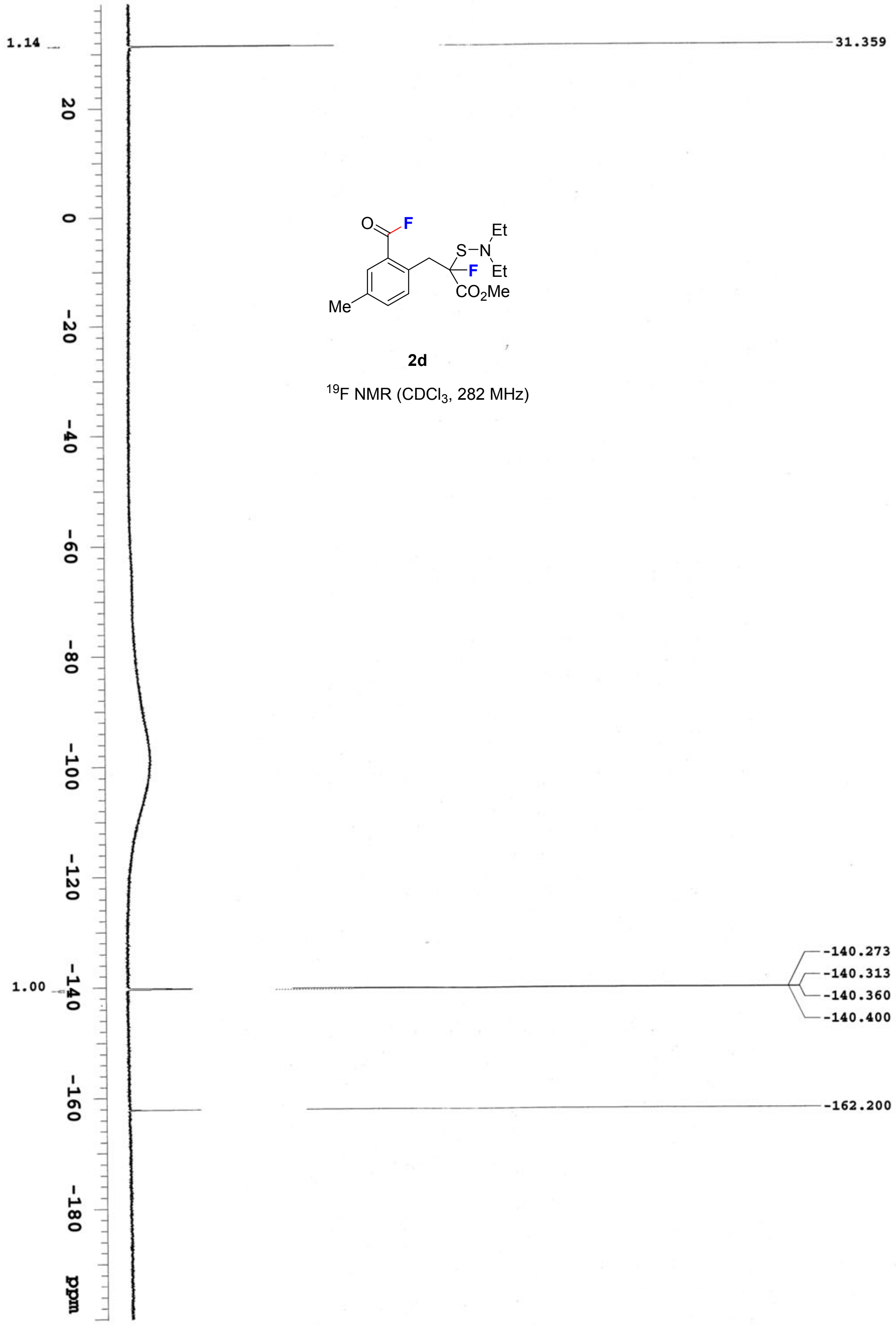

2d

^{19}F NMR ($CDCl_3$, 282 MHz)

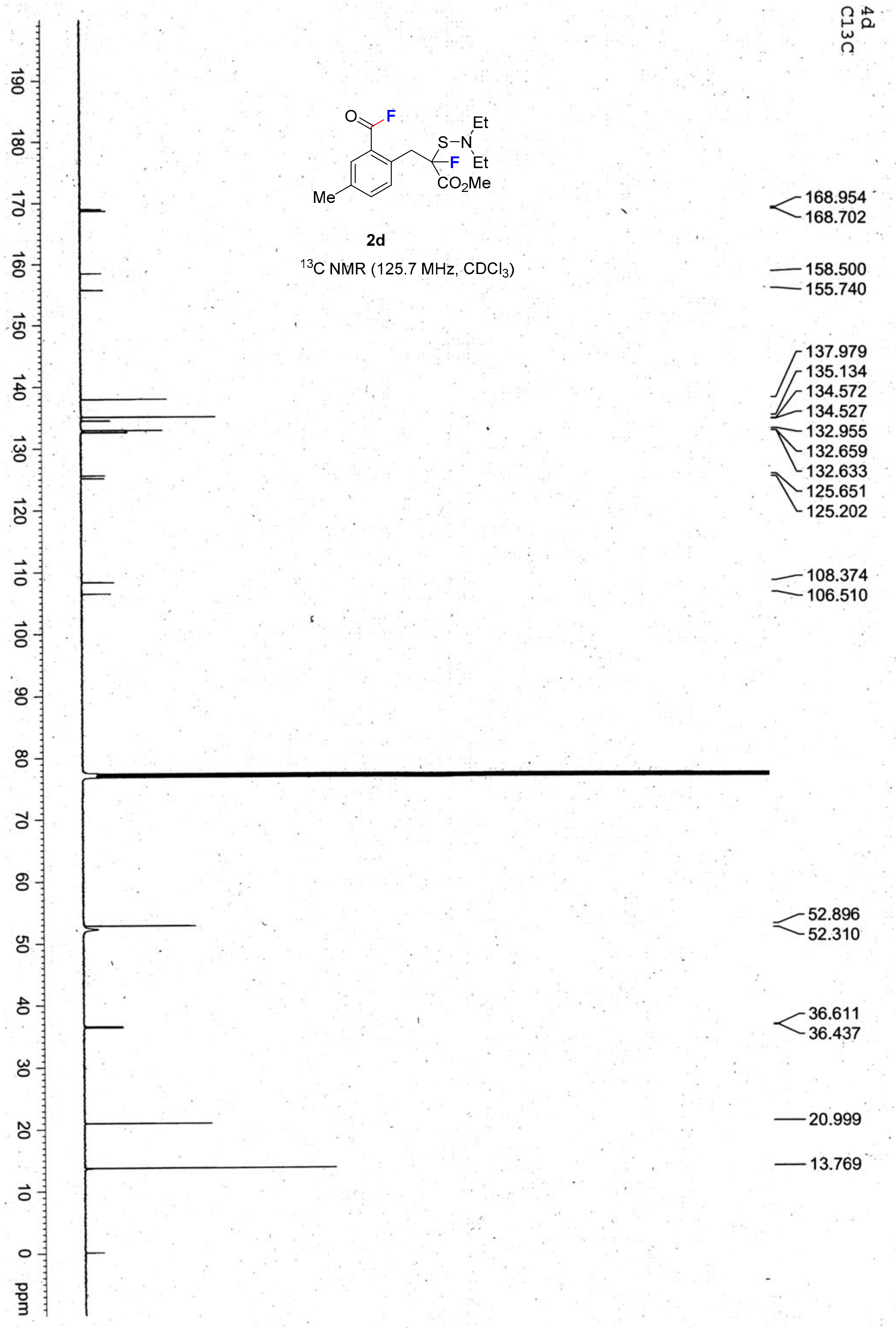

2d

^{13}C NMR (125.7 MHz, $CDCl_3$)

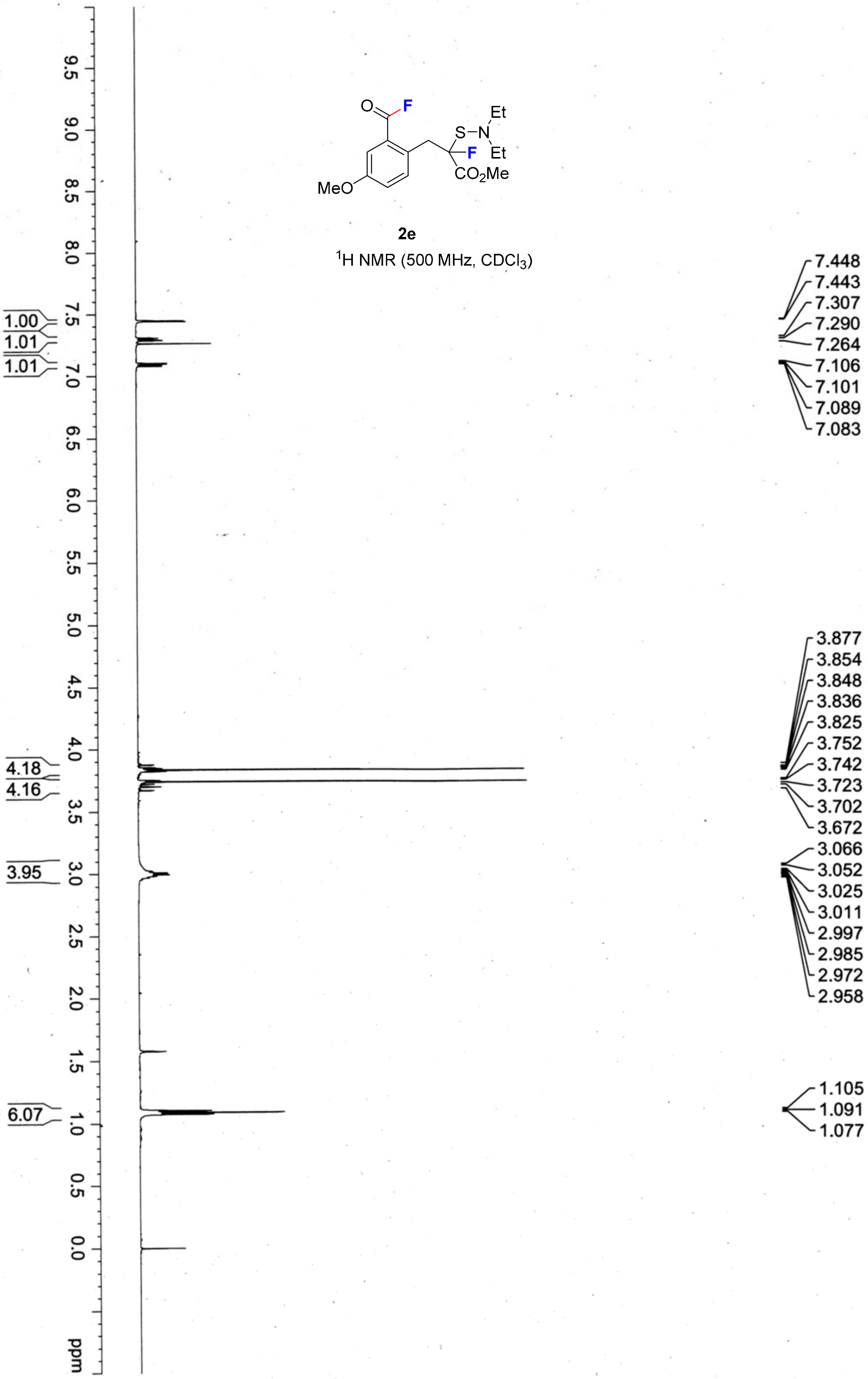

2e

^{1}H NMR (500 MHz, $CDCl_3$)

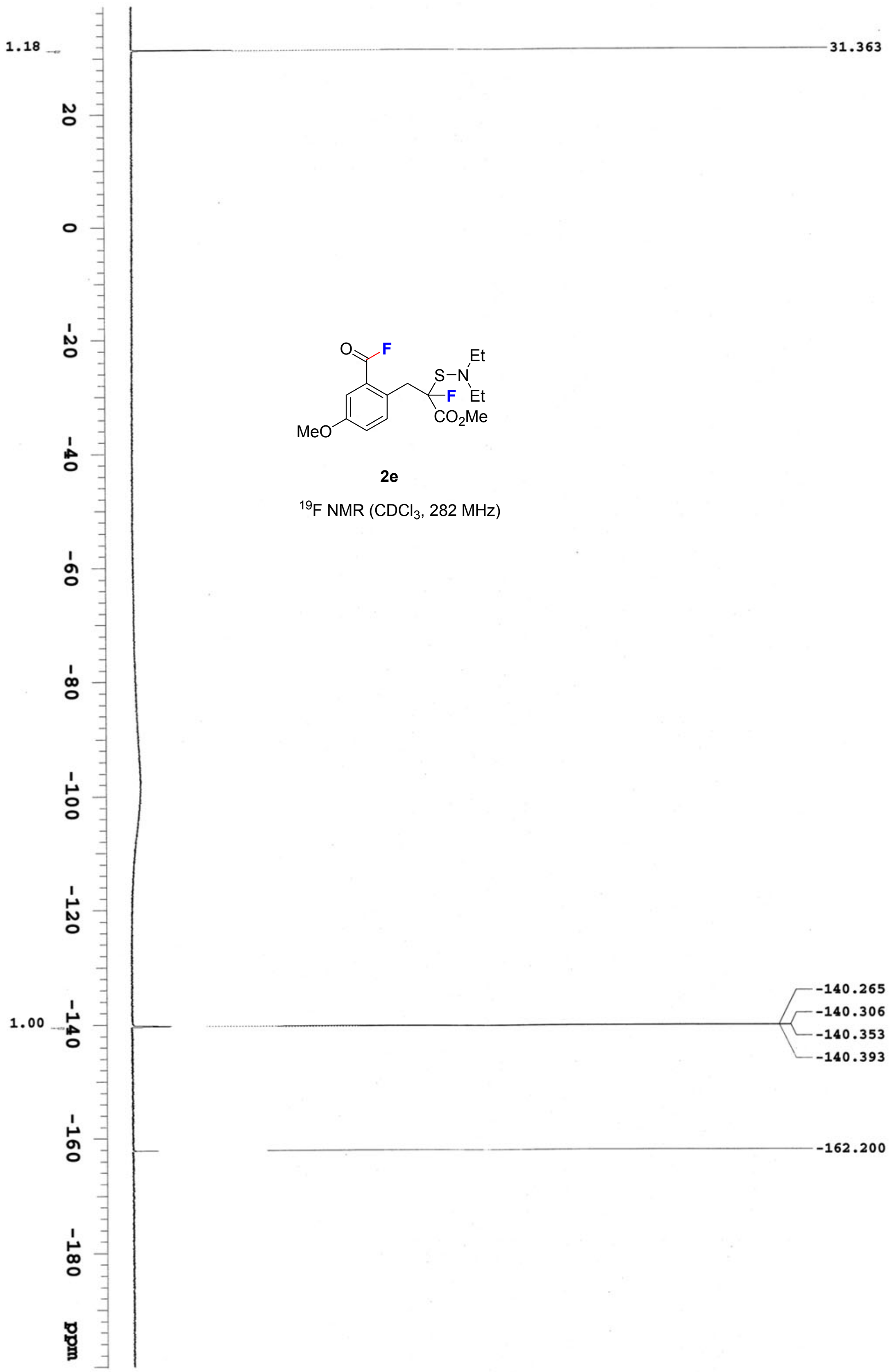

2e

^{19}F NMR ($CDCl_3$, 282 MHz)

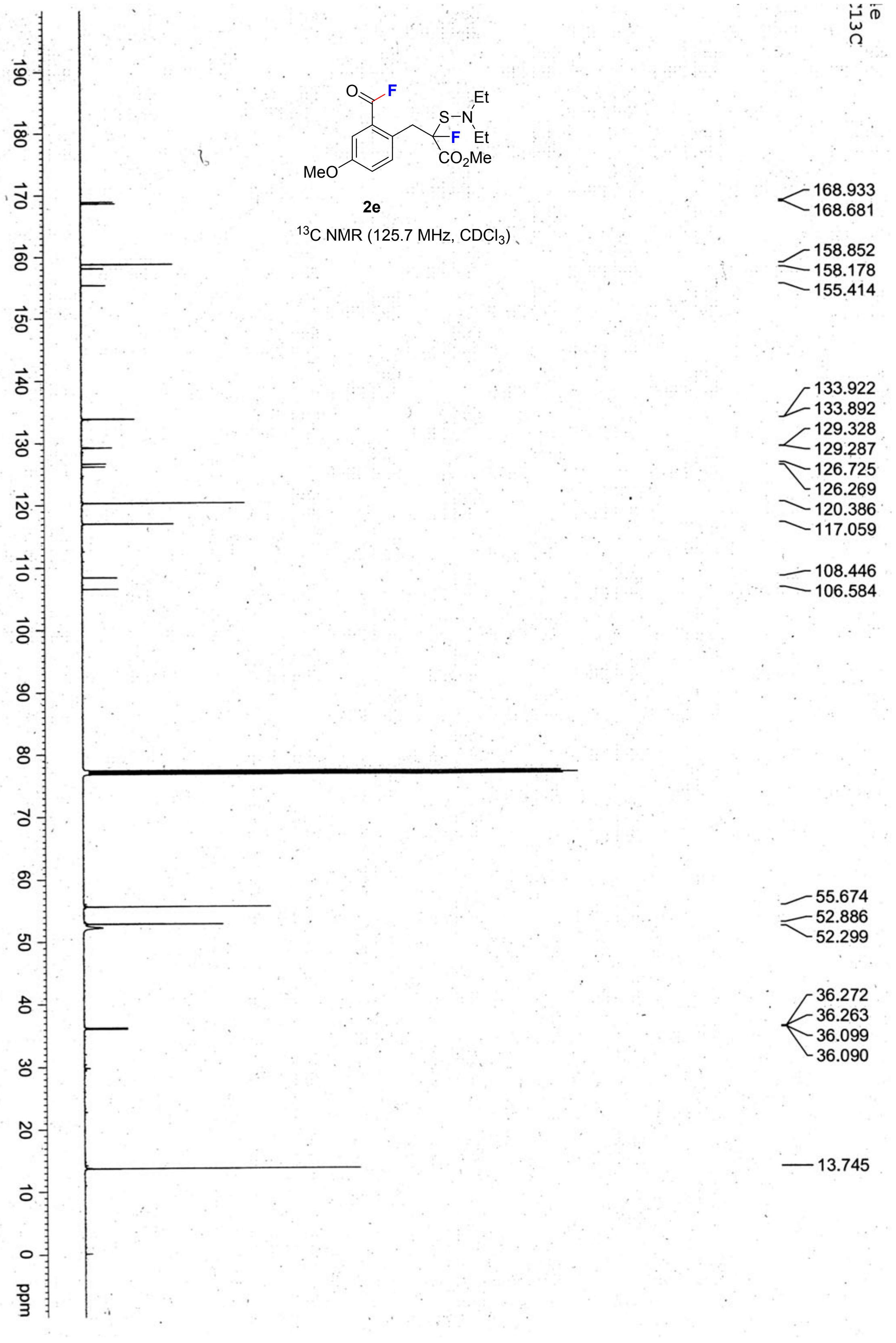

2e
^{13}C NMR (125.7 MHz, CDCl$_3$)
168.933
168.681
158.852
158.178
155.414
133.922
133.892
129.328
129.287
126.725
126.269
120.386
117.059
108.446
106.584
55.674
52.886
52.299
36.272
36.263
36.099
36.090
13.745
ppm

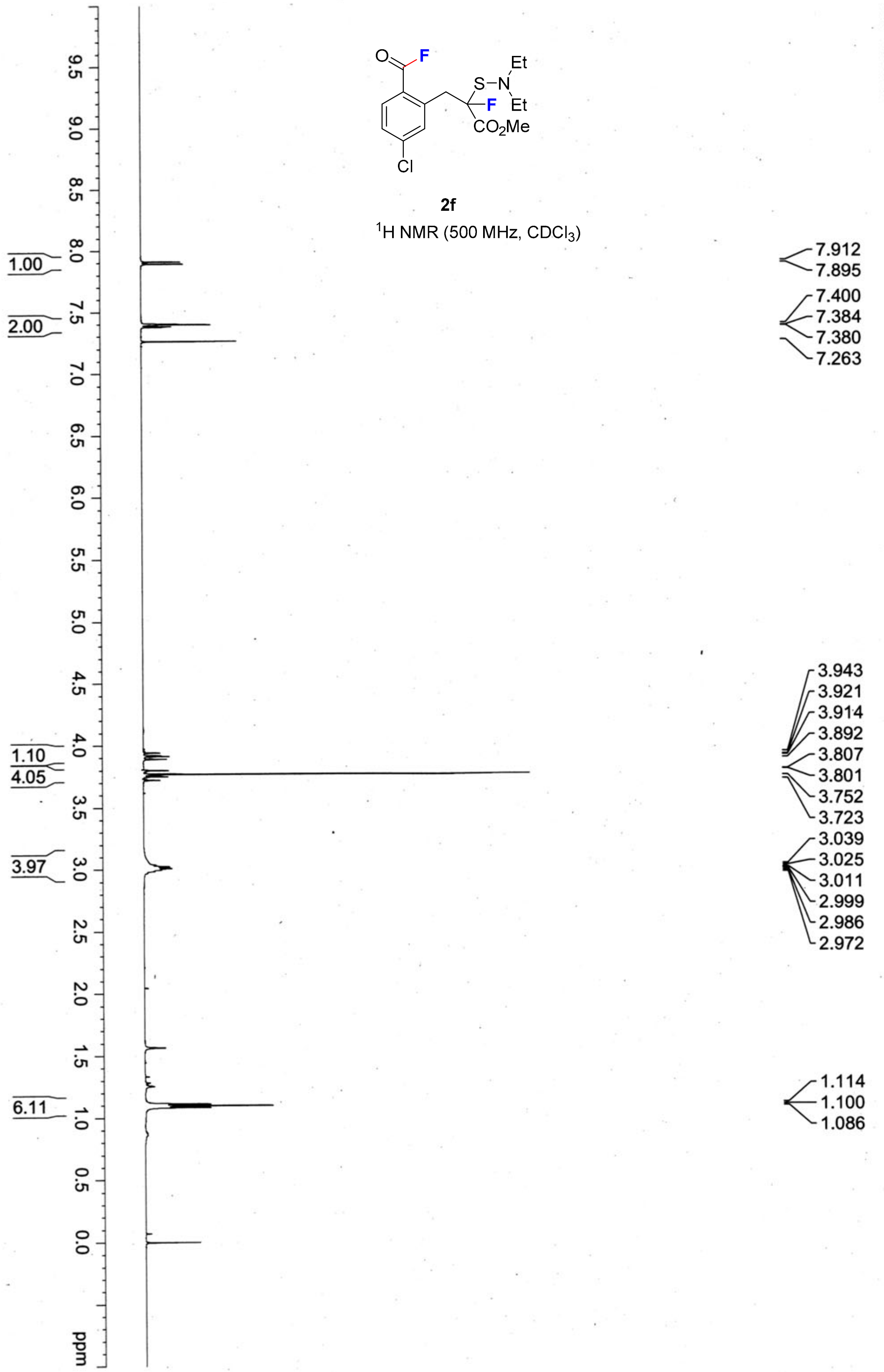

2f

^{1}H NMR (500 MHz, CDCl$_3$)

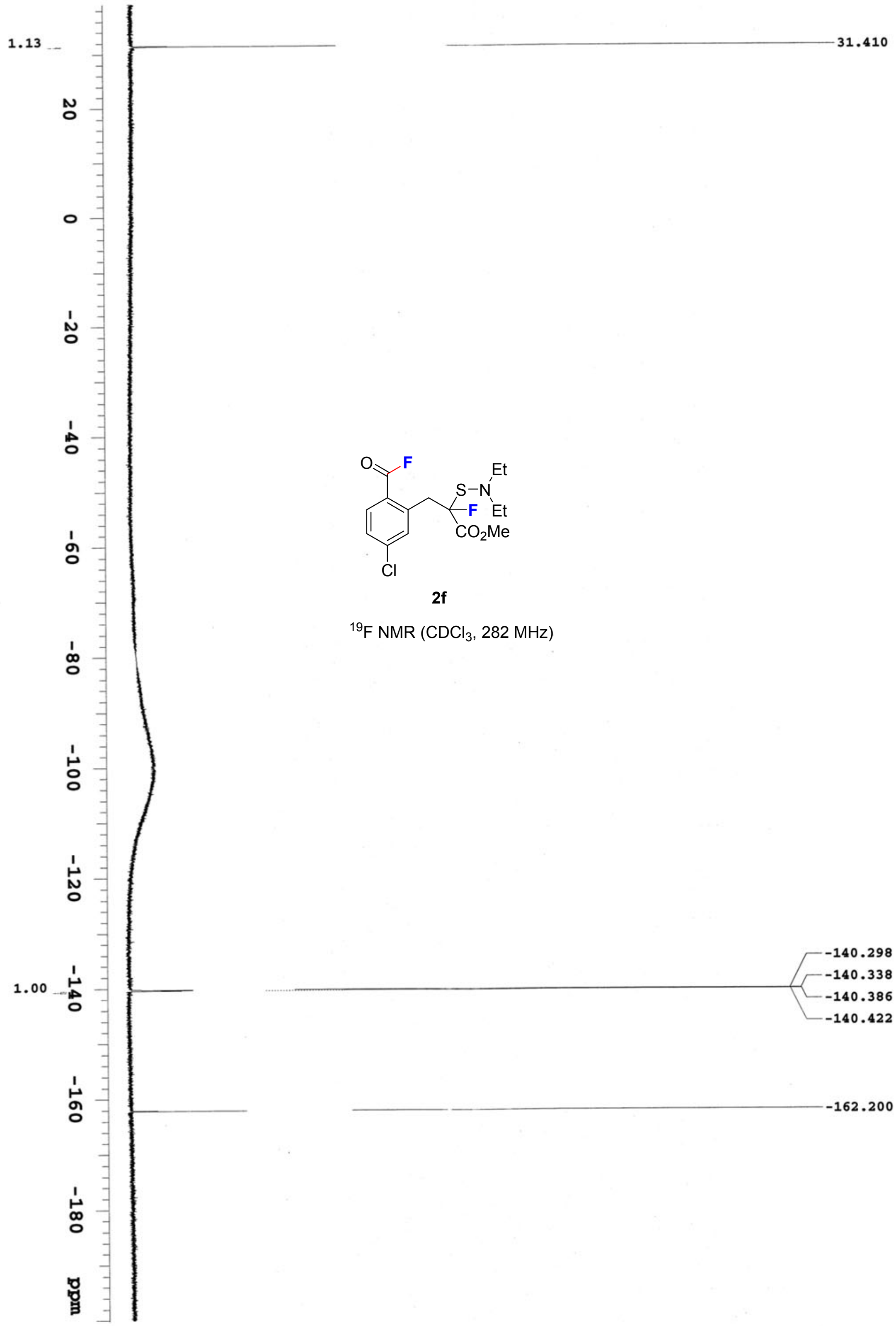

2f

^{19}F NMR ($CDCl_3$, 282 MHz)

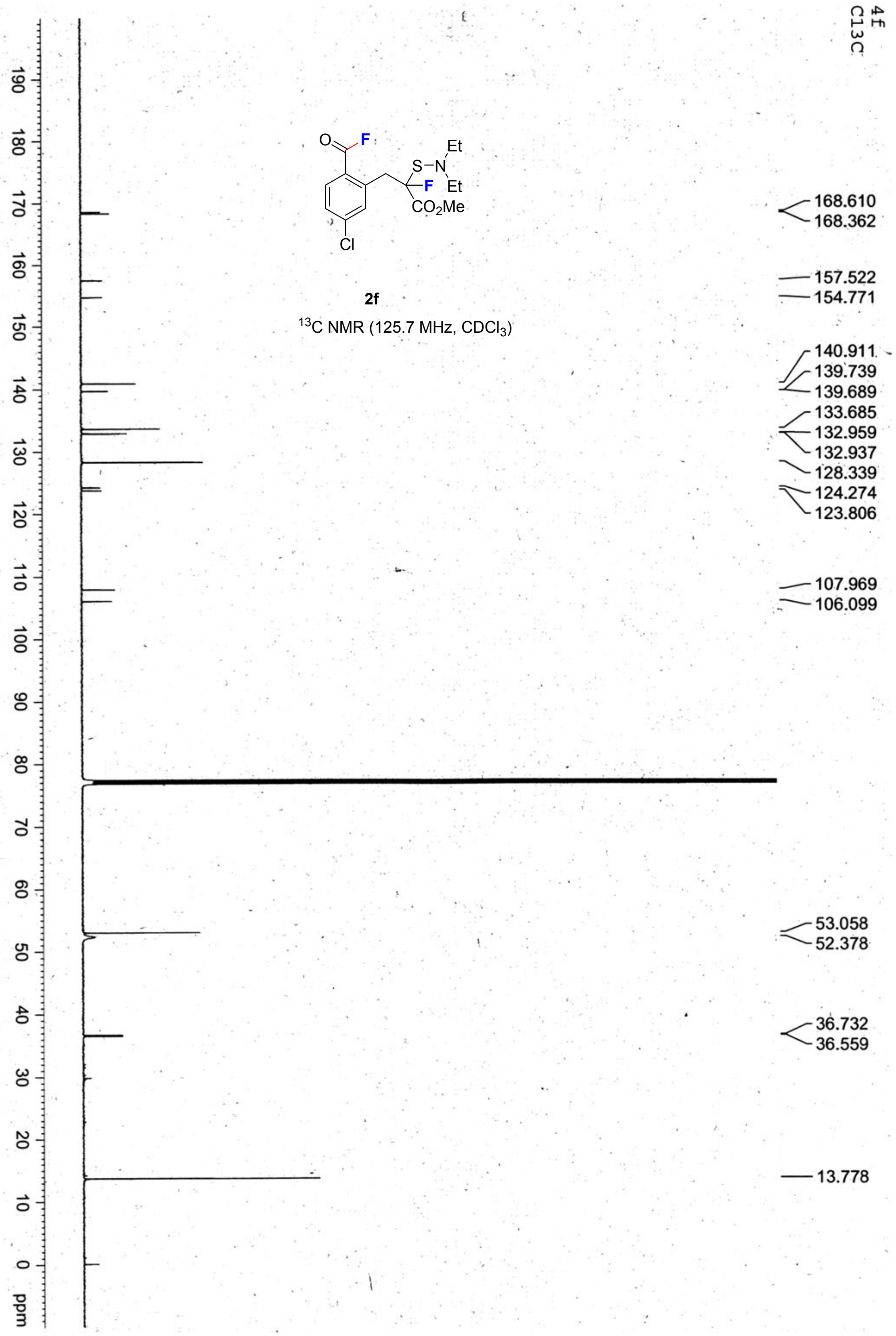

2f

^{13}C NMR (125.7 MHz, CDCl$_3$)

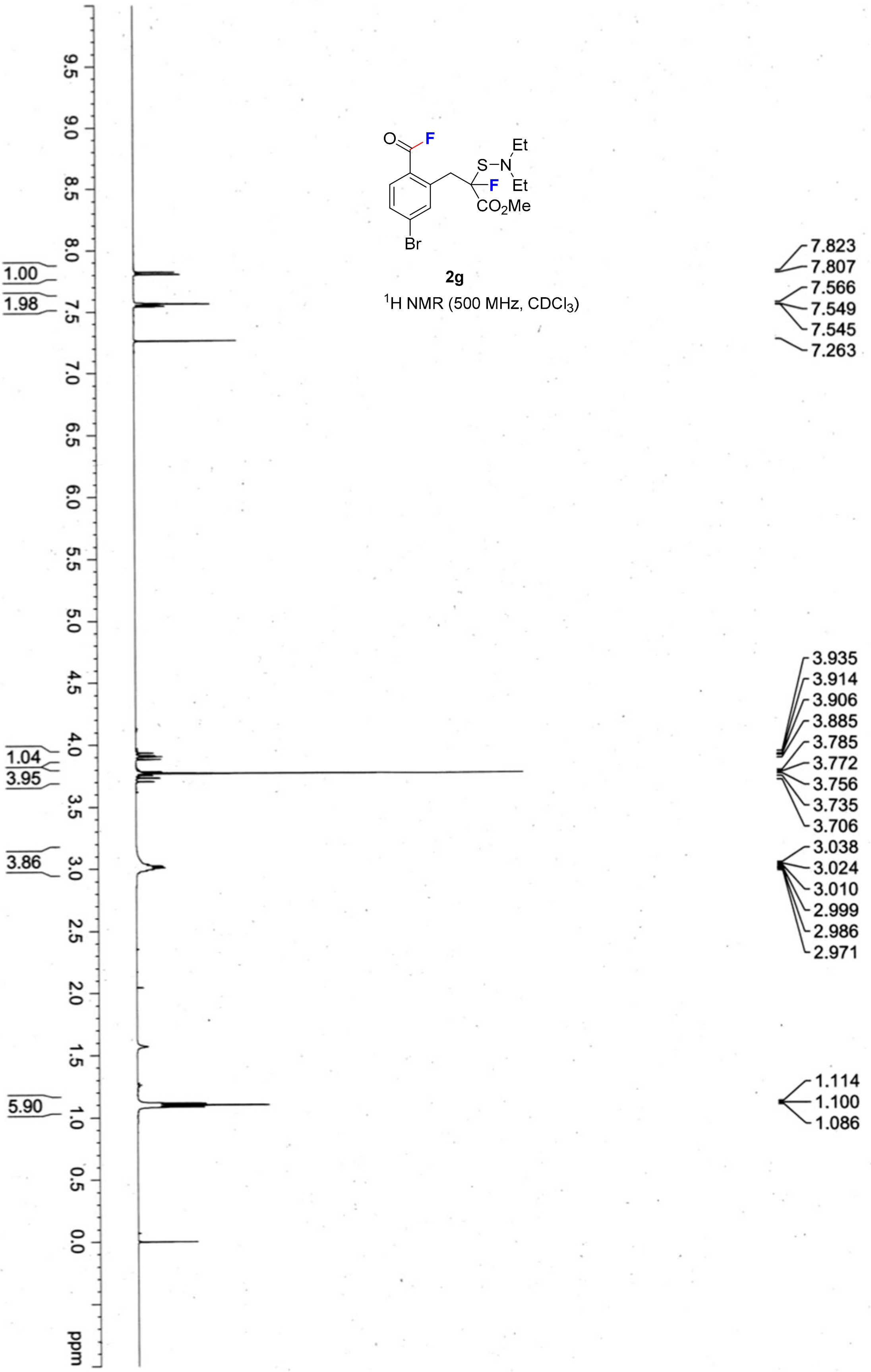

2g

^{1}H NMR (500 MHz, $CDCl_3$)

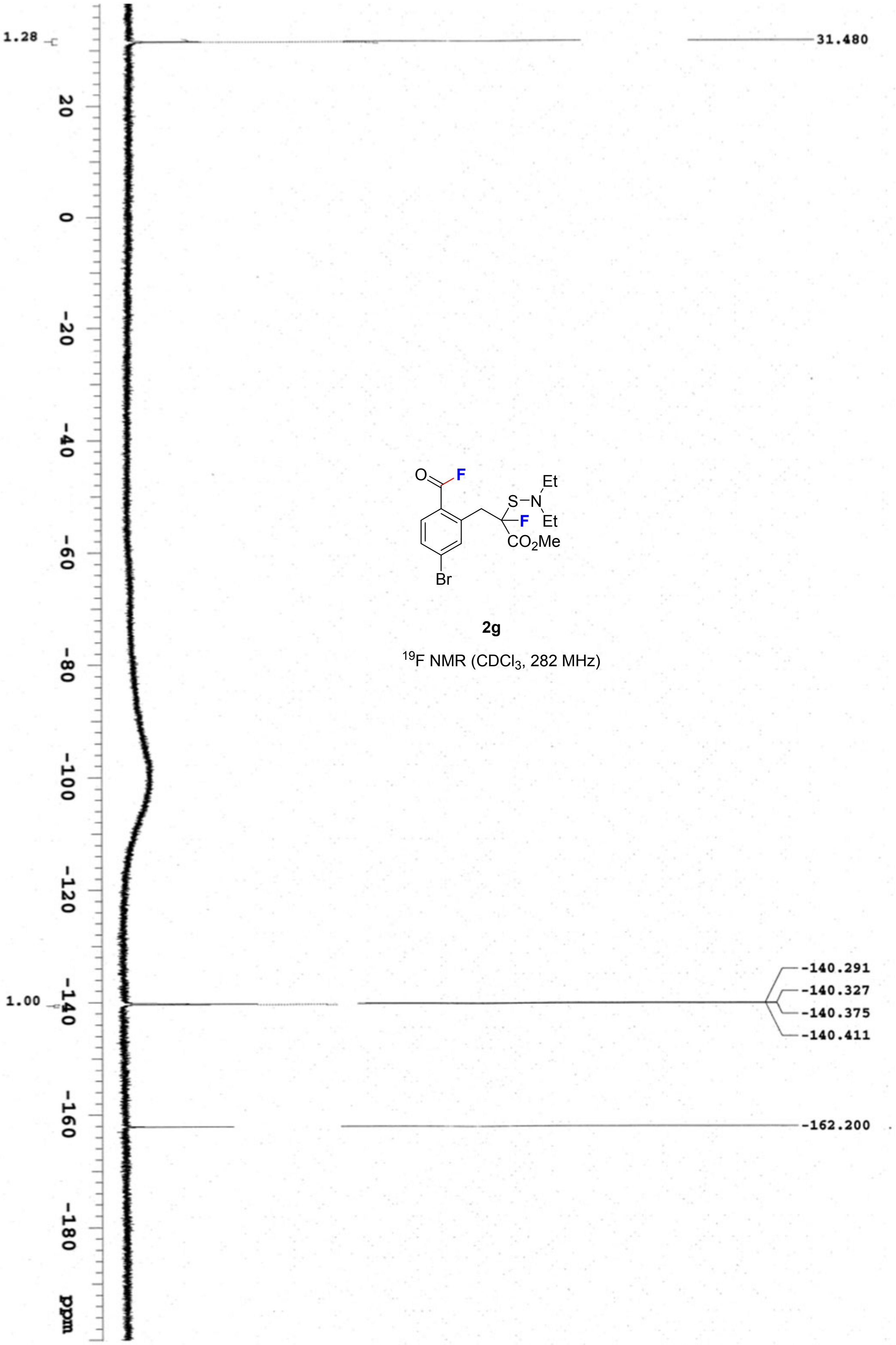

2g

^{19}F NMR ($CDCl_3$, 282 MHz)

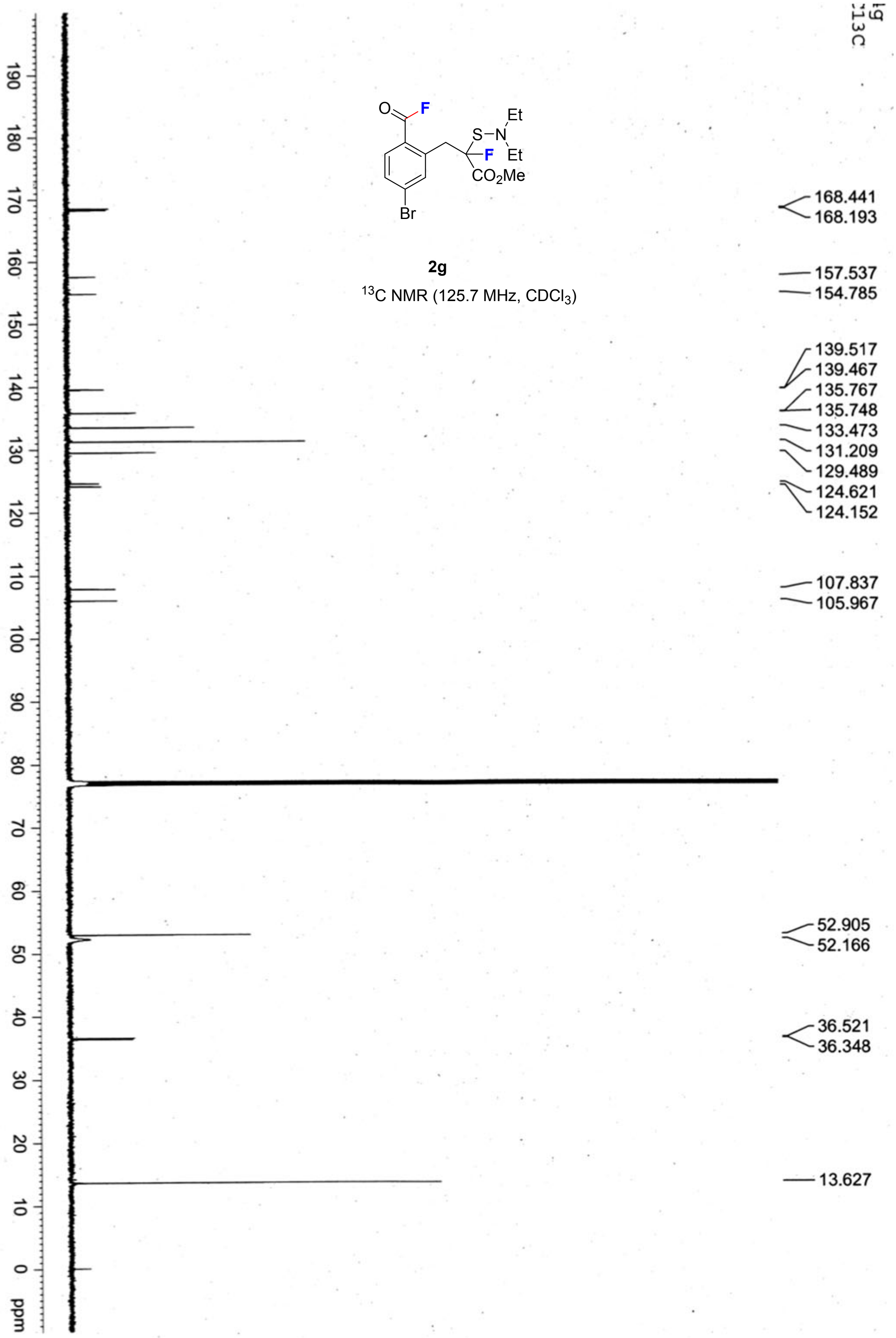

2g

^{13}C NMR (125.7 MHz, $CDCl_3$)

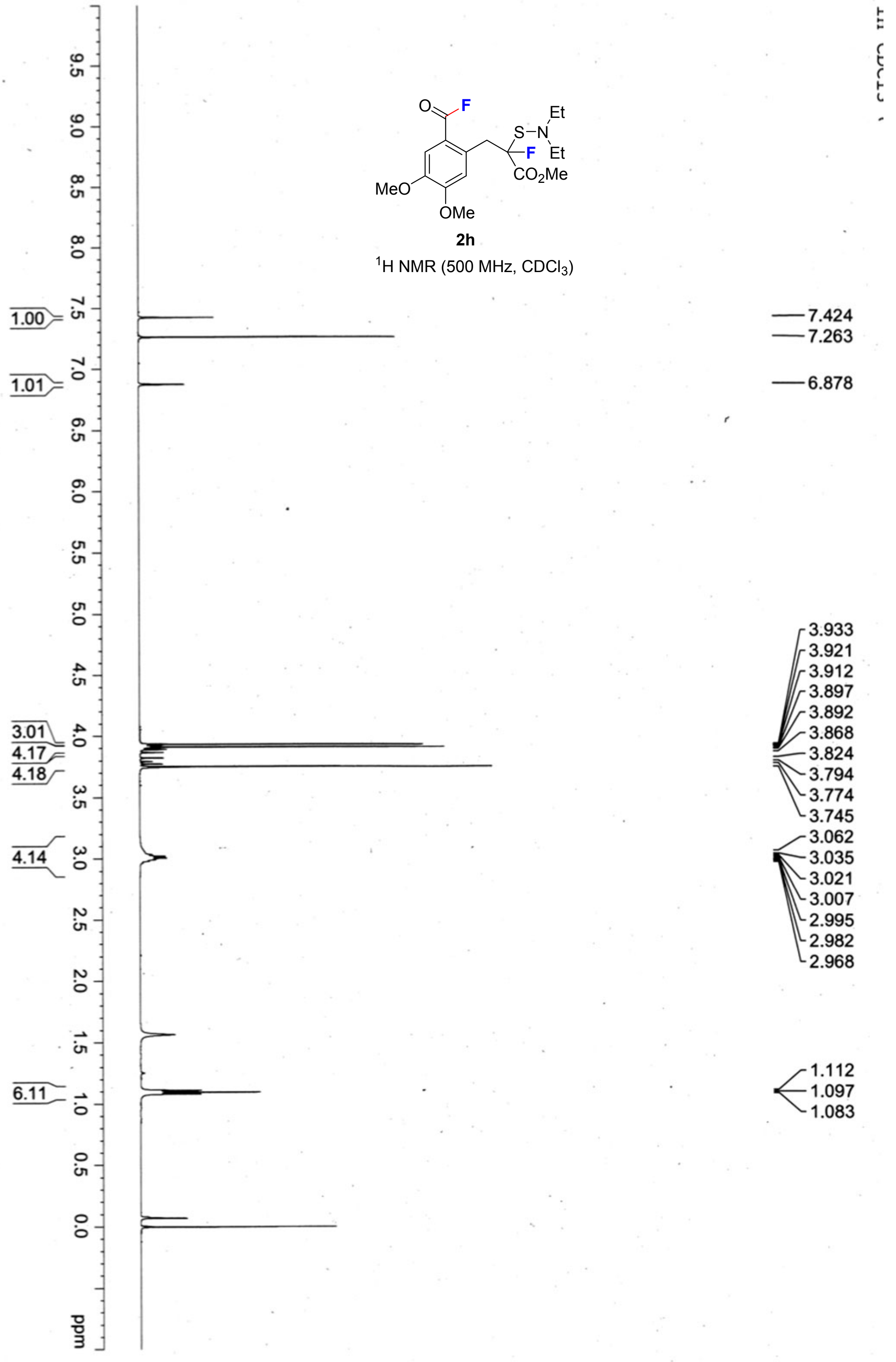

2h

^{1}H NMR (500 MHz, $CDCl_3$)

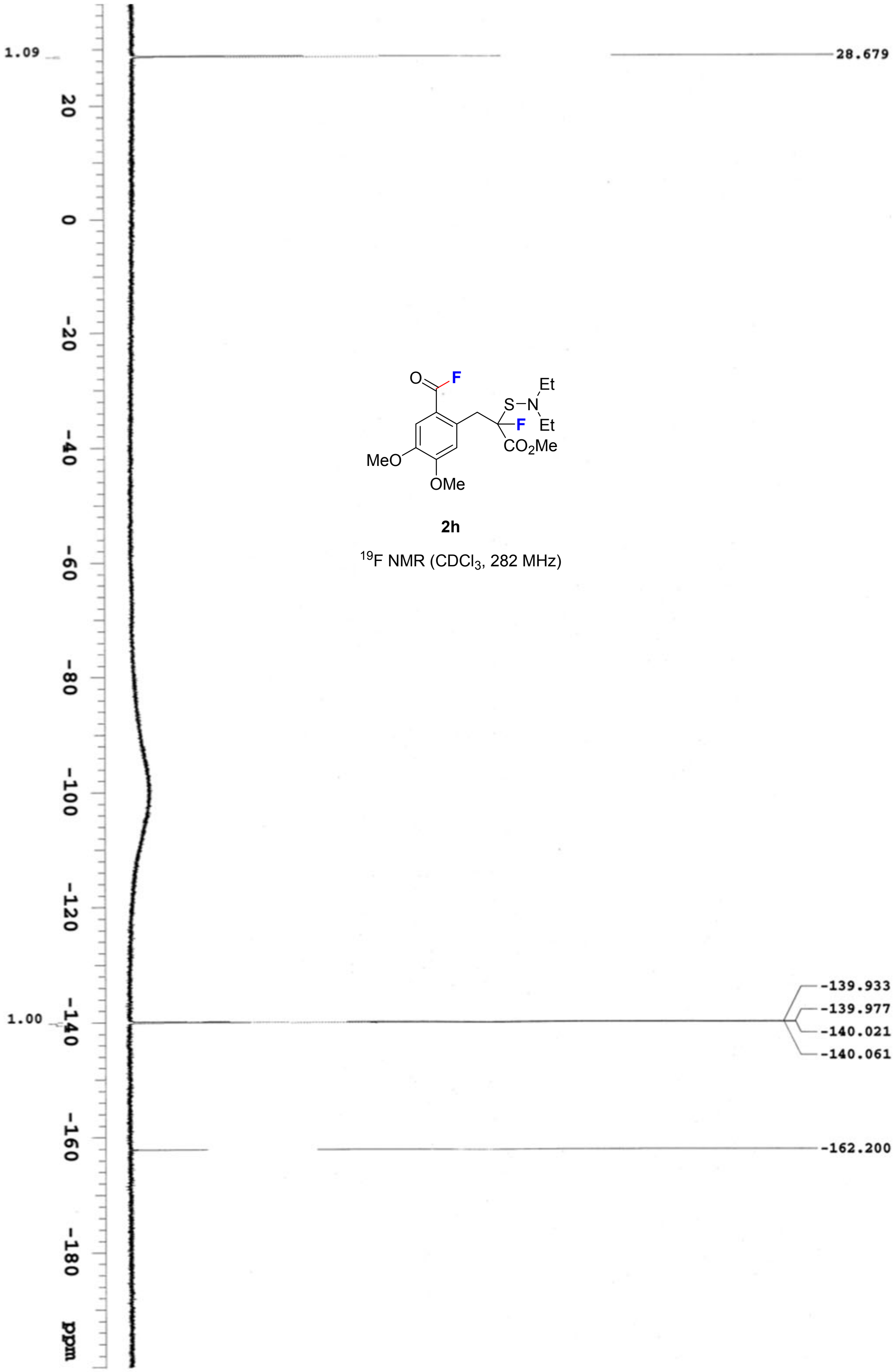

2h

^{19}F NMR ($CDCl_3$, 282 MHz)

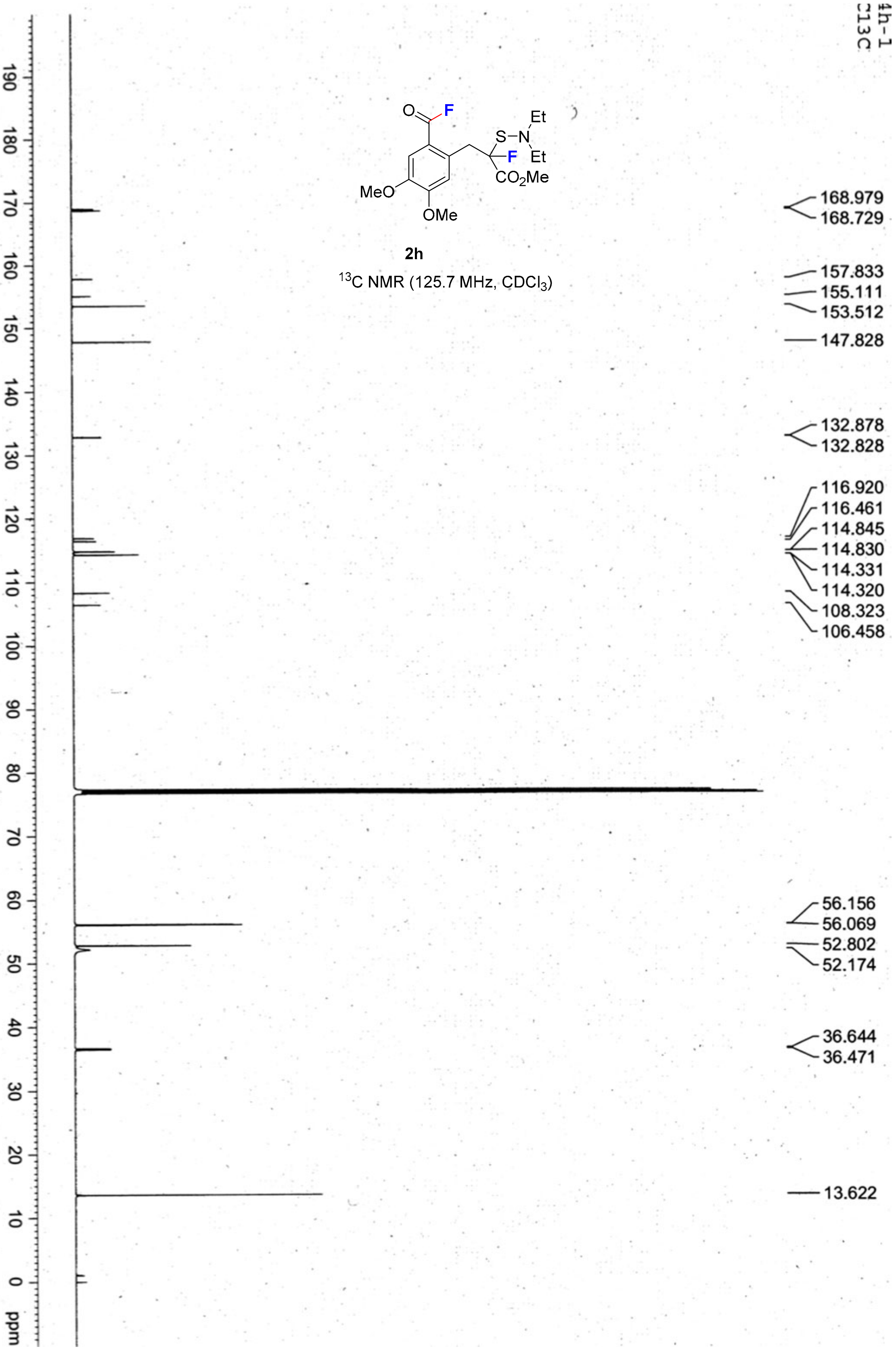

2h

^{13}C NMR (125.7 MHz, $CDCl_3$)

2i

^{1}H NMR (500 MHz, $CDCl_3$)

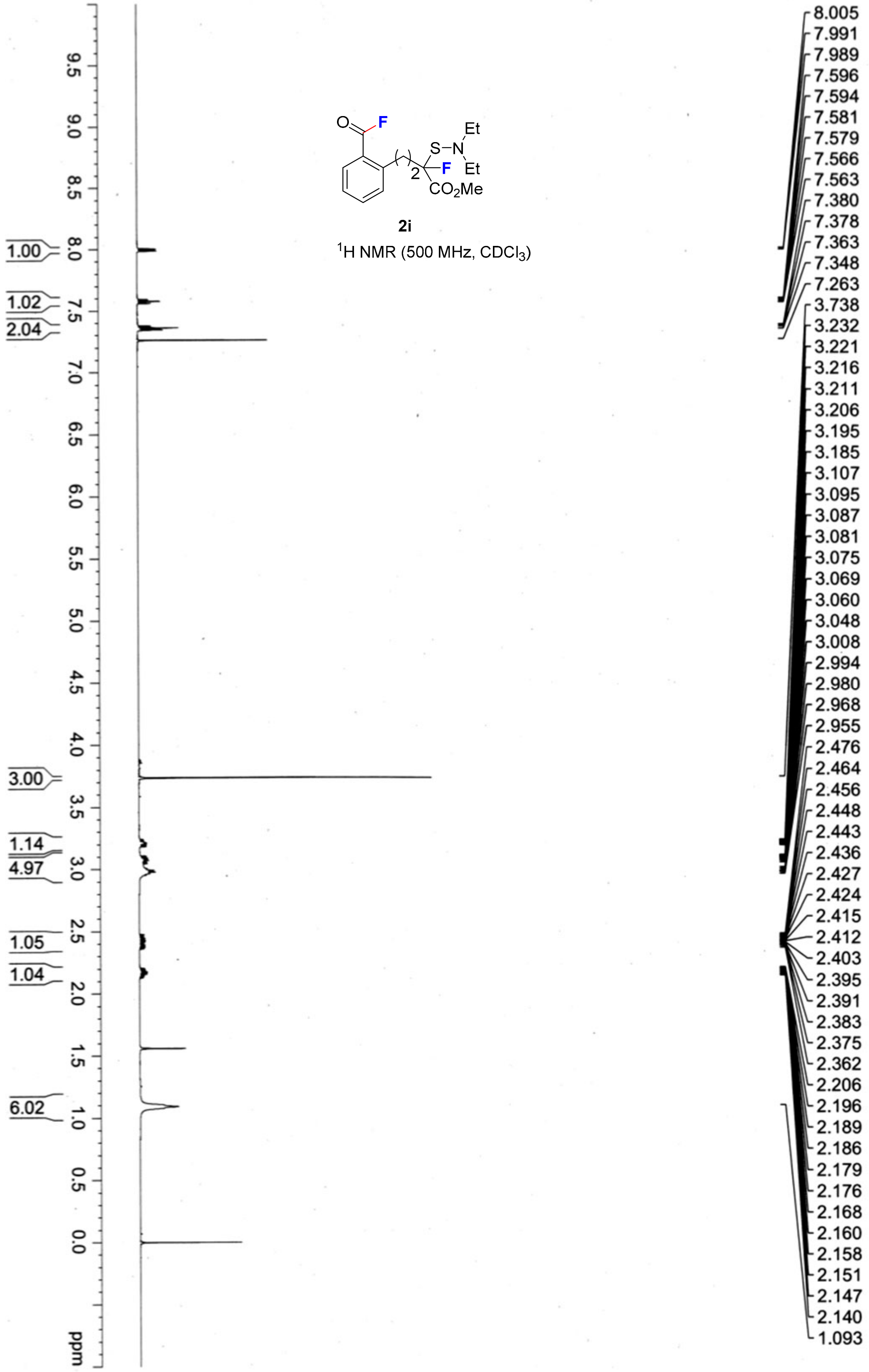

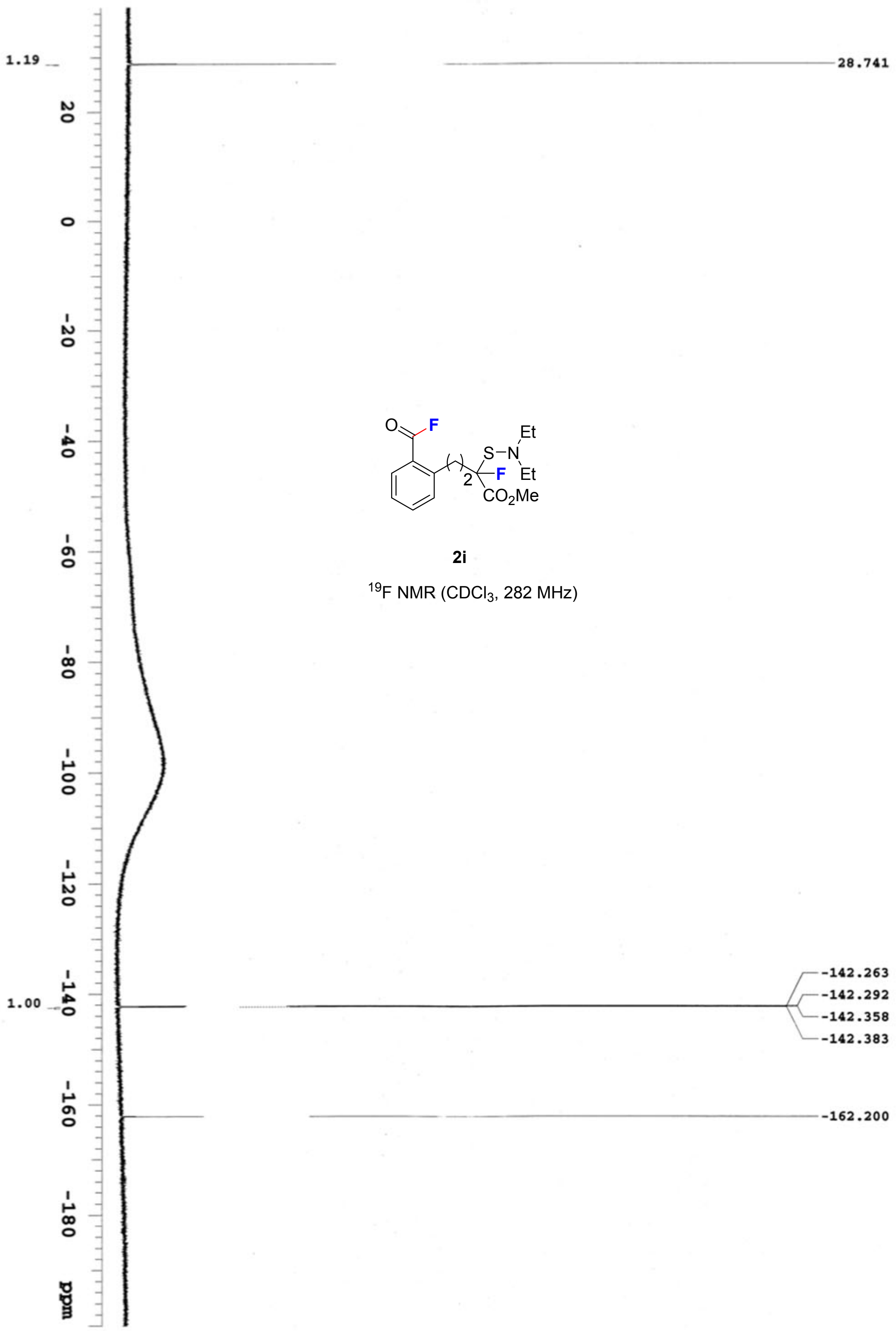

2i

^{19}F NMR ($CDCl_3$, 282 MHz)

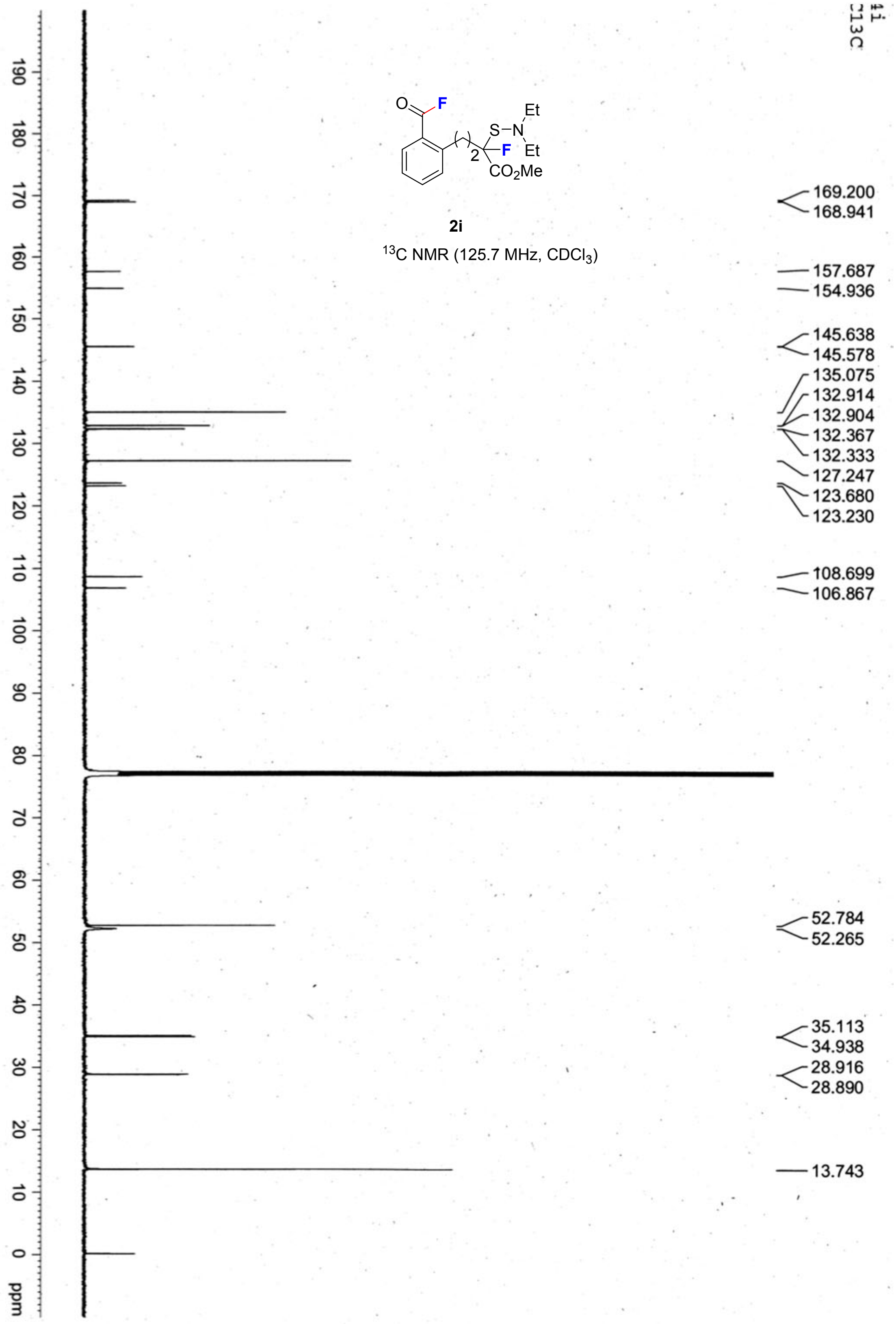

2i

^{13}C NMR (125.7 MHz, $CDCl_3$)

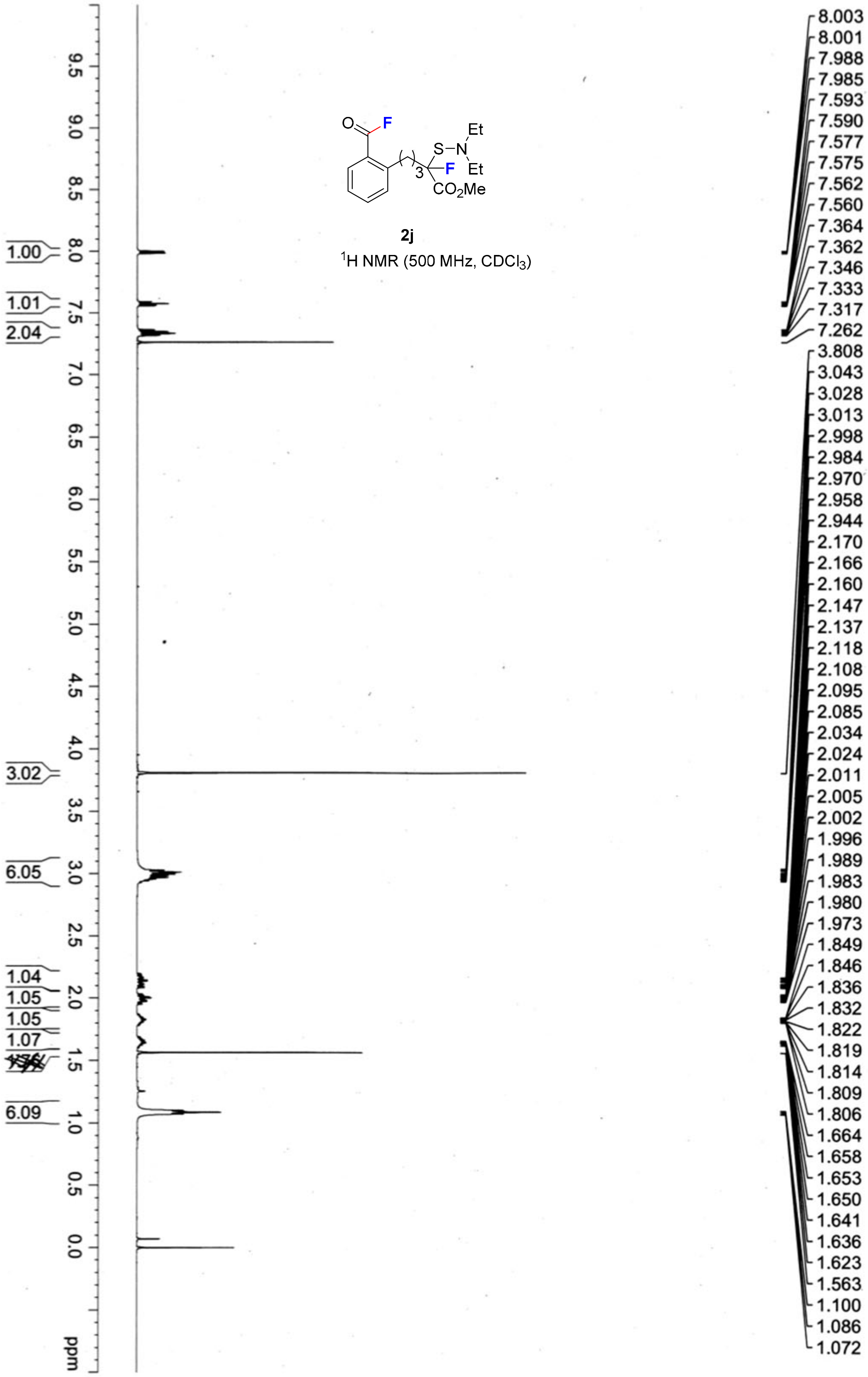

2j

^{1}H NMR (500 MHz, $CDCl_3$)

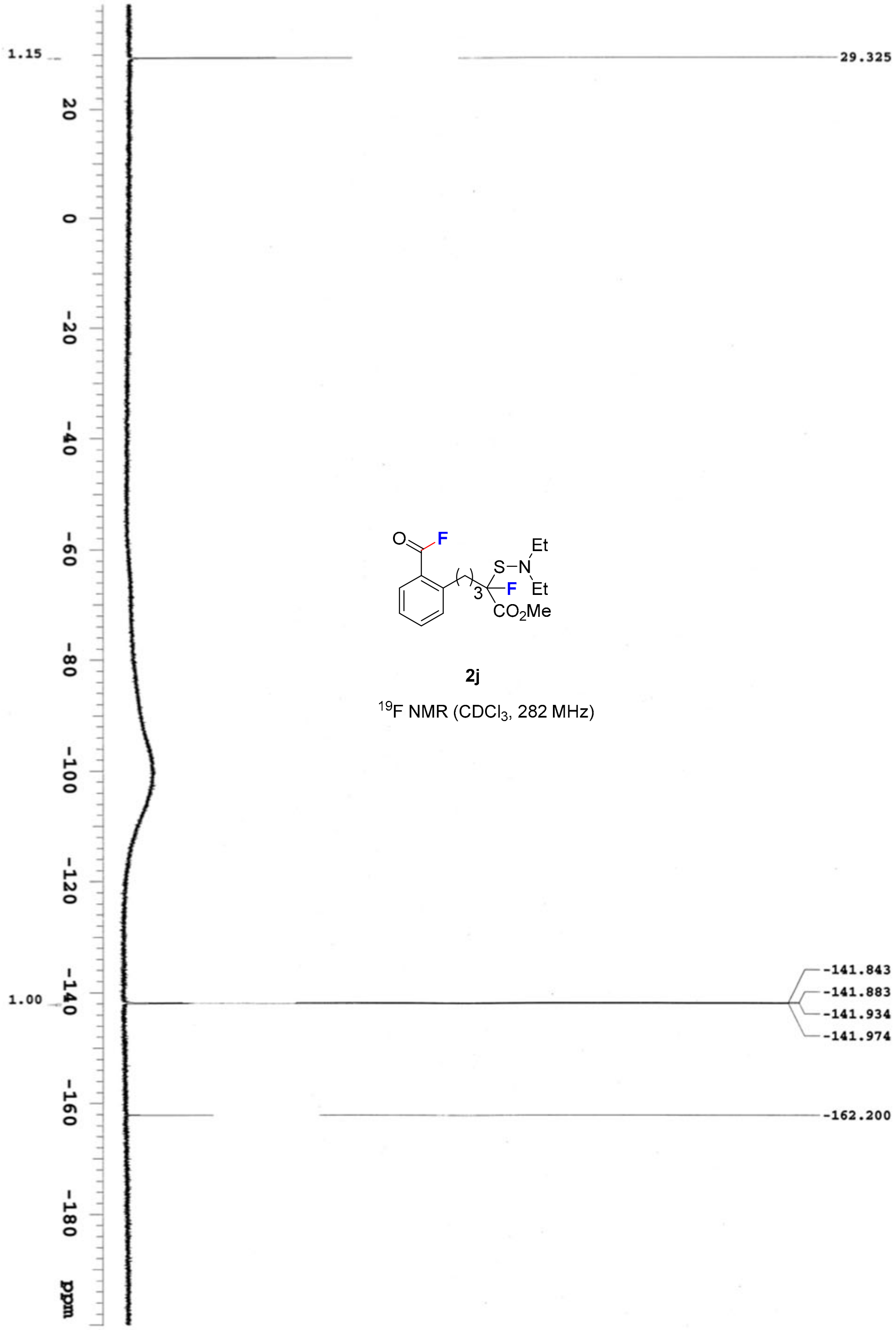

2j

^{19}F NMR ($CDCl_3$, 282 MHz)

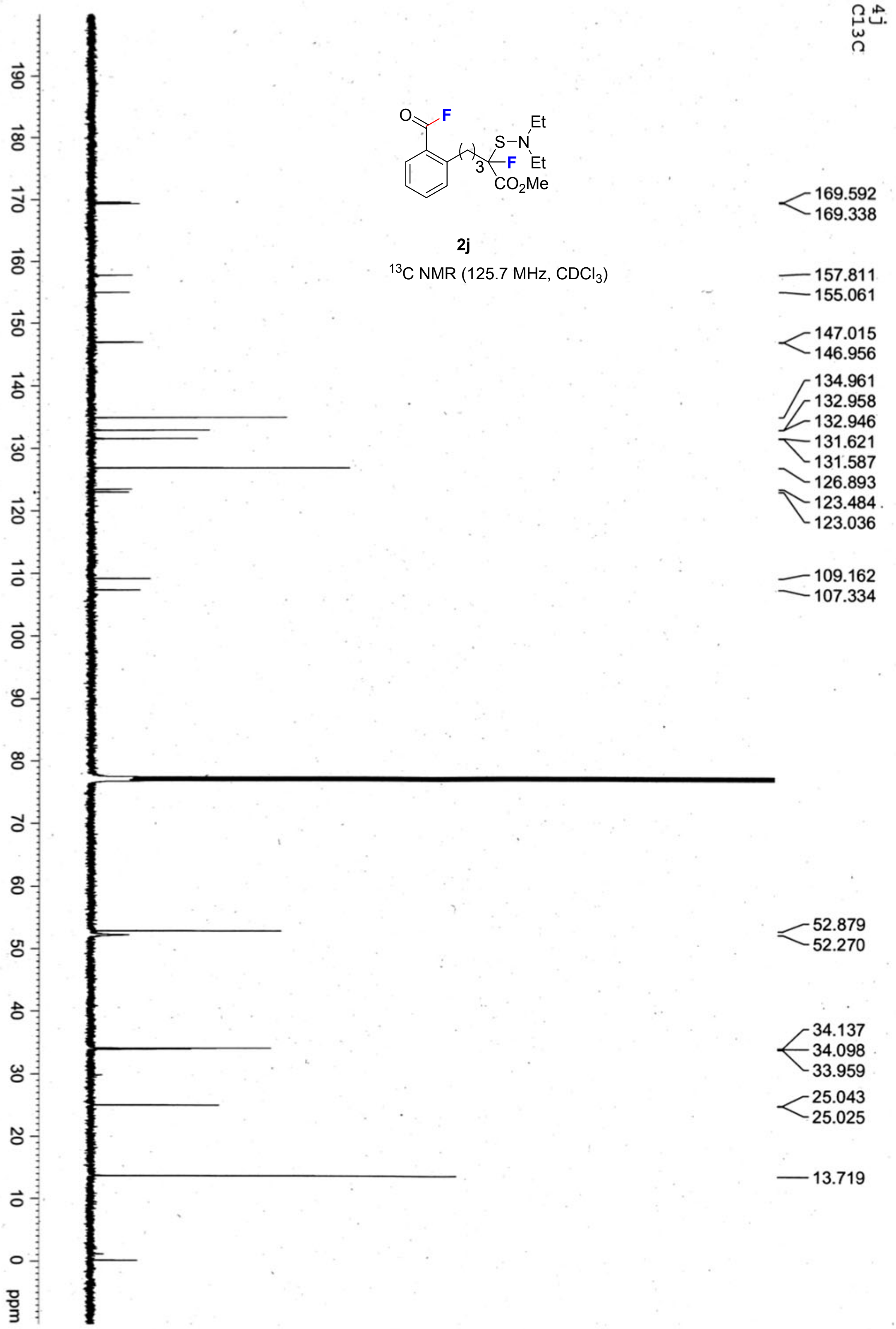

2j

^{13}C NMR (125.7 MHz, $CDCl_3$)

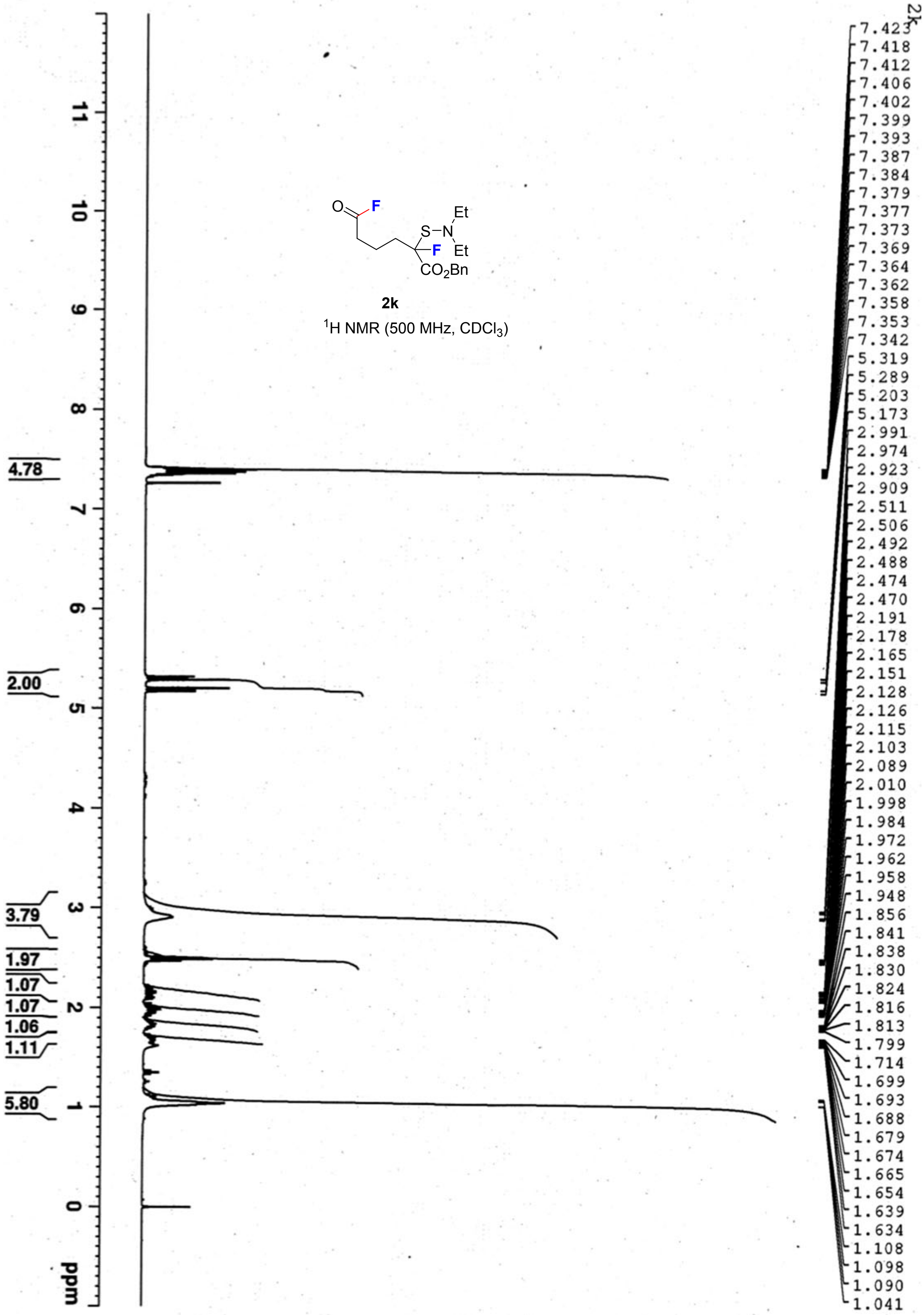

2k
^1H NMR (500 MHz, $CDCl_3$)

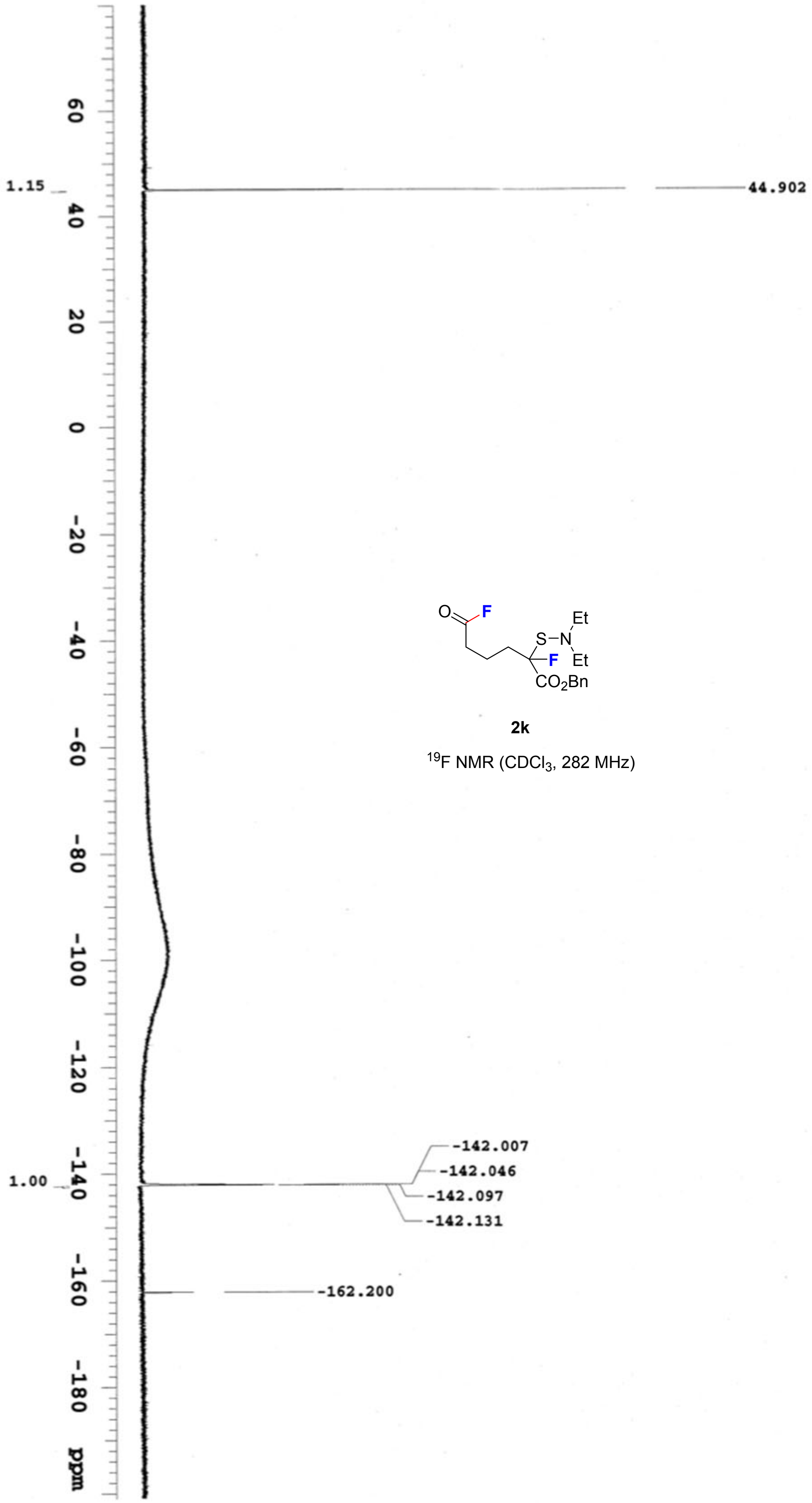

2k

^{19}F NMR ($CDCl_3$, 282 MHz)

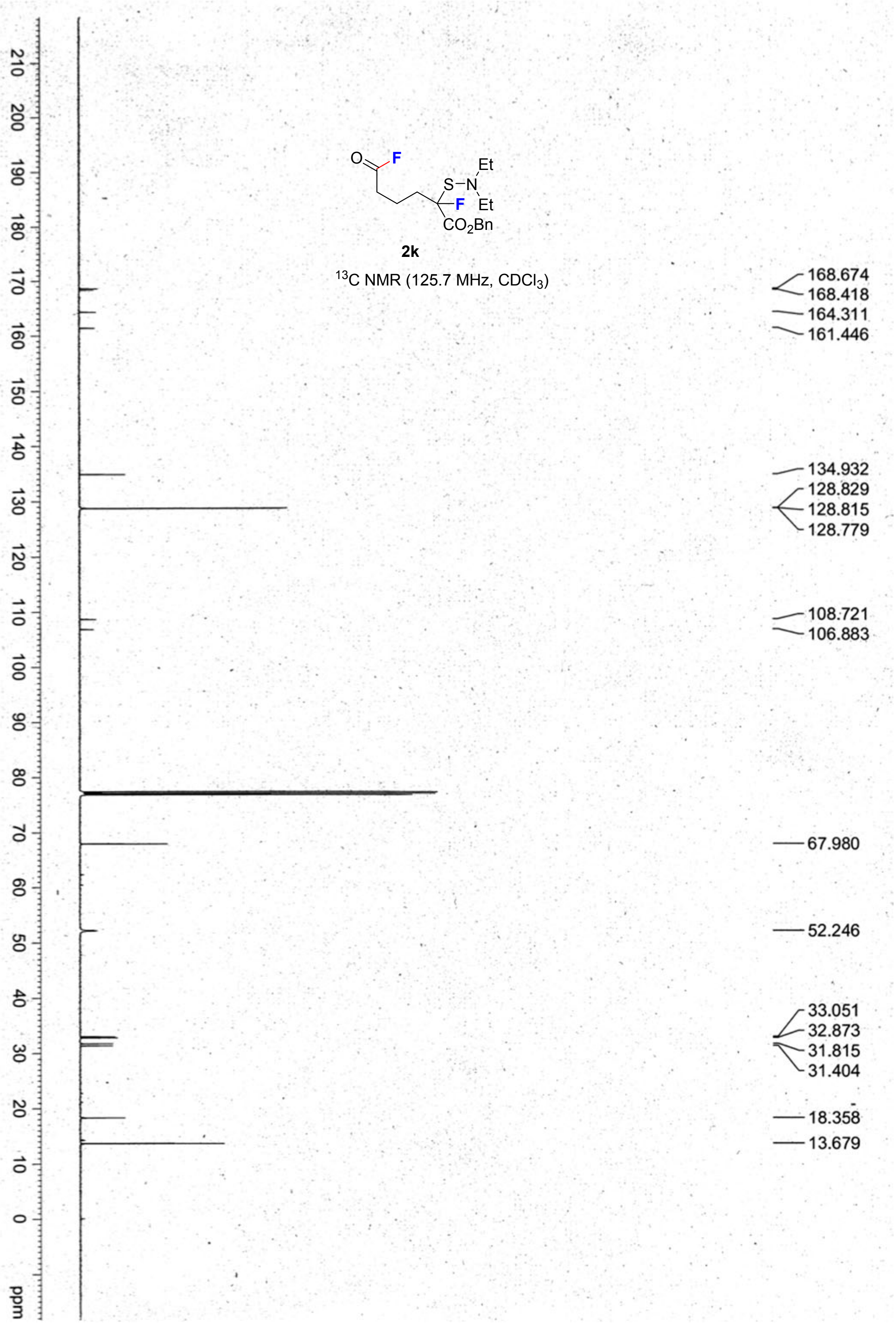

Et
S–N
F
Et
CO_2Bn
2k
^{13}C NMR (125.7 MHz, $CDCl_3$)
168.674
168.418
164.311
161.446
134.932
128.829
128.815
128.779
108.721
106.883
67.980
52.246
33.051
32.873
31.815
31.404
18.358
13.679
210
200
190
180
170
160
150
140
130
120
110
100
90
80
70
60
50
40
30
20
10
0
ppm

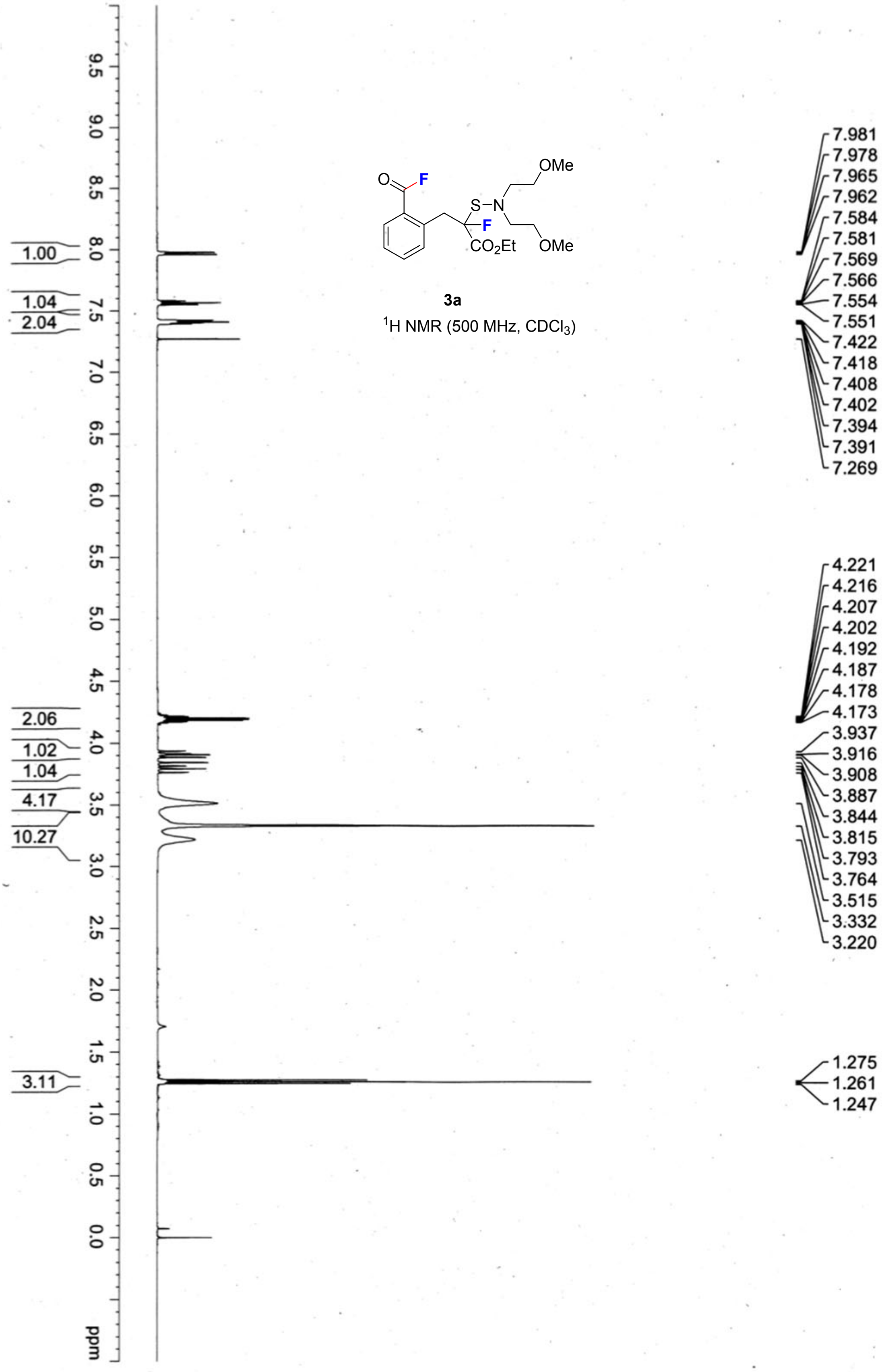
O
F
S
N
F
OMe
OMe
CO2Et
3a
1H NMR (500 MHz, CDCl3)
7.981
7.978
7.965
7.962
7.584
7.581
7.569
7.566
7.554
7.551
7.422
7.418
7.408
7.402
7.394
7.391
7.269
4.221
4.216
4.207
4.202
4.192
4.187
4.178
4.173
3.937
3.916
3.908
3.887
3.844
3.815
3.793
3.764
3.515
3.332
3.220
1.275
1.261
1.247
1.00
1.04
2.04
2.06
1.02
1.04
4.17
10.27
3.11
9.5
9.0
8.5
8.0
7.5
7.0
6.5
6.0
5.5
5.0
4.5
4.0
3.5
3.0
2.5
2.0
1.5
1.0
0.5
0.0
ppm

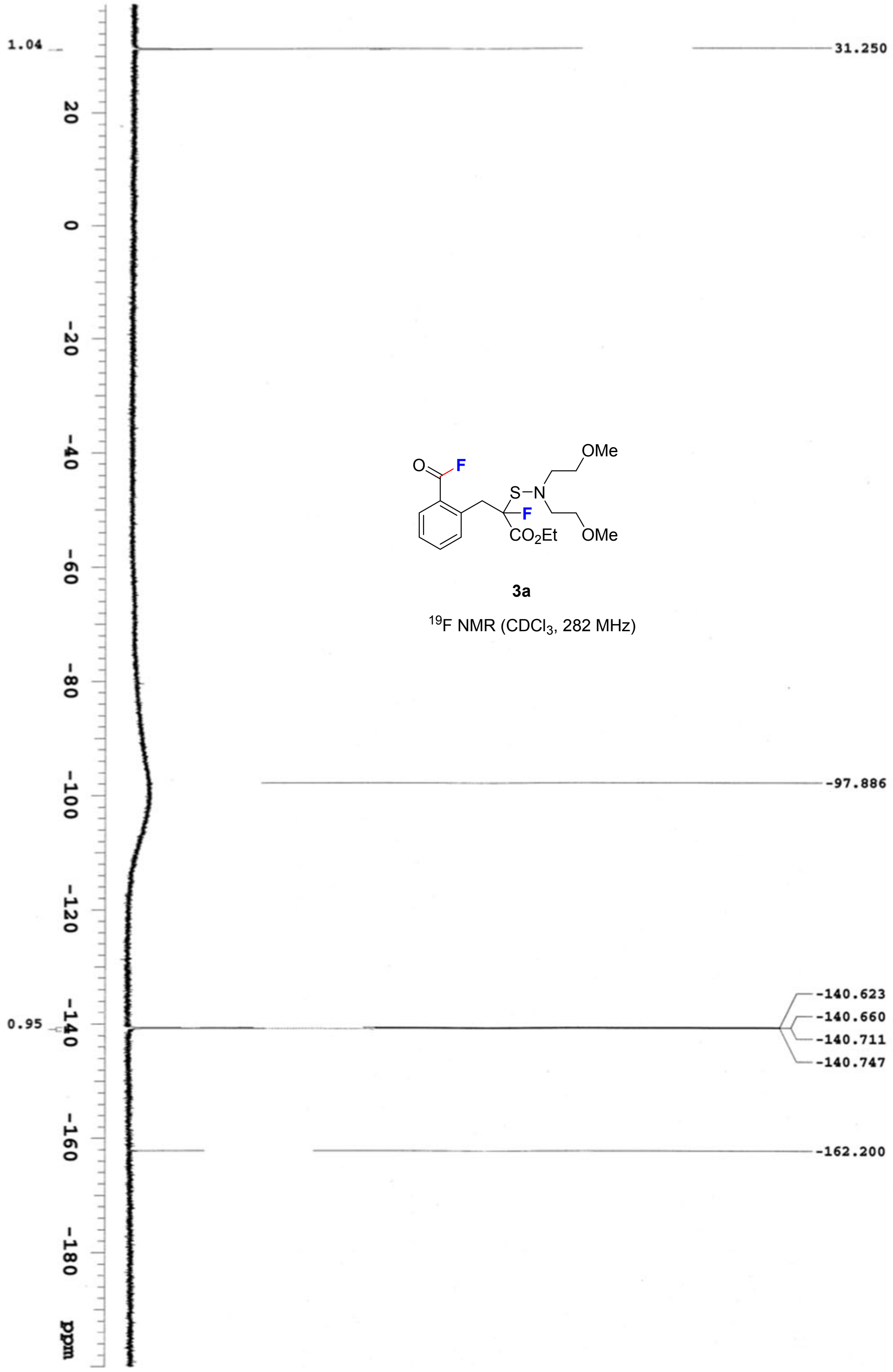

3a

^{19}F NMR ($CDCl_3$, 282 MHz)

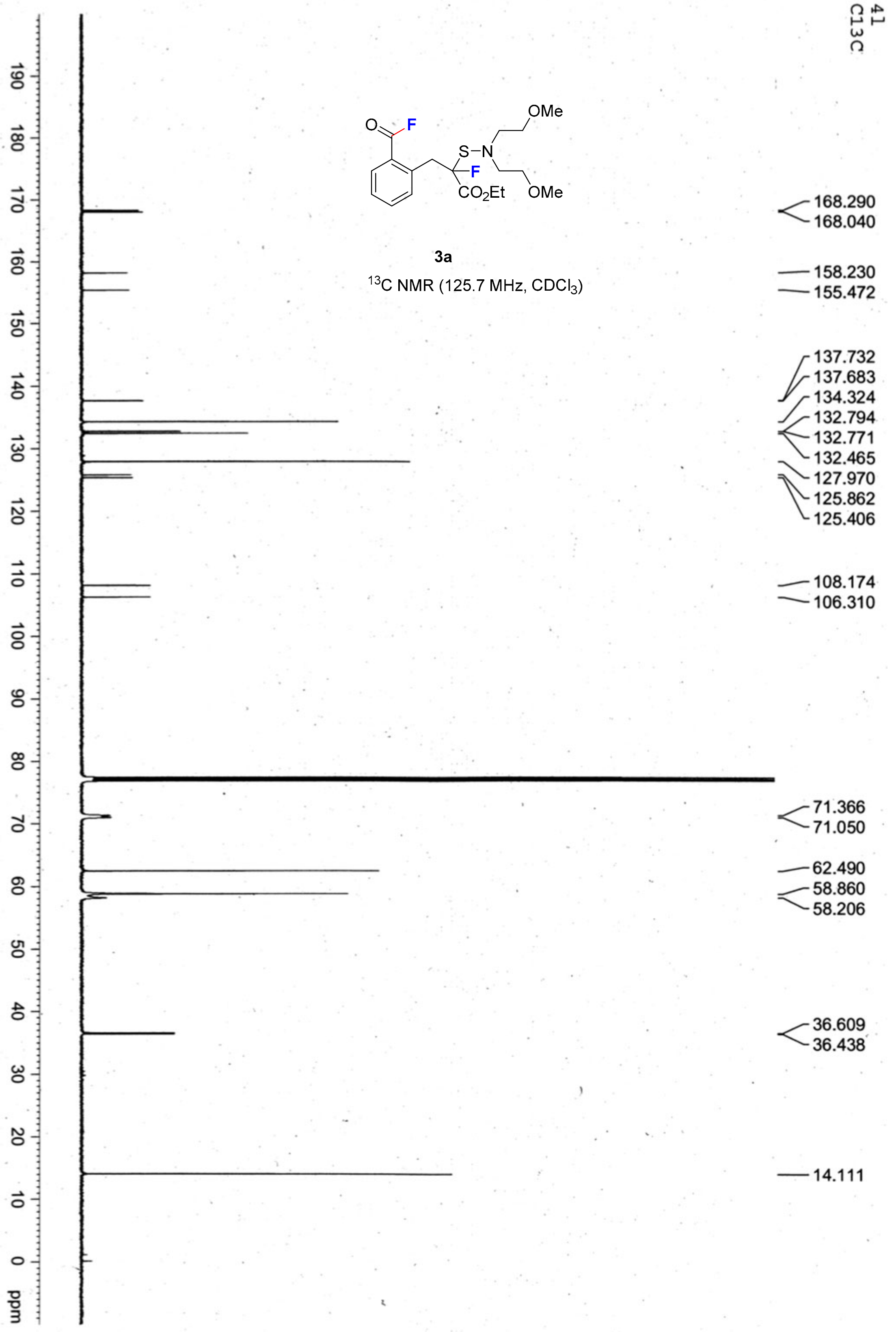

3a

^{13}C NMR (125.7 MHz, $CDCl_3$)

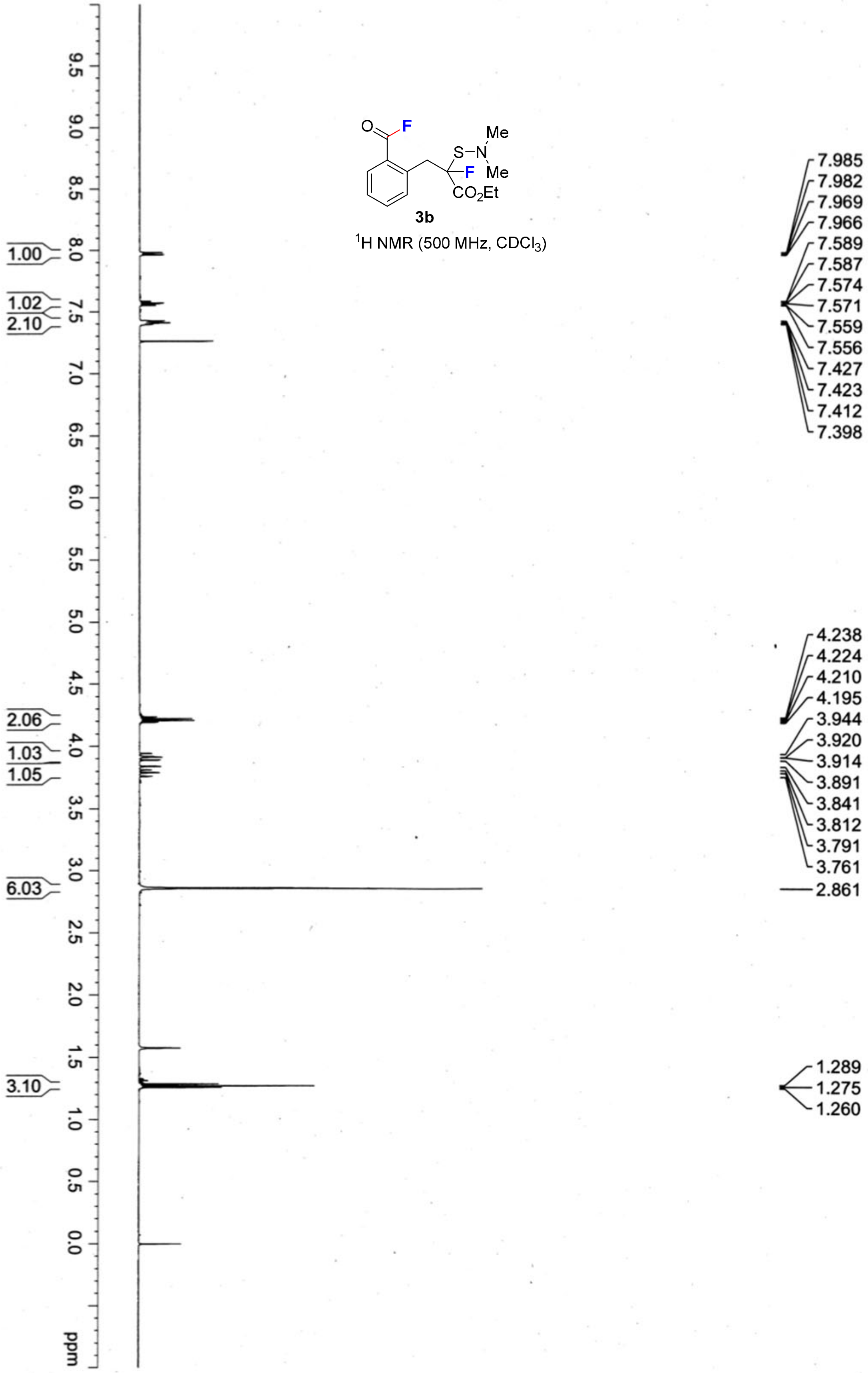

3b

^{1}H NMR (500 MHz, $CDCl_3$)

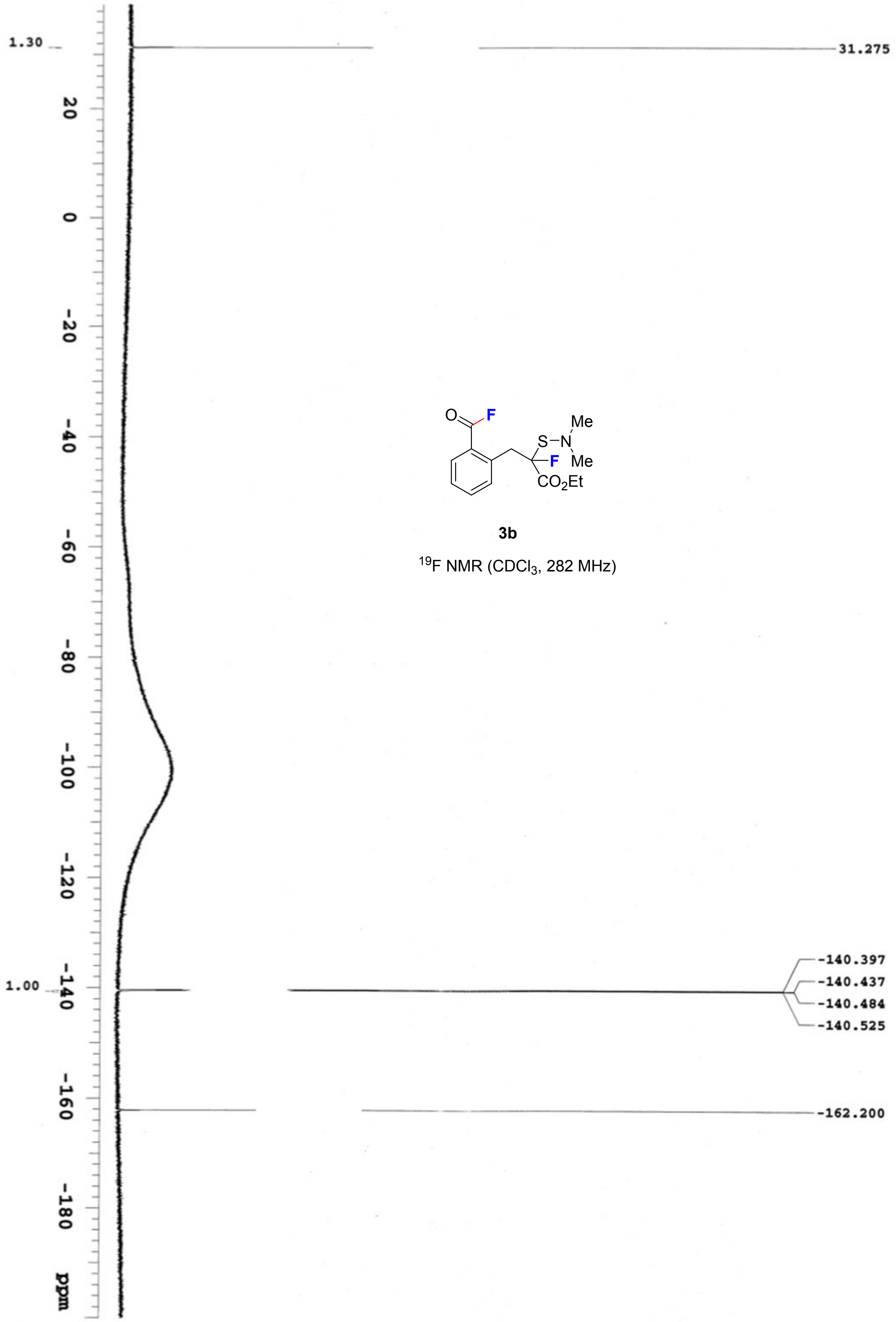

3b

^{19}F NMR ($CDCl_3$, 282 MHz)

3b

^{13}C NMR (125.7 MHz, $CDCl_3$)

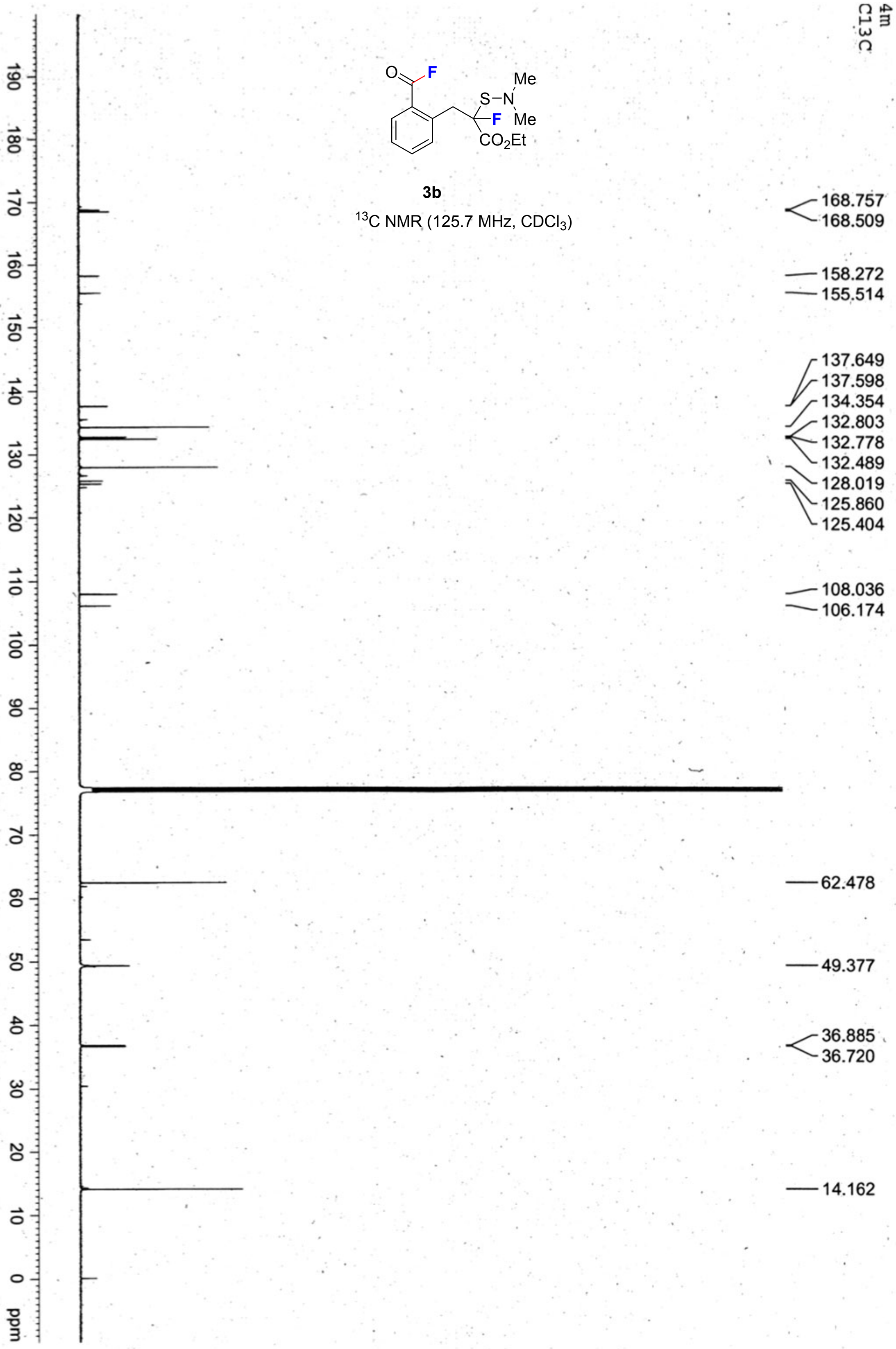

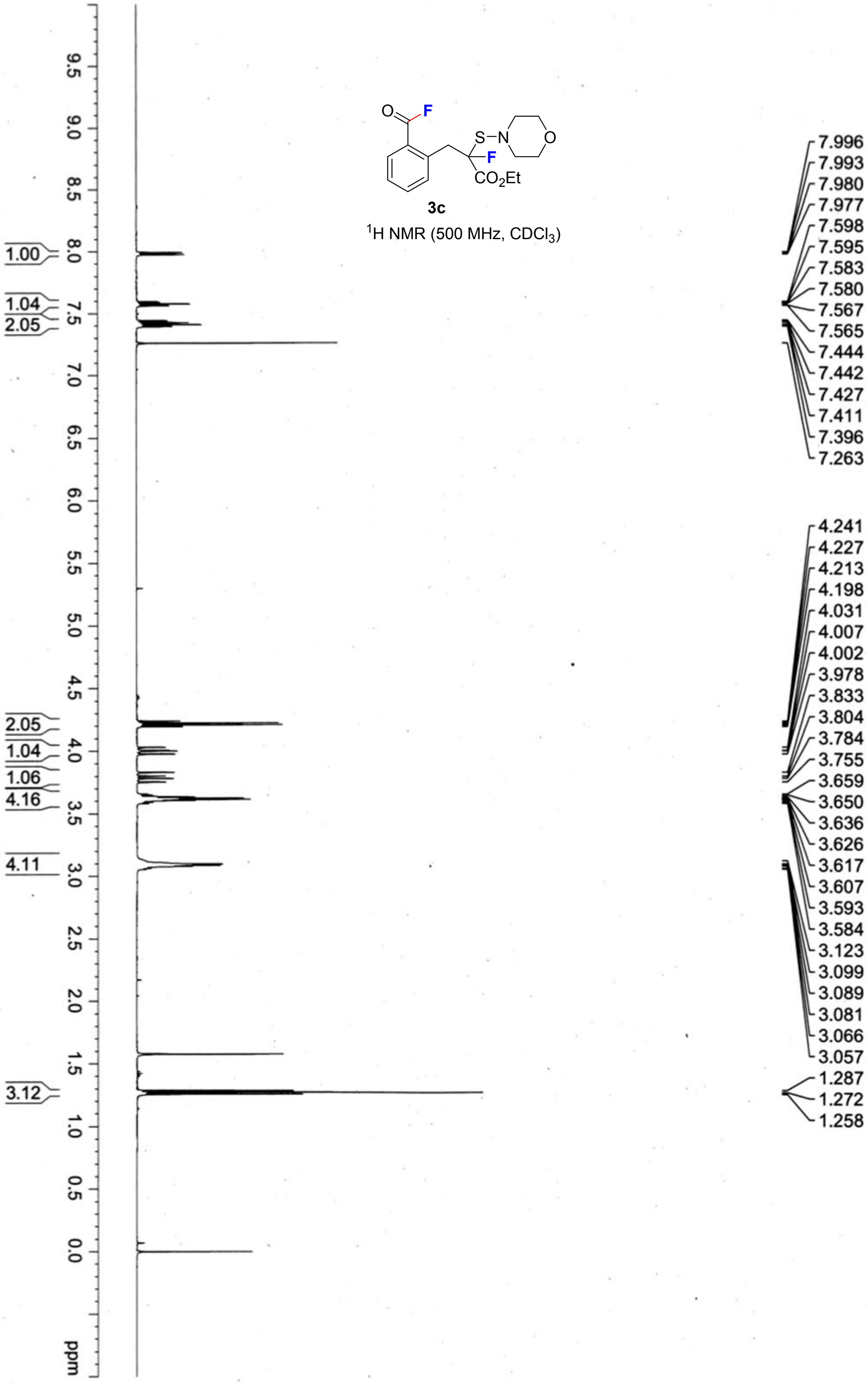

3c

^{1}H NMR (500 MHz, $CDCl_3$)

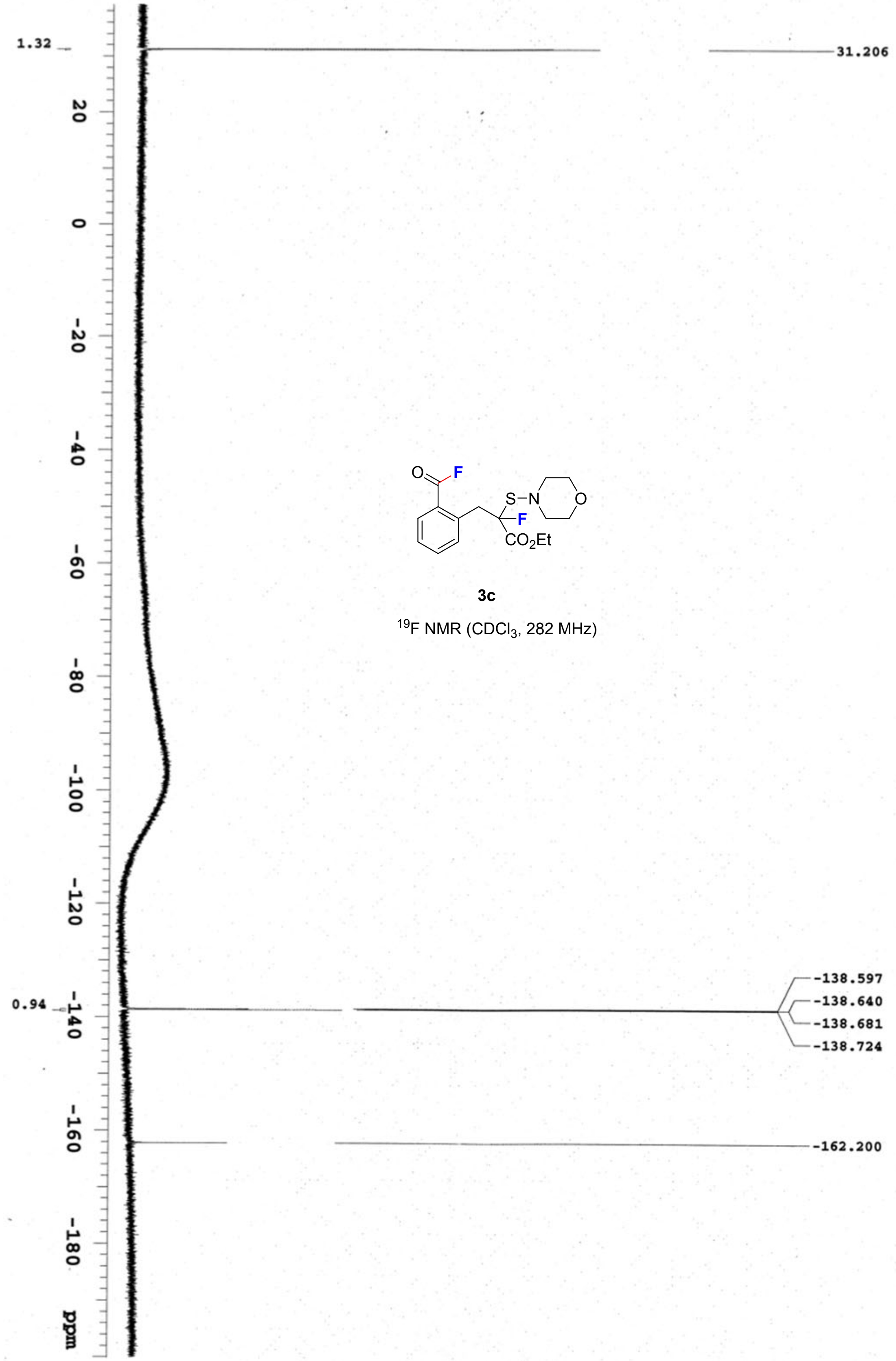

3c

^{19}F NMR ($CDCl_3$, 282 MHz)

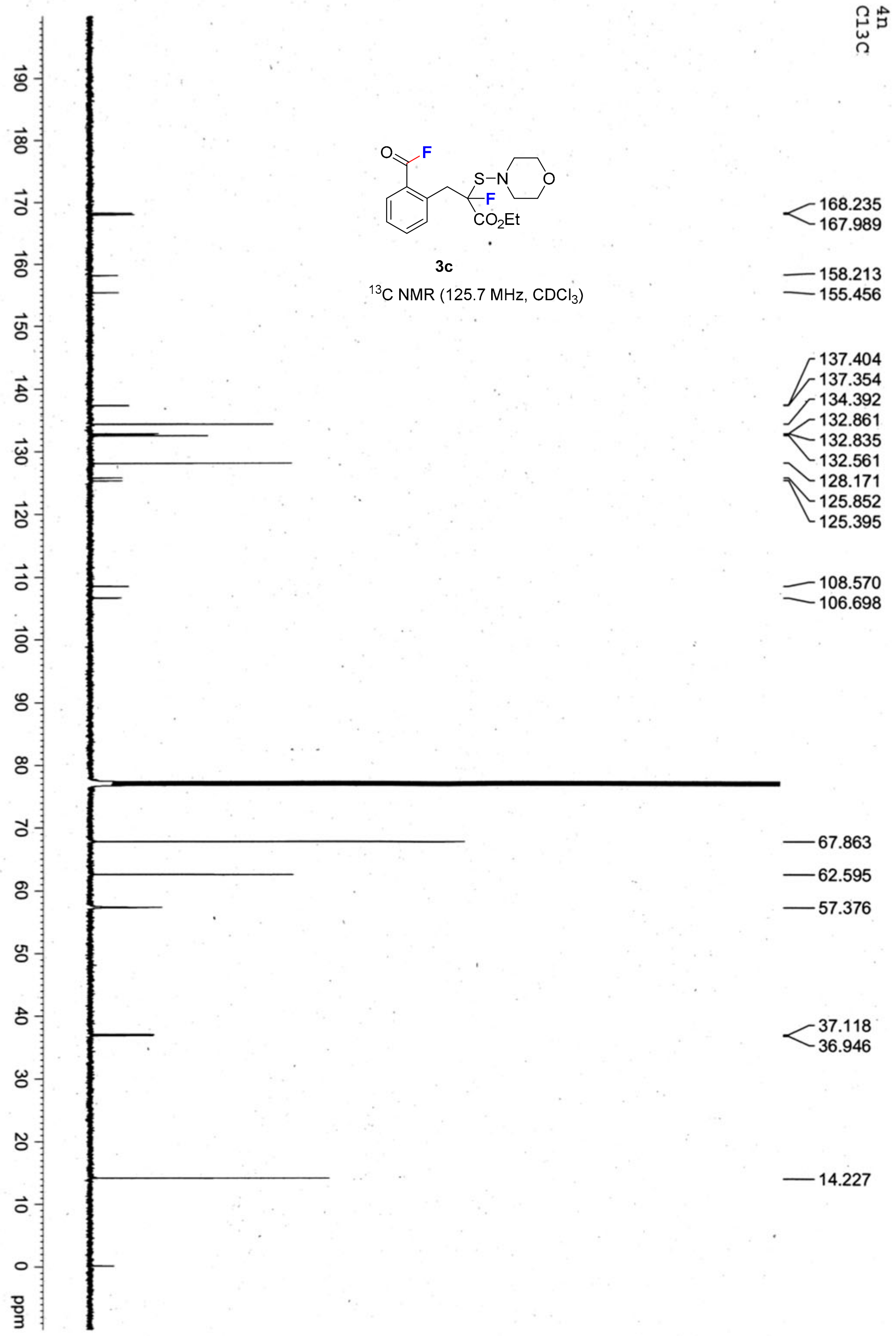
4n
C13C
3c
13C NMR (125.7 MHz, CDCl3)
168.235
167.989
158.213
155.456
137.404
137.354
134.392
132.861
132.835
132.561
128.171
125.852
125.395
108.570
106.698
67.863
62.595
57.376
37.118
36.946
14.227
ppm

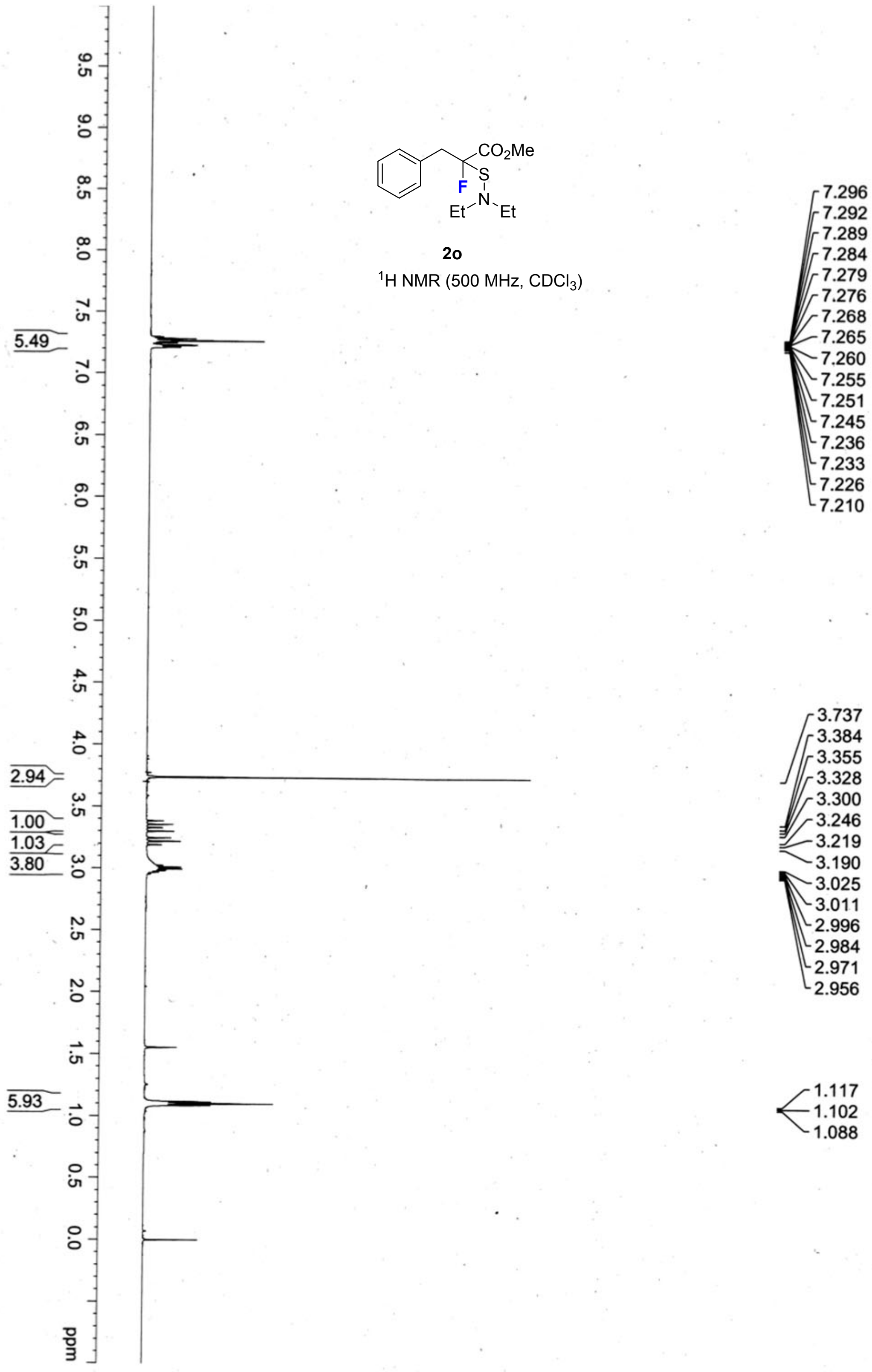

2o

^{1}H NMR (500 MHz, $CDCl_3$)

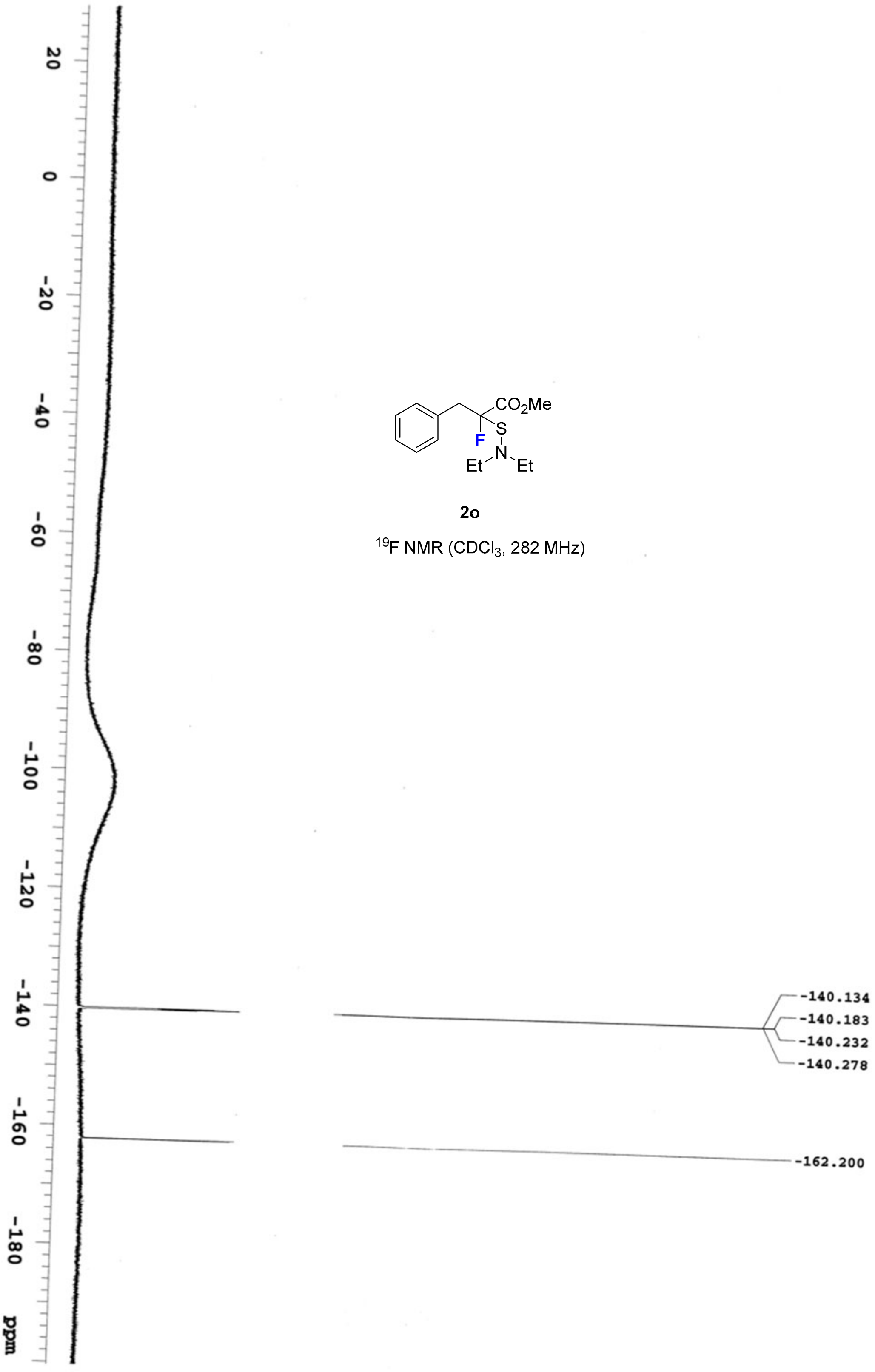

2o

^{19}F NMR ($CDCl_3$, 282 MHz)

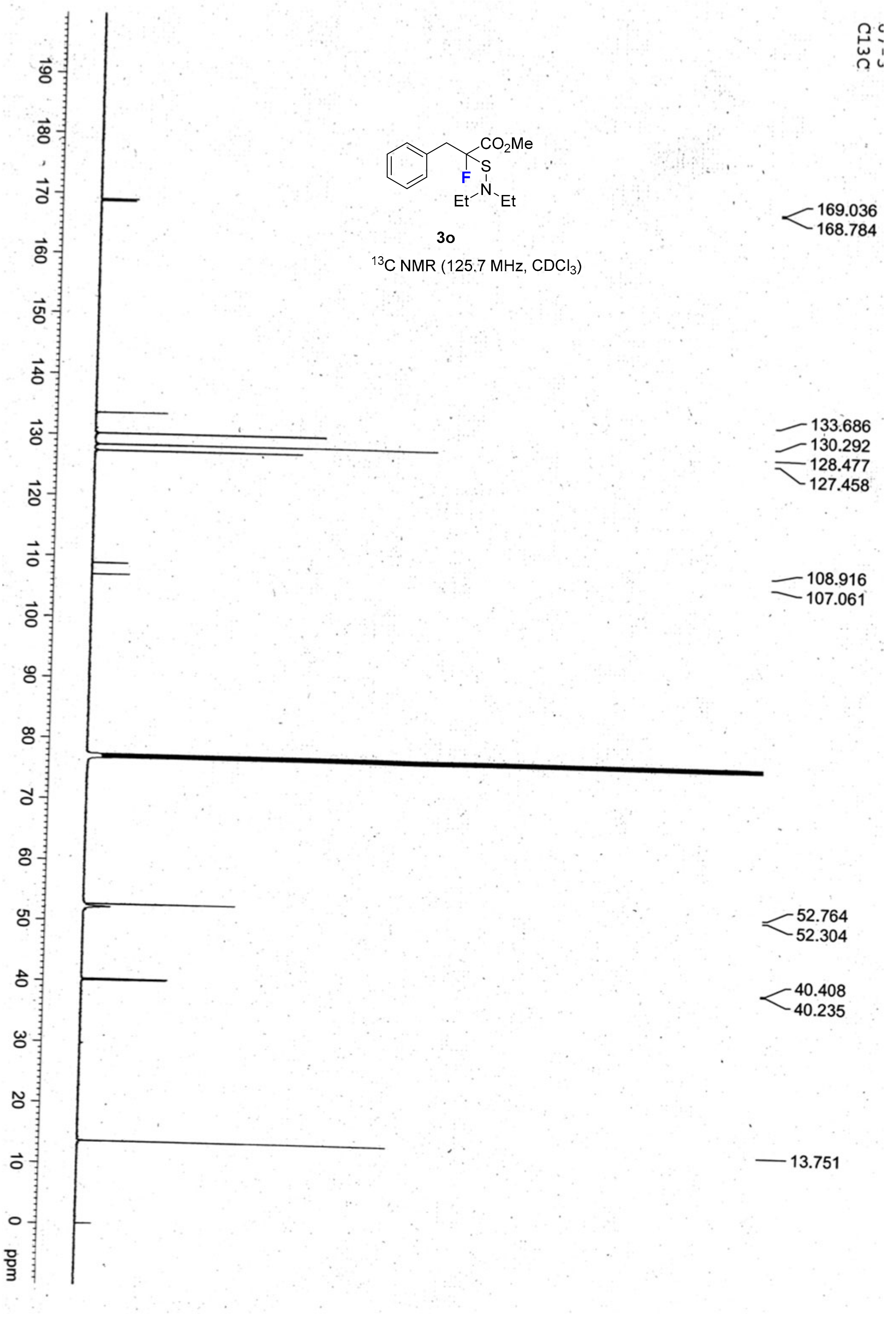

3o

^{13}C NMR (125.7 MHz, $CDCl_3$)

21. ^{1}H NMR, ^{13}C NMR and ^{19}F NMR spectra for doubly fluoro-functionalization compounds 4a—4o (Table 2)

4a

^{1}H NMR (500 MHz, $CDCl_3$)

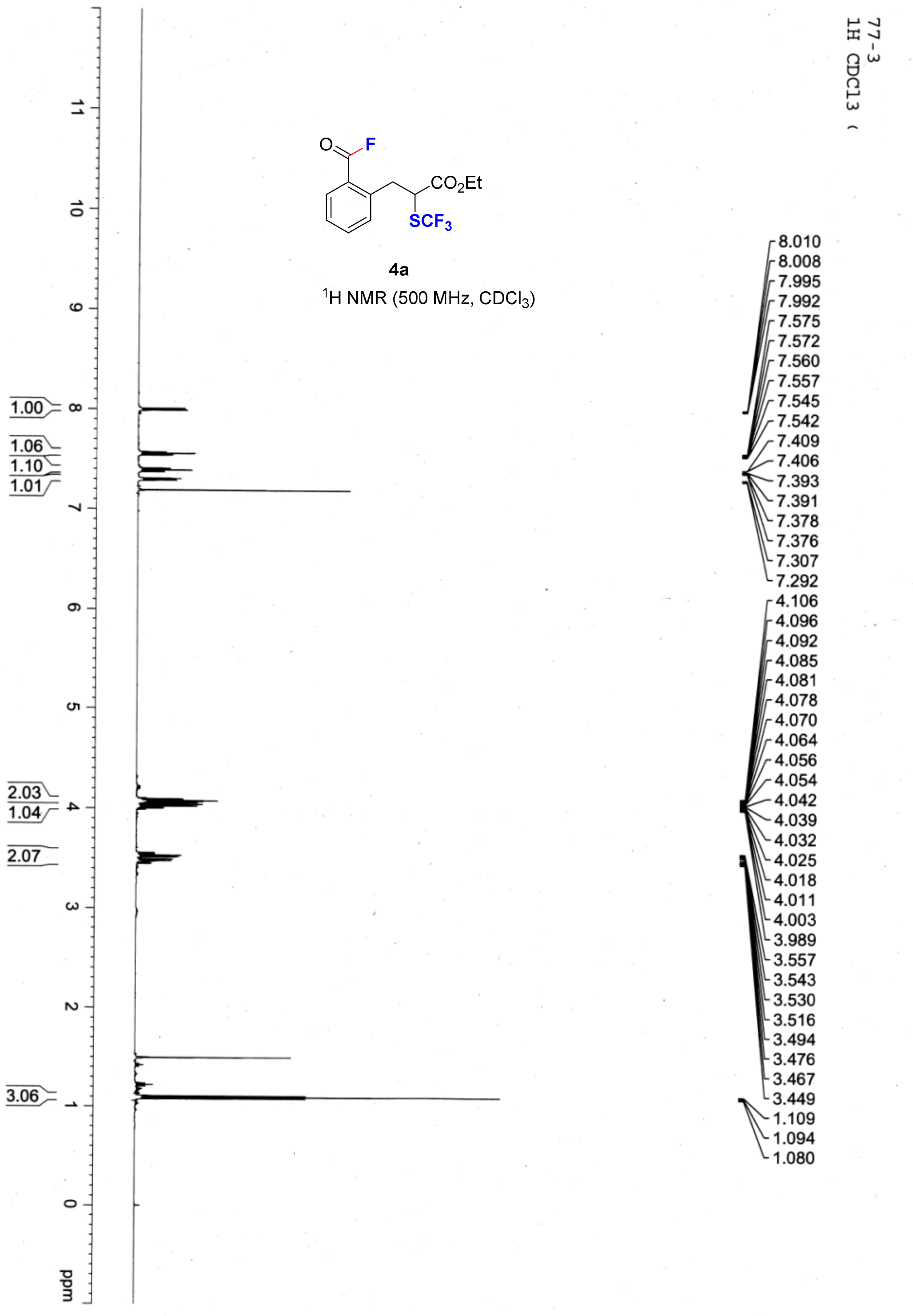

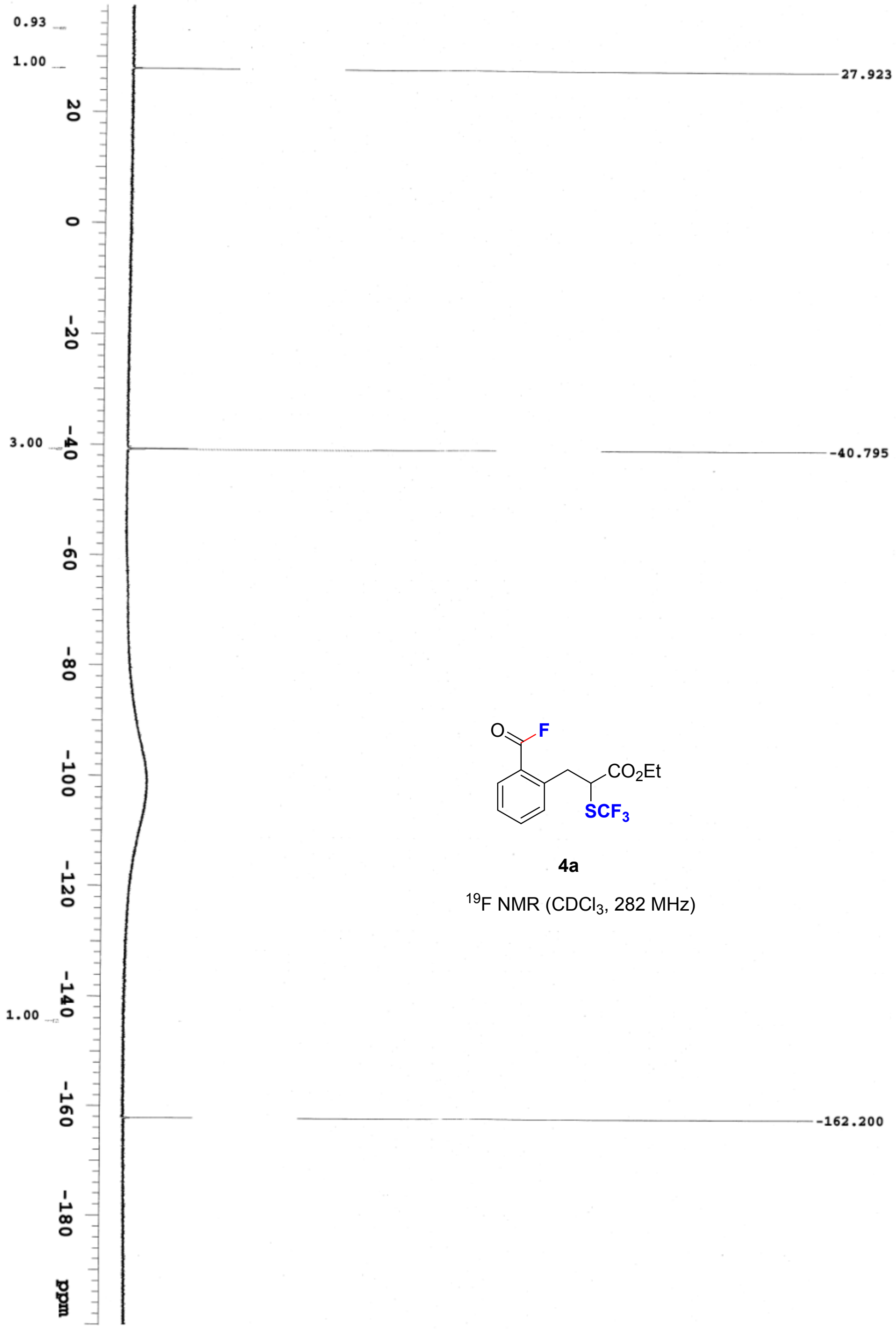

4a

^{19}F NMR ($CDCl_3$, 282 MHz)

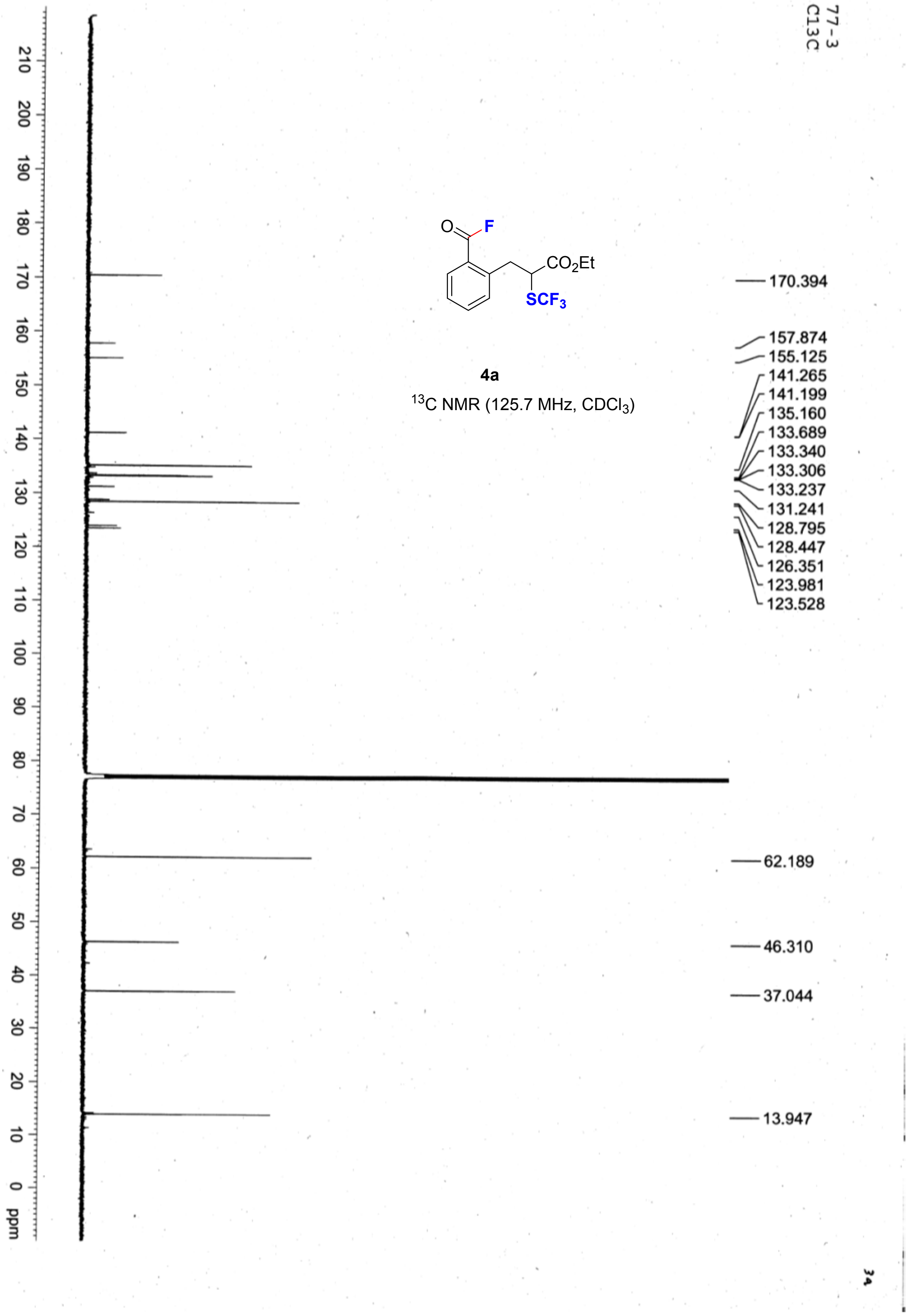

4a

^{13}C NMR (125.7 MHz, $CDCl_3$)

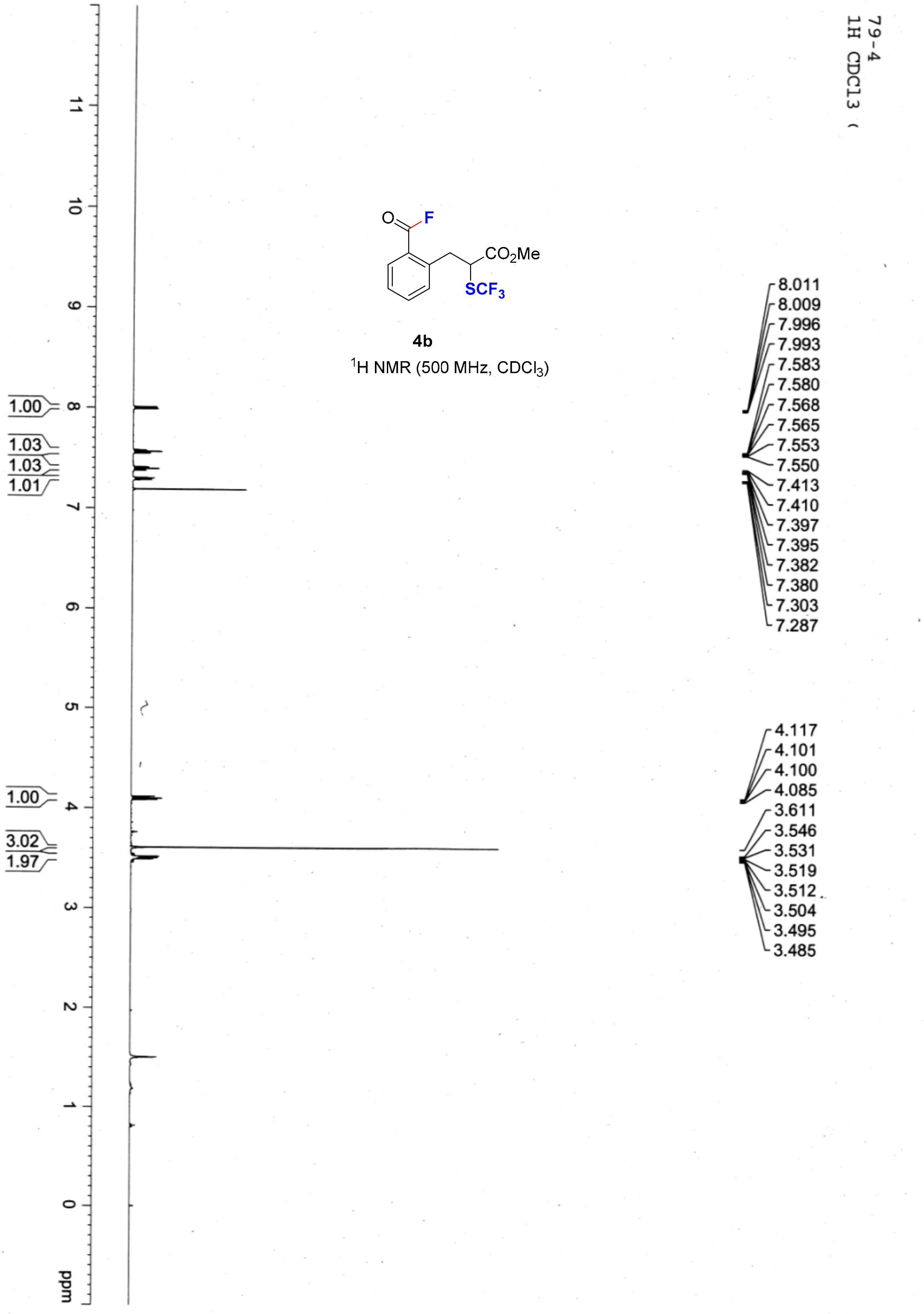

4b

^{1}H NMR (500 MHz, $CDCl_3$)

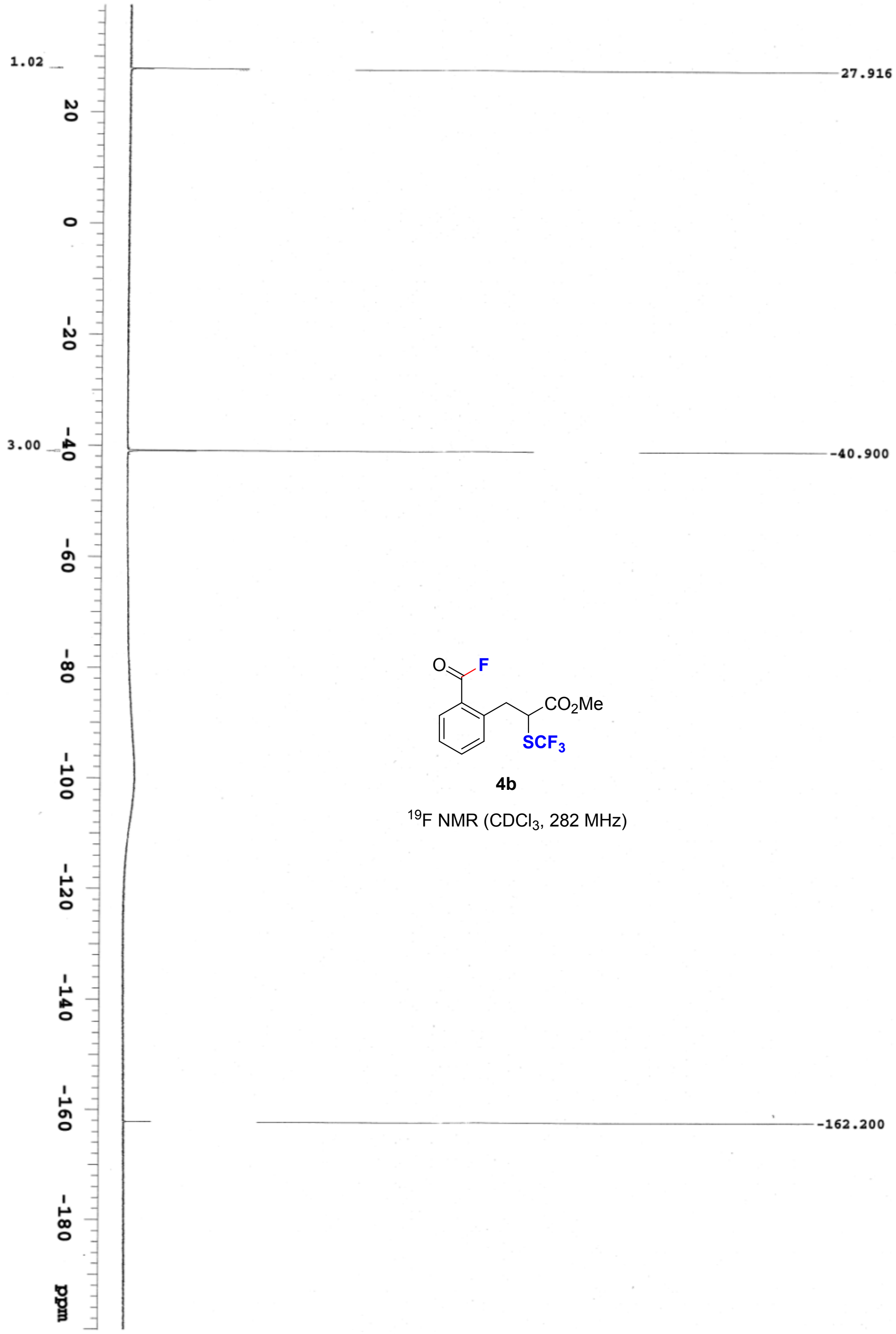

4b

^{19}F NMR ($CDCl_3$, 282 MHz)

3b

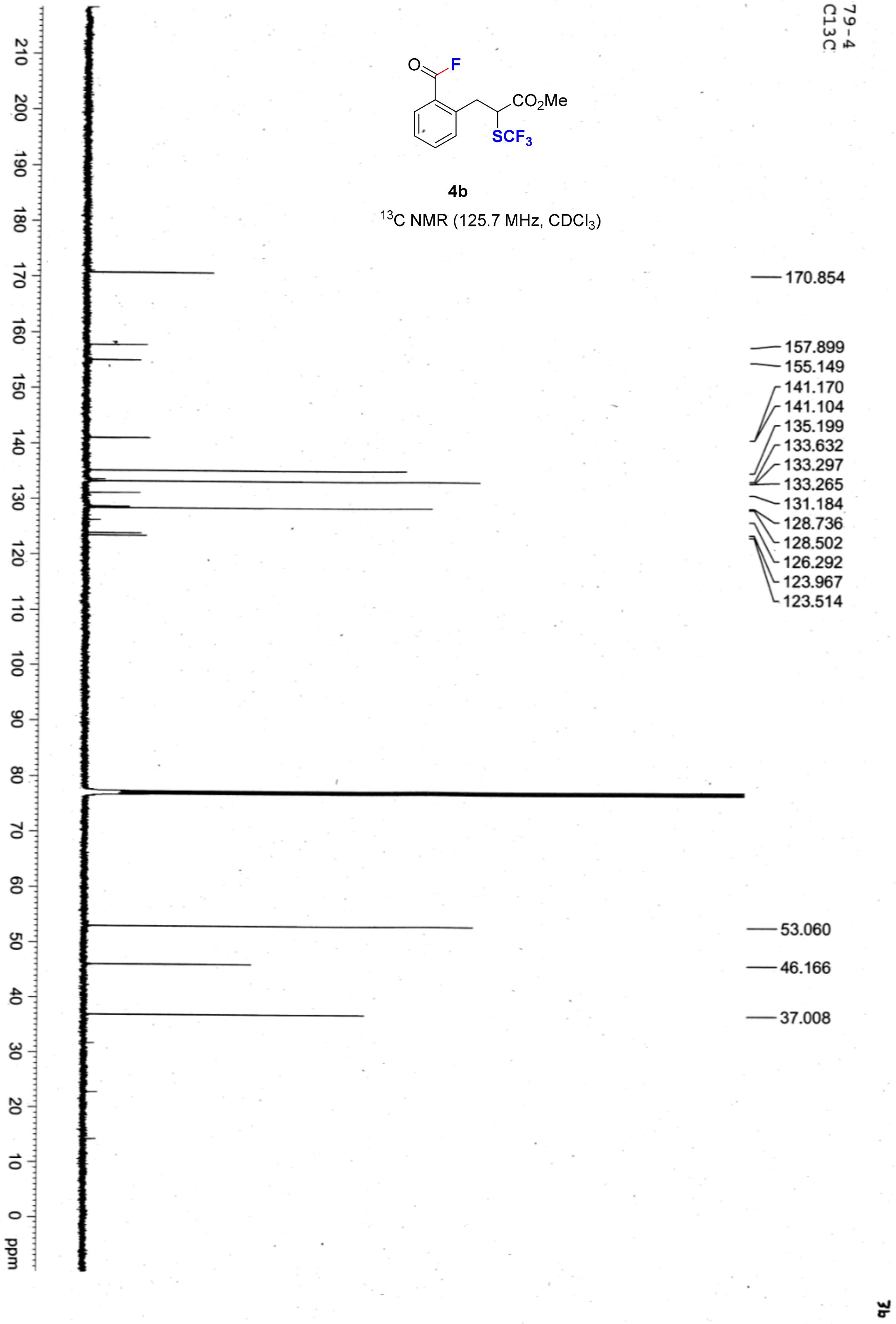

4b

^{13}C NMR (125.7 MHz, $CDCl_3$)

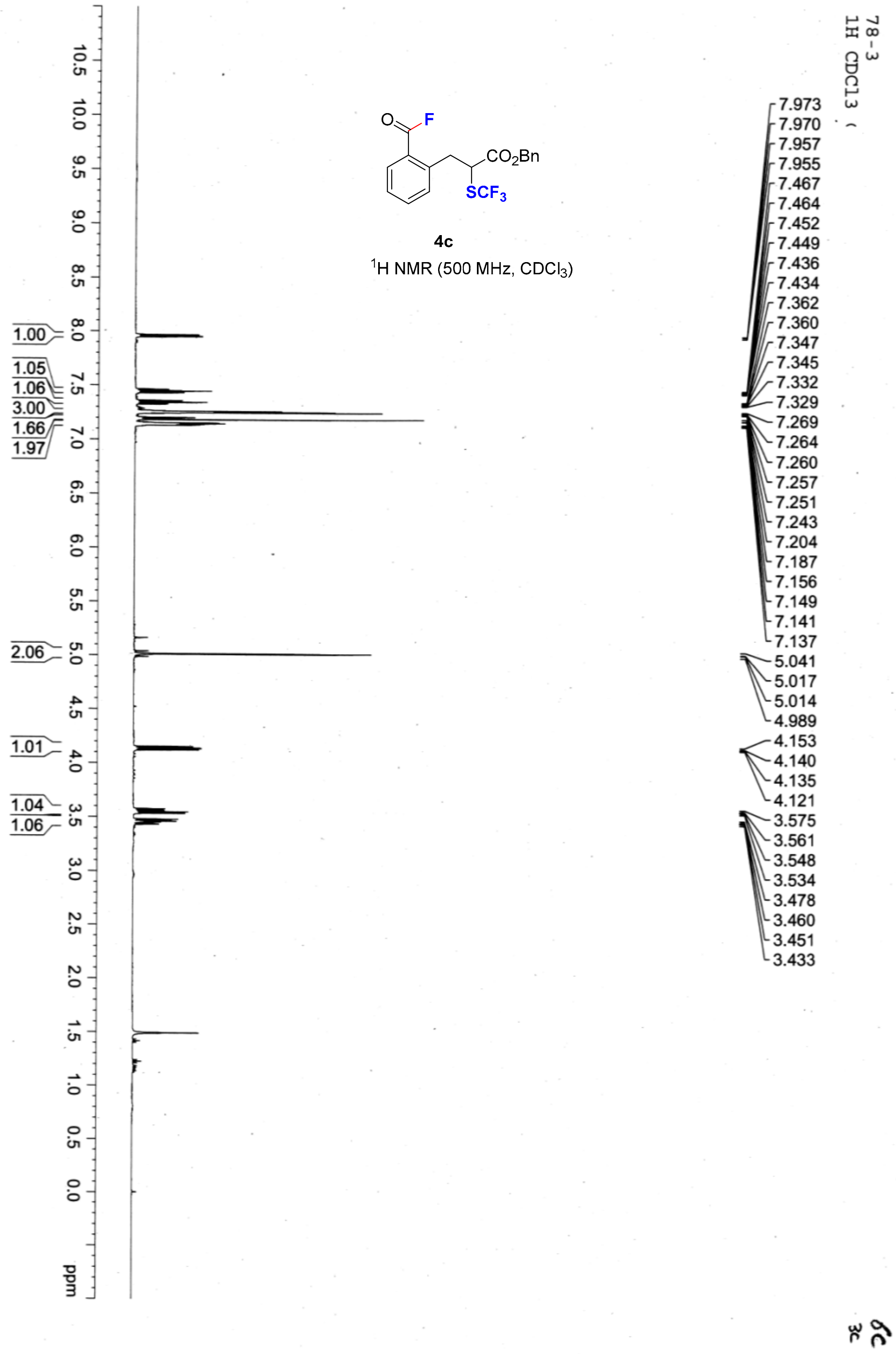

4c

^{1}H NMR (500 MHz, $CDCl_3$)

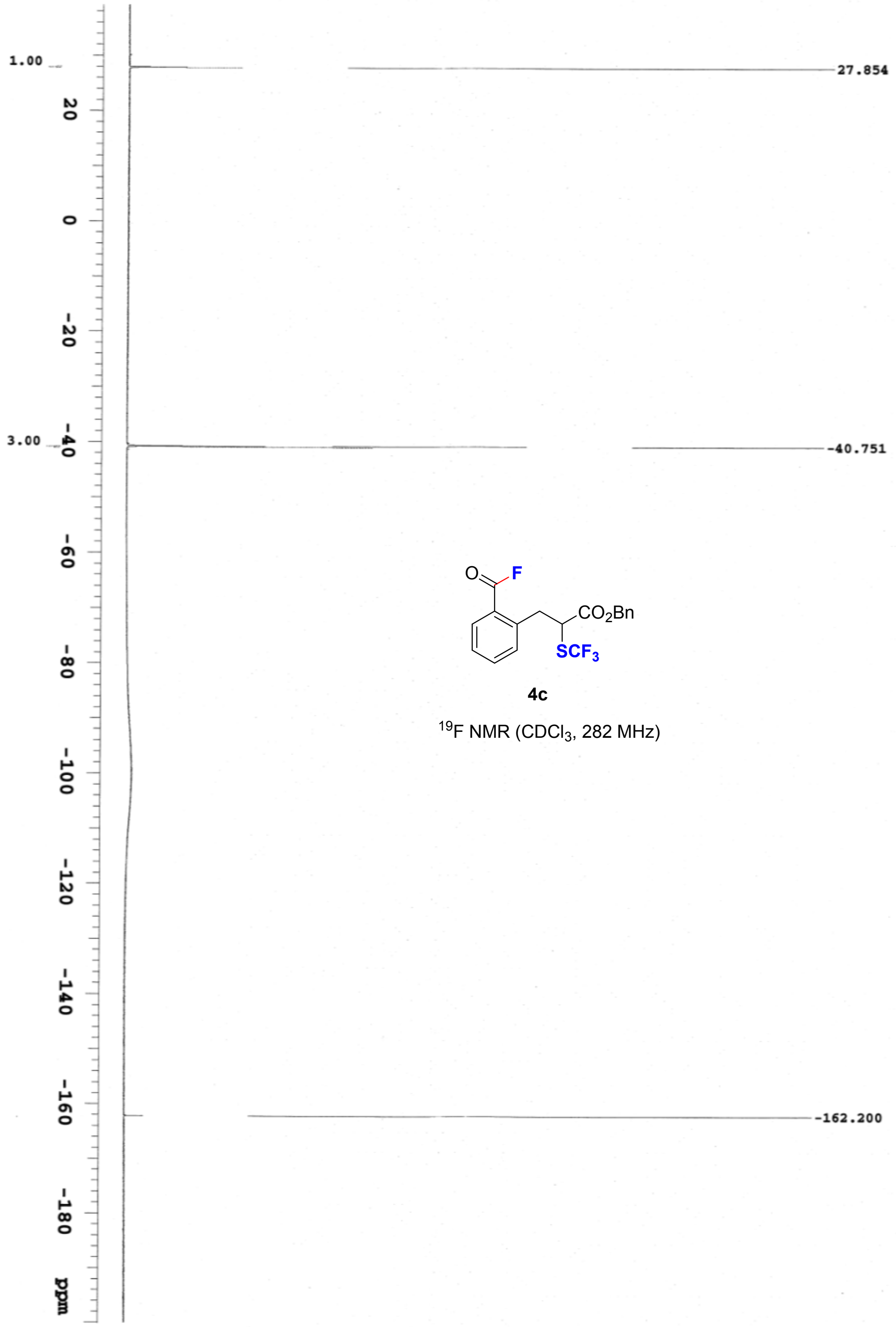

4c

^{19}F NMR ($CDCl_3$, 282 MHz)

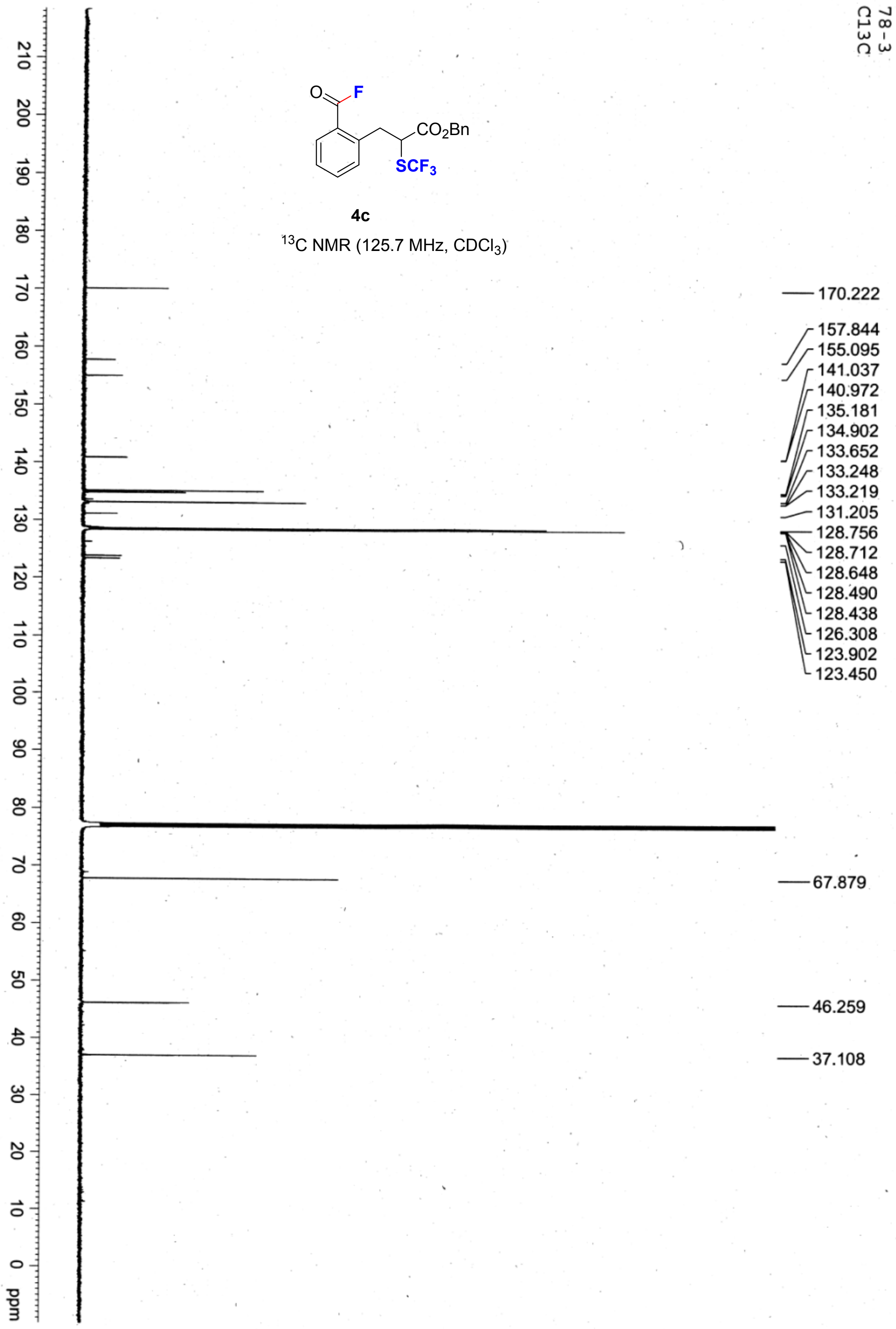

4c

^{13}C NMR (125.7 MHz, $CDCl_3$)

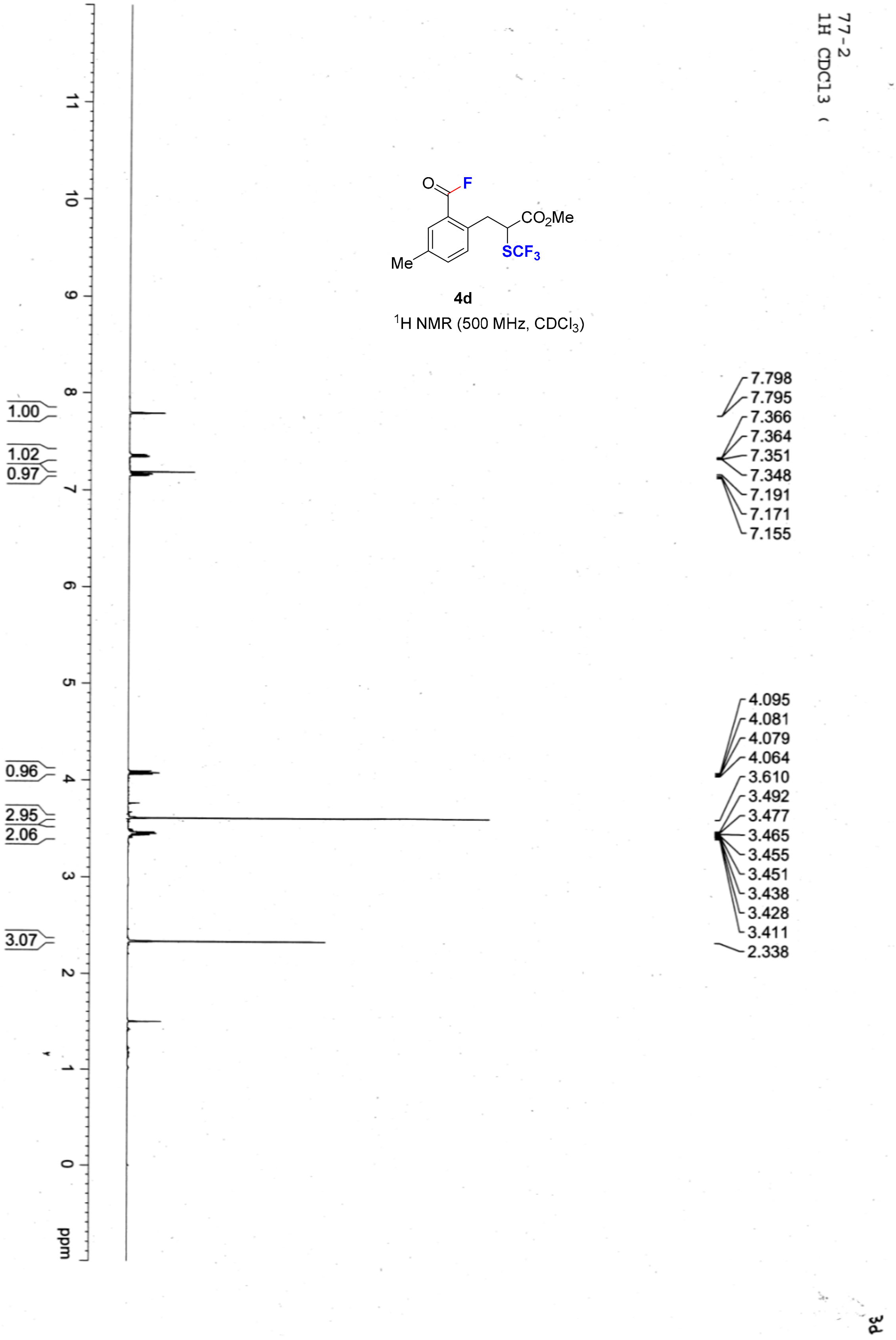
77-2
1H CDCl3 (
4d
^{1}H NMR (500 MHz, $CDCl_3$)
O
F
CO_2Me
SCF_3
Me
7.798
7.795
7.366
7.364
7.351
7.348
7.191
7.171
7.155
4.095
4.081
4.079
4.064
3.610
3.492
3.477
3.465
3.455
3.451
3.438
3.428
3.411
2.338
1.00
1.02
0.97
0.96
2.95
2.06
3.07
11
10
9
8
7
6
5
4
3
2
1
0
ppm
3d

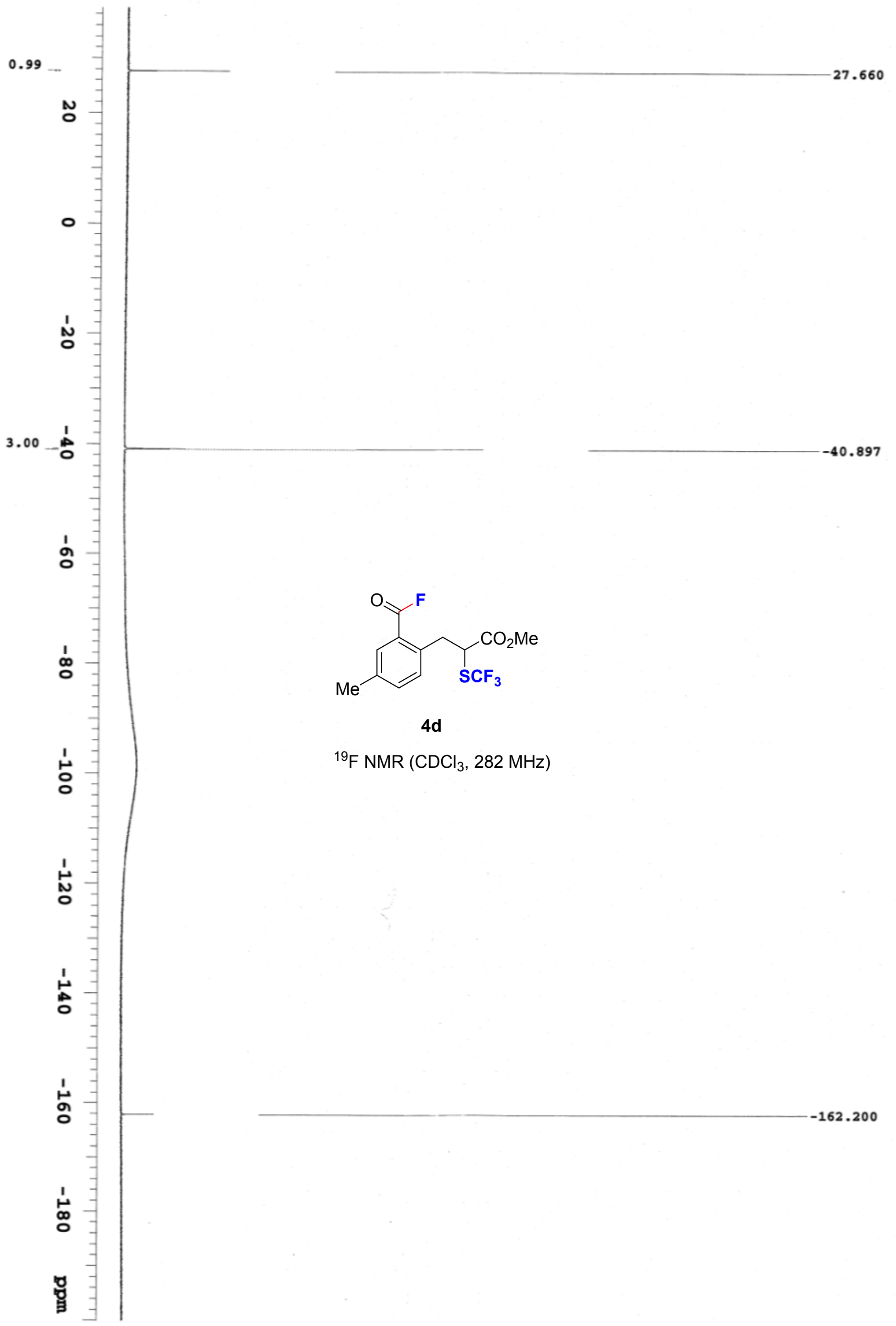

4d

^{19}F NMR ($CDCl_3$, 282 MHz)

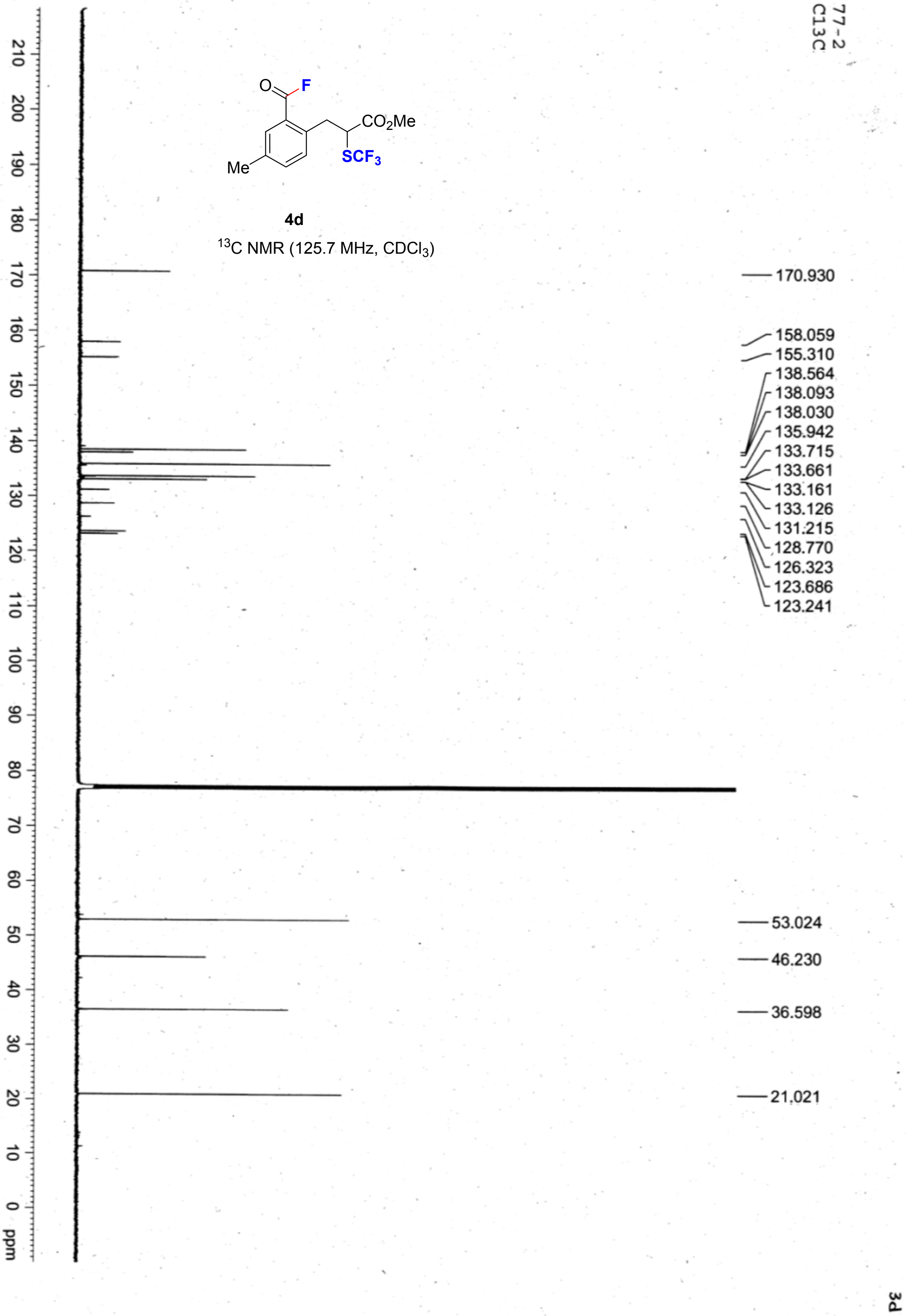

4d

^{13}C NMR (125.7 MHz, $CDCl_3$)

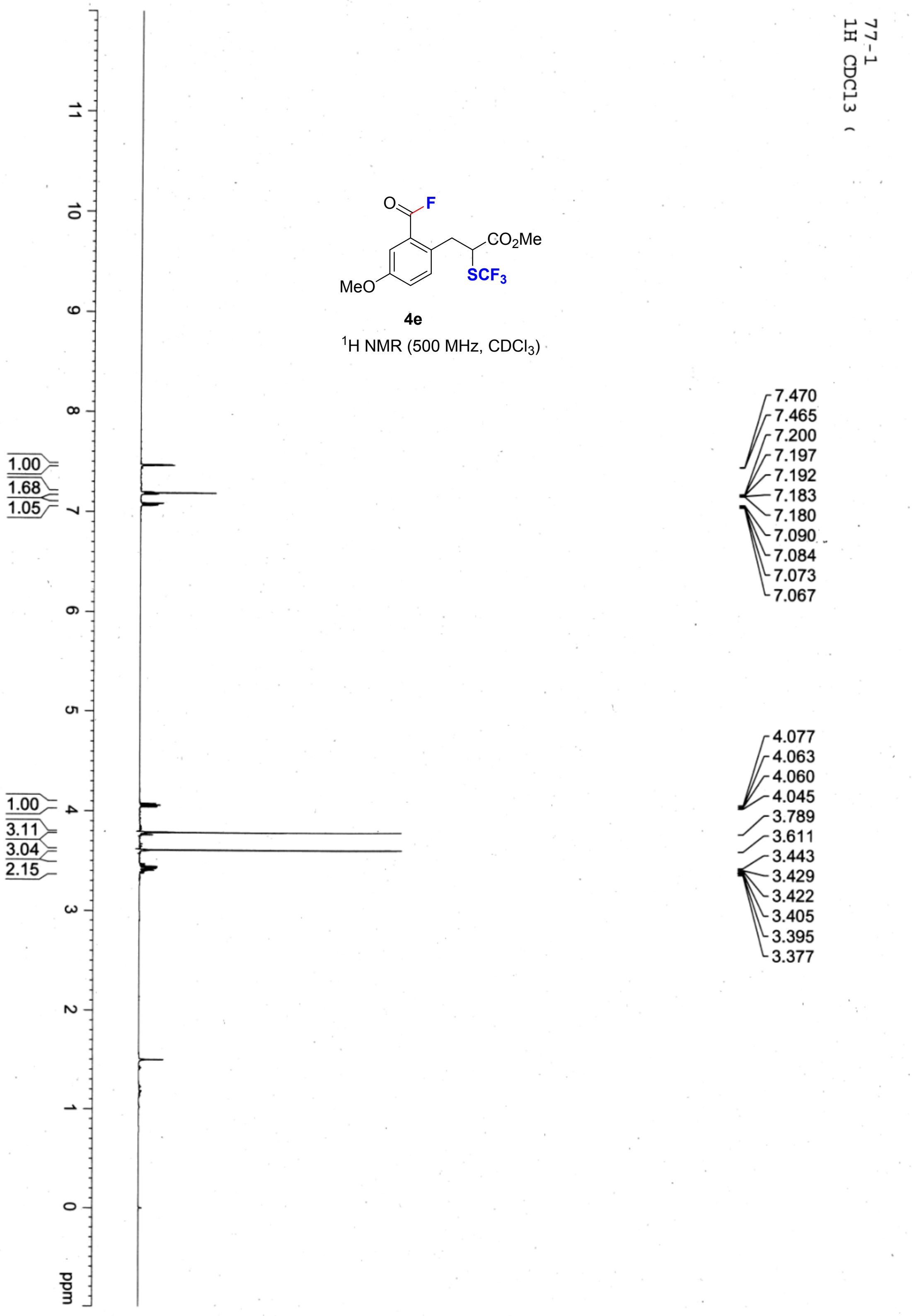

4e

^{1}H NMR (500 MHz, $CDCl_3$)

3e

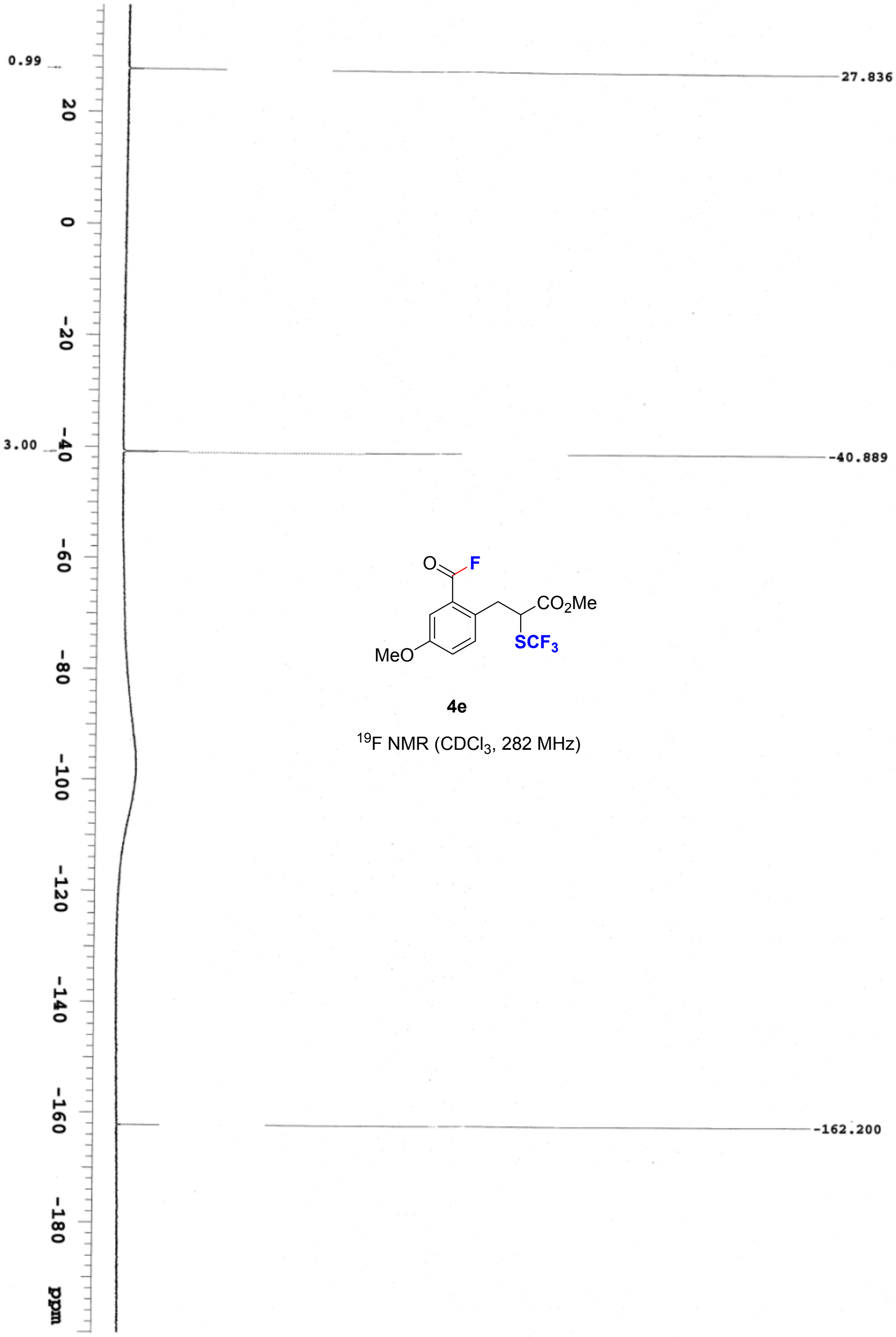

4e

^{19}F NMR ($CDCl_3$, 282 MHz)

3e

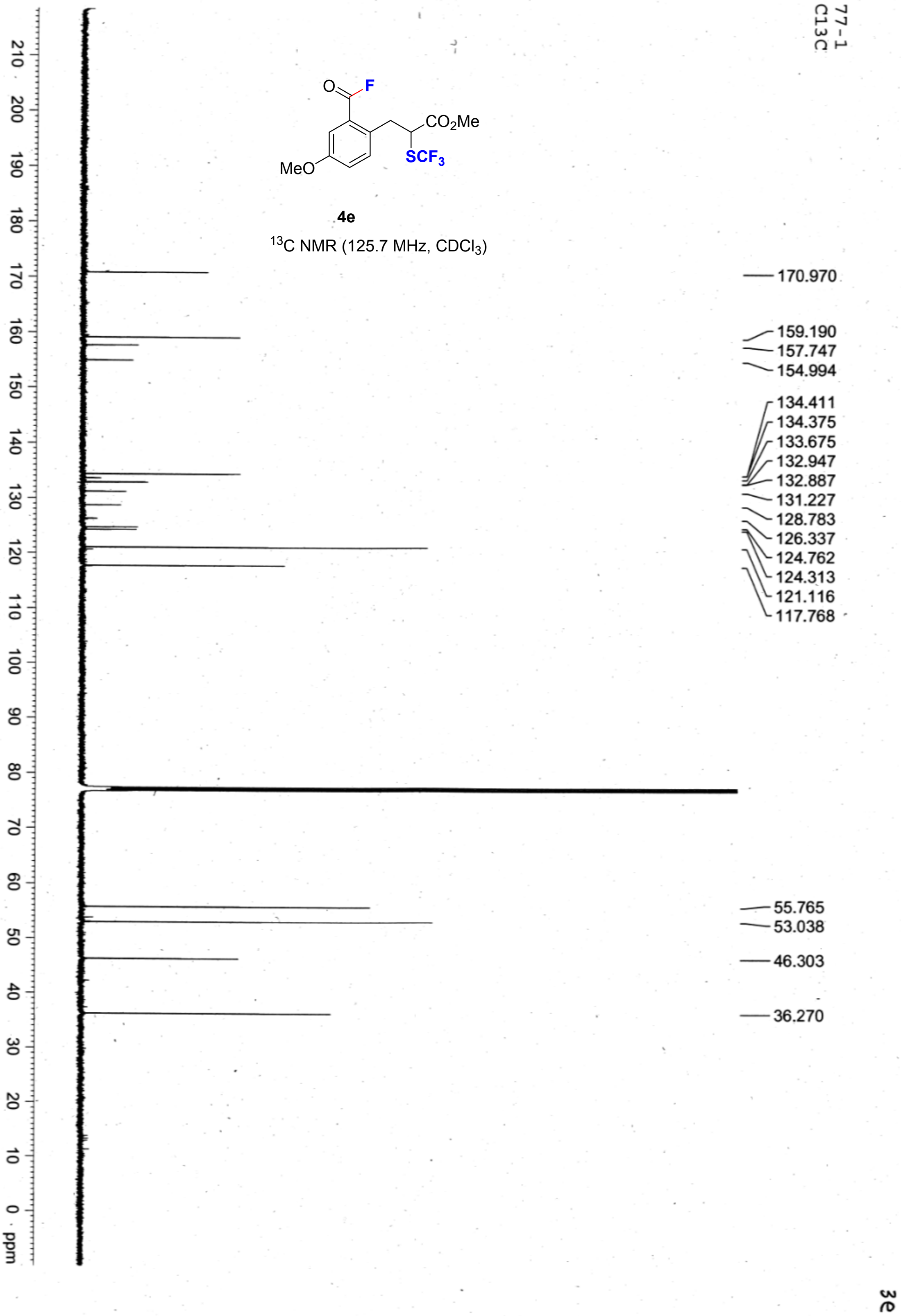
77-1
C13C
CO2Me
MeO
SCF3
O
F
4e
13C NMR (125.7 MHz, CDCl3)
170.970
159.190
157.747
154.994
134.411
134.375
133.675
132.947
132.887
131.227
128.783
126.337
124.762
124.313
121.116
117.768
55.765
53.038
46.303
36.270
210
200
190
180
170
160
150
140
130
120
110
100
90
80
70
60
50
40
30
20
10
0
ppm
3e

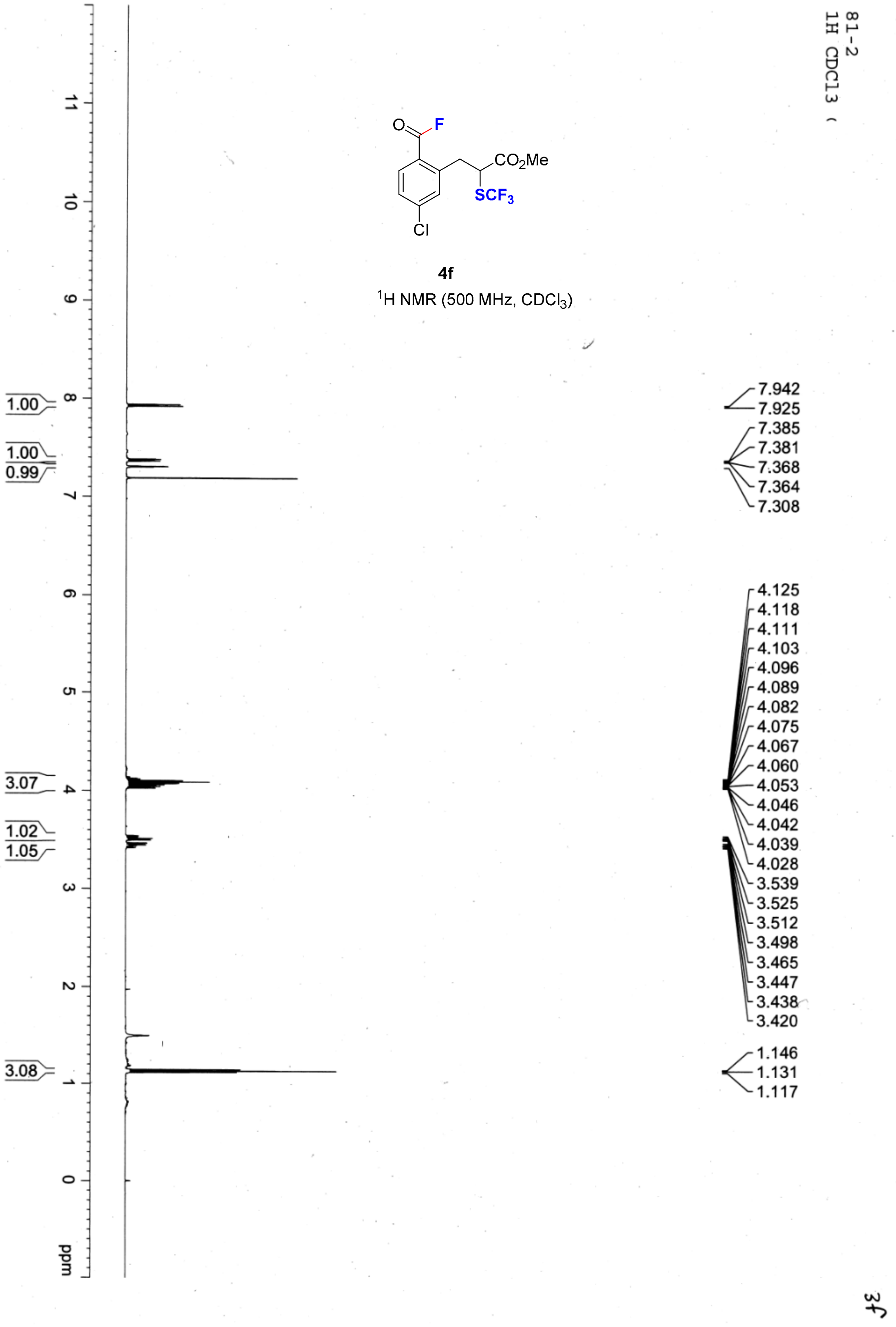

4f

^1H NMR (500 MHz, $CDCl_3$)

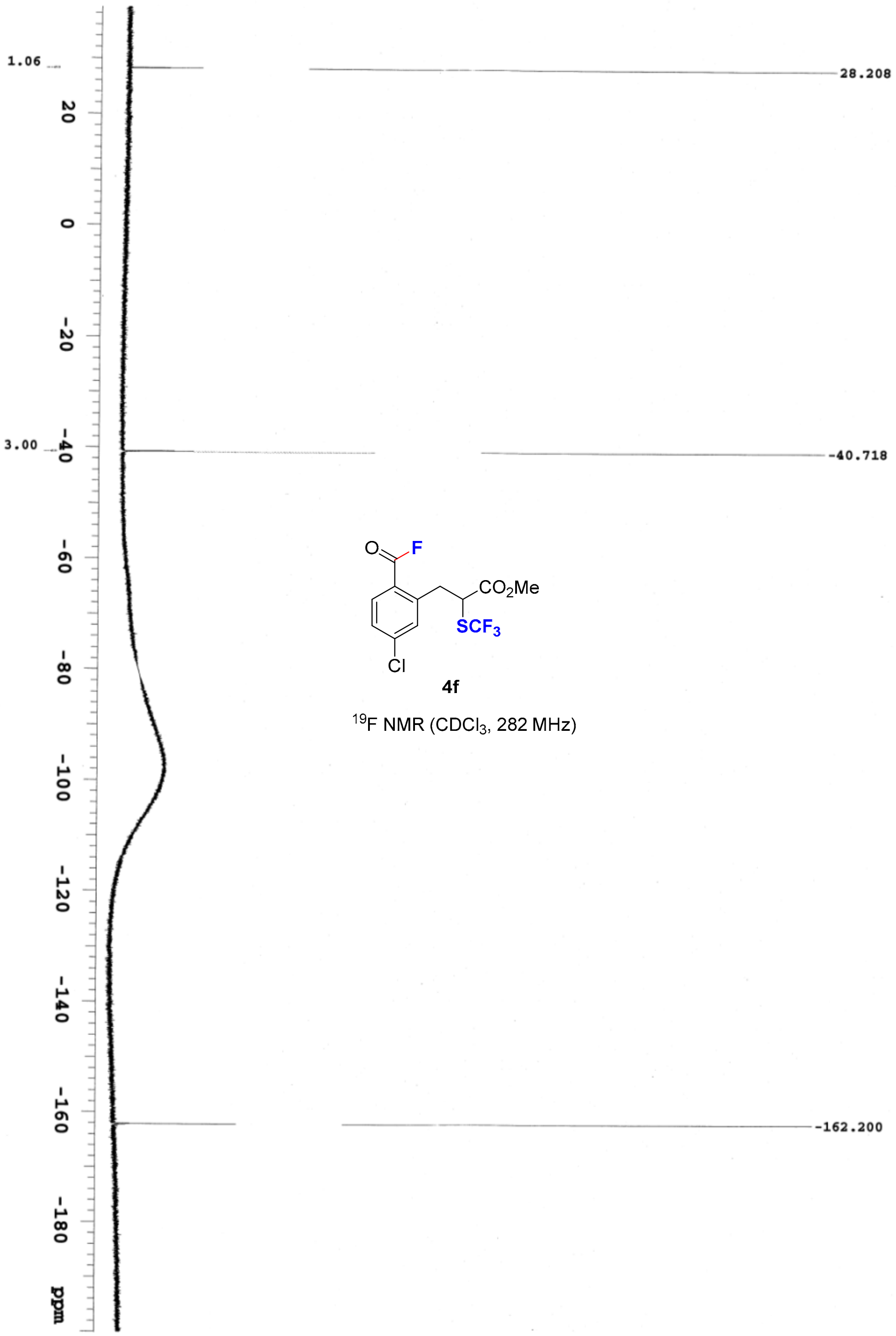

4f

^{19}F NMR ($CDCl_3$, 282 MHz)

3f

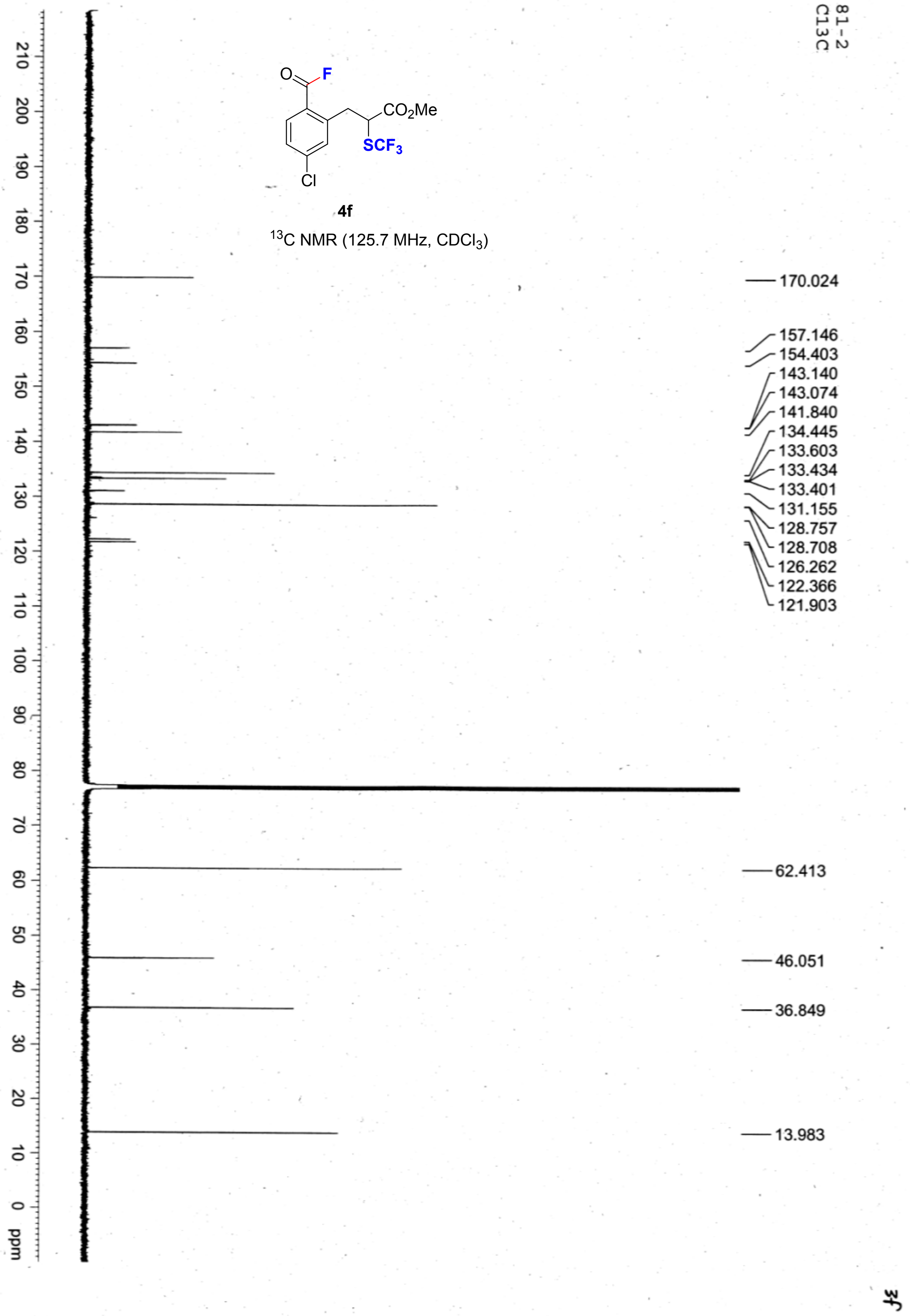
81-2
C13C
4f
^{13}C NMR (125.7 MHz, $CDCl_3$)
170.024
157.146
154.403
143.140
143.074
141.840
134.445
133.603
133.434
133.401
131.155
128.757
128.708
126.262
122.366
121.903
62.413
46.051
36.849
13.983
ppm

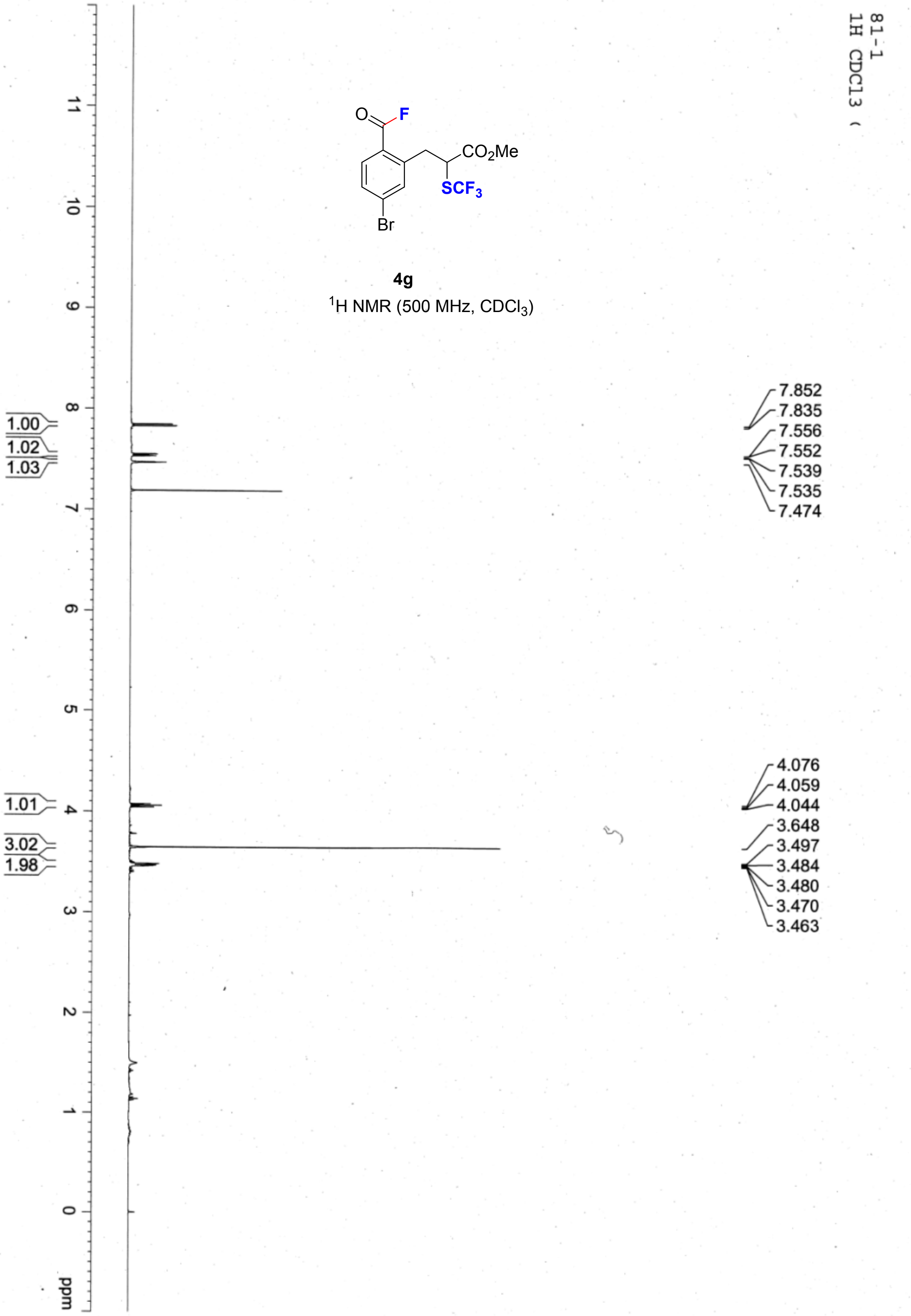

4g

^{1}H NMR (500 MHz, $CDCl_3$)

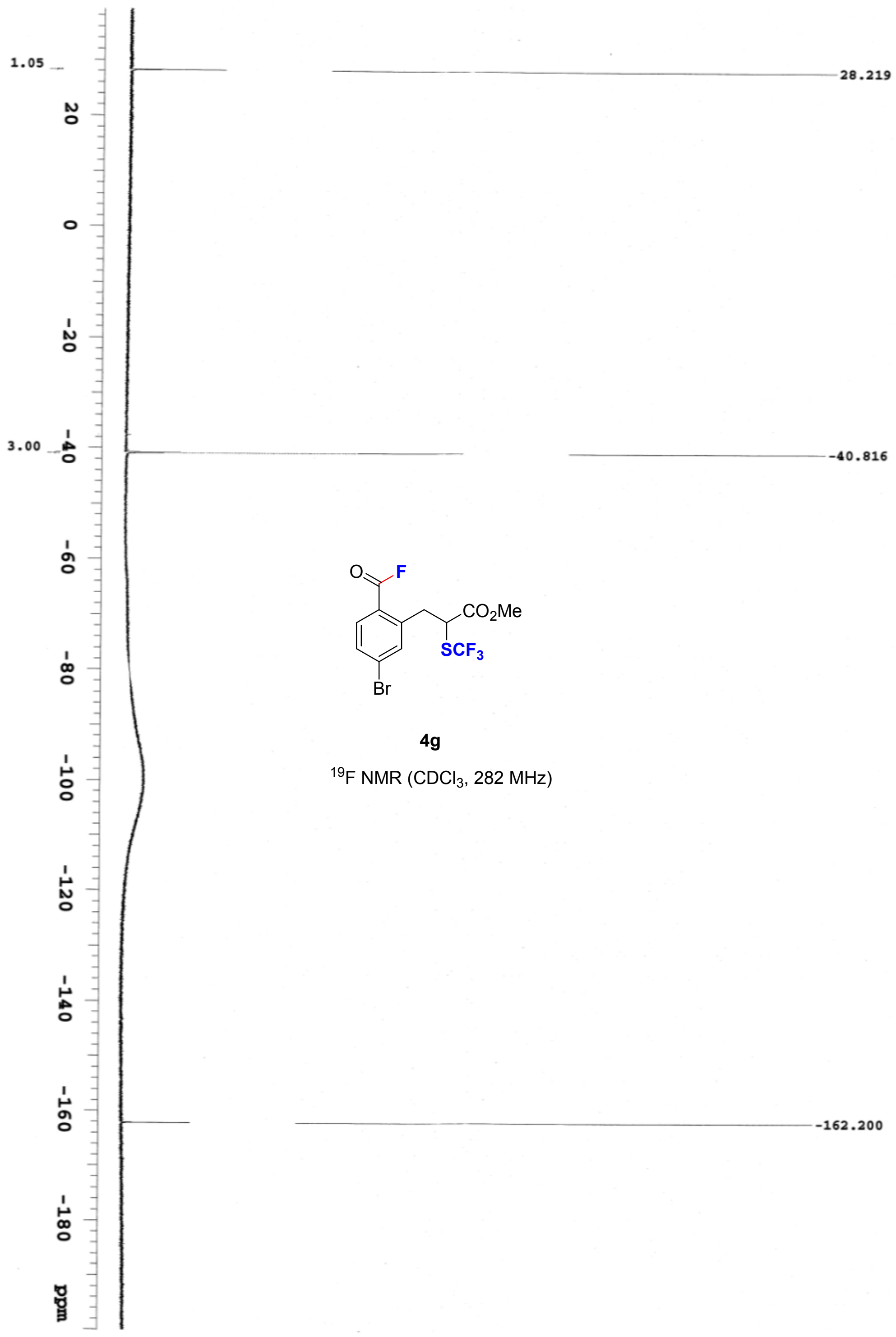

4g

^{19}F NMR ($CDCl_3$, 282 MHz)

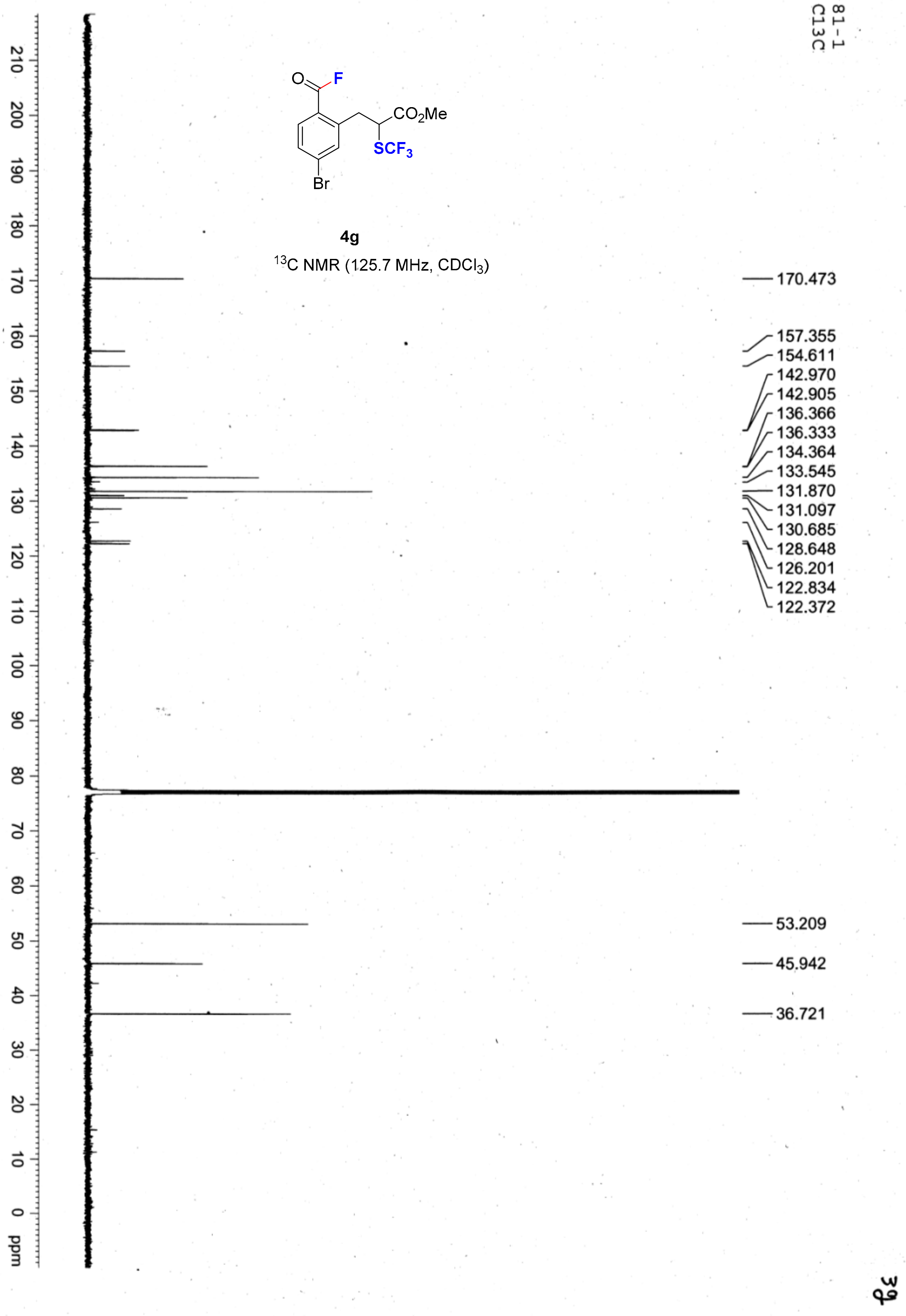

4g

^{13}C NMR (125.7 MHz, $CDCl_3$)

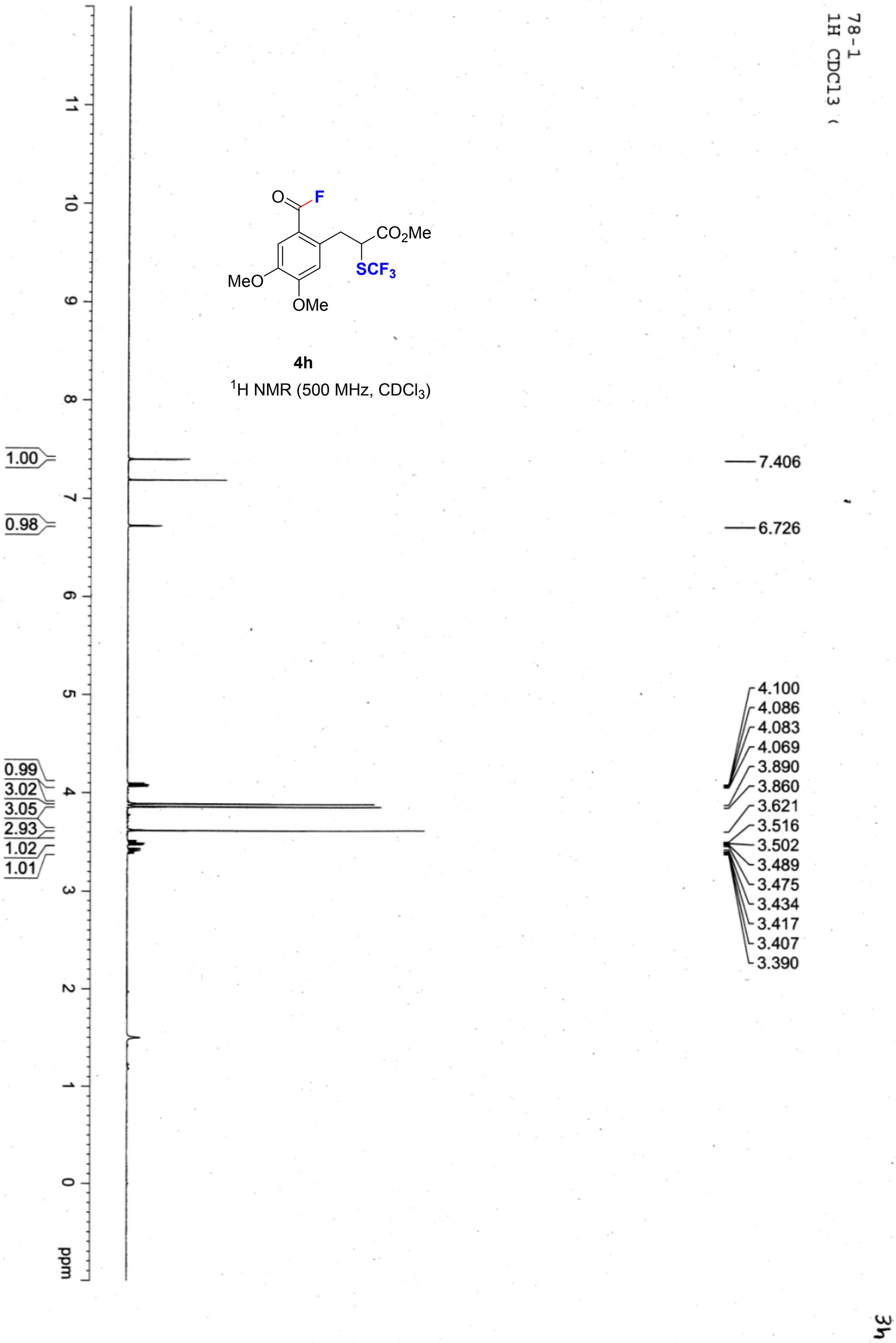

4h

^{1}H NMR (500 MHz, $CDCl_3$)

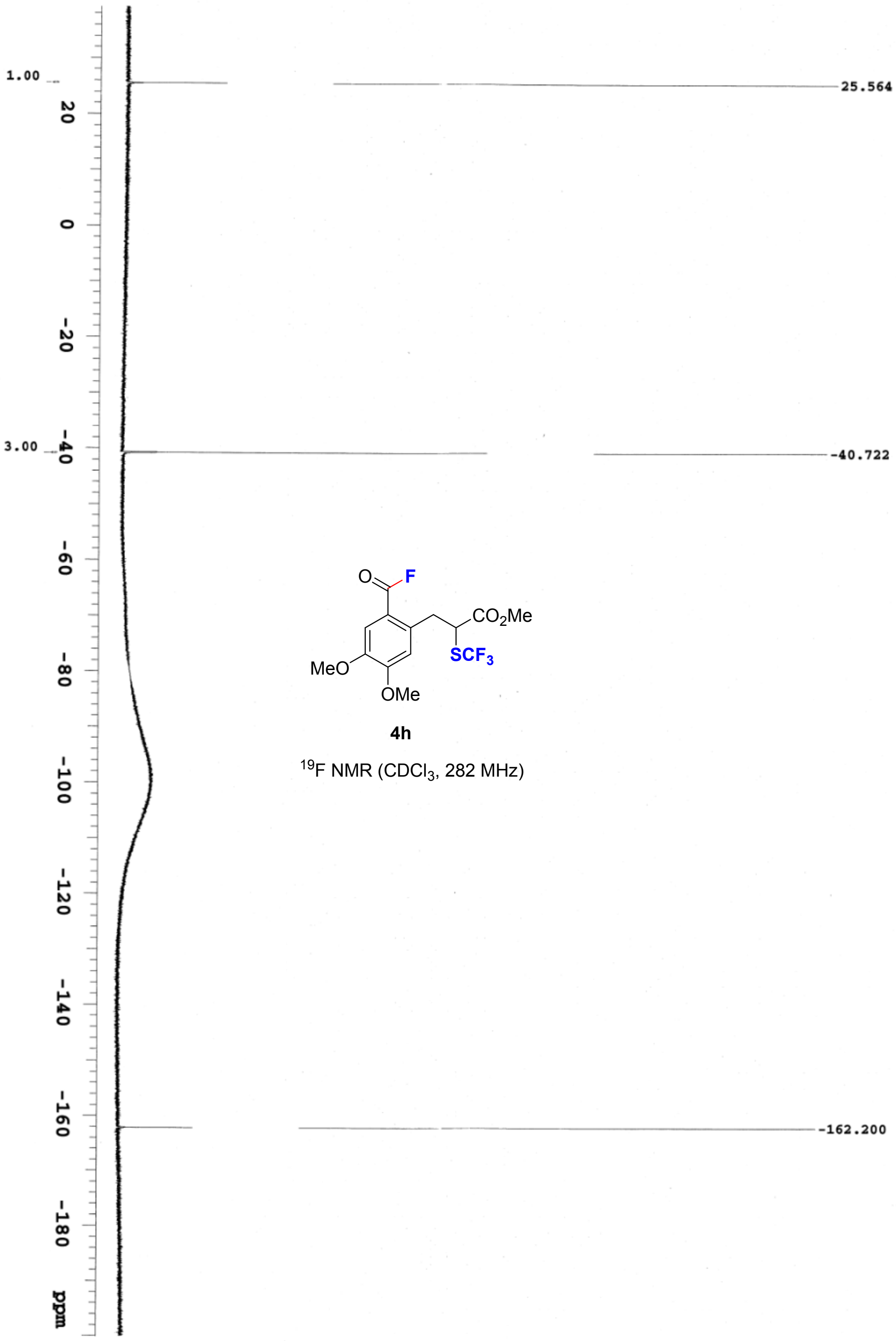

4h

^{19}F NMR ($CDCl_3$, 282 MHz)

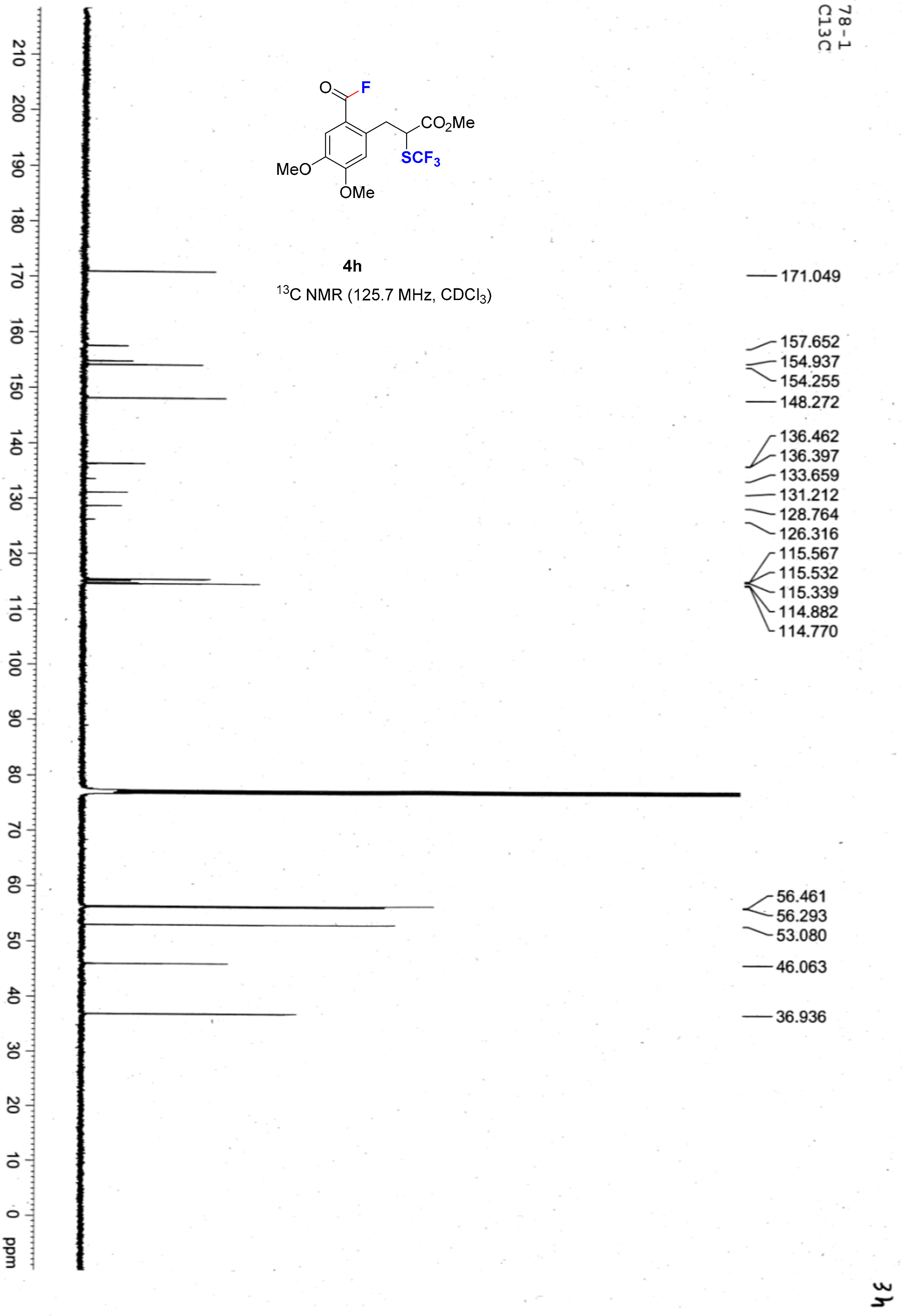

4h

^{13}C NMR (125.7 MHz, $CDCl_3$)

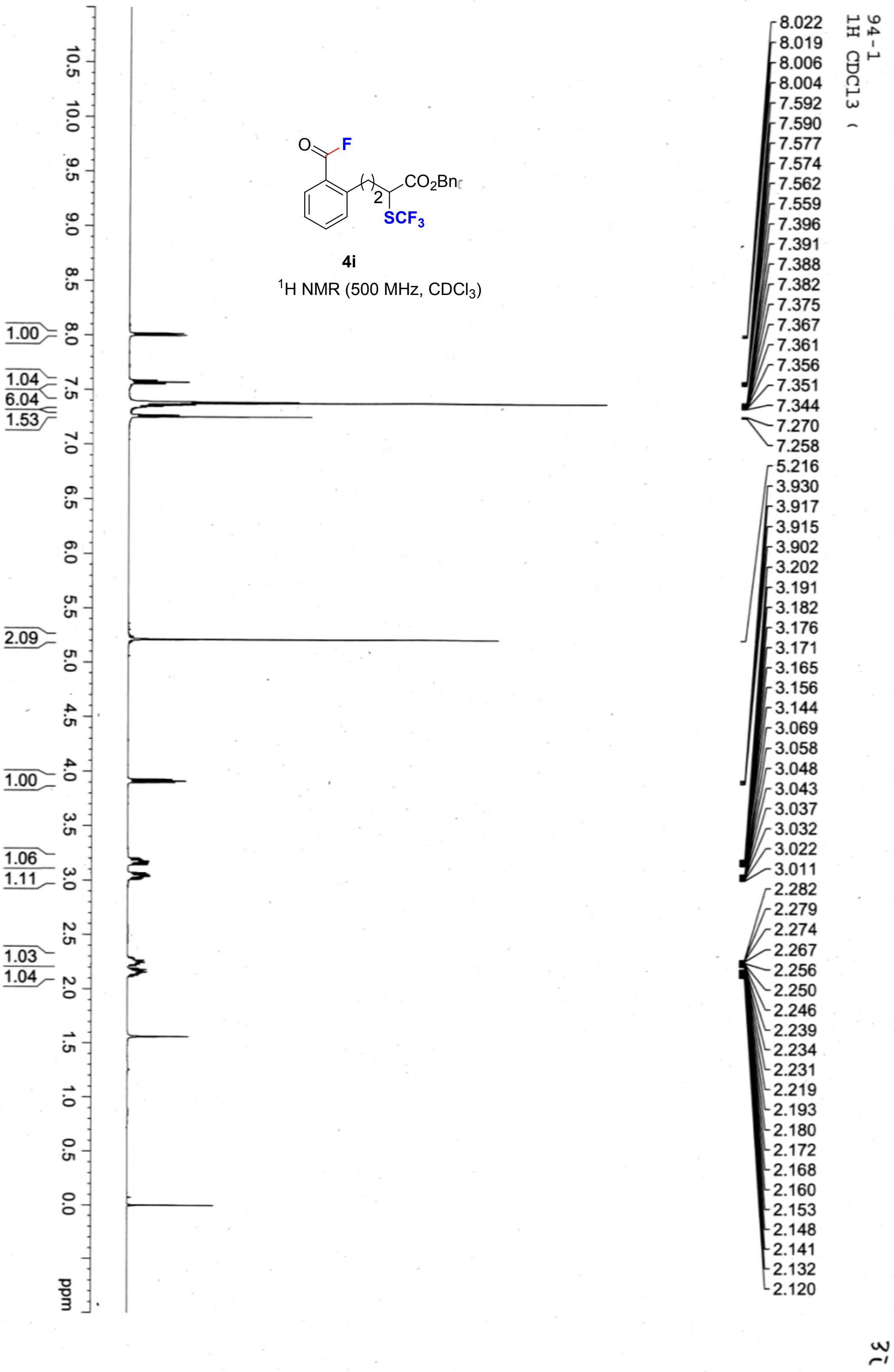
O
F
CO2Bn
2
SCF3
4i
1H NMR (500 MHz, CDCl3)
94-1
1H CDCl3
8.022
8.019
8.006
8.004
7.592
7.590
7.577
7.574
7.562
7.559
7.396
7.391
7.388
7.382
7.375
7.367
7.361
7.356
7.351
7.344
7.270
7.258
5.216
3.930
3.917
3.915
3.902
3.202
3.191
3.182
3.176
3.171
3.165
3.156
3.144
3.069
3.058
3.048
3.043
3.037
3.032
3.022
3.011
2.282
2.279
2.274
2.267
2.256
2.250
2.246
2.239
2.234
2.231
2.219
2.193
2.180
2.172
2.168
2.160
2.153
2.148
2.141
2.132
2.120
1.00
1.04
6.04
1.53
2.09
1.00
1.06
1.11
1.03
1.04
ppm

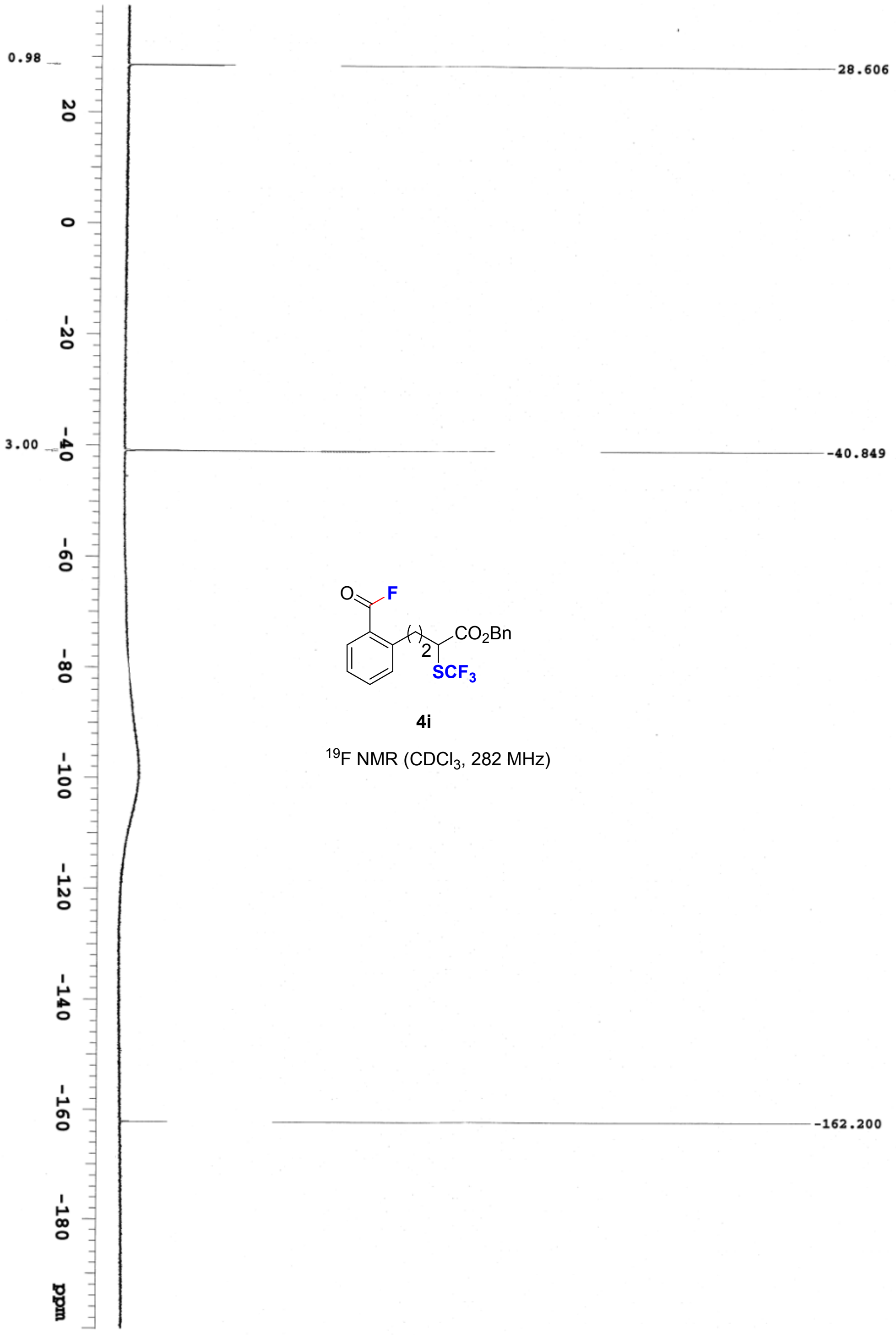

4i

^{19}F NMR ($CDCl_3$, 282 MHz)

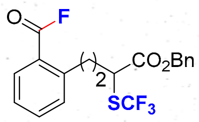

4i

^{13}C NMR (125.7 MHz, $CDCl_3$)

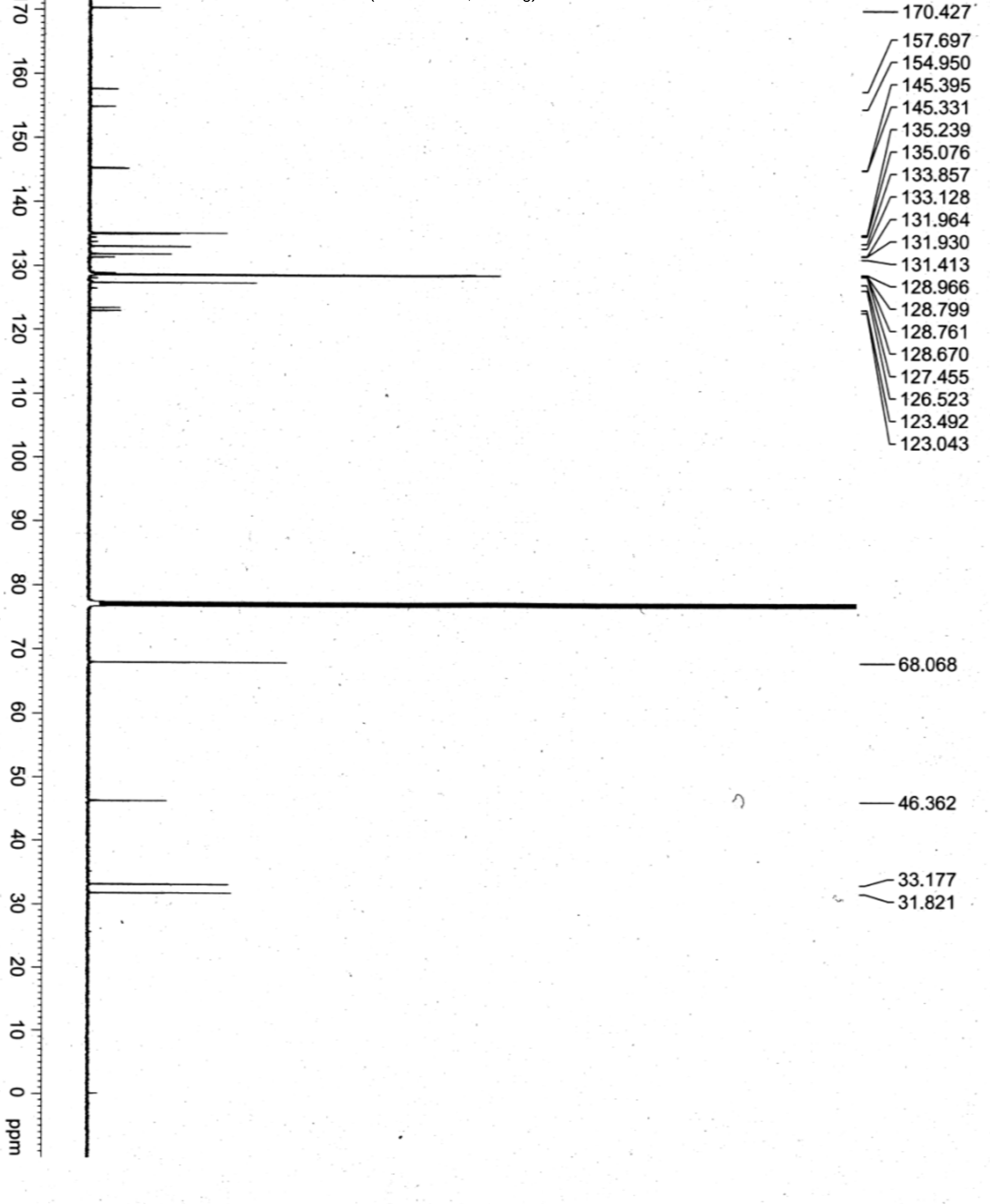

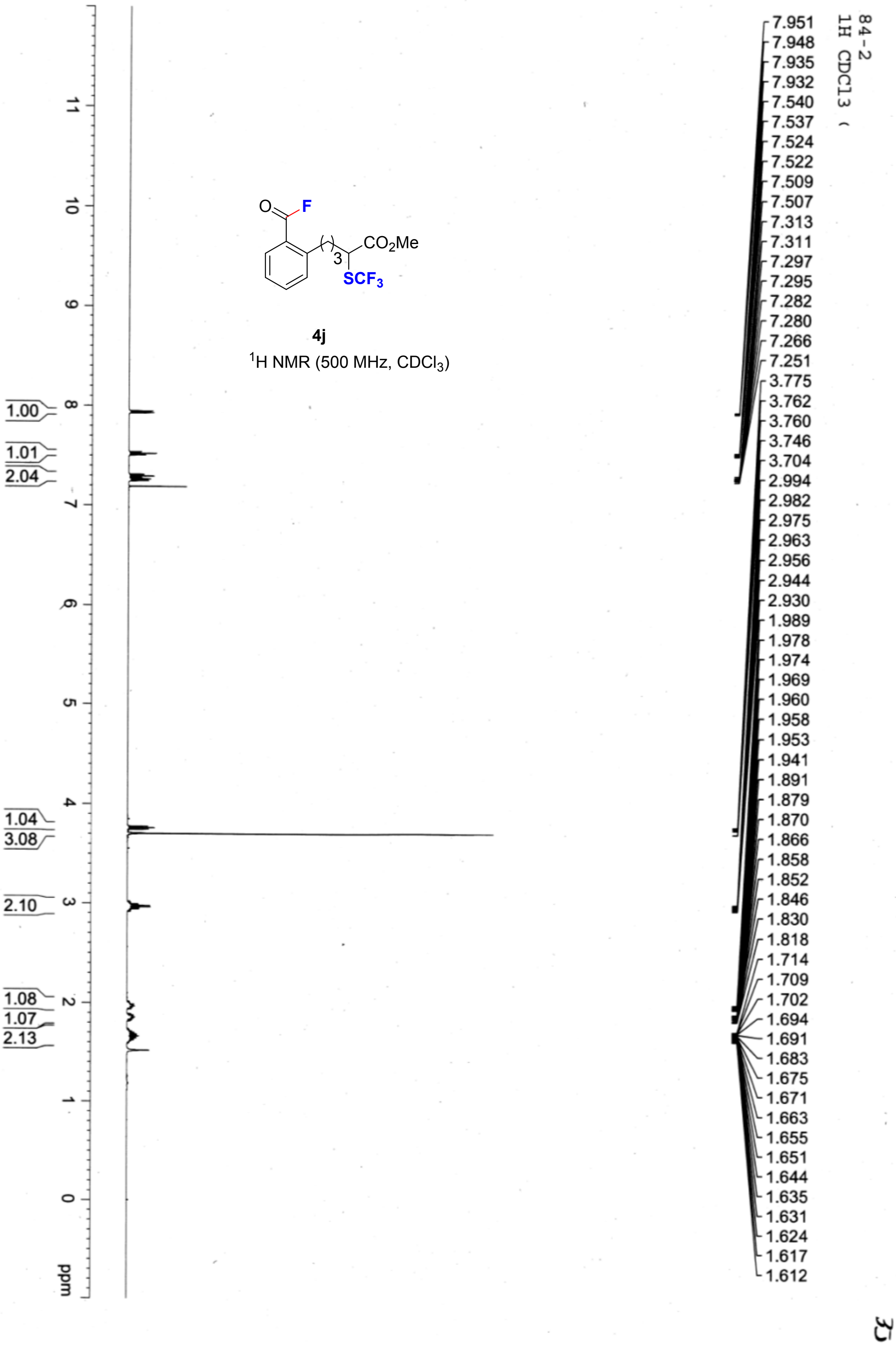

4j

^{1}H NMR (500 MHz, $CDCl_3$)

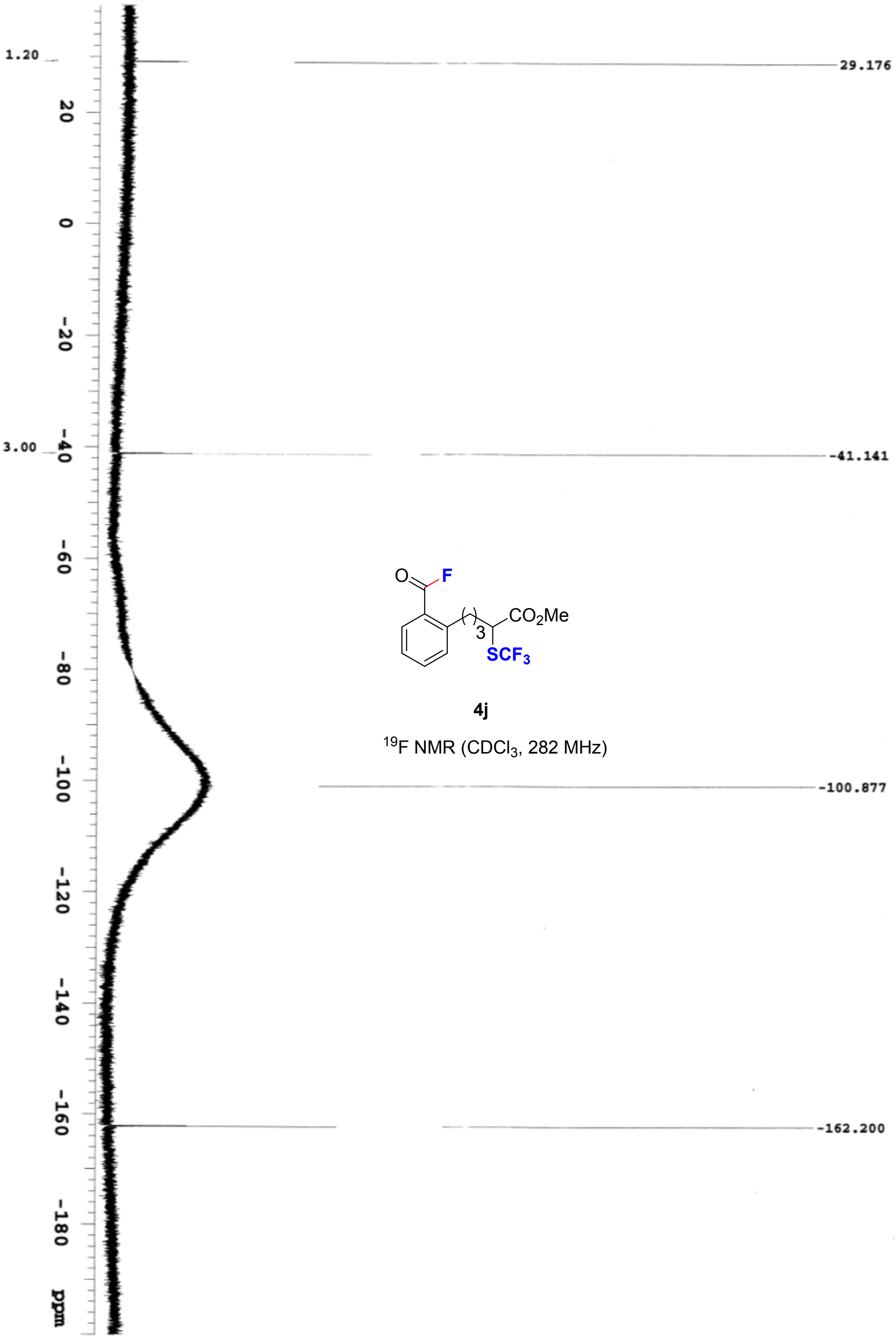

4j

^{19}F NMR ($CDCl_3$, 282 MHz)

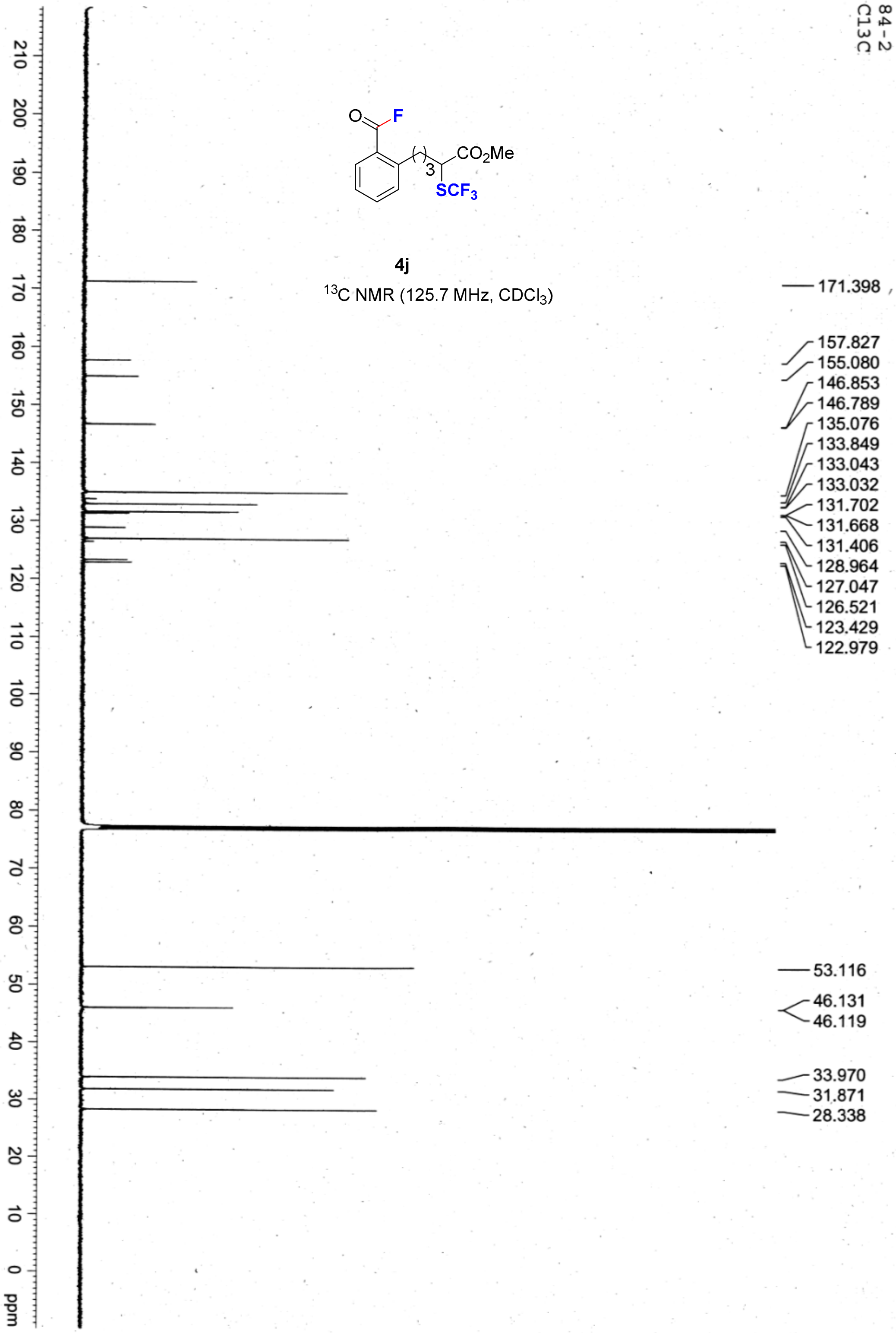

4j

^{13}C NMR (125.7 MHz, $CDCl_3$)

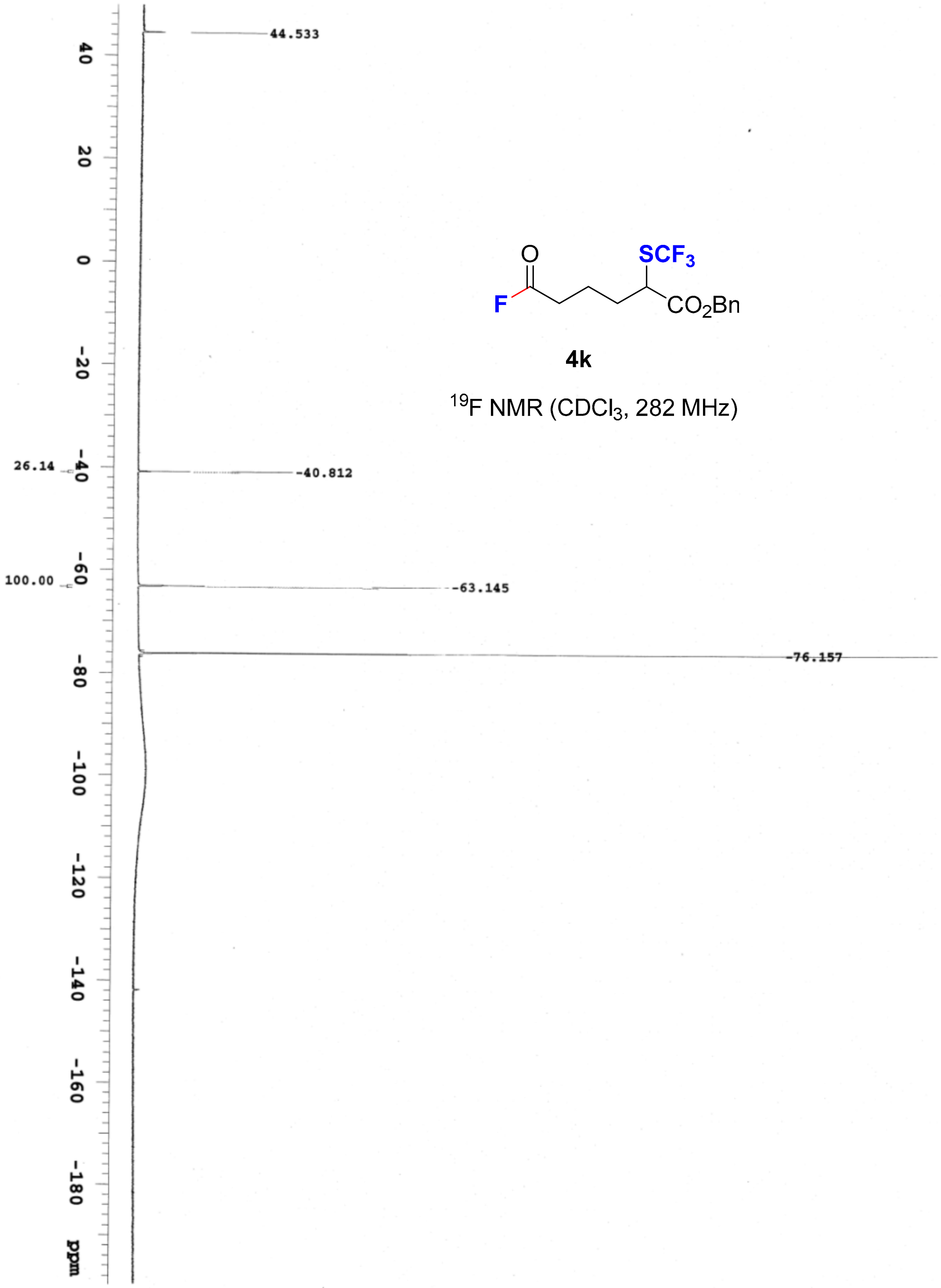

4k

^{19}F NMR ($CDCl_3$, 282 MHz)

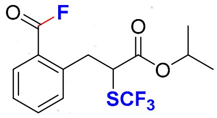

4l

^{1}H NMR (500 MHz, CDCl$_3$)

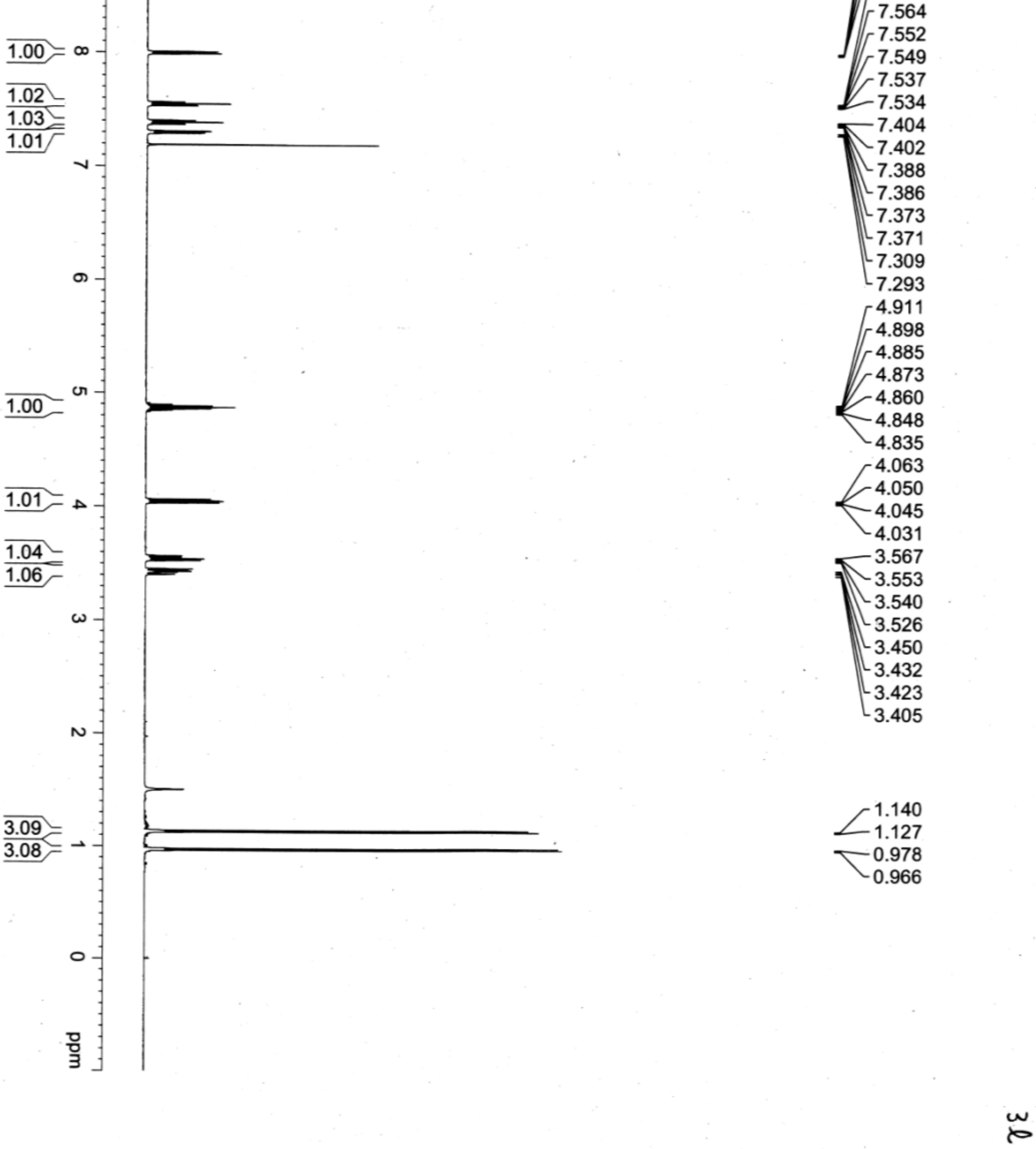

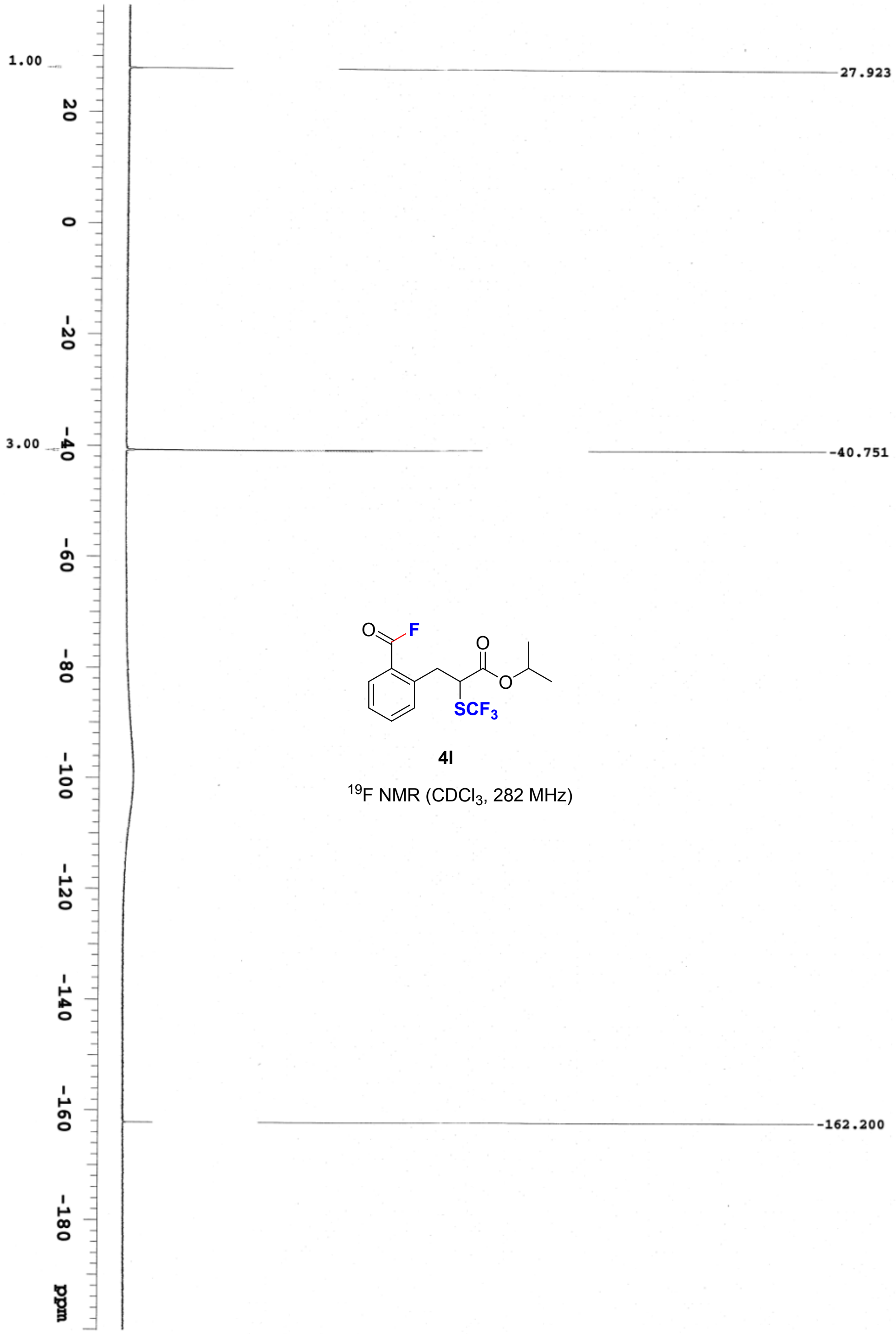

4l

^{19}F NMR ($CDCl_3$, 282 MHz)

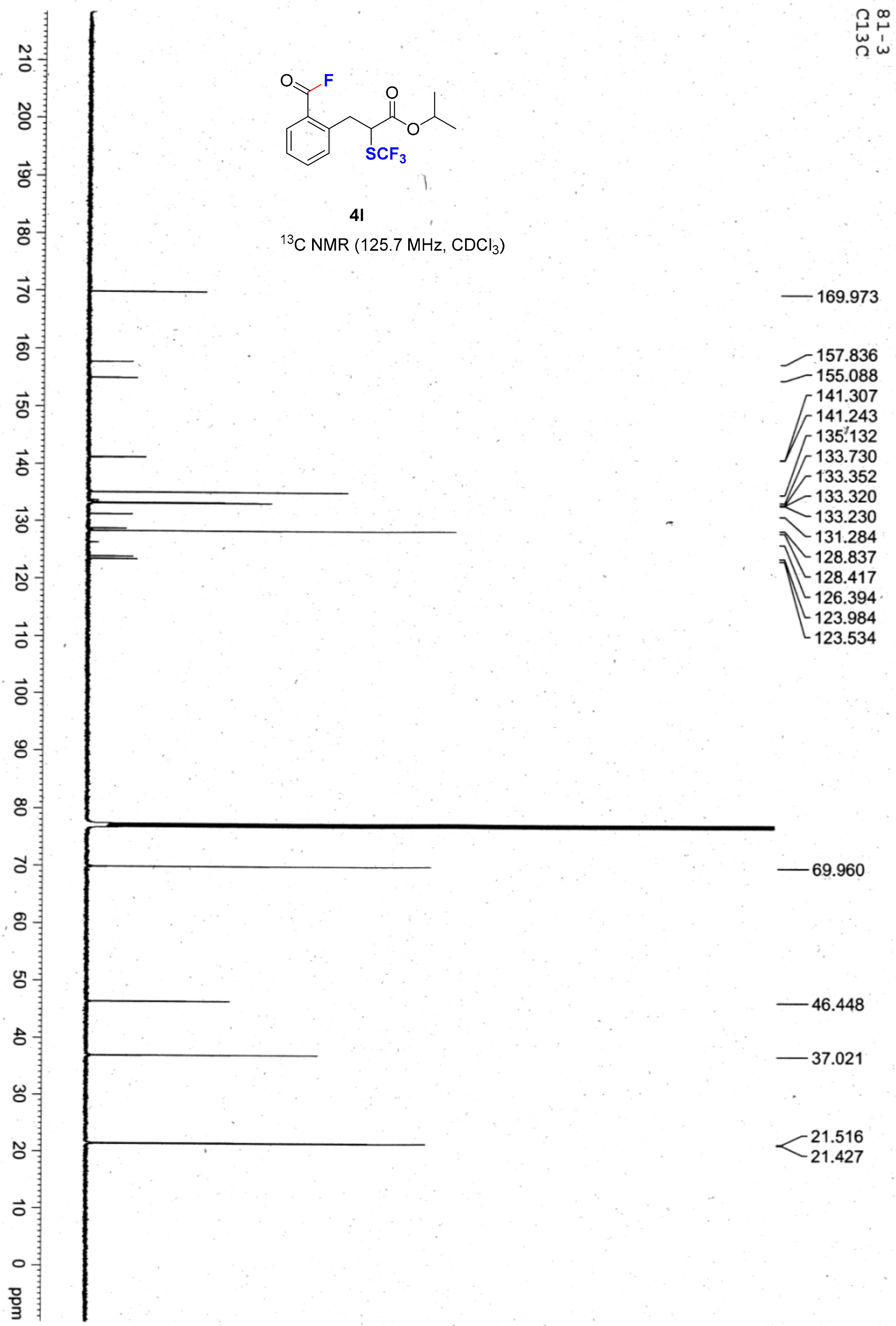

4l

^{13}C NMR (125.7 MHz, $CDCl_3$)

3l

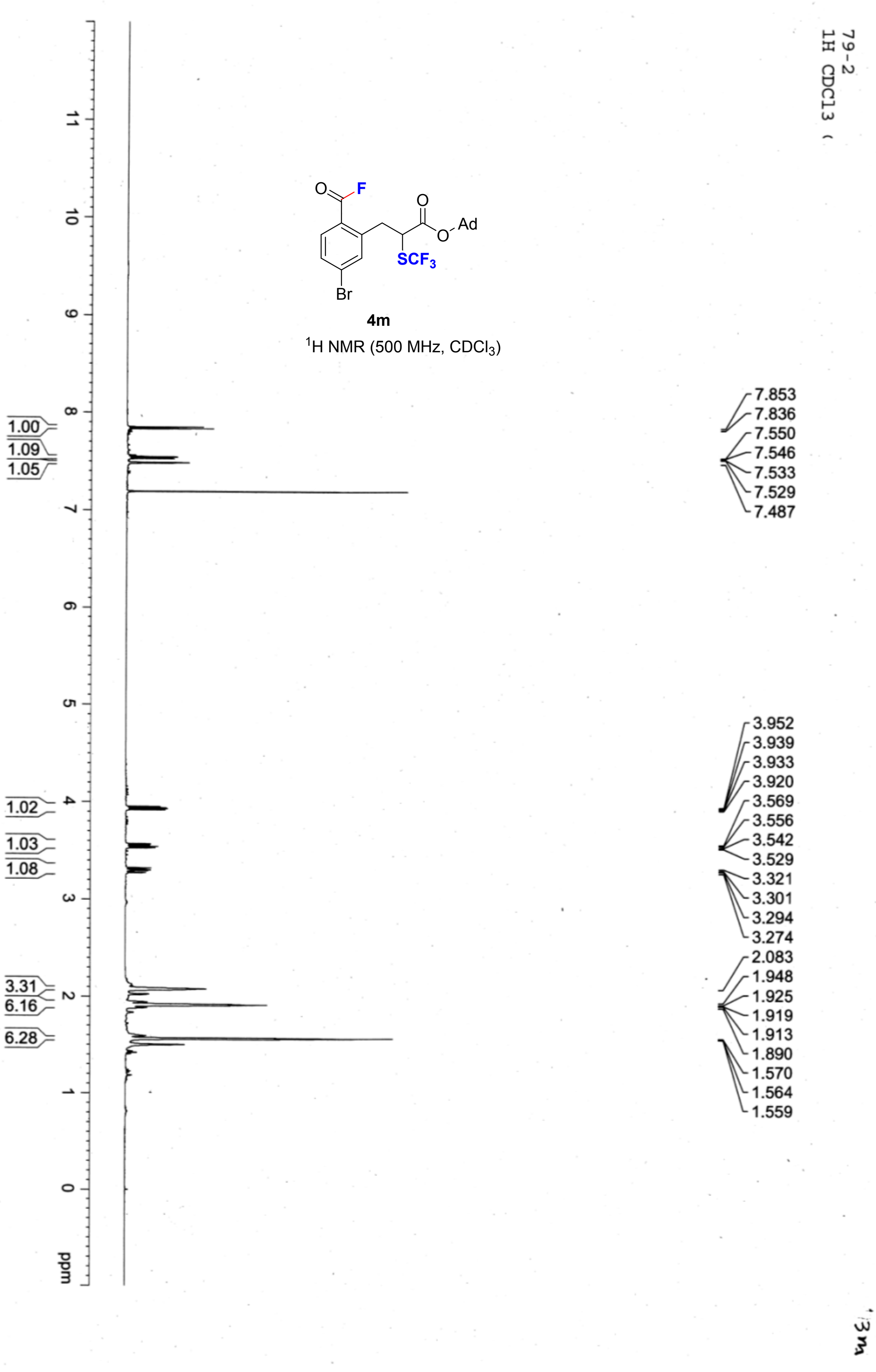

4m

^{1}H NMR (500 MHz, CDCl$_3$)

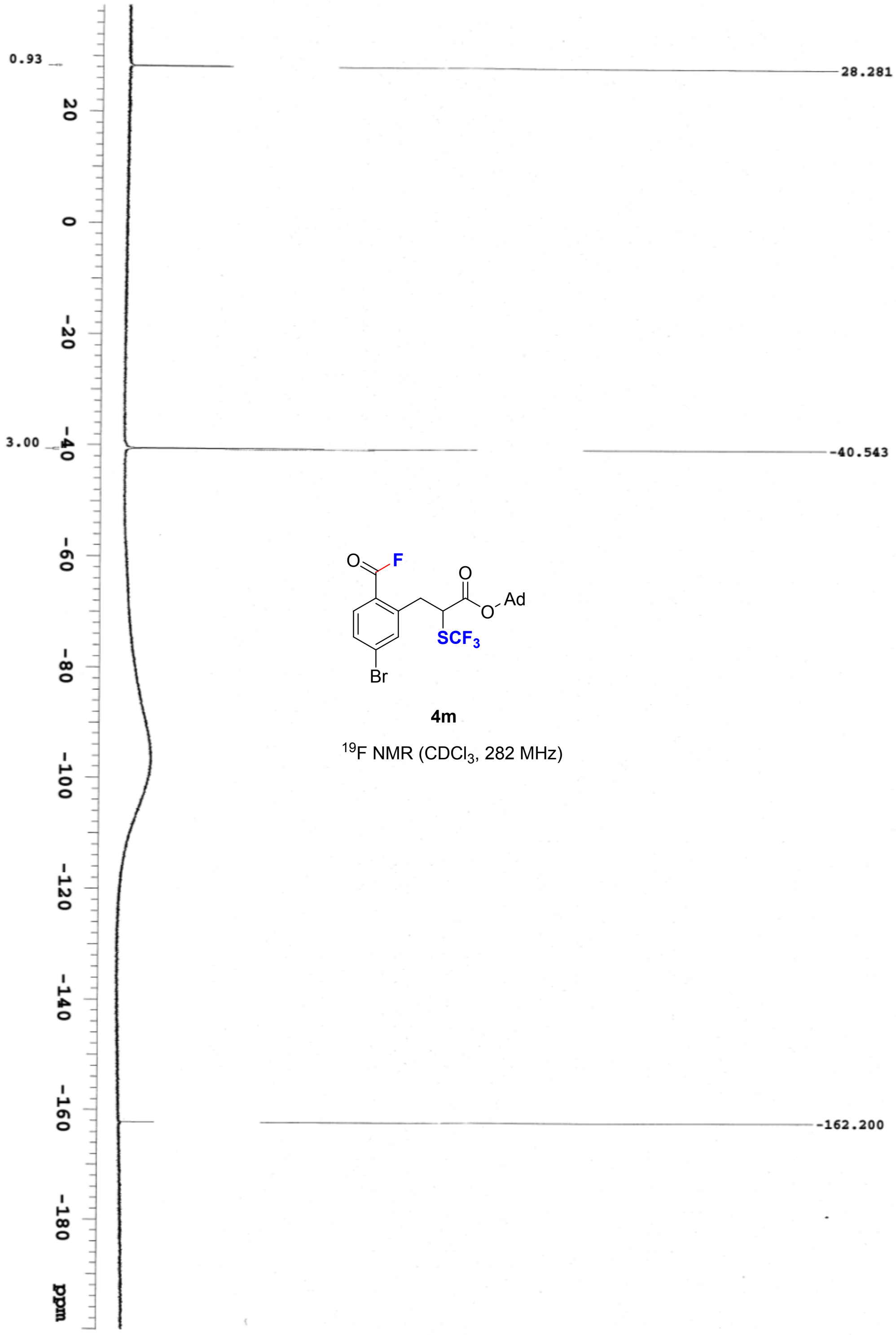

4m

^{19}F NMR ($CDCl_3$, 282 MHz)

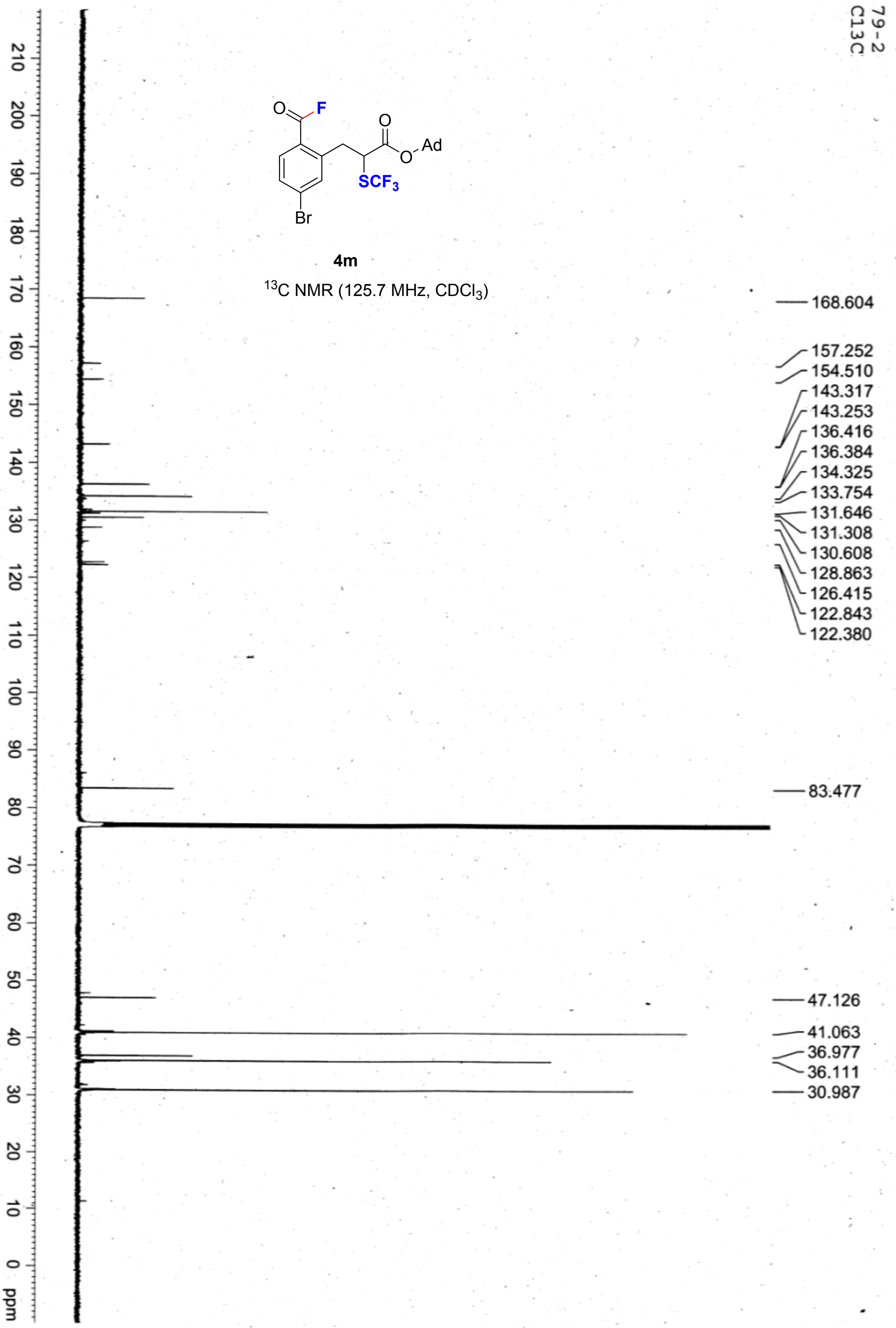

4m
^{13}C NMR (125.7 MHz, CDCl$_3$)
168.604
157.252
154.510
143.317
143.253
136.416
136.384
134.325
133.754
131.646
131.308
130.608
128.863
126.415
122.843
122.380
83.477
47.126
41.063
36.977
36.111
30.987
ppm

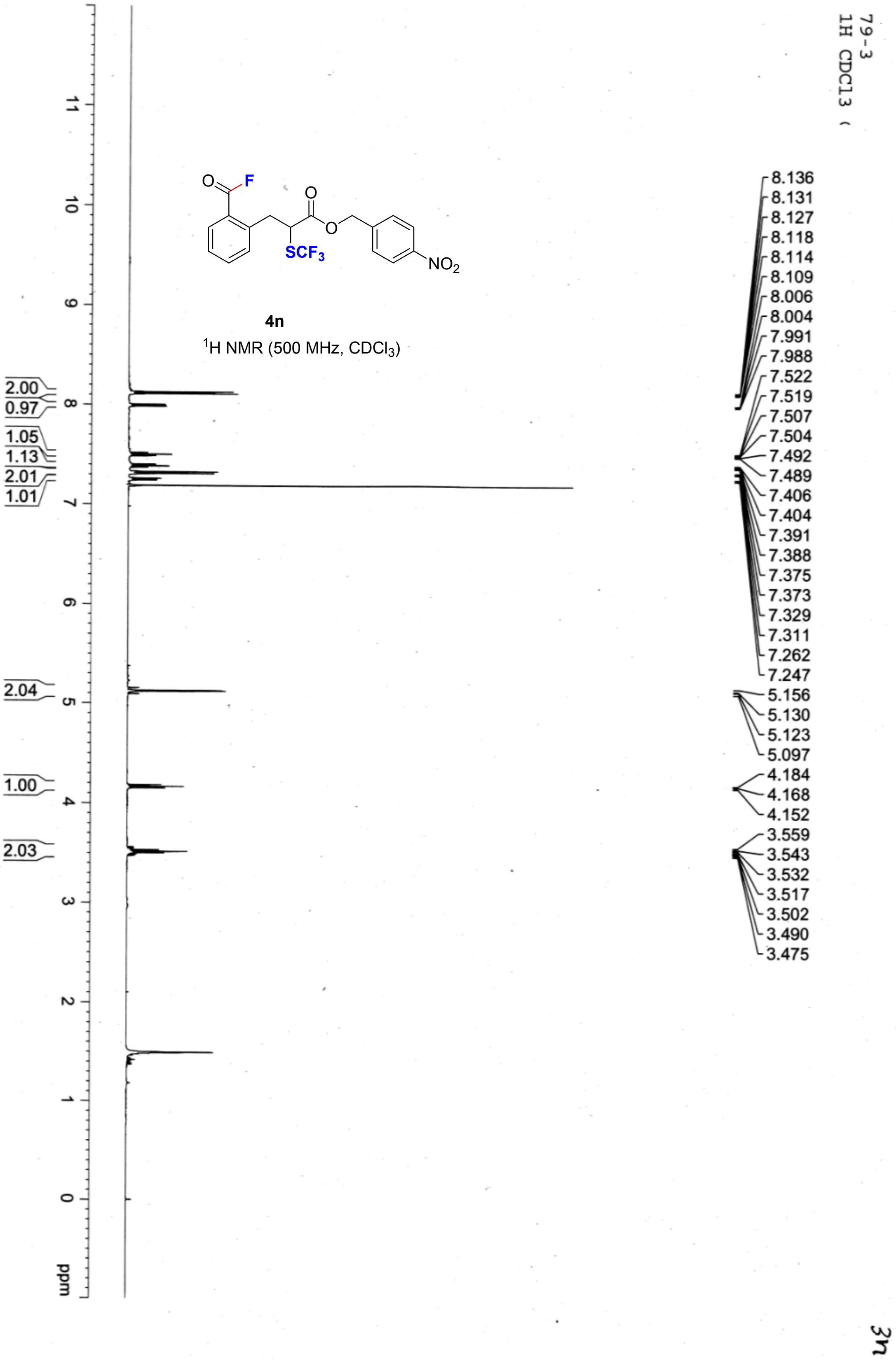

4n

^{1}H NMR (500 MHz, $CDCl_3$)

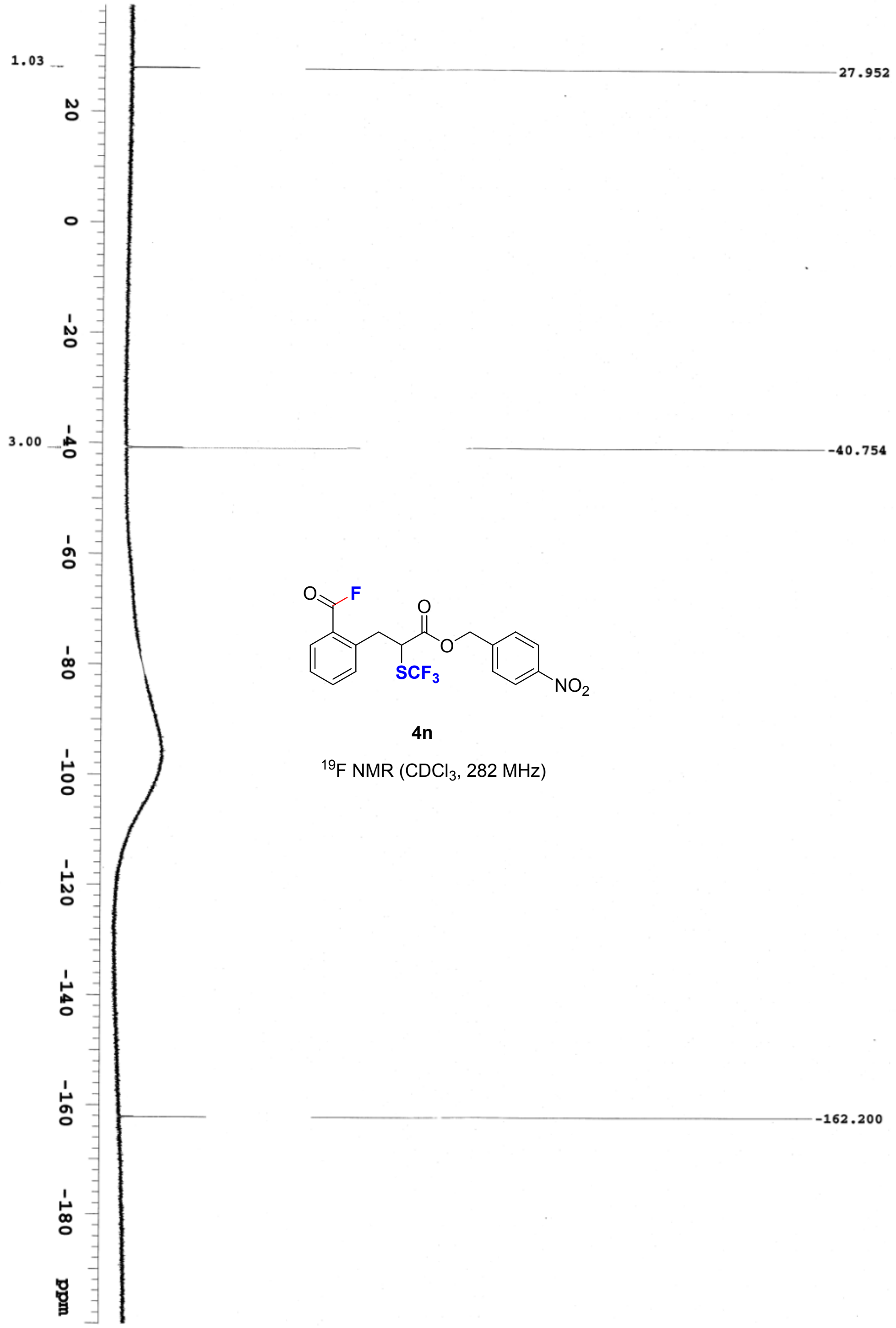

4n

^{19}F NMR ($CDCl_3$, 282 MHz)

3n

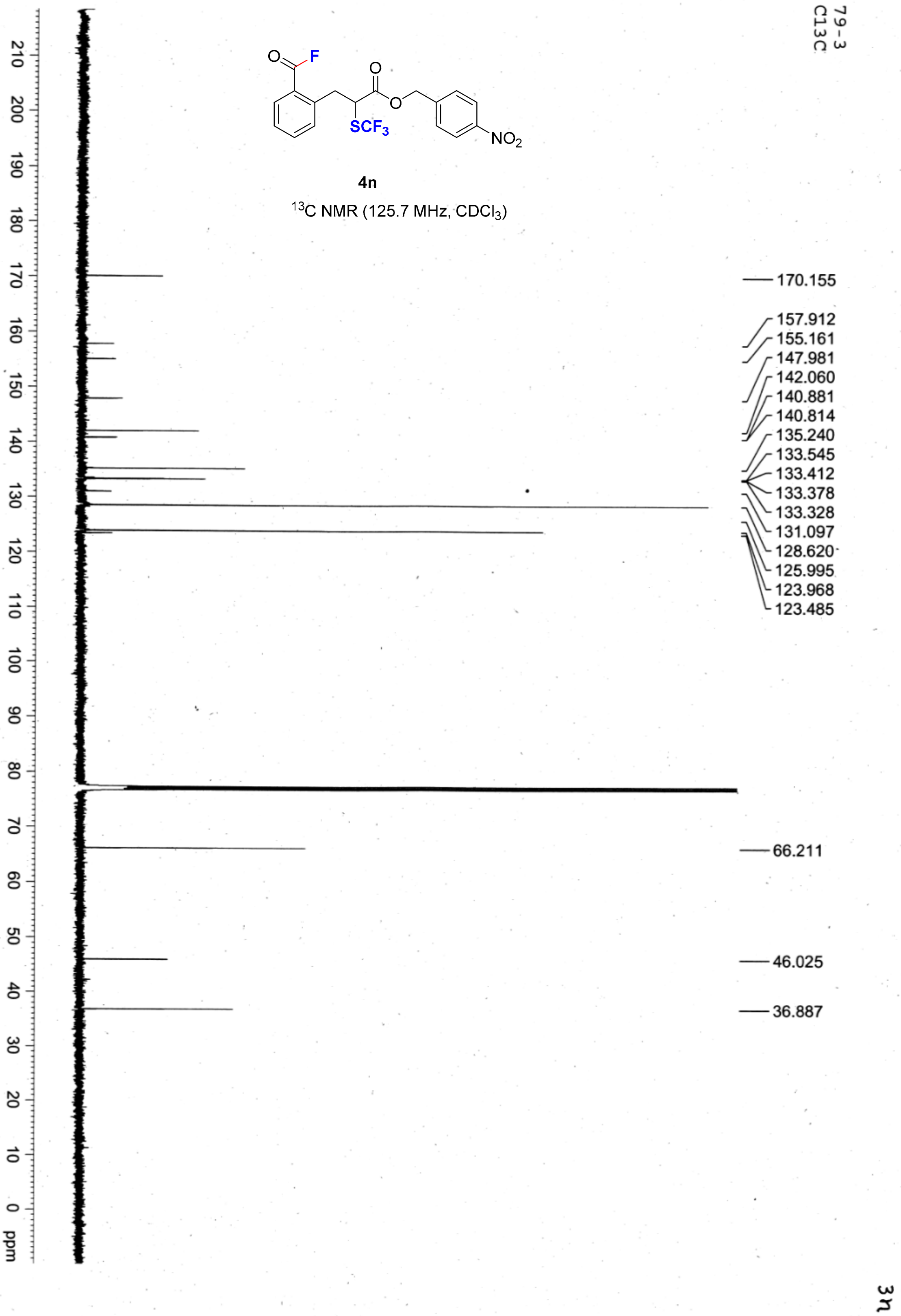

4n

^{13}C NMR (125.7 MHz, $CDCl_3$)

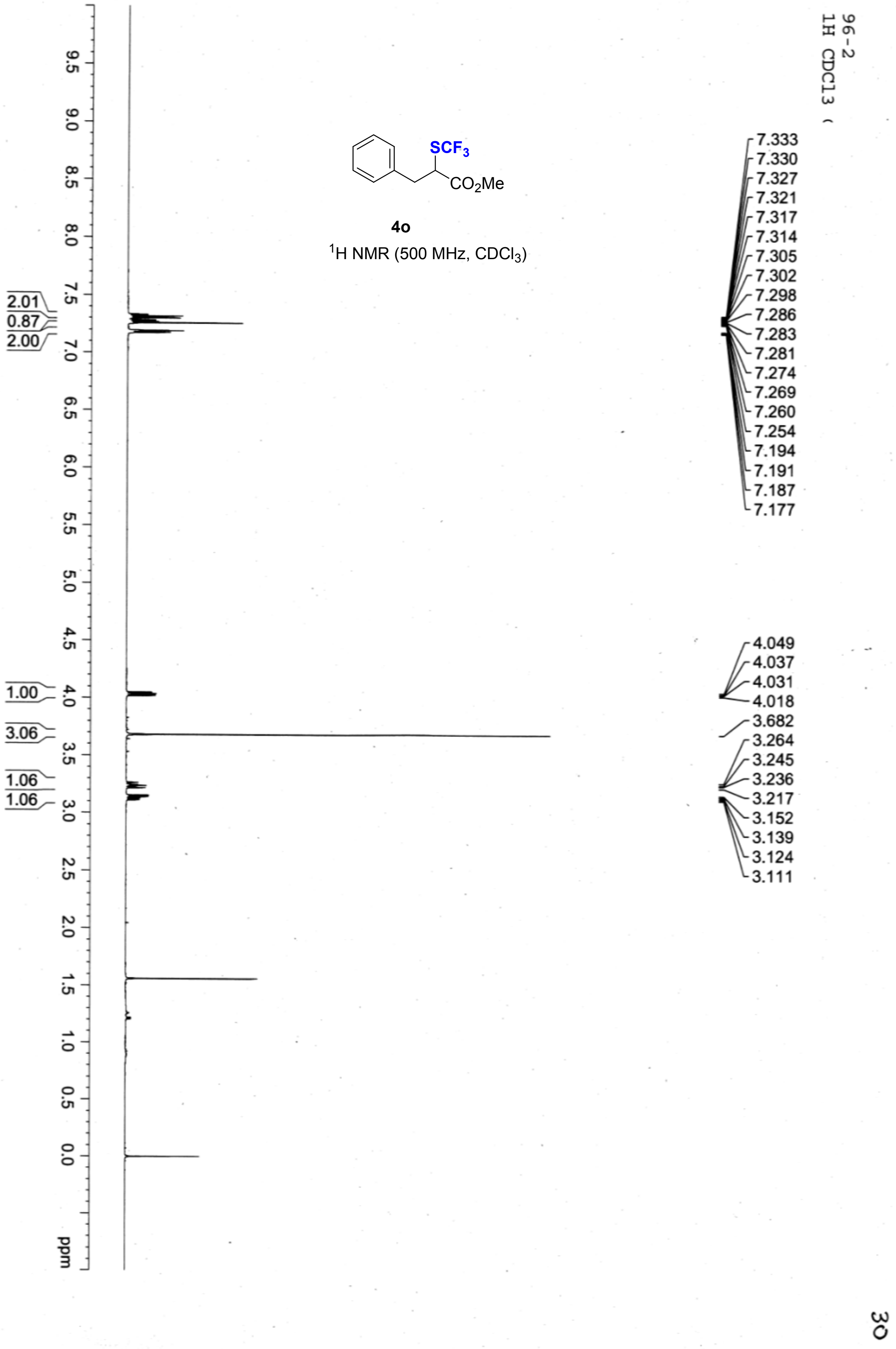
96-2
1H CDCl3
SCF3
CO2Me
4o
1H NMR (500 MHz, CDCl3)
7.333
7.330
7.327
7.321
7.317
7.314
7.305
7.302
7.298
7.286
7.283
7.281
7.274
7.269
7.260
7.254
7.194
7.191
7.187
7.177
4.049
4.037
4.031
4.018
3.682
3.264
3.245
3.236
3.217
3.152
3.139
3.124
3.111
2.01
0.87
2.00
1.00
3.06
1.06
1.06
ppm
30

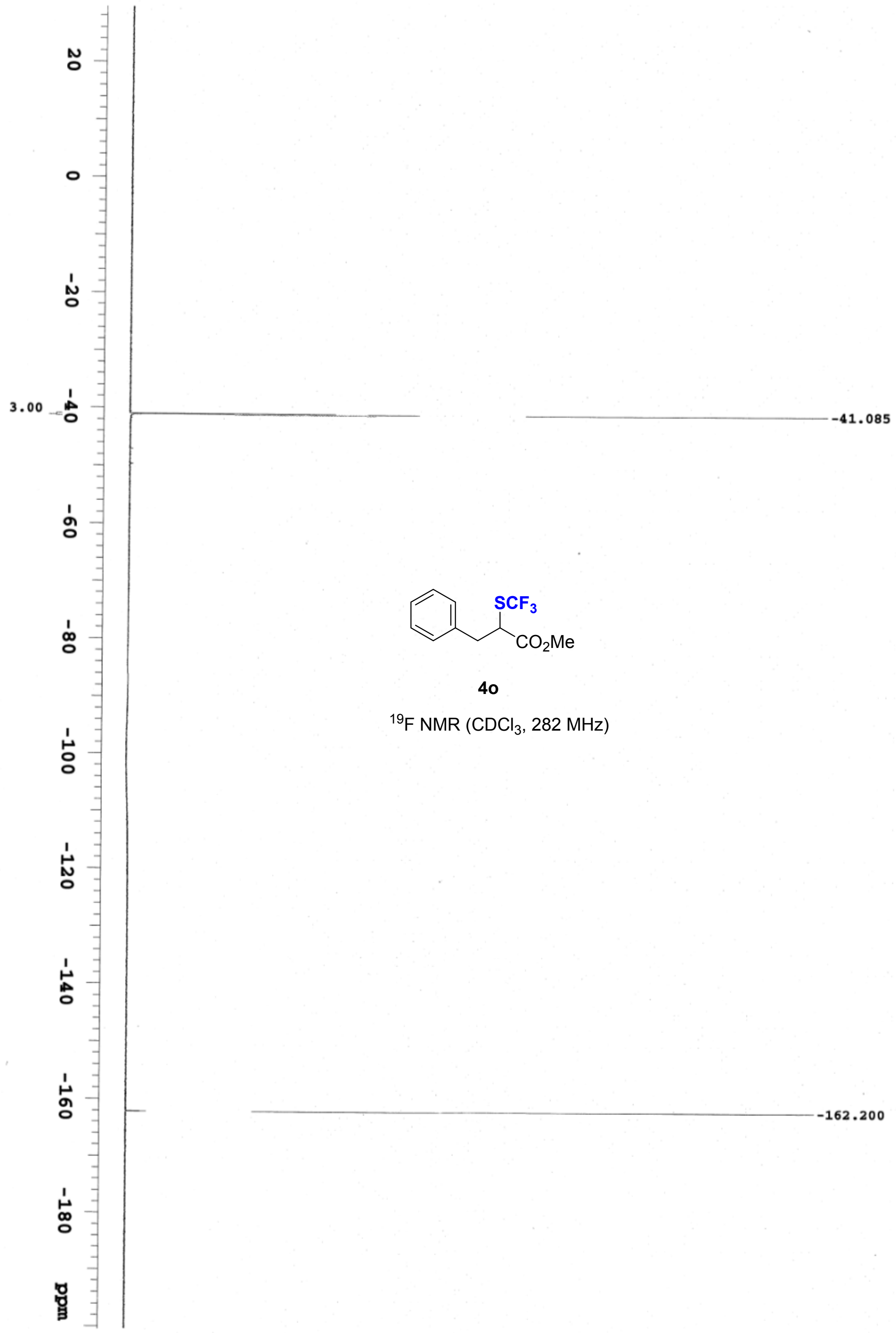

4o

^{19}F NMR ($CDCl_3$, 282 MHz)

4o

^{13}C NMR (125.7 MHz, $CDCl_3$)

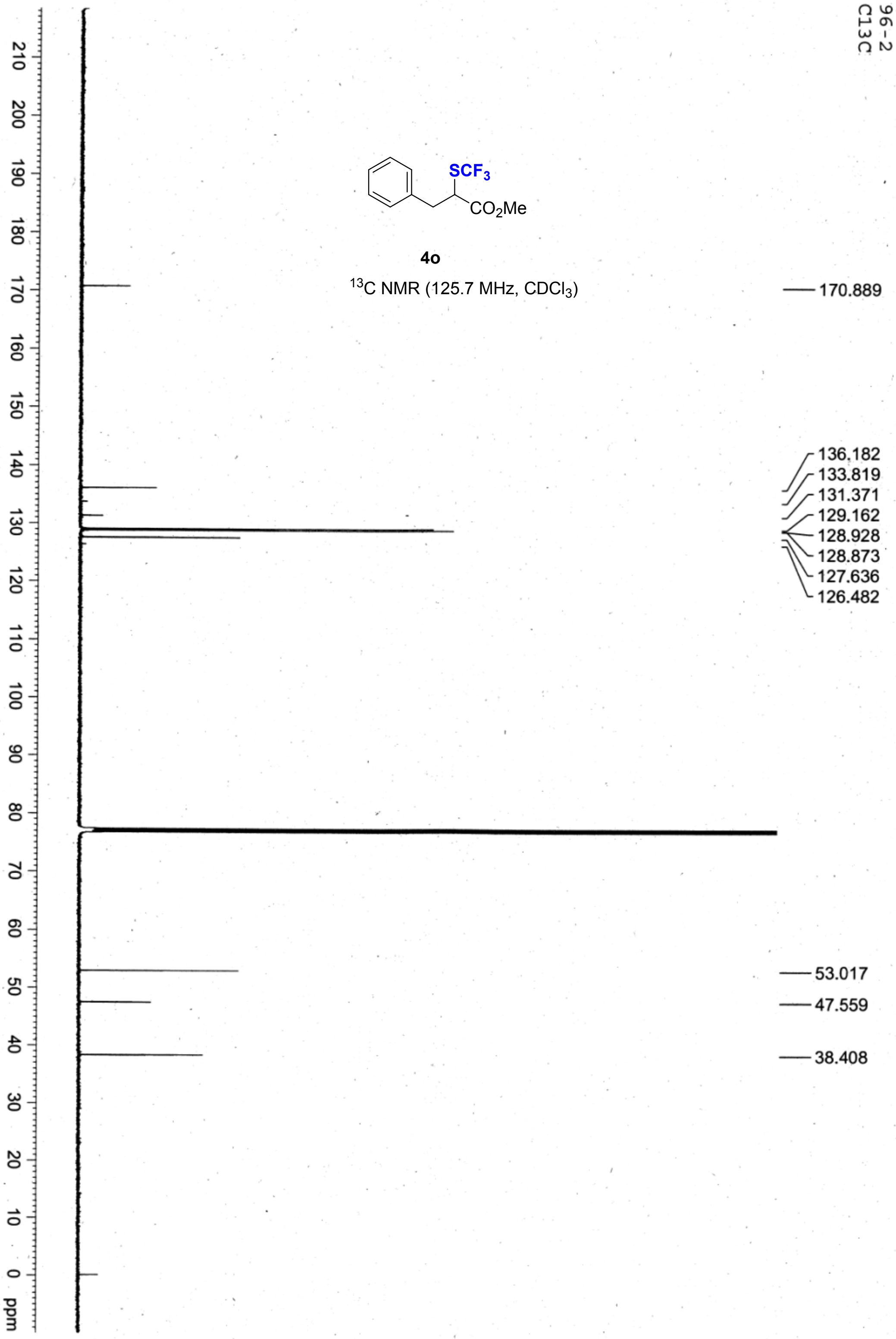

22. ^{1}H NMR, ^{13}C NMR and ^{19}F NMR spectra for transformation of acid fluorides 2b and 4b to ketones 5a, 6a amides 5b, 6b and ester 6c (Scheme 2);

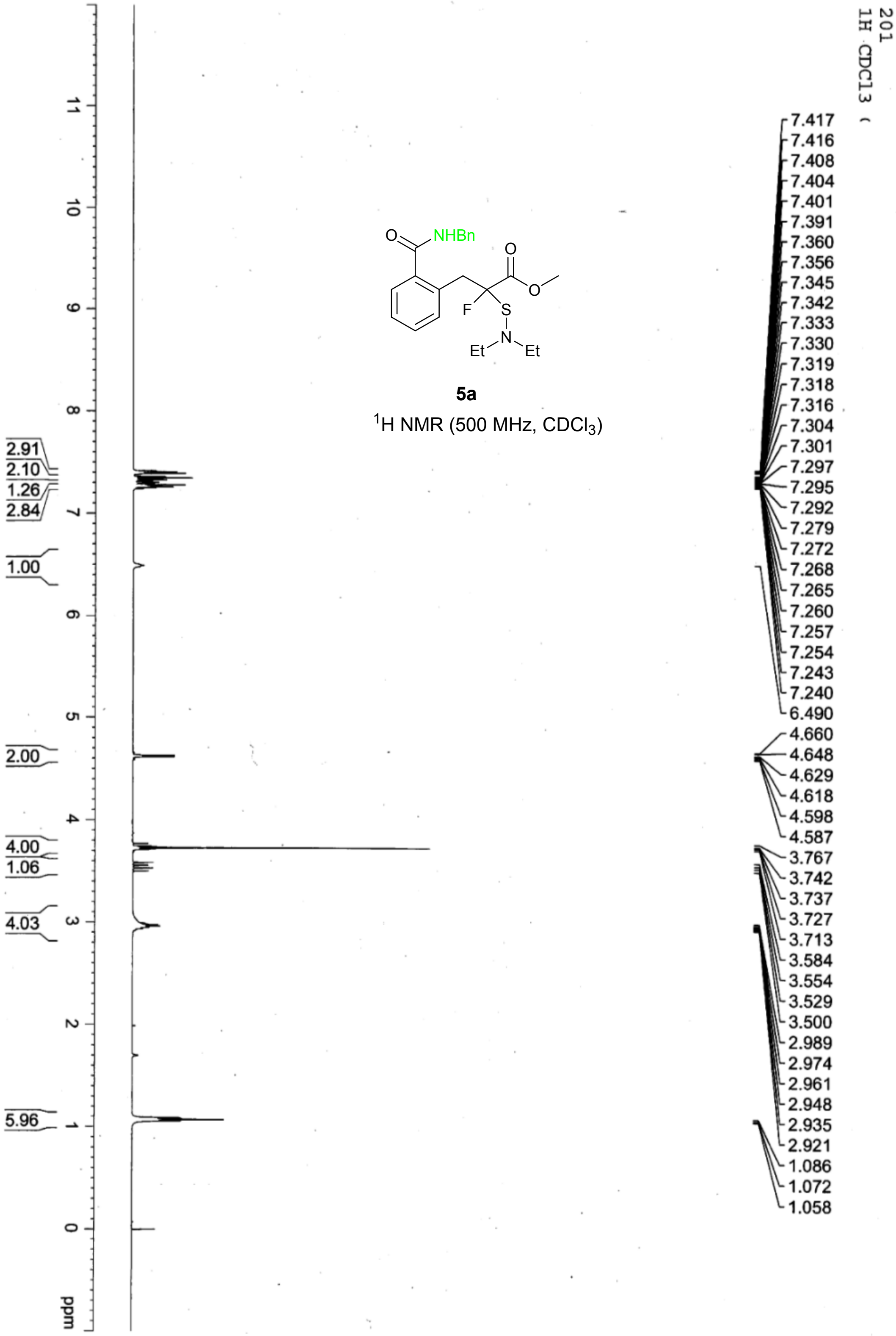

5a

^{1}H NMR (500 MHz, $CDCl_3$)

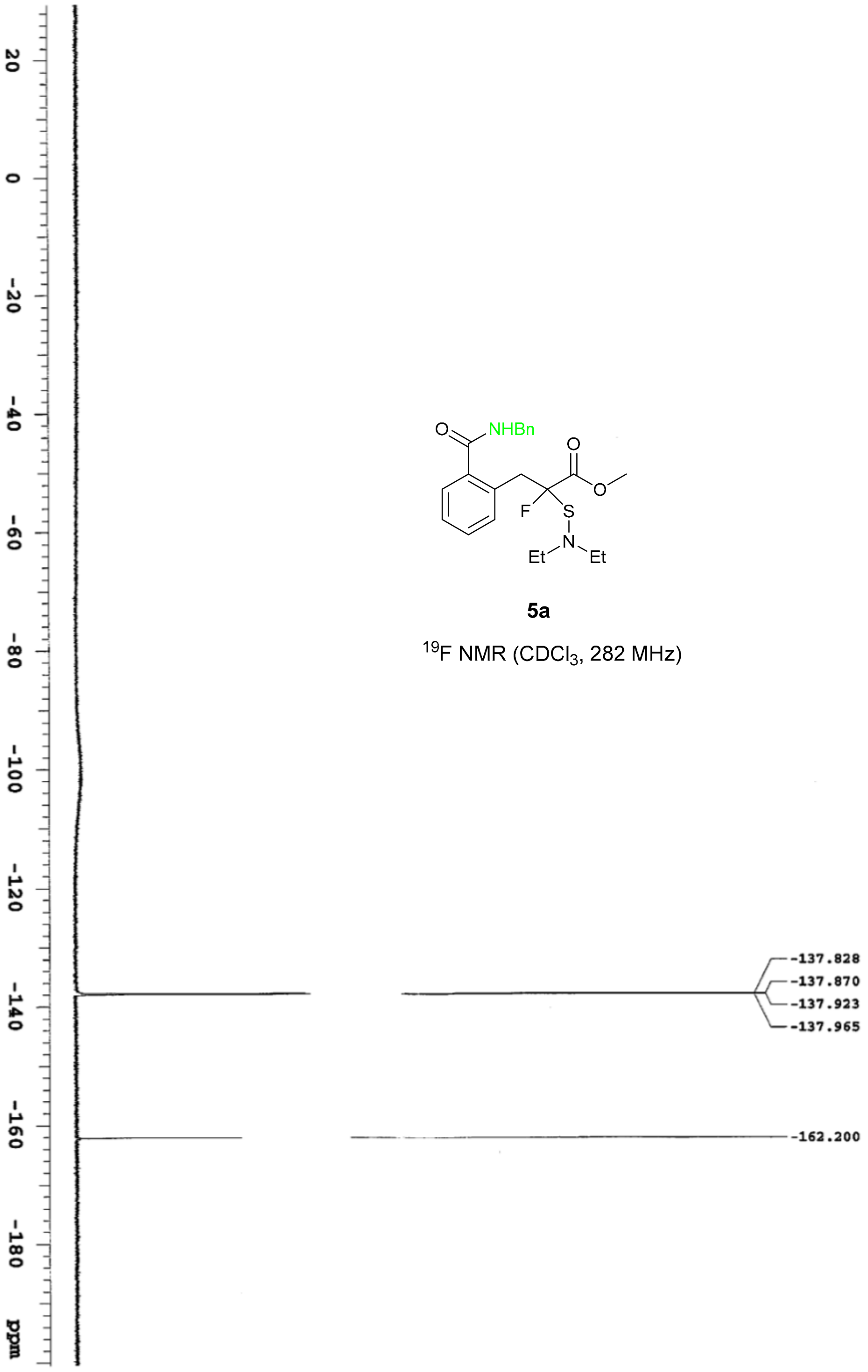

5a

^{19}F NMR ($CDCl_3$, 282 MHz)

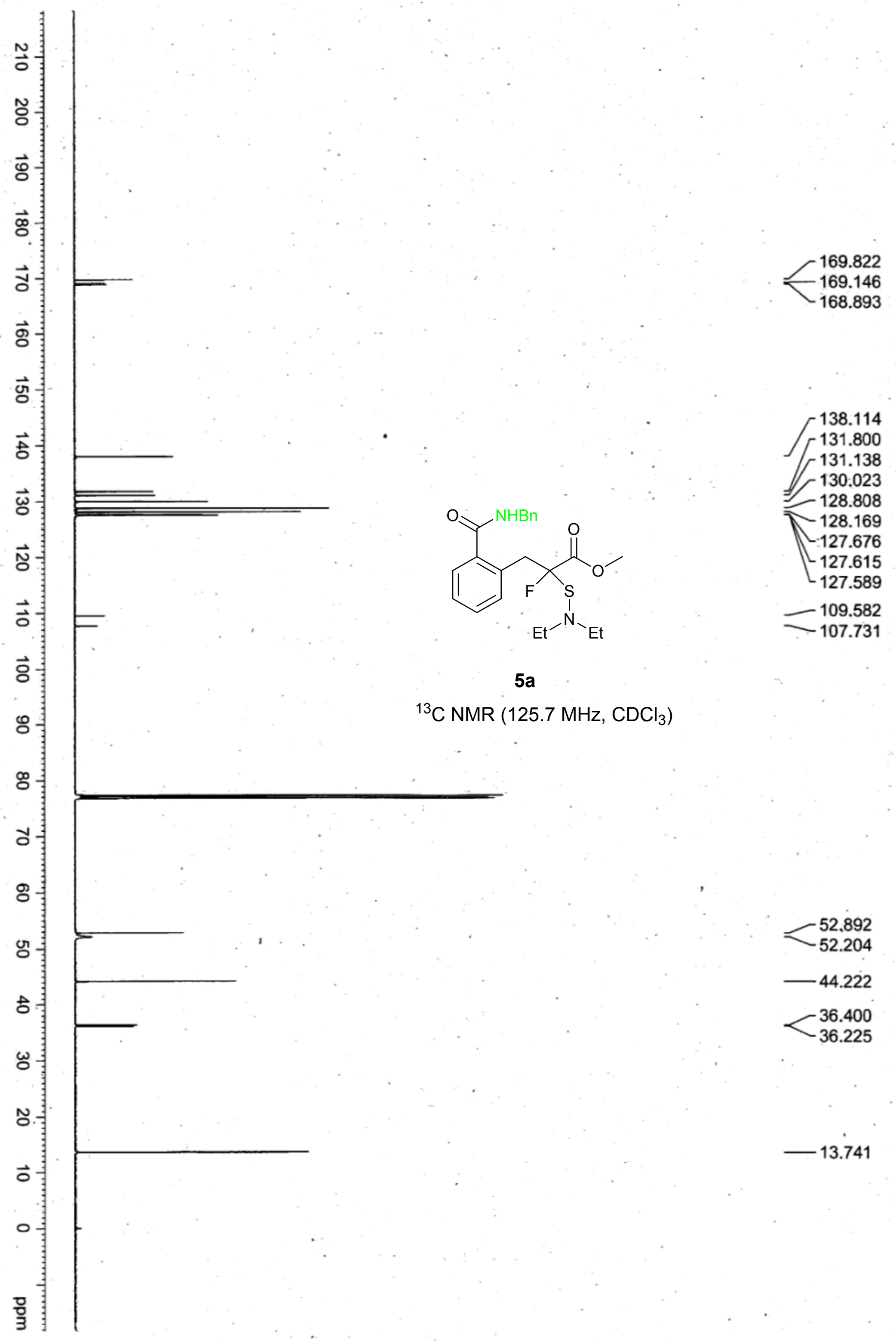

5a

^{13}C NMR (125.7 MHz, $CDCl_3$)

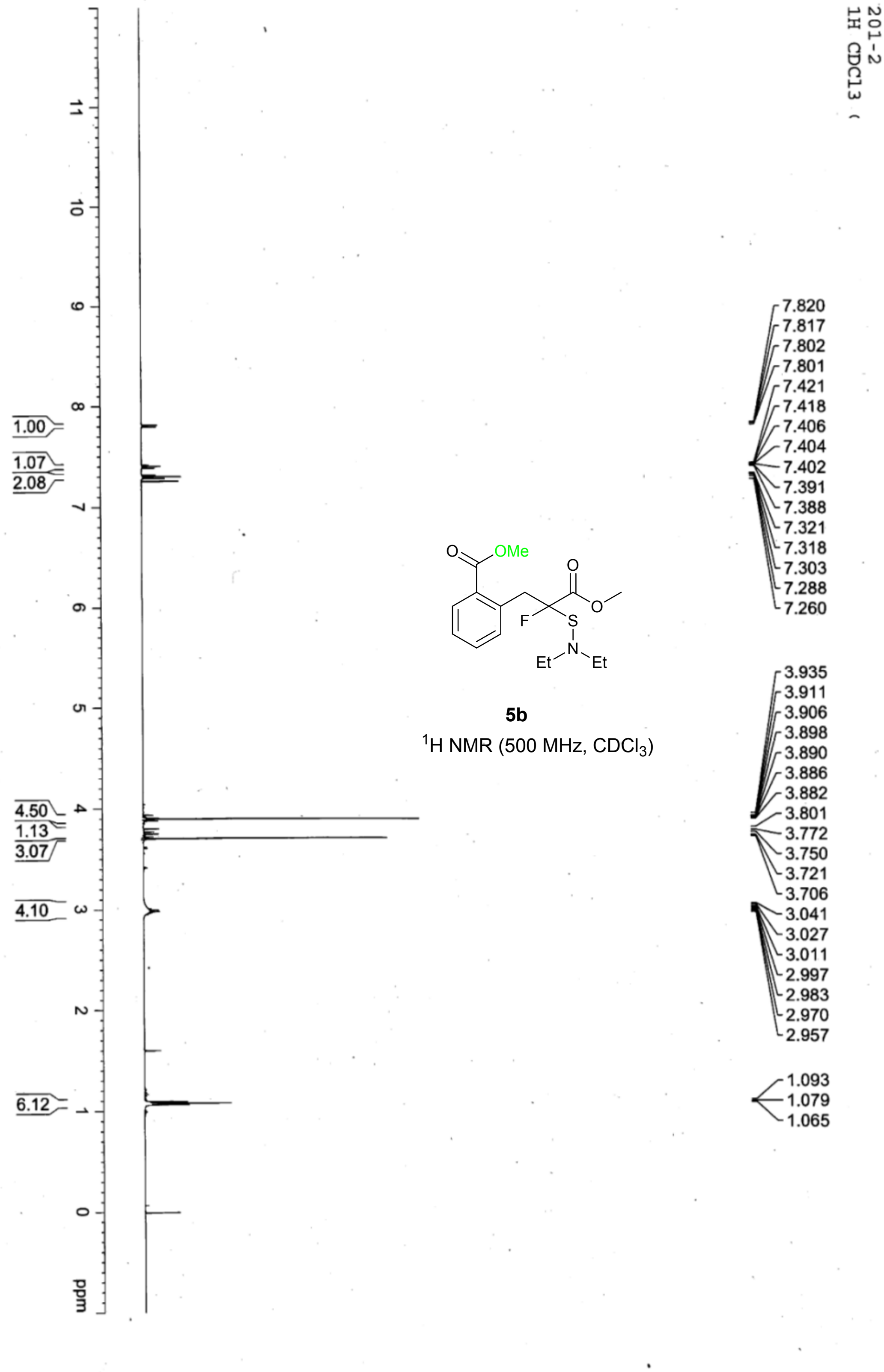

5b

^{1}H NMR (500 MHz, $CDCl_3$)

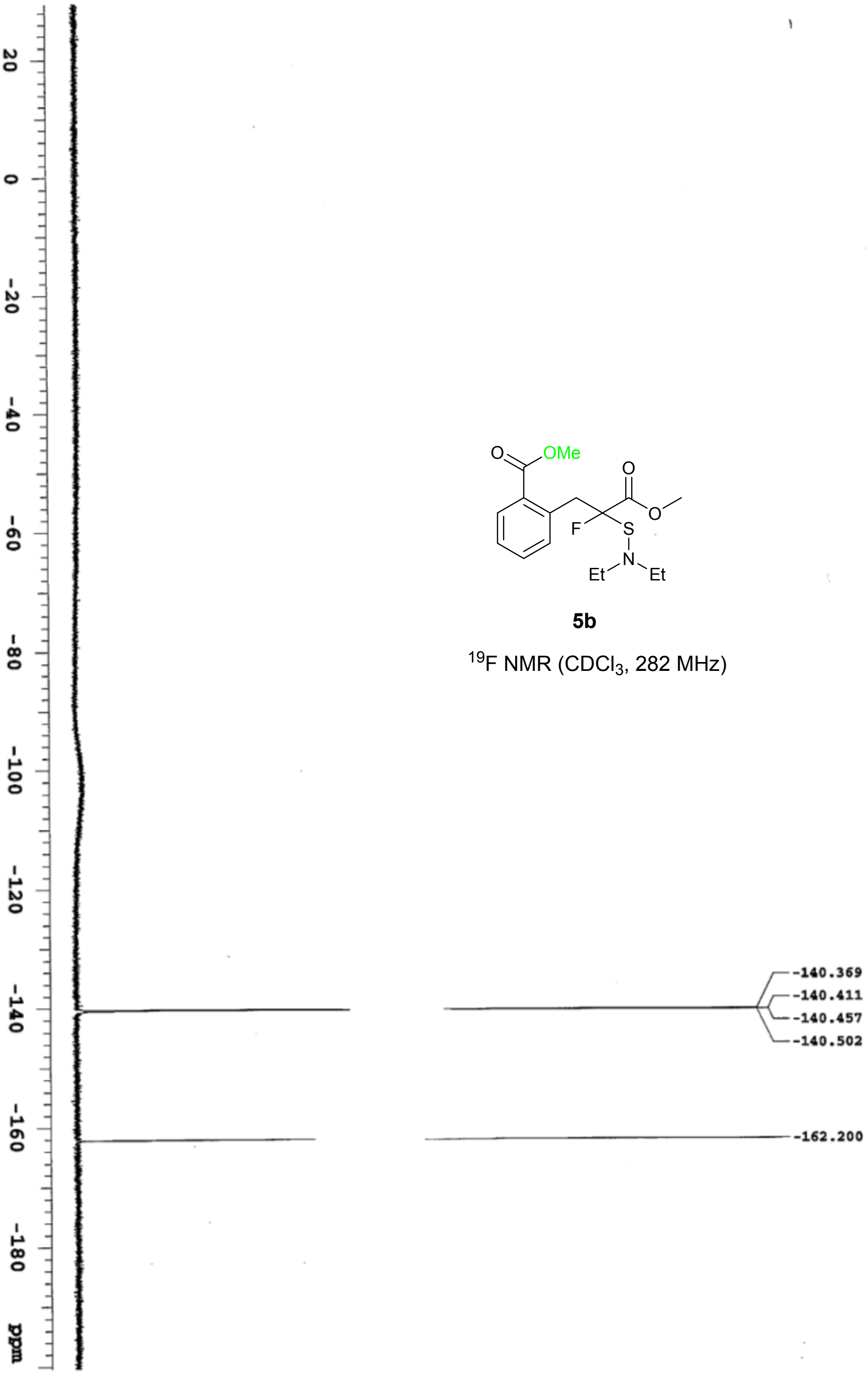

5b

^{19}F NMR ($CDCl_3$, 282 MHz)

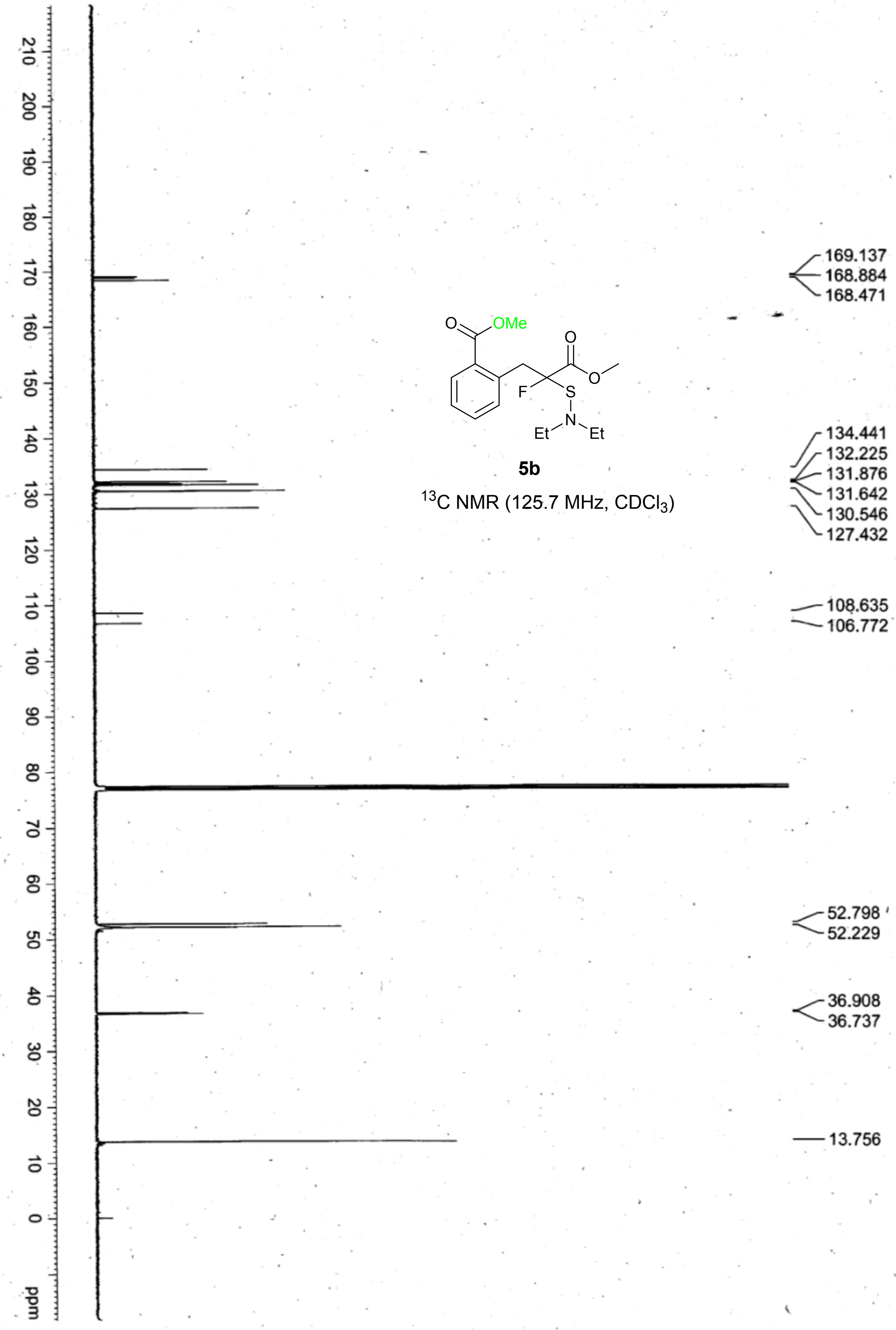

5b

^{13}C NMR (125.7 MHz, $CDCl_3$)

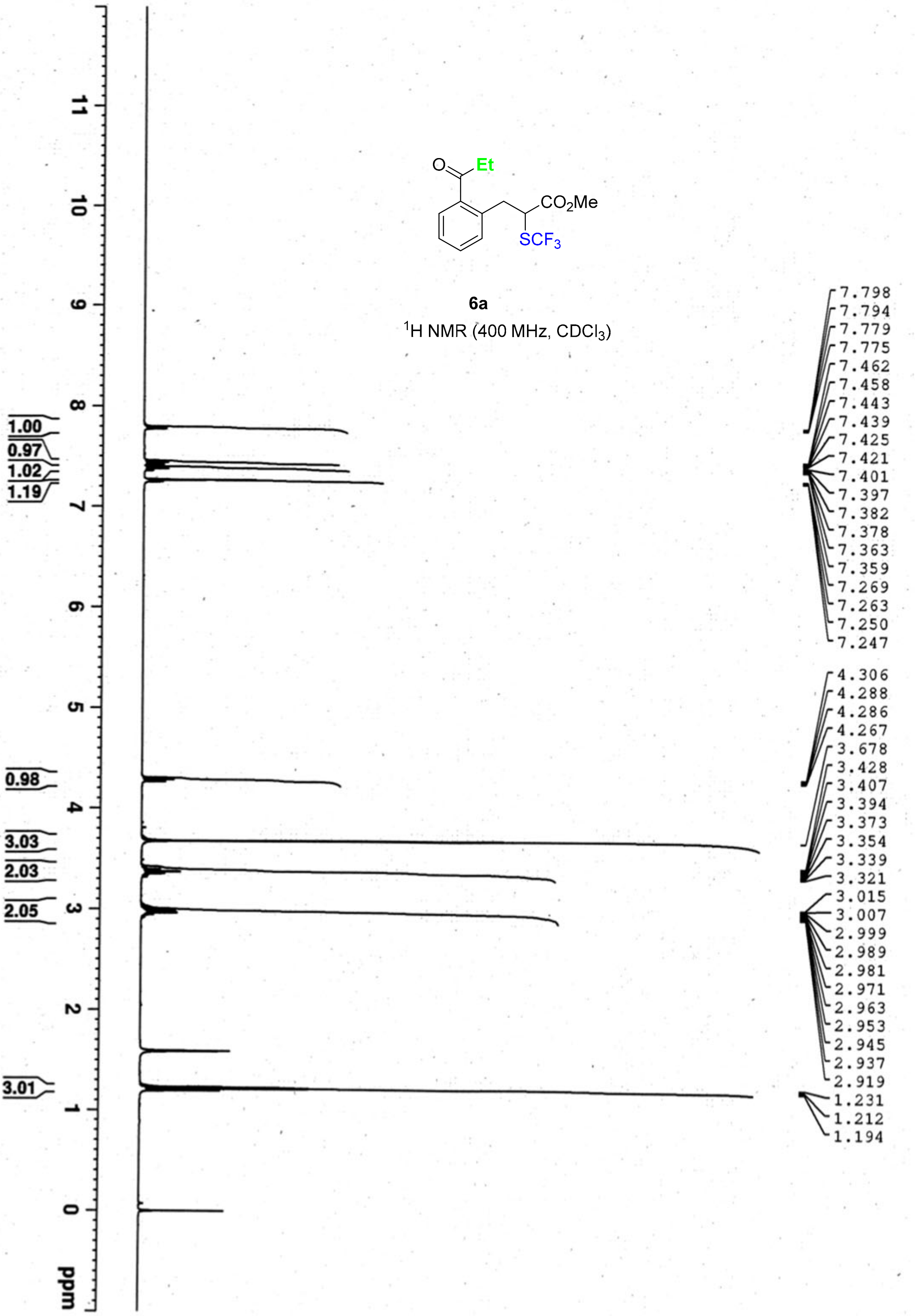
O
Et
CO2Me
SCF3
6a
1H NMR (400 MHz, CDCl3)
7.798
7.794
7.779
7.775
7.462
7.458
7.443
7.439
7.425
7.421
7.401
7.397
7.382
7.378
7.363
7.359
7.269
7.263
7.250
7.247
4.306
4.288
4.286
4.267
3.678
3.428
3.407
3.394
3.373
3.354
3.339
3.321
3.015
3.007
2.999
2.989
2.981
2.971
2.963
2.953
2.945
2.937
2.919
1.231
1.212
1.194
1.00
0.97
1.02
1.19
0.98
3.03
2.03
2.05
3.01
11
10
9
8
7
6
5
4
3
2
1
0
ppm

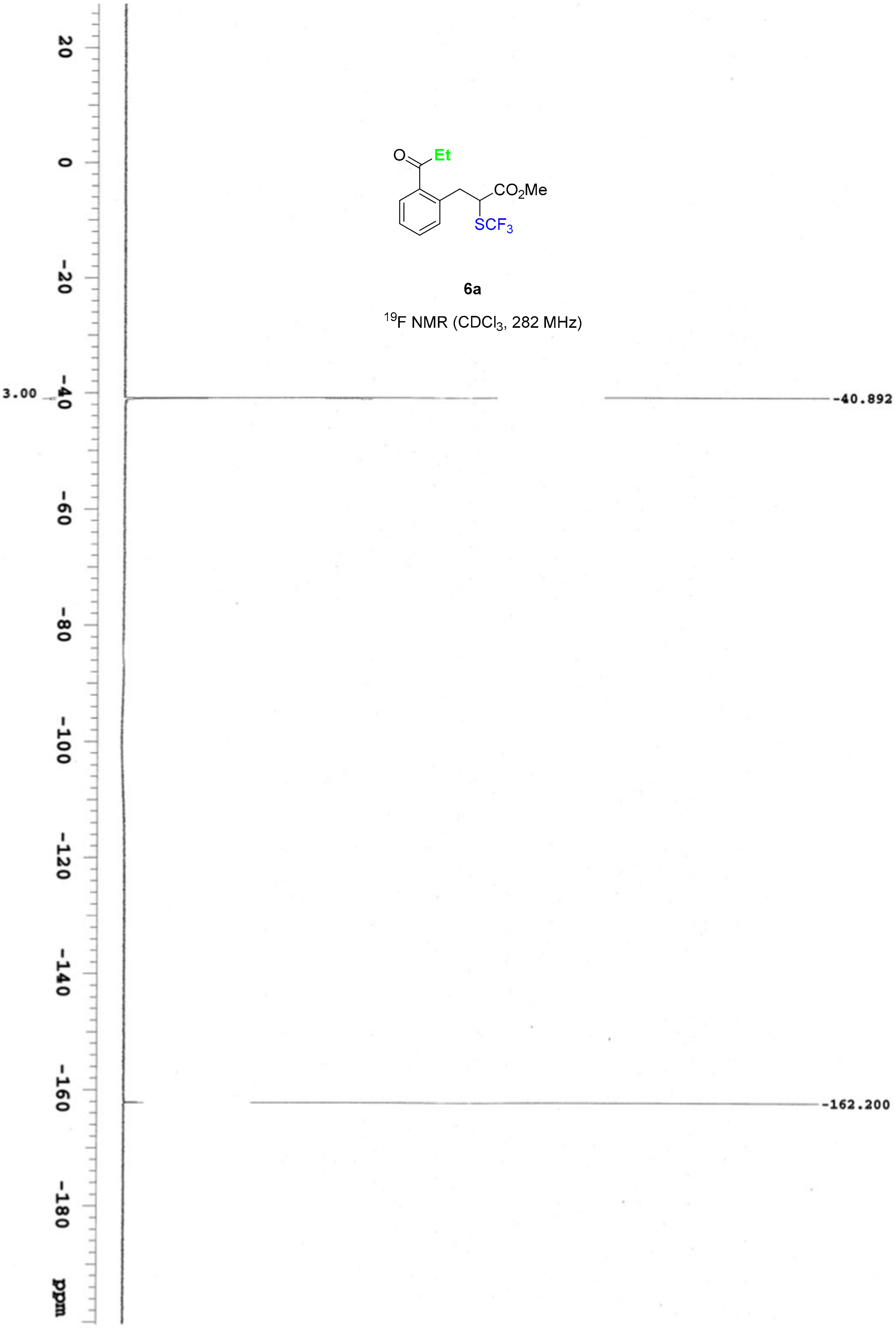

6a

^{19}F NMR ($CDCl_3$, 282 MHz)

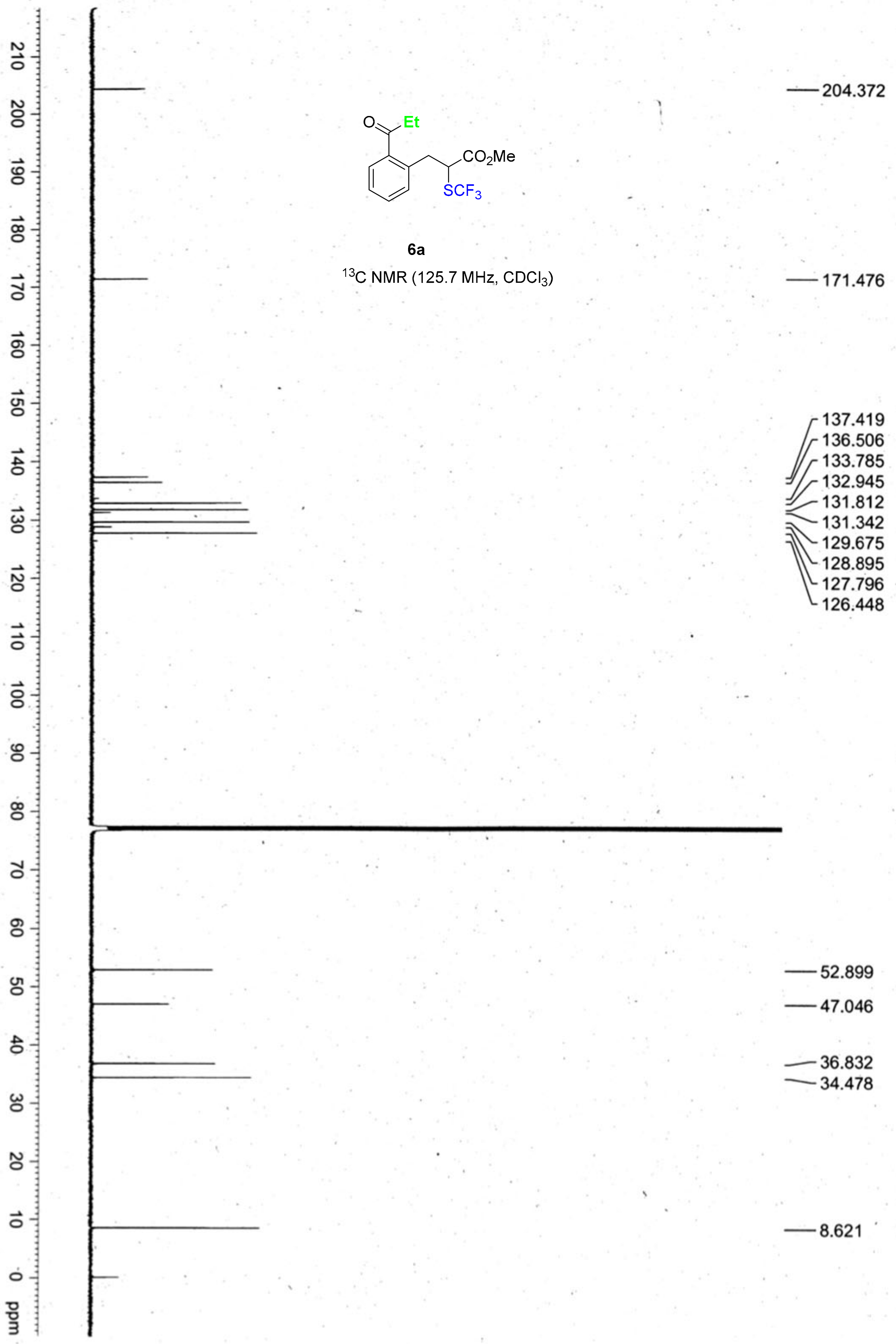

6a

^{13}C NMR (125.7 MHz, $CDCl_3$)

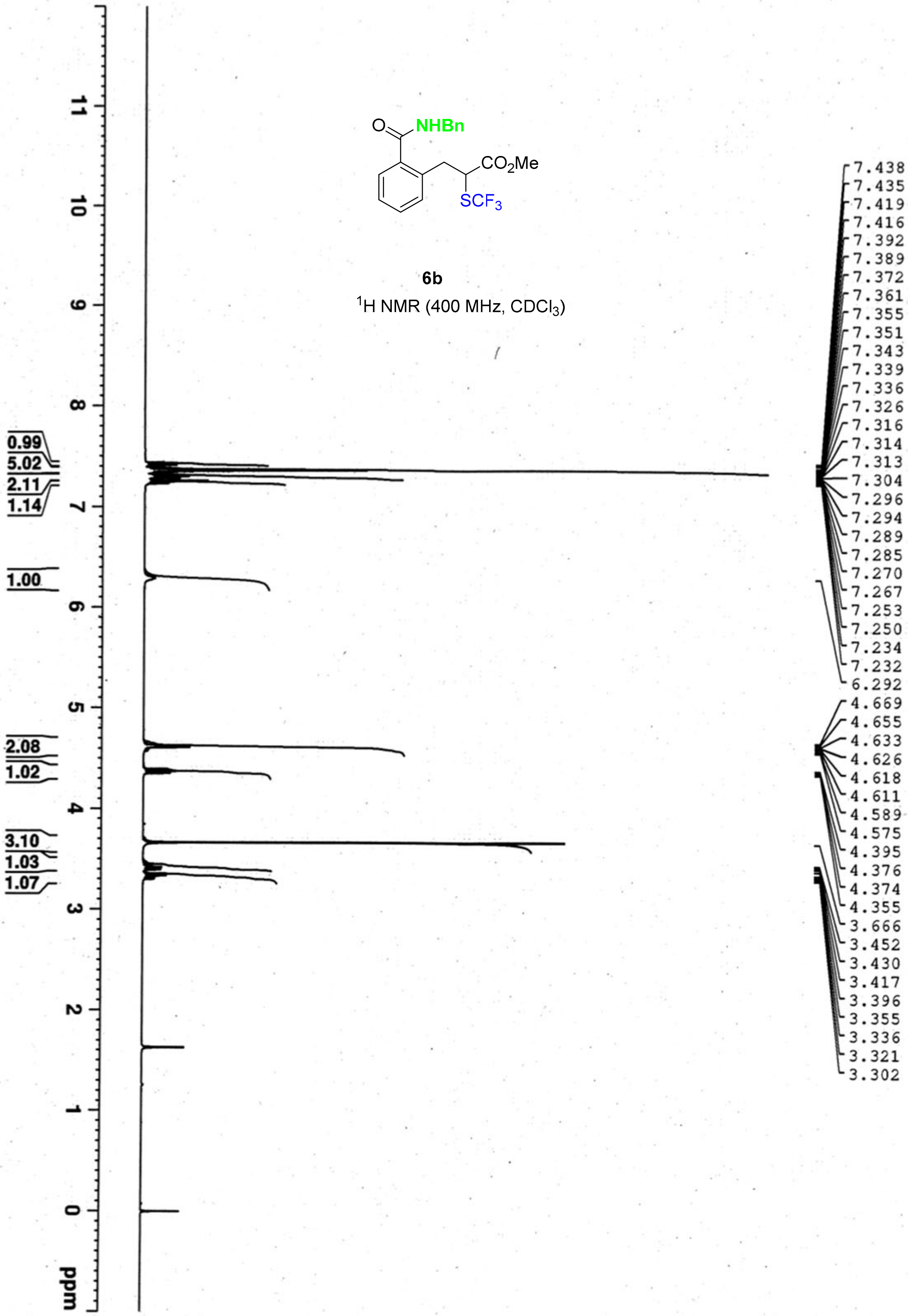

6b

^1H NMR (400 MHz, $CDCl_3$)

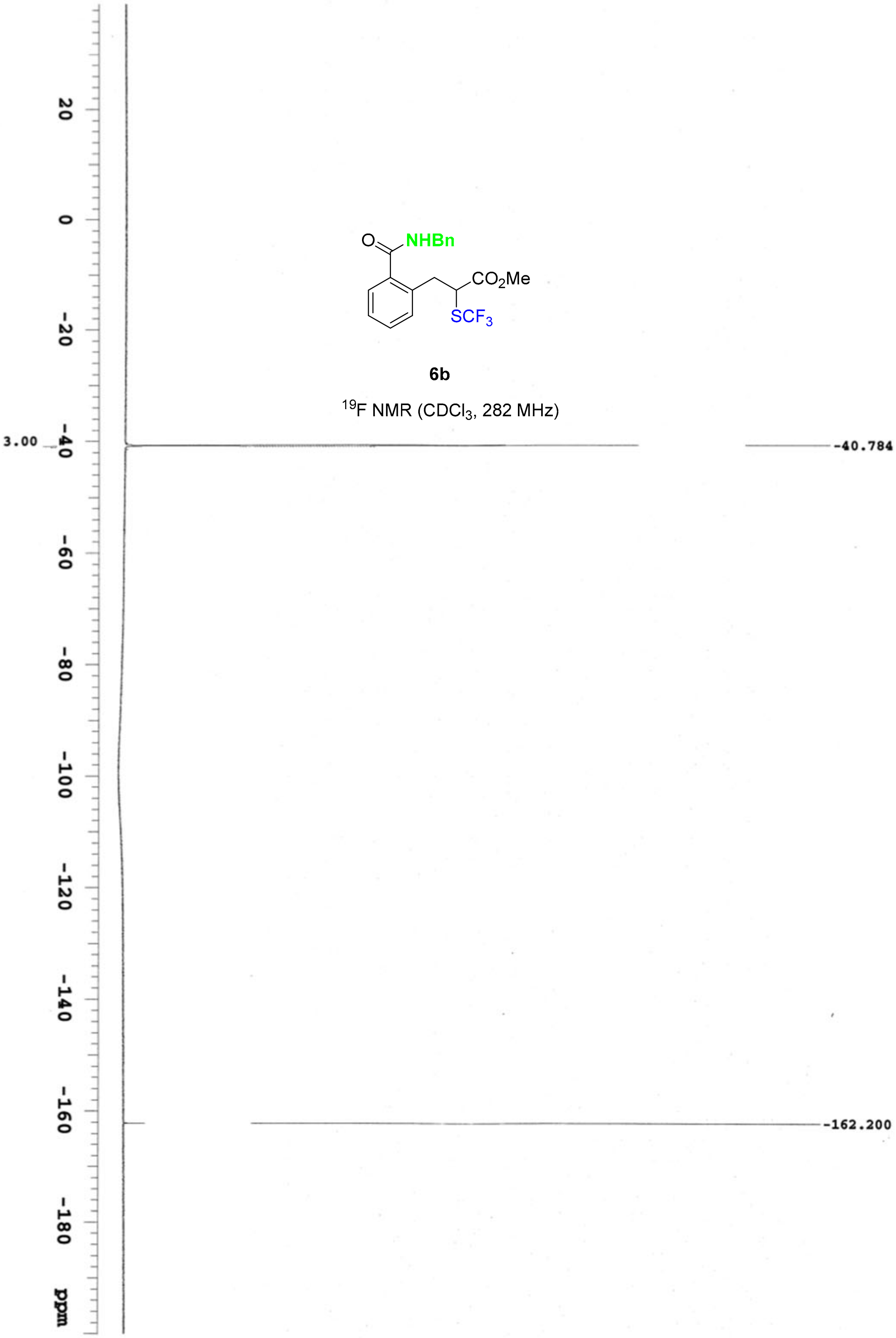

6b

^{19}F NMR ($CDCl_3$, 282 MHz)

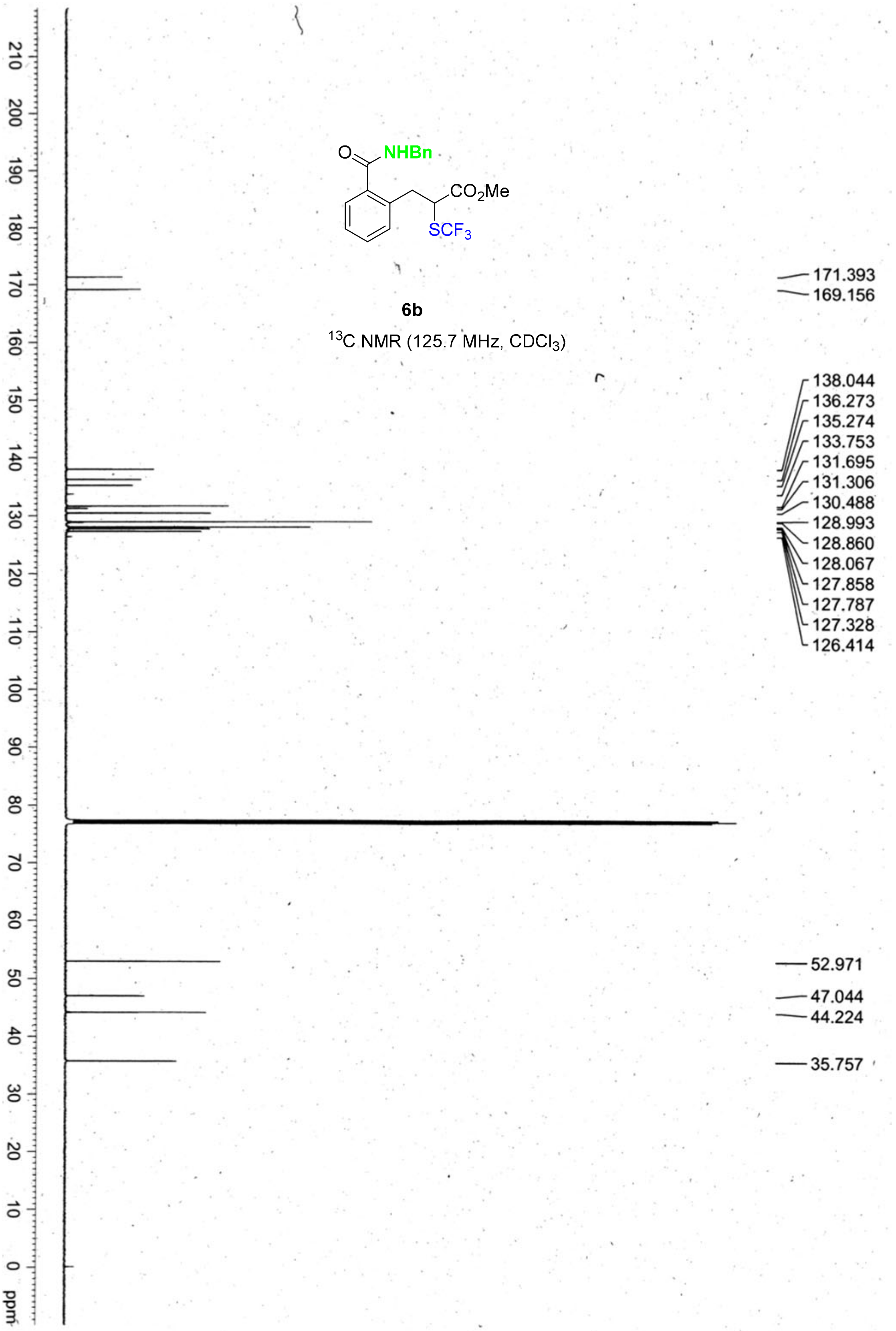

6b

^{13}C NMR (125.7 MHz, $CDCl_3$)

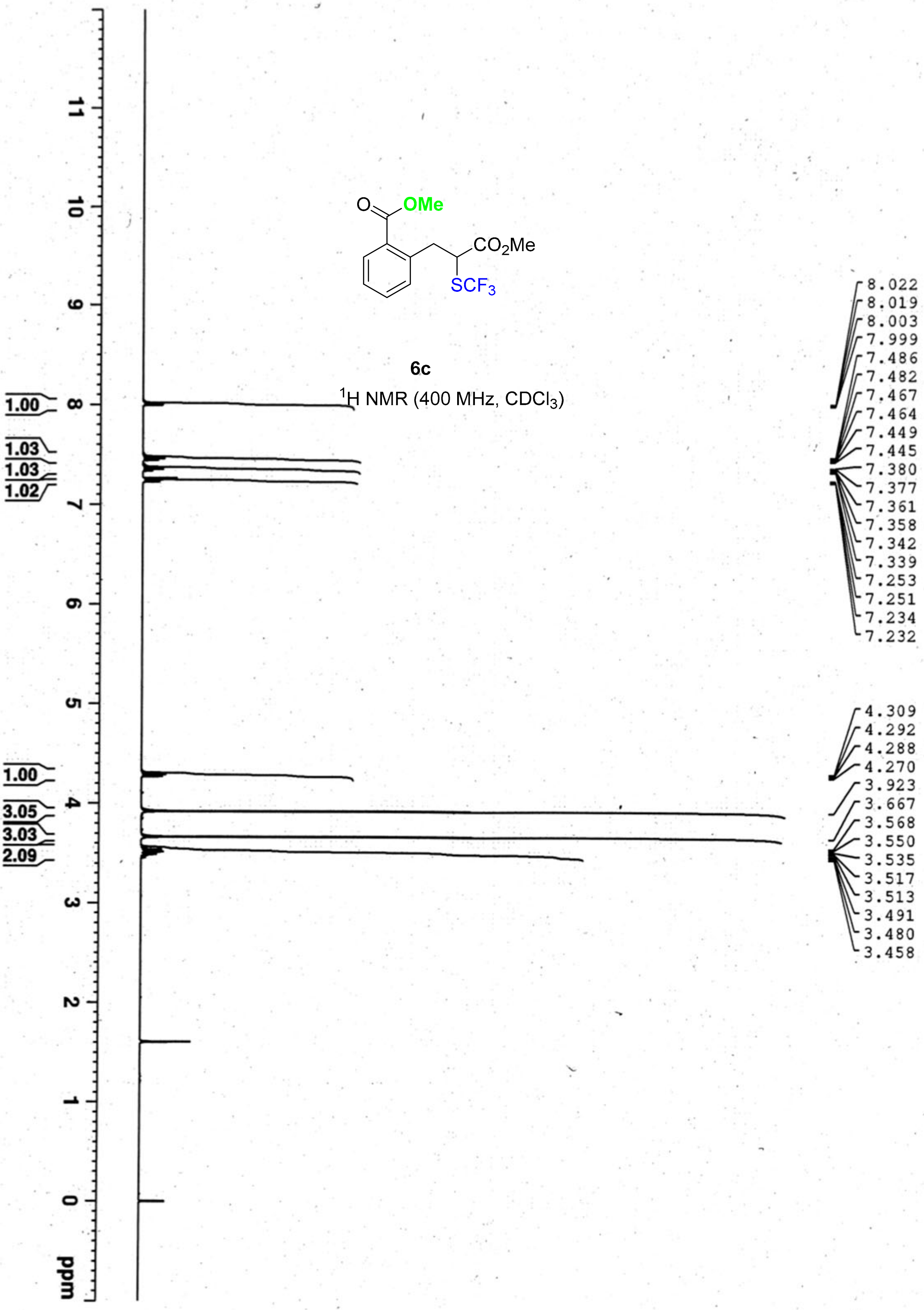

6c

^1H NMR (400 MHz, $CDCl_3$)

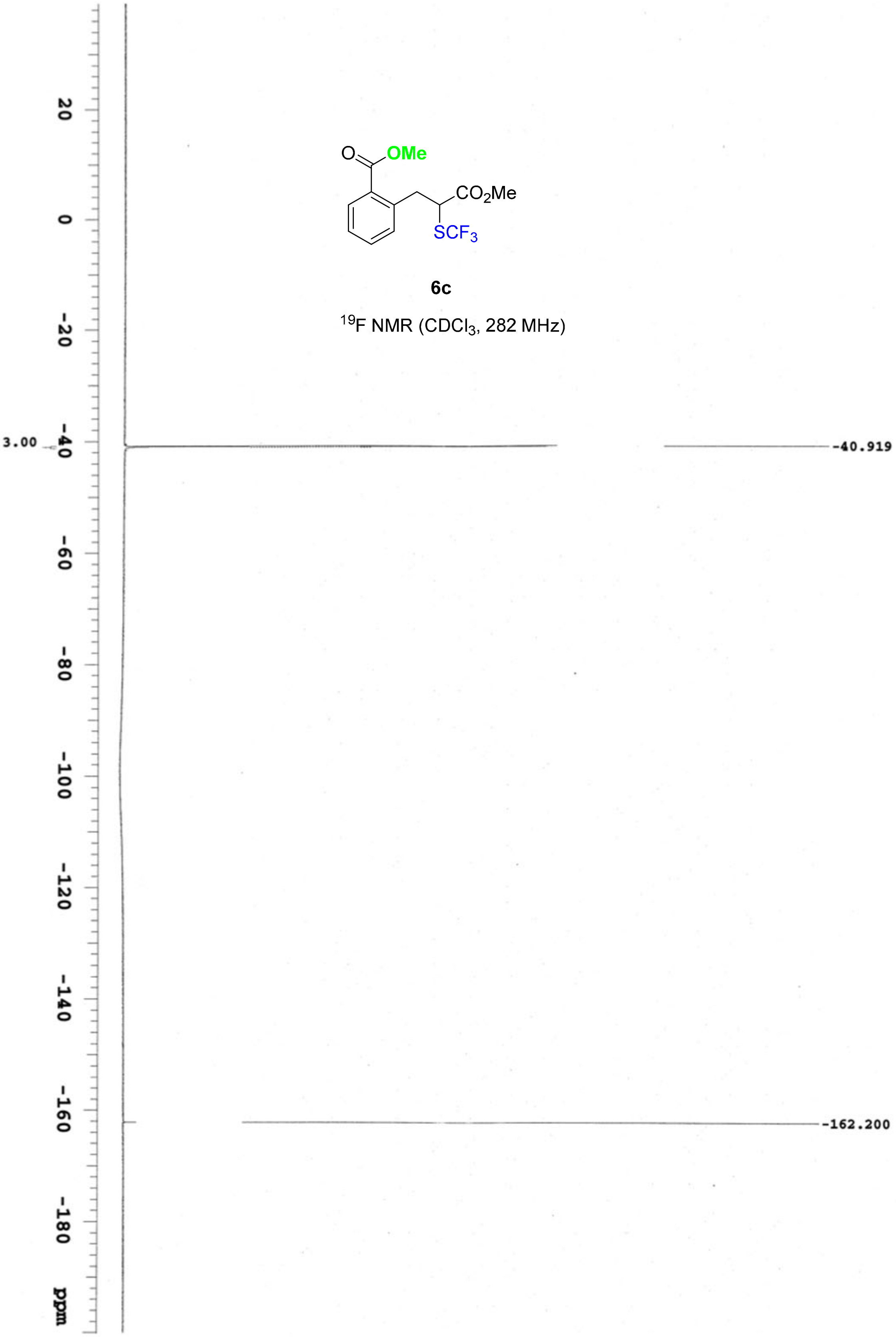

6c

^{19}F NMR ($CDCl_3$, 282 MHz)

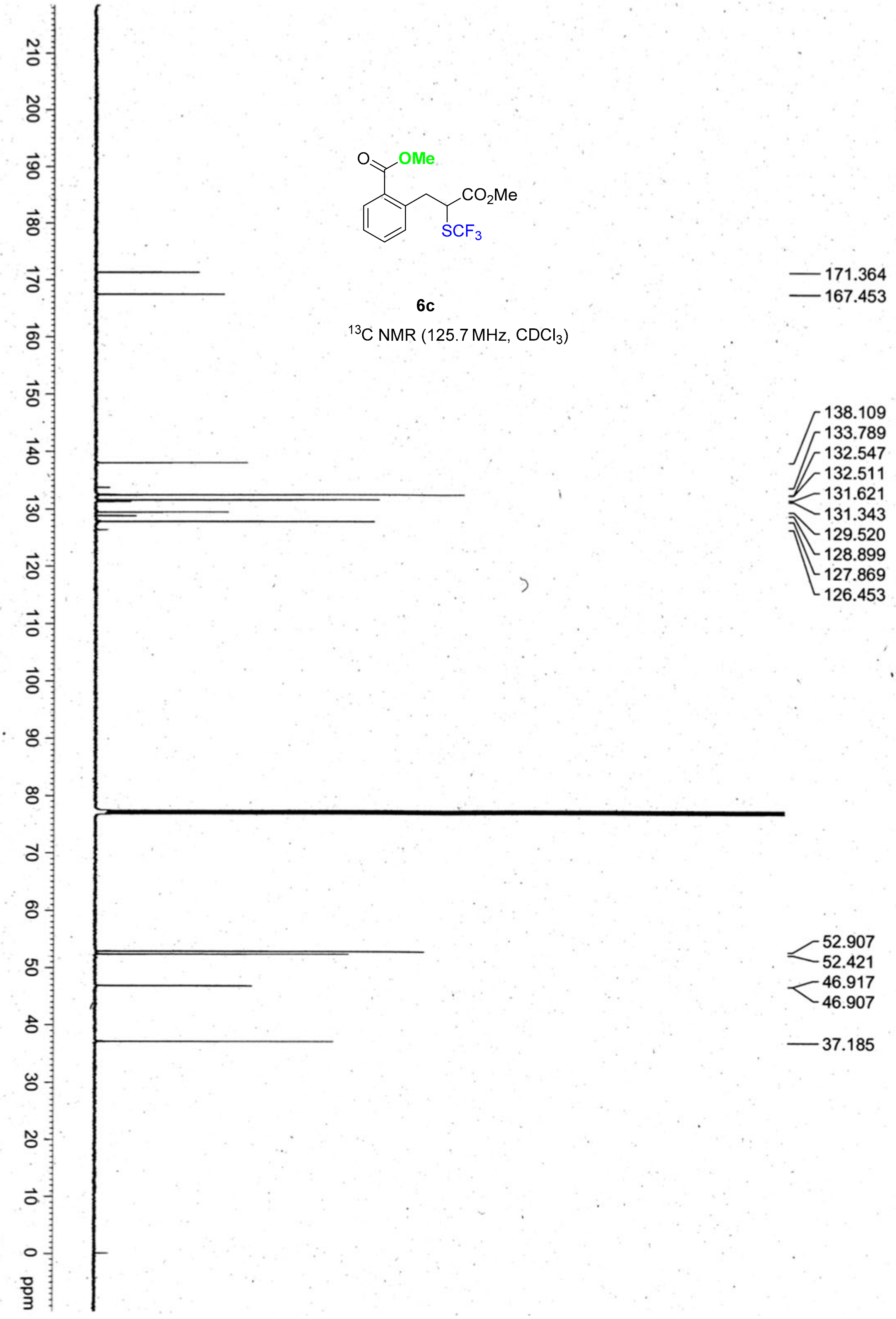

6c

^{13}C NMR (125.7 MHz, $CDCl_3$)

23. ^{1}H NMR, ^{13}C NMR and ^{19}F NMR spectra for product 7b (Scheme 3)

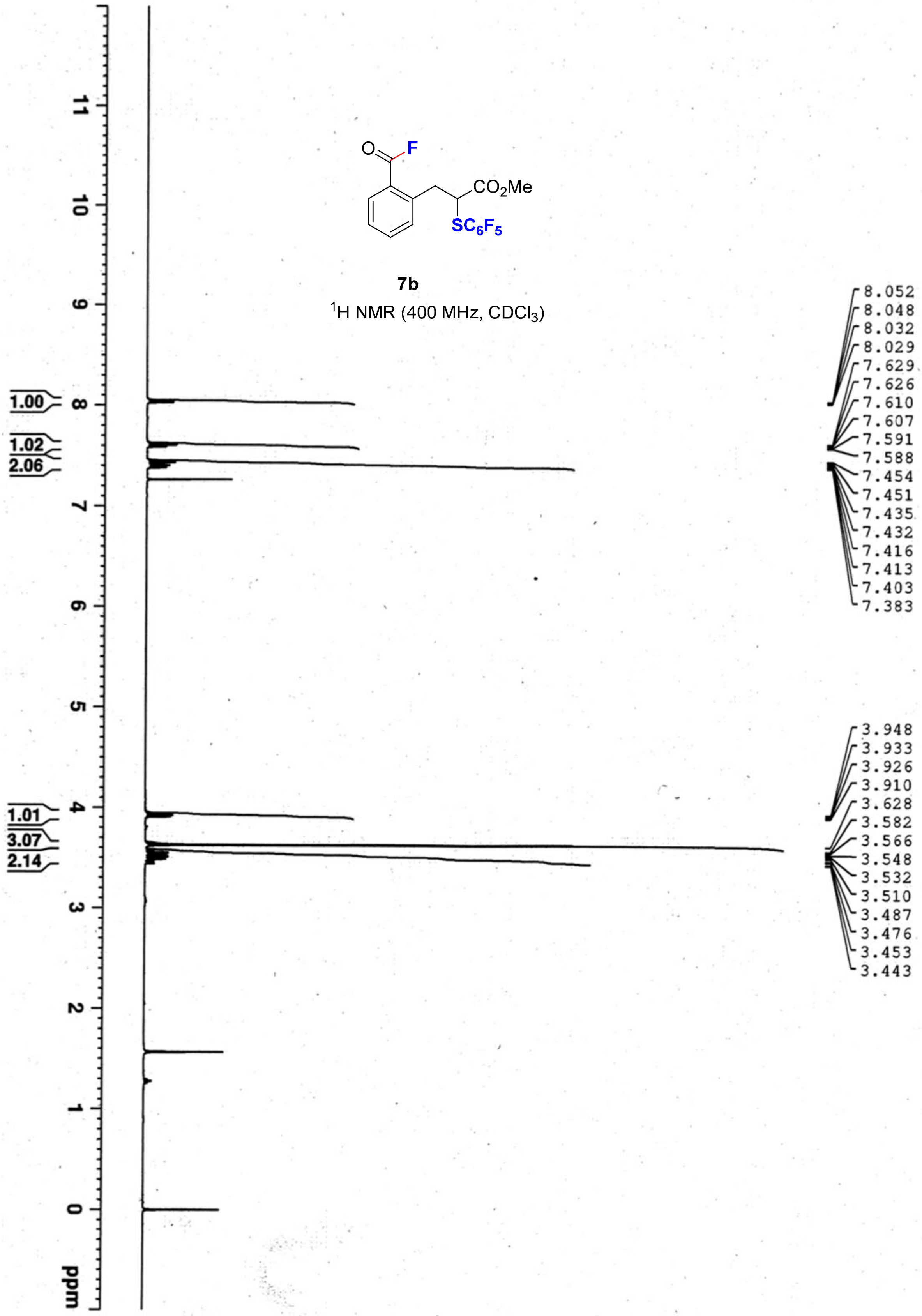

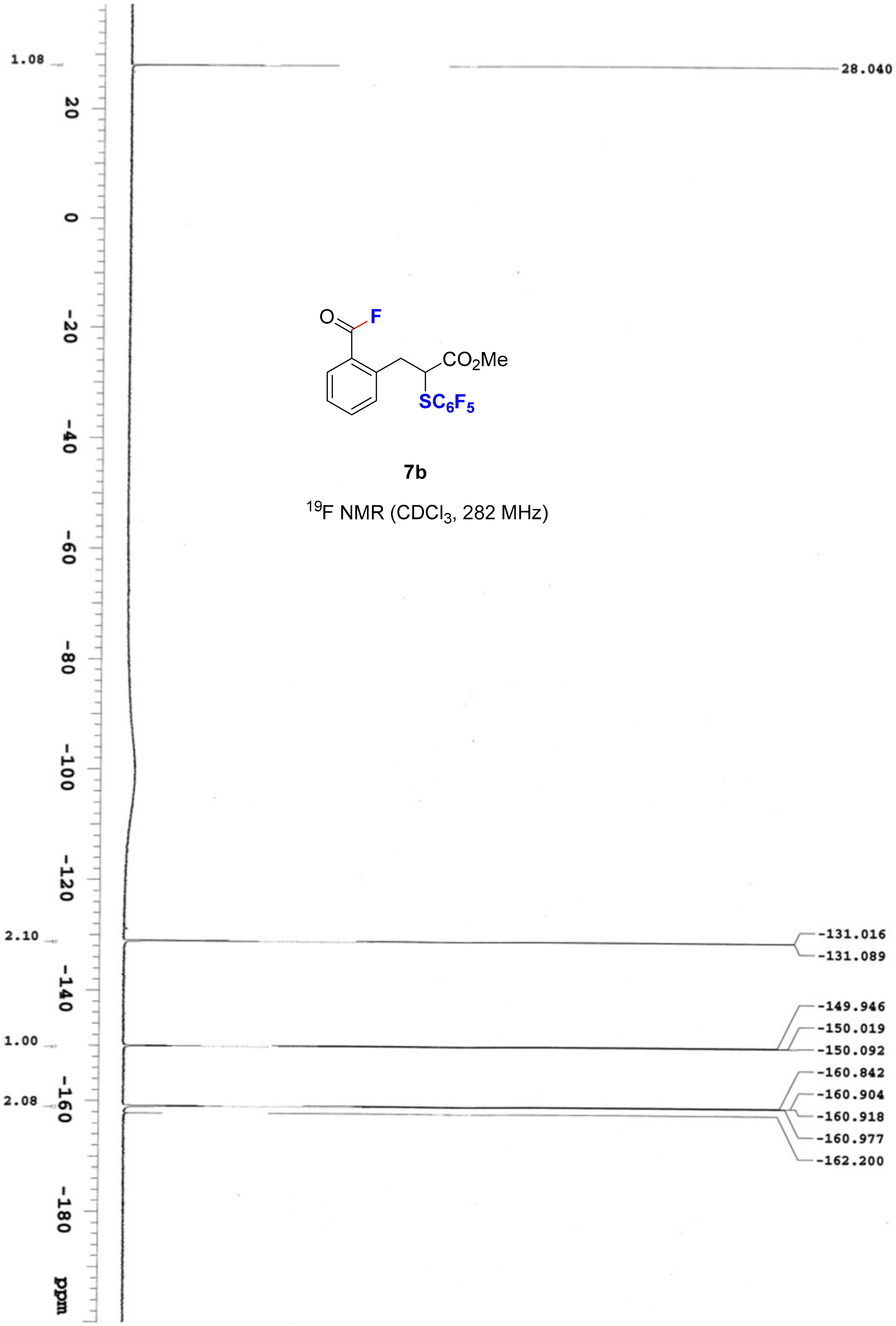

7b

^{19}F NMR ($CDCl_3$, 282 MHz)

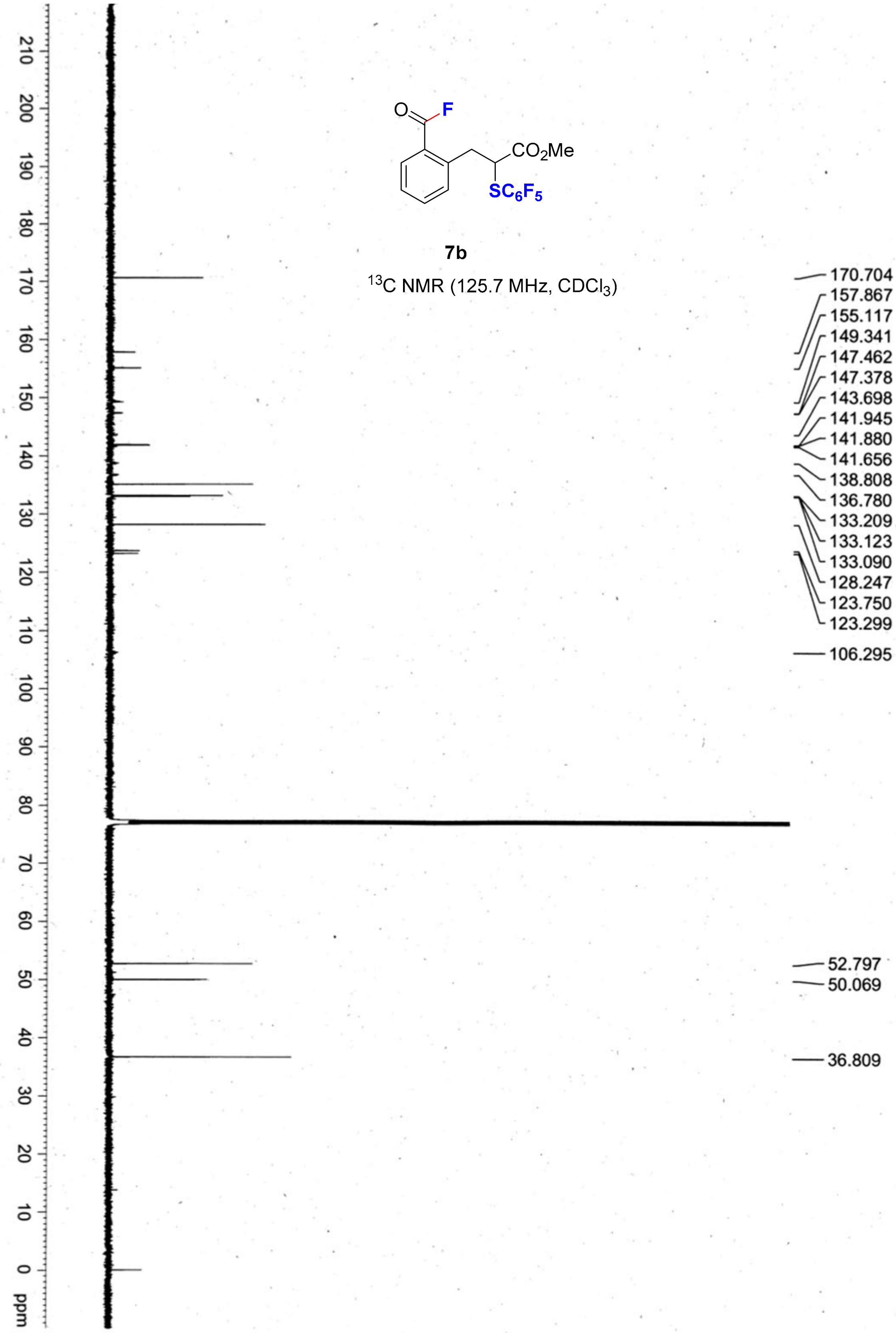

7b

^{13}C NMR (125.7 MHz, $CDCl_3$)

24. ^{1}H NMR, ^{13}C NMR and ^{19}F NMR spectra for product 9 and 10 (Scheme 4);

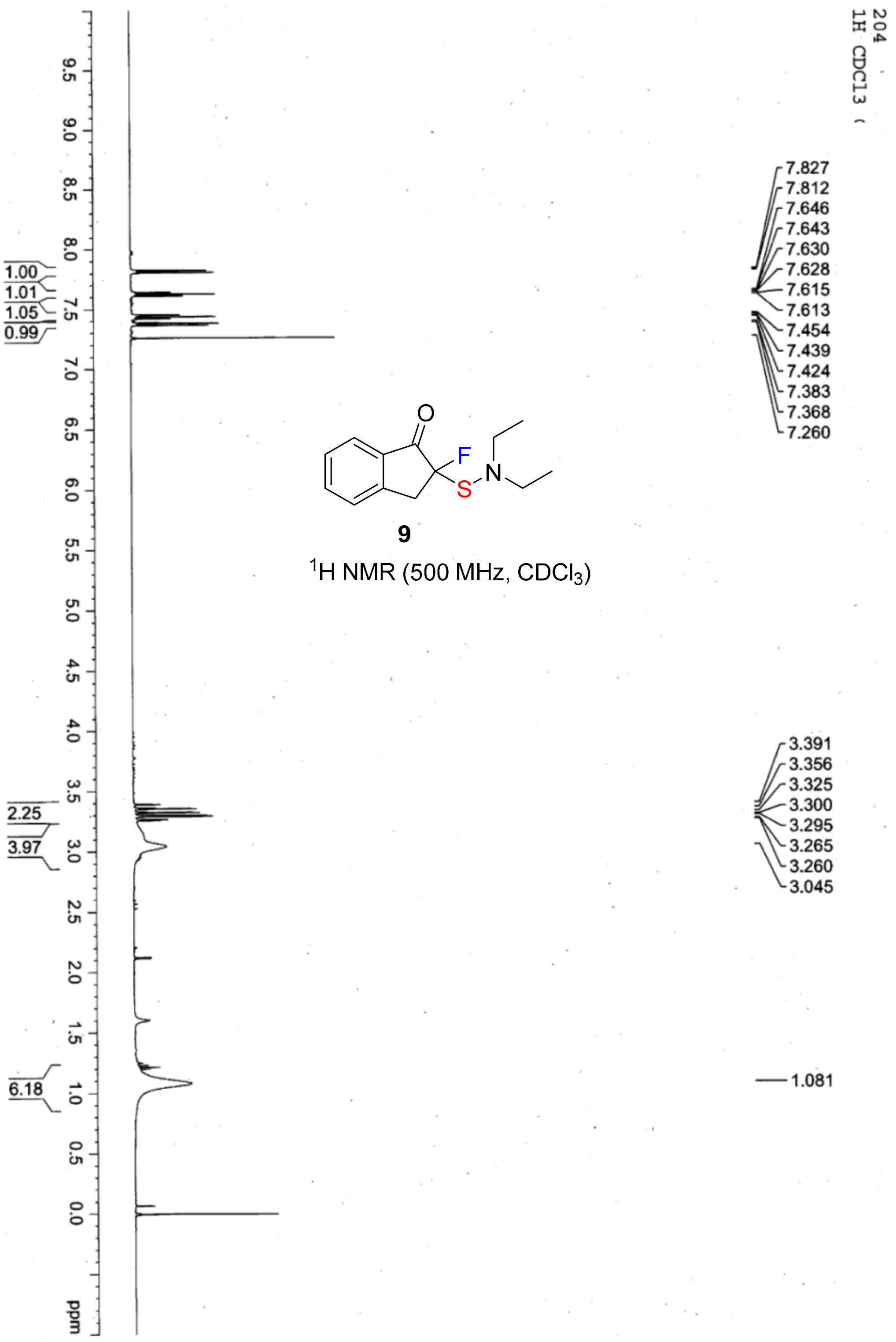

9

^{1}H NMR (500 MHz, $CDCl_3$)

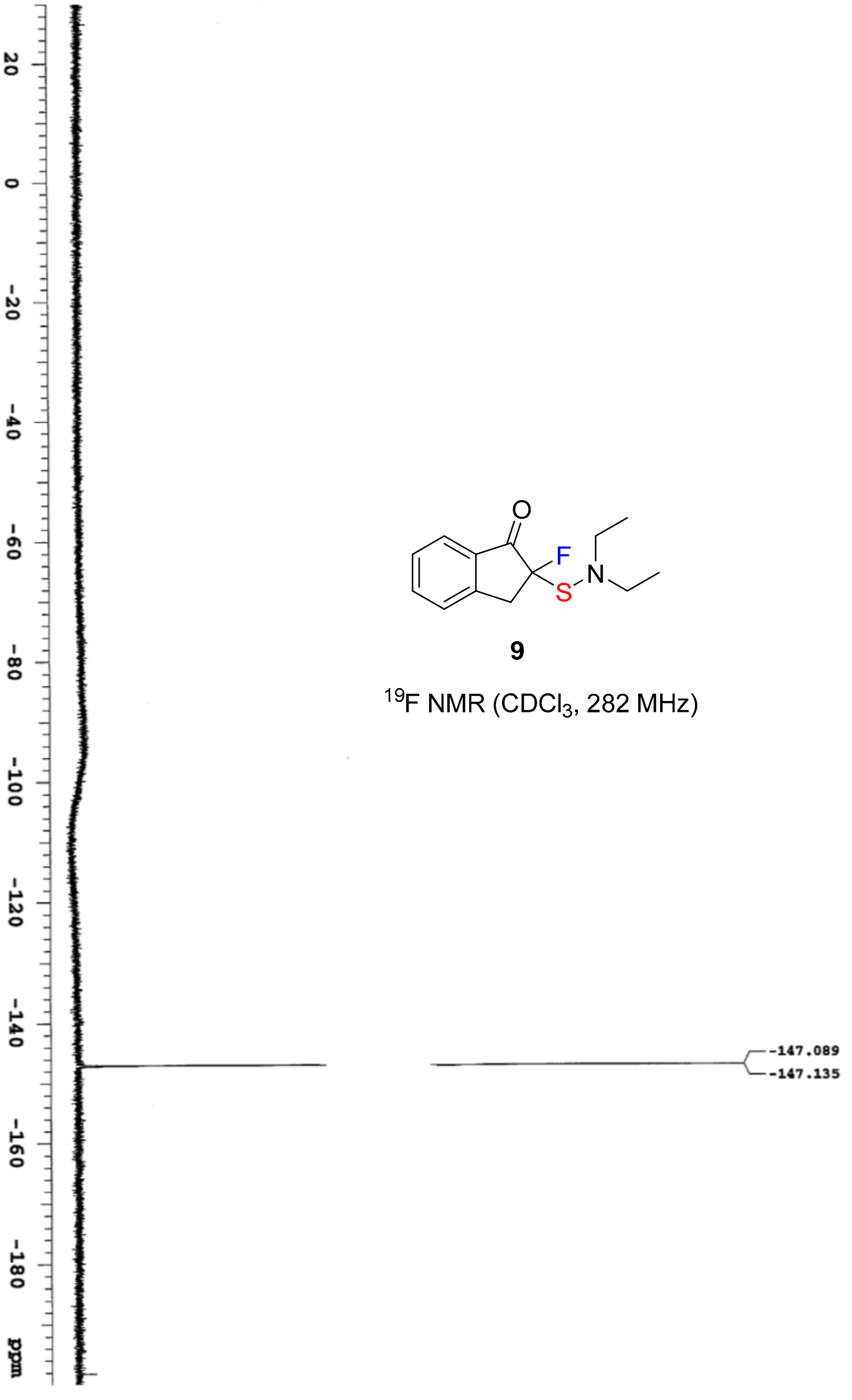

9

^{19}F NMR ($CDCl_3$, 282 MHz)

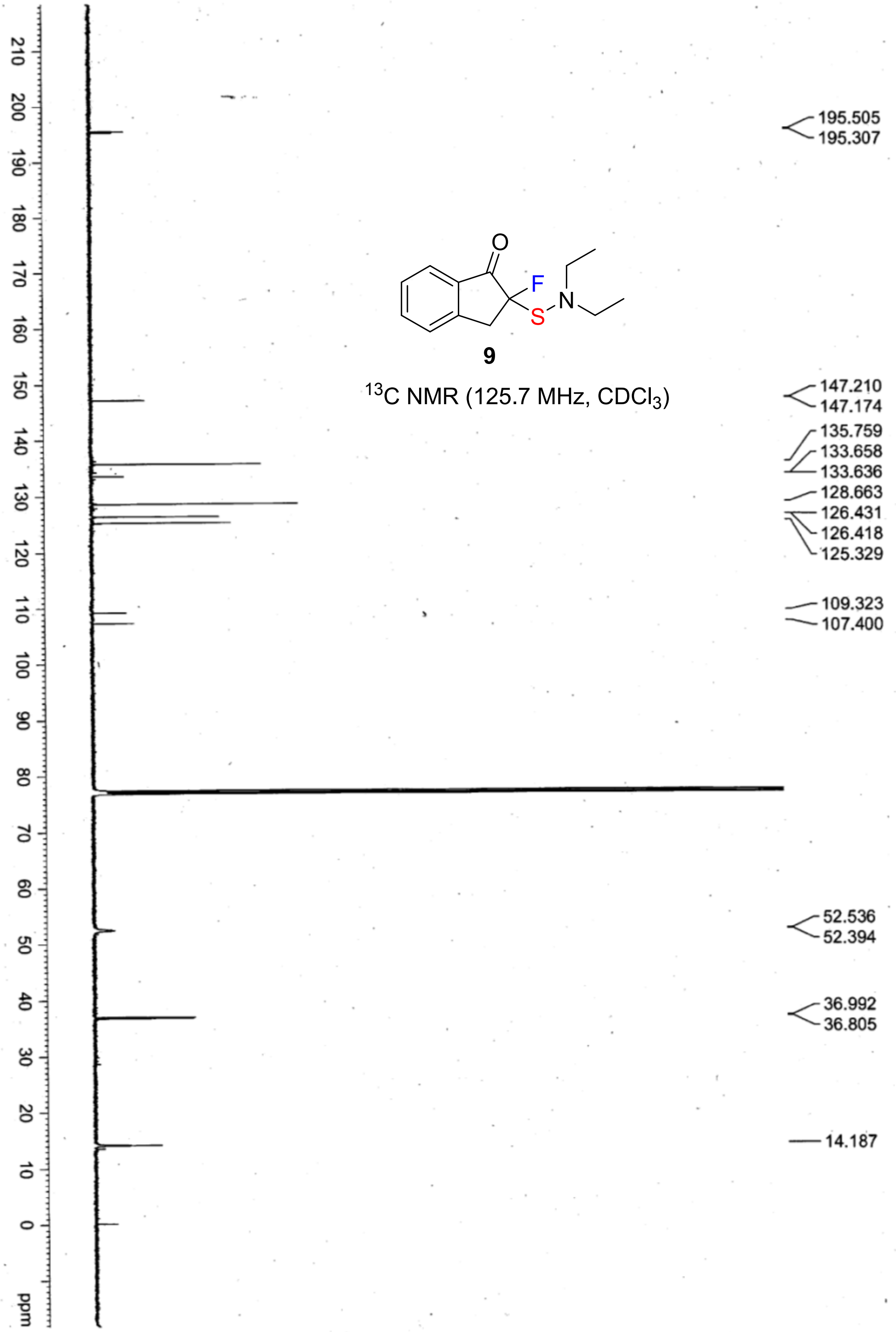

9

^{13}C NMR (125.7 MHz, $CDCl_3$)

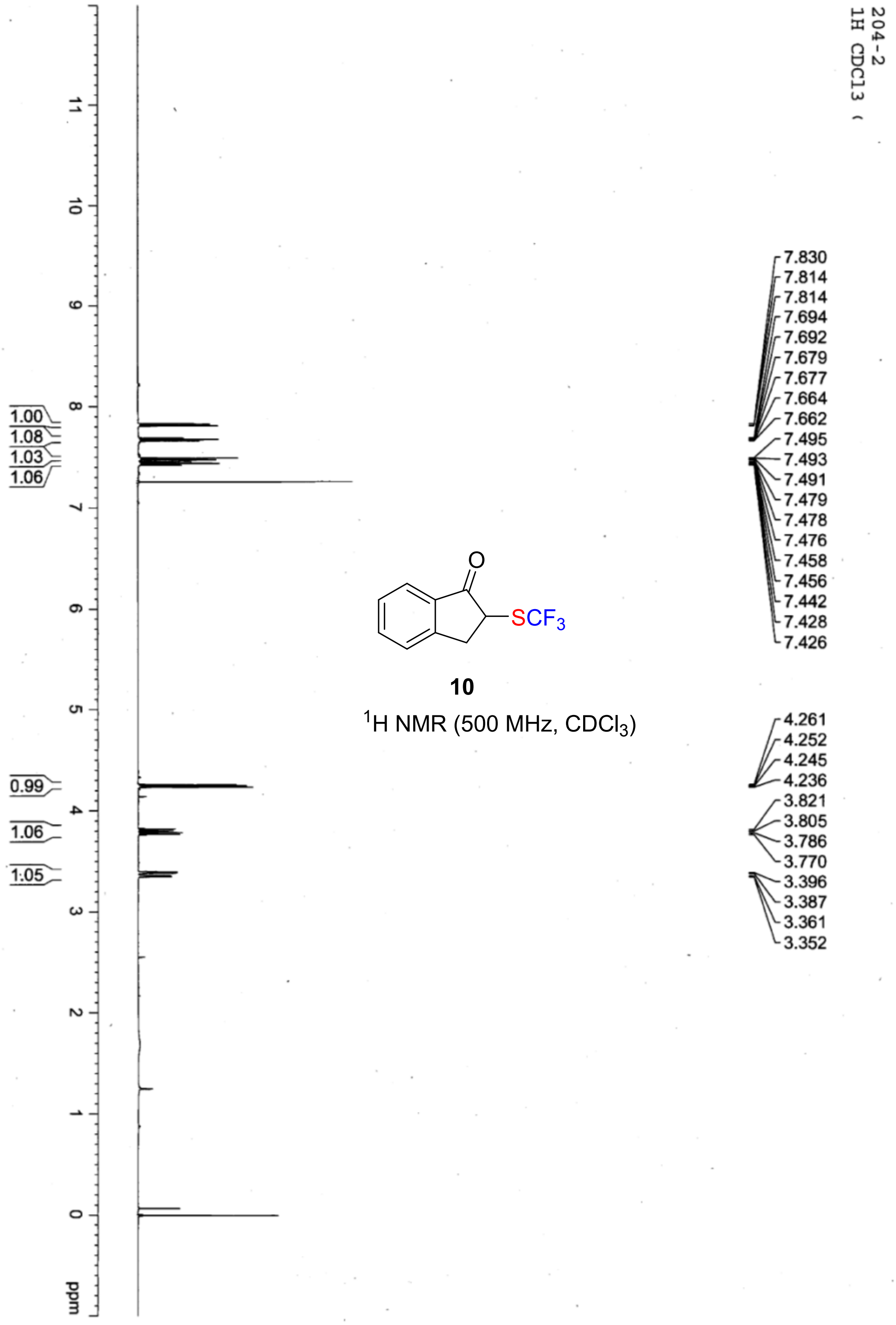

10

^{1}H NMR (500 MHz, $CDCl_3$)

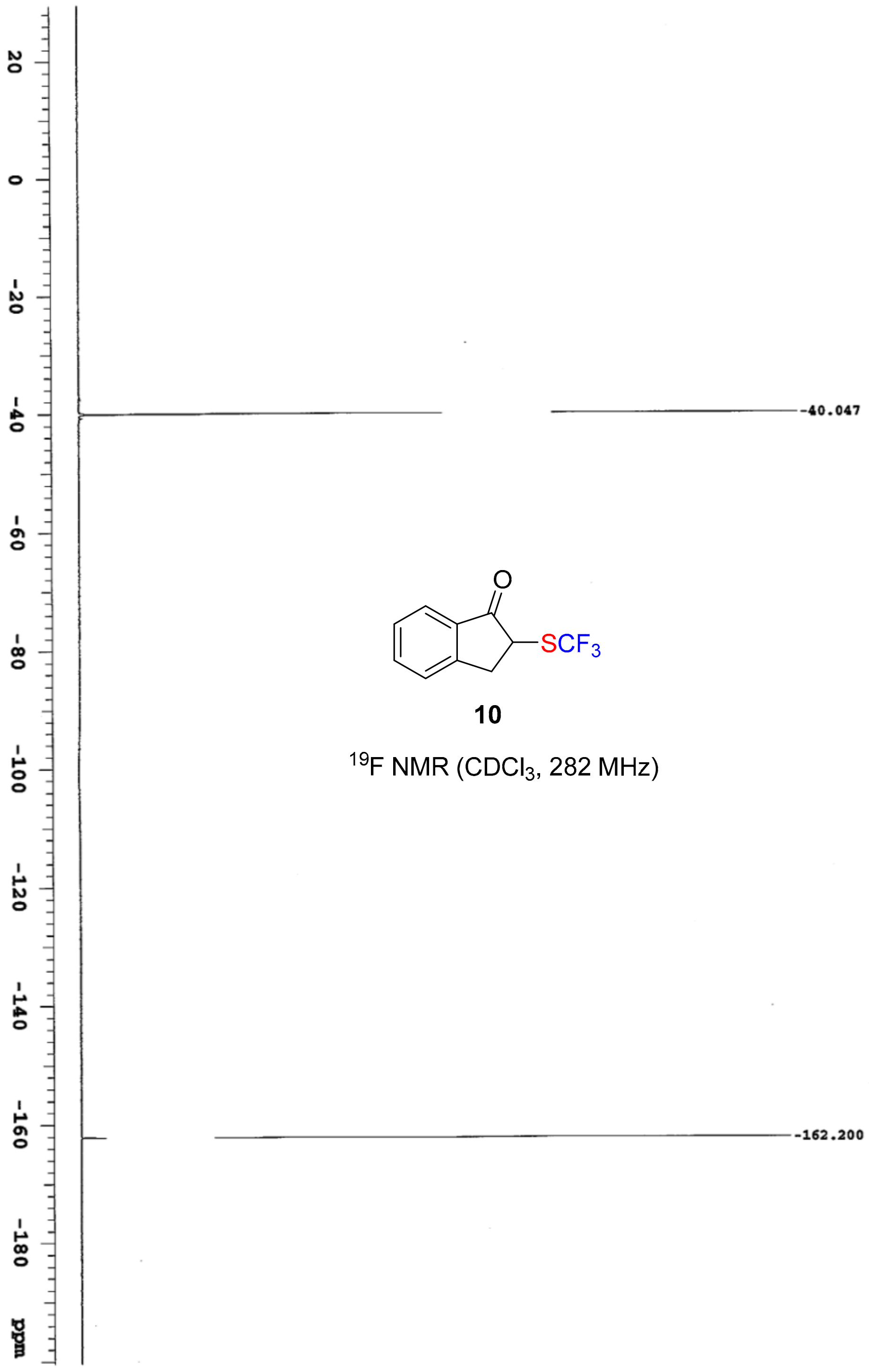

10

^{19}F NMR ($CDCl_3$, 282 MHz)

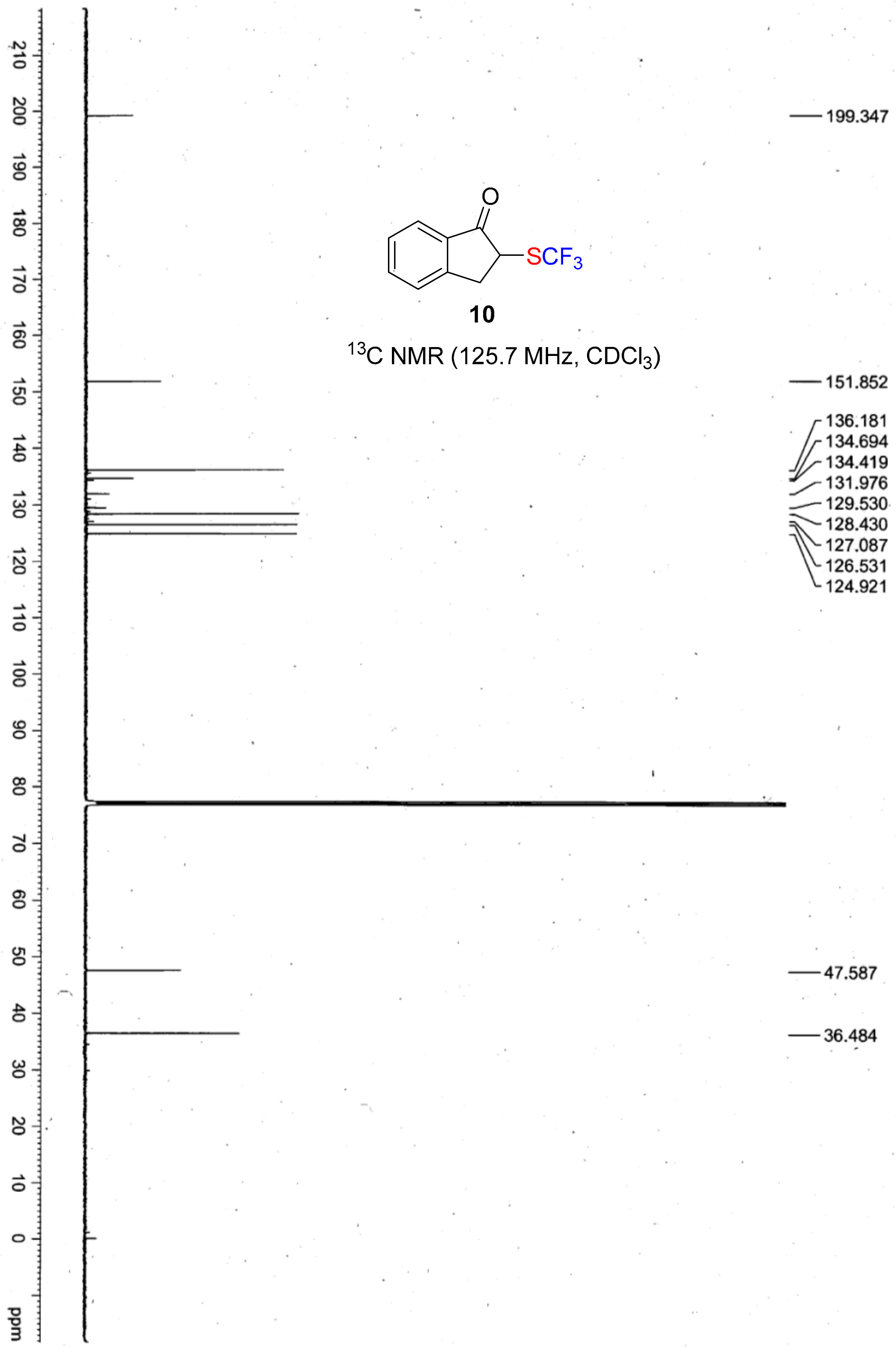
O
SCF3
10
13C NMR (125.7 MHz, CDCl3)
199.347
151.852
136.181
134.694
134.419
131.976
129.530
128.430
127.087
126.531
124.921
47.587
36.484
210
200
190
180
170
160
150
140
130
120
110
100
90
80
70
60
50
40
30
20
10
0
ppm
